# The genotypic and phenotypic landscape of *PDHA1*-related pyruvate dehydrogenase complex deficiency

**DOI:** 10.1093/brain/awaf430

**Published:** 2025-11-14

**Authors:** Kajus Merkevicius, Dmitrii Smirnov, Lea D Schlieben, Rebecca Ganetzky, René G Feichtinger, Huafang Jiang, Fang Fang, Tomohiro Ebihara, Kei Murayama, Giulia Ferrera, Anna Ardissone, Dariusz Rokicki, Dorota Wesol-Kucharska, Sabine Schröder, Peter Bauer, Aida Bertoli-Avella, Elsebeth Østergaard, Peter Freisinger, Mirian C H Janssen, Matias Wagner, Omar Abouyousef, Bader Alhaddad, Lama AlAbdi, Fowzan Alkuraya, Charlotte L Alston, Anna Baghdasaryan, Diana Barca, Ivo Barić, Marcello Bellusci, Andrea Bevot, Eugen Boltshauser, Ingo Borggraefe, Juliette Bouchereau, Claudio Bruno, Birute Burnyte, Amy Calhoun, Kari Casas, Mahmut Coker, Ellen Crushell, Pascal De Lonlay, Carlo Dionisi-Vici, Felix Distelmaier, Marni J Falk, Ana Cristina Ferreira, Carlos R Ferreira, Can Ficicioglu, Gulden Fatma Gokçay, Johannes Häberle, Oliver Heath, Albrecht Hellenschmidt, Julia Hoefele, Georg F Hoffmann, Tomas Honzik, Martina Huemer, Patrícia Janeiro, Amel Karaa, Çiğdem Seher Kasapkara, Ilse Kern, Joerg Klepper, Thomas Klopstock, Ina Knerr, Johannes Koch, Zita Krumina, Costanza Lamperti, Elise Lebigot, Zhimei Liu, Esther M Maier, Diego Martinelli, Robert McFarland, Bryce Mendelsohn, Maria Judit Molnar, Helen Mundy, Marie Cecile Nassogne, Anabela Oliveira, Katrin Õunap, Chiara Panicucci, Sumit Parikh, Heidi Peters, Samia Pichard, Barbara Plecko, Danijela P Ramadža, Gabriela M Repetto, Isabel Rivera, Richard J Rodenburg, Alessandro Rossi, Manuel Schiff, Kathrin Seidemann, Wendy E Smith, Sérgia Soares, Barbara Siri, Katja Steinbrucker, Pasquale Striano, Jolanta Sykut-Cegielska, Galit Tal, Robert W Taylor, Konstantinos Tsiakas, Sema Kalkan Ucar, Eva Hoytema van Konijnenburg, Mathias Woidy, Joy Yaplito-Lee, Yilmaz Yildiz, Martin Zenker, Petra Zsidegh, Dominik Westphal, Wolfgang Sperl, Thomas Meitinger, Garry K Brown, Holger Prokisch, Johannes A Mayr, Saskia B Wortmann

**Affiliations:** Expertise Centre for Mitochondrial Diseases (Mitohaus), University Children’s Hospital, Paracelsus Medical University (PMU) Salzburg, Salzburg 5020, Austria; Faculty of Medicine, Clinic of Paediatrics, Institute of Clinical Medicine, Vilnius University, Vilnius 03101, Lithuania; Institute of Biosciences, Life Sciences Center, Vilnius University, Vilnius 10257, Lithuania; School of Medicine, Institute of Human Genetics, Klinikum Rechts der Isar, Technical University of Munich, Munich 81675, Germany; Institute of Neurogenomics, Computational Health Centre, Helmholtz Zentrum München, Neuherberg 85764, Germany; School of Medicine, Institute of Human Genetics, Klinikum Rechts der Isar, Technical University of Munich, Munich 81675, Germany; Institute of Neurogenomics, Computational Health Centre, Helmholtz Zentrum München, Neuherberg 85764, Germany; Department of Pediatrics, Division of Human Genetics, Mitochondrial Medicine Frontier Program, Children’s Hospital of Philadelphia, Philadelphia, PA 19104, USA; Department of Pediatrics, University of Pennsylvania Perelman School of Medicine, Philadelphia, PA 19104, USA; Center for Computational Genomic Medicine, Children’s Hospital of Philadelphia, Philadelphia, PA 19104, USA; Expertise Centre for Mitochondrial Diseases (Mitohaus), University Children’s Hospital, Paracelsus Medical University (PMU) Salzburg, Salzburg 5020, Austria; Department of Pediatrics, Weifang Maternal and Children Health Hospital, Weifang 261000, China; Department of Neurology, Beijing Children’s Hospital, Capital Medical University, National Center for Children’s Health, Beijing 100005, China; Department of Neurology, Beijing Children’s Hospital, Capital Medical University, National Center for Children’s Health, Beijing 100005, China; Institute of Neurogenomics, Computational Health Centre, Helmholtz Zentrum München, Neuherberg 85764, Germany; Diagnostics and Therapeutics of Intractable Diseases, Intractable Disease Research Center, Graduate School of Medicine, Juntendo University, Tokyo 113-842, Japan; Department of Metabolism, Chiba Children’s Hospital, Chiba City 266-0007, Japan; Department of Pediatric Neurosciences, Child Neurology Unit, Fondazione IRCCS Istituto Neurologico Carlo Besta, Milan 20133, Italy; Department of Pediatric Neurosciences, Child Neurology Unit, Fondazione IRCCS Istituto Neurologico Carlo Besta, Milan 20133, Italy; Department of Paediatrics, Nutrition and Metabolic Diseases, The Children’s Memorial Health Institute, Warsaw 04-730, Poland; Department of Paediatrics, Nutrition and Metabolic Diseases, The Children’s Memorial Health Institute, Warsaw 04-730, Poland; CENTOGENE GmbH, Rostock 18055, Germany; CENTOGENE GmbH, Rostock 18055, Germany; Department of Medicine, Clinic III, Hematology, Oncology, Palliative Medicine, University of Rostock, Rostock 18051, Germany; Department of Pediatrics, Rare Diseases and Metabolic Medicine, Pomeranian Medical University, 171-252 Szczecin, Poland; CENTOGENE GmbH, Rostock 18055, Germany; Department of Clinical Genetics, Copenhagen University Hospital Rigshospitalet, Copenhagen 2100, Denmark; Department of Clinical Medicine, University of Copenhagen, Copenhagen 2200, Denmark; Klinikum am Steinenberg, Children’s Hospital Reutlingen, Reutlingen 72764, Germany; Department of Pediatrics and Internal Medicine, Radboudumc Amalia Childrens Hospital, Radboud Center for Mitochondrial Medicine, Nijmegen 6525, The Netherlands; School of Medicine, Institute of Human Genetics, Klinikum Rechts der Isar, Technical University of Munich, Munich 81675, Germany; Institute of Neurogenomics, Computational Health Centre, Helmholtz Zentrum München, Neuherberg 85764, Germany; Division of Pediatric Neurology, Developmental Medicine and Social Pediatrics, Department of Pediatrics, Dr. von Hauner Children’s Hospital, Ludwig-Maximilian University (LMU) Munich, Munich 80337, Germany; Department of Translational Genomics, Center for Genomic Medicine, King Faisal Specialist Hospital and Research Center, Riyadh 11211, Saudi Arabia; School of Medicine, Institute of Human Genetics, Klinikum Rechts der Isar, Technical University of Munich, Munich 81675, Germany; Institute of Neurogenomics, Computational Health Centre, Helmholtz Zentrum München, Neuherberg 85764, Germany; Lifera Omics, Riyadh 11452, Saudi Arabia; Department of Translational Genomics, Center for Genomic Medicine, King Faisal Specialist Hospital and Research Center, Riyadh 11211, Saudi Arabia; Department of Translational Genomics, Center for Genomic Medicine, King Faisal Specialist Hospital and Research Center, Riyadh 11211, Saudi Arabia; Mitochondrial Research Group, Translational and Clinical Research Institute, Faculty of Medical Sciences, Newcastle University, Newcastle upon Tyne NE2 4HH, UK; NHS Highly Specialised Service for Rare Mitochondrial Disorders, Newcastle upon Tyne Hospitals NHS Foundation Trust, Newcastle upon Tyne NE1 4LP, UK; Division of General Pediatrics, Department of Pediatrics and Adolescent Medicine, Medical University of Graz, Graz 8010, Austria; Pediatric Neurology Department, Carol Davila University of Medicine and Pharmacy, Alexandru Obregia Clinical Hospital, Bucharest 050474, Romania; Department of Pediatrics, University Hospital Centre, Zagreb and University of Zagreb, School of Medicine, Zagreb 10000, Croatia; Centro de Referencia Nacional (CSUR) y Europeo (MetabERN) en Enfermedades Metabólicas, Hospital Universitario 12 de Octubre, Instituto de Investigación i+12, CIBERER, Madrid 28041, Spain; Neuropediatrics, General Pediatrics, Diabetology, Endocrinology and Social Pediatrics, University of Tuebingen, University Hospital Tübingen, Tübingen 72016, Germany; Department of Neuropediatrics, University Children’s Hospital Zurich, Zurich 8008, Switzerland; Division of Pediatric Neurology, Developmental Medicine and Social Pediatrics, Department of Pediatrics, Dr. von Hauner Children’s Hospital, Ludwig-Maximilian University (LMU) Munich, Munich 80337, Germany; Reference Center for Inherited Metabolic Diseases and Reference Center for Mitochondrial Disorders (CARAMMEL), Hopital Necker-Enfants Malades, AP-HP, University Paris Cité, Paris 75015, France; Department of Neurosciences, Rehabilitation, Ophthalmology, Genetics, Maternal and Child Health (DINOGMI), University of Genoa, Genoa 16132, Italy; Paediatric Neurology and Muscle Disease Unit, IRCCS Istituto Giannina Gaslini, Genoa 16147, Italy; Institute of Biomedical Sciences, Faculty of Medicine, Vilnius University, Vilnius 03101, Lithuania; Division of Medical Genetics and Genomics, Stead Family Department of Pediatrics, University of Iowa, Iowa City, IA 52242, USA; Sanford Health, Medical Genetics, Fargo, North Dakota 58103, USA; Division of Metabolism and Nutrition, Department of Pediatrics, Faculty of Medicine, Ege University, Izmir 35100, Turkey; National Centre for Inherited Metabolic Disorders, Children’s Health Ireland, Dublin D01 XD99, Ireland; Reference Center for Inherited Metabolic Diseases, Hopital Necker Enfants Malades, Institut Imagine, INEM, AP-HP, University Paris Descartes, Paris 75015, France; Division of Metabolic Diseases and Hepatology, Bambino Gesù Children’s Hospital, IRCCS, Rome 00165, Italy; Department of General Pediatrics, Neonatology and Pediatric Cardiology, Medical Faculty, University Children’s Hospital, Heinrich-Heine-University, Düsseldorf 40225, Germany; Department of Pediatrics, Division of Human Genetics, Mitochondrial Medicine Frontier Program, Children’s Hospital of Philadelphia, Philadelphia, PA 19104, USA; Department of Pediatrics, University of Pennsylvania Perelman School of Medicine, Philadelphia, PA 19104, USA; Reference Center of Inherited Metabolic Disease, Unidade Local de Saúde de São José, Lisbon Clinical Academic Center, Lisboa 1169-045, Portugal; Eunice Kennedy Shriver National Institute of Child Health and Human Development, National Institutes of Health, Bethesda, MD 20892, USA; Department of Pediatrics, University of Pennsylvania Perelman School of Medicine, Philadelphia, PA 19104, USA; Section of Metabolic Disease, The Children’s Hospital of Philadelphia, Philadelphia, PA 19104, USA; Division of Nutrition and Metabolism, Istanbul Medical Faculty Children’s Hospital, Istanbul University, Istanbul 34390, Turkey; Division of Metabolism & Children’s Research Center, University Children’s Hospital, Zürich 8032, Switzerland; Expertise Centre for Mitochondrial Diseases (Mitohaus), University Children’s Hospital, Paracelsus Medical University (PMU) Salzburg, Salzburg 5020, Austria; Department for Pediatrics, Klinikum Karlsruhe, Karlsruhe 76133, Germany; Institute of Human Genetics, TUM School of Medicine and Health, Technical University of Munich, Munich 81675, Germany; Institute of Human Genetics, University Hospital, Ludwig-Maximilians University, Munich 80336, Germany; Medical Faculty Heidelberg, and Center for Pediatric and Adolescent Medicine, Department I, Division of Pediatric Neurology and Metabolic Medicine, Heidelberg University, University Hospital Heidelberg, Heidelberg 69120, Germany; Department of Paediatrics and Inherited Metabolic Disorders, First Faculty of Medicine, Charles University and General University Hospital in Prague, 128 08 Praha, Czech Republic; Department of Paediatrics, LKH Bregenz, Bregenz 6900, Austria; Division of Metabolism, University Children’s Hospital, Zürich 8008, Switzerland; Reference Center for Metabolic Diseases, Pediatric Department, Hospital de Santa Maria, ULSSM, Lisboa 1169-045, Portugal; Faculdade de Medicina, Universidade de Lisboa, Lisboa 1649-028, Portugal; Department of Paediatrics, Division of Genetics, Massachusetts General Hospital, Harvard Medical School, Boston, MA 02114, USA; Department of Pediatric Metabolic Diseases, Children’s Hospital, Ankara Bilkent City Hospital, Ankara 06800, Türkiye; Department of Pediatrics, Geneva University Hospital, Geneva 1205, Switzerland; Department of Neuropediatrics, Children’s Hospital Aschaffenburg-Alzenau, Aschaffenburg 63739, Germany; Department of Neurology, Friedrich-Baur-Institute, LMU University Hospital, Ludwig-Maximilians-Universität München, Munich 80336, Germany; German Center for Neurodegenerative Diseases (DZNE) Munich, Ludwig-Maximilians-Universität München, 81377 Munich, Germany; Munich Cluster for Systems Neurology (SyNergy), Munich 81377, Germany; National Centre for Inherited Metabolic Disorders, Children’s Health Ireland, Dublin D01 XD99, Ireland; Expertise Centre for Mitochondrial Diseases (Mitohaus), University Children’s Hospital, Paracelsus Medical University (PMU) Salzburg, Salzburg 5020, Austria; Department of Biology and Microbiology, Riga Stradiņš University, Riga LV-1007, Latvia; Unit of Medical Genetics and Neurogenetics, Fondazione IRCCS Istituto Neurologico Carlo Besta, Milan 20126, Italy; Biochemistry Department, Bicêtre Hospital, APHP Paris Saclay, Le Kremlin Bicêtre 94270, France; Department of Neurology, Beijing Children’s Hospital, Capital Medical University, National Center for Children’s Health, Beijing 100005, China; Section of Inborn Errors of Metabolism, Dr. von Hauner Children’s Hospital, University of Munich, Munich 80337, Germany; Division of Metabolic Diseases and Hepatology, Bambino Gesù Children’s Hospital, IRCCS, Rome 00165, Italy; Mitochondrial Research Group, Translational and Clinical Research Institute, Faculty of Medical Sciences, Newcastle University, Newcastle upon Tyne NE2 4HH, UK; NHS Highly Specialised Service for Rare Mitochondrial Disorders, Newcastle upon Tyne Hospitals NHS Foundation Trust, Newcastle upon Tyne NE1 4LP, UK; Department of Medical Genetics, Kaiser Permanente Oakland Medical Center, Oakland, CA 94611, USA; Institute of Genomic Medicine and Rare Disorders, Semmelweis University, Budapest 1085, Hungary; Department of Inherited Metabolic Disease, Evelina London Children’s Hospital, London SE1 7EH, UK; Service de Neurologie Pédiatrique, Cliniques Universitaires Saint-Luc, UCLouvain, Brussels 1200, Belgium; Medicine Department, Santa Maria University Hospital, Lisbon 1649-028, Portugal; Genetics and Personalized Medicine Clinic, Tartu University Hospital, Tartu 50406, Estonia; Department of Genetics and Personalized Medicine, Institute of Clinical Medicine, University of Tartu, Tartu 50406, Estonia; Centre of Translational and Experimental Myology, IRCCS Istituto Giannina Gaslini, Genoa 16147, Italy; Center for Child Neurology, Cleveland Clinic Children’s Hospital, Cleveland, OH 44195, USA; Department of Metabolic Medicine, Royal Children’s Hospital, Melbourne, Victoria 3052, Australia; Reference Center for Inherited Metabolic Diseases and Reference Center for Mitochondrial Disorders (CARAMMEL), Hopital Necker-Enfants Malades, AP-HP, University Paris Cité, Paris 75015, France; Division of General Pediatrics, Department of Pediatrics and Adolescent Medicine, Medical University of Graz, Graz 8010, Austria; Department of Pediatrics, University Hospital Centre, Zagreb and University of Zagreb, School of Medicine, Zagreb 10000, Croatia; Facultad de Medicina, Center for Genetics and Genomics, Clinica Alemana Universidad del Desarrollo, Santiago 7550000, Chile; Faculdade de Farmácia, iMed.ULisboa-Instituto de Investigação do Medicamento, Universidade de Lisboa, Lisbon 1649-003, Portugal; Department of Pediatrics and Internal Medicine, Radboudumc Amalia Childrens Hospital, Radboud Center for Mitochondrial Medicine, Nijmegen 6525, The Netherlands; Department of Translational Medicine, Section of Paediatrics, University of Naples ‘Federico II’, Naples 80131, Italy; Reference Center for Inherited Metabolic Diseases and Reference Center for Mitochondrial Disorders (CARAMMEL), Hopital Necker-Enfants Malades, AP-HP, University Paris Cité, Paris 75015, France; Department of Pediatric Cardiology and Intensive Care Medicine, Hannover Medical School, Hannover 30625, Germany; Division of Genetics, MaineHealth Maine Medical Center Portland, Barbara Bush Children’s Hospital, Portland, ME 04102, USA; Neuropediatrics Unit, of the Pediatrics Department, Pedro Hispano Hospital, ULSM, Matosinhos 4464, Portugal; Division of Metabolic Diseases and Hepatology, Bambino Gesù Children’s Hospital, IRCCS, Rome 00165, Italy; Expertise Centre for Mitochondrial Diseases (Mitohaus), University Children’s Hospital, Paracelsus Medical University (PMU) Salzburg, Salzburg 5020, Austria; Department of Neurosciences, Rehabilitation, Ophthalmology, Genetics, Maternal and Child Health (DINOGMI), University of Genoa, Genoa 16132, Italy; Paediatric Neurology and Muscle Disease Unit, IRCCS Istituto Giannina Gaslini, Genoa 16147, Italy; Department of Inborn Errors of Metabolism and Paediatrics, The Institute of Mother and Child, Warsaw 01-211, Poland; Metabolic Clinic and Pediatric Department B, Ruth Rappaport Children’s Hospital, Rambam Health Care Campus, Haifa 3109601, Israel; Rappaport Faculty of Medicine, Technion-Israel Institute of Technology, Haifa 3109601, Israel; Mitochondrial Research Group, Translational and Clinical Research Institute, Faculty of Medical Sciences, Newcastle University, Newcastle upon Tyne NE2 4HH, UK; NHS Highly Specialised Service for Rare Mitochondrial Disorders, Newcastle upon Tyne Hospitals NHS Foundation Trust, Newcastle upon Tyne NE1 4LP, UK; Department for Inborn Metabolic Diseases, University Children’s Hospital, University Medical Center Hamburg-Eppendorf, Hamburg 20246, Germany; Division of Metabolism and Nutrition, Department of Pediatrics, Faculty of Medicine, Ege University, Izmir 35100, Turkey; Department of Metabolic Diseases, Wilhelmina Children’s Hospital University Medical Center Utrecht, Utrecht 3584 EA, The Netherlands; Department for Inborn Metabolic Diseases, University Children’s Hospital, University Medical Center Hamburg-Eppendorf, Hamburg 20246, Germany; Department of Metabolic Medicine, Royal Children’s Hospital, Melbourne, Victoria 3052, Australia; Division of Pediatric Metabolism, Department of Pediatrics, Faculty of Medicine, Hacettepe University, Ankara 06230, Turkey; Institute of Human Genetics, University Hospital, Magdeburg 39120, Germany; Bókay Street Department, Pediatric Centre, Semmelweis University, Budapest 1083, Hungary; Institute of Human Genetics, University Hospital Salzburg, Paracelsus Medical University, Salzburg 5020, Austria; Expertise Centre for Mitochondrial Diseases (Mitohaus), University Children’s Hospital, Paracelsus Medical University (PMU) Salzburg, Salzburg 5020, Austria; School of Medicine, Institute of Human Genetics, Klinikum Rechts der Isar, Technical University of Munich, Munich 81675, Germany; Oxford Medical Genetics Laboratories, Oxford University Hospitals NHS Foundation Trust, The Churchill Hospital, Oxford OX3 7LE, UK; School of Medicine, Institute of Human Genetics, Klinikum Rechts der Isar, Technical University of Munich, Munich 81675, Germany; Institute of Neurogenomics, Computational Health Centre, Helmholtz Zentrum München, Neuherberg 85764, Germany; German Center for Child and Adolescent Health (DZKJ), Partner Site Munich, Munich 80337, Germany; Expertise Centre for Mitochondrial Diseases (Mitohaus), University Children’s Hospital, Paracelsus Medical University (PMU) Salzburg, Salzburg 5020, Austria; Institute of Human Genetics, University Hospital Salzburg, Paracelsus Medical University, Salzburg 5020, Austria; Expertise Centre for Mitochondrial Diseases (Mitohaus), University Children’s Hospital, Paracelsus Medical University (PMU) Salzburg, Salzburg 5020, Austria

**Keywords:** inborn errors of metabolism, inborn metabolic disease, mitochondrial disease, genotype-phenotype correlation, ketogenic diet, treatment

## Abstract

This retrospective study on X-linked *PDHA1-*related pyruvate dehydrogenase complex (PDHc) deficiency combined a systematic literature review with a multicentre survey exploring genotypes, phenotypes and survival.

Data from 891 individuals (45% unpublished) were included. Of note, 53% of cases were females. Median age at last assessment was 6 years (range 0–80 years, *n* = 622).

We detected 331 different (118 unpublished) *PDHA1* variants, of which 75% (305/405) had occurred *de novo*. Variants in this study were uploaded to ClinVar (SCV006297015—SCV006297345). The 10 most frequent variants accounted for 36% of the diagnoses. Sixty-nine per cent of the variants were private; missense (50%) and frameshift (20%) variants were most common. Frameshift/nonsense (FS/N) variants in males (44/401, 11%) were confined to regions escaping nonsense-mediated decay (NMD) and were significantly less frequent than in females (151/461, 33%). Neonatal or infantile (405/529, 77%) presentations were most frequent, with pre/perinatal abnormalities reported in 47% (159/342). FS/N variants in the NMD-predicted region 3.9 [95% confidence interval (CI) 1.54–11.04] times increased the odds of fetal findings. Females presented significantly earlier [2 months, interquartile range (IQR) 7.0, *n* = 224] than males (8 months, IQR 16.6, *n* = 233), with increased risk of neonatal presentation [odds ratio (OR) 3.01 (95% CI 1.279–7.616)] when harbouring FS/N variants in the NMD-predicted region. The overall (*n* = 242) mean survival time was 10.9 (95% CI 9.9–11.9) years. On average, females survived 4.5 (95% CI 2.62–6.40) years longer than males despite presenting more severe phenotypes. Poor survival was associated with male sex [hazard ratio (HR) 3.3 (95% CI 1.95–5.62)], neonatal presentation [HR 5.5 (95% CI 2.17–14.09)], FS/N variants in the NMD-predicted region [HR 4.0 (95% CI 1.78, 9.16)] and splice variants [HR 2.3 (95% CI 1.15, 4.59)]. More severe clinical phenotypes were predicted by neonatal or infantile presentations and by female sex. Developmental delay (DD), intellectual disability (ID), muscle hypotonia, abnormal movements, seizures, feeding difficulties and microcephaly were the most frequent phenotypes, all occurring in more than half. Corpus callosum or basal ganglia alterations and cerebral atrophy were common. Four per cent (36/891) were reported to have mild phenotypes with no DD nor ID (25/36 males).

This is the largest dataset on a nuclear-encoded defect of mitochondrial energy metabolism. The genotypic and phenotypic details further defines the disease landscape and can be used for variant interpretation. The correlations between genotypes, sex, phenotypes and survival, adds substantial improvement to counselling.

## Teaser

Merkevicius *et al.* analysed data from 891 individuals with *PDHA1*-related pyruvate dehydrogenase complex deficiency, a disorder of mitochondrial energy metabolism. The results reveal how age at onset, sex, and genetic variant type relate to clinical features and survival, offering insights that may improve diagnosis and management.

## Introduction

The pyruvate dehydrogenase complex (PDHc) is a multienzyme complex of pyruvate dehydrogenase (E1-), dihydrolipoamide acetyltransferase (E2-), dihydrolipoamide dehydrogenase (E3-subunit) and the E3-binding protein.^1-3^ PDHc catalyses the oxidative decarboxylation of pyruvate to acetyl-coenzyme A (acetyl-CoA) using cofactors thiamine pyrophosphate (TPP), lipoamide, coenzyme A, flavin adenine dinucleotide (FAD) and nicotinamide adenine dinucleotide (NAD). Acetyl-CoA synthesis links glycolysis to the citric acid cycle and downstream oxidative phosphorylation, resulting in energy production. This irreversible process is fundamental for mitochondrial energy metabolism.

During embryonic neurogenesis, the human nervous system shifts from aerobic glycolysis to oxidative phosphorylation.^4,5^ Under normoglycaemic conditions, the brain relies almost entirely on PDHc, utilizing glucose or ketone bodies during fasting.^6^ Similarly, the peripheral nervous system relies on glucose and lactate for energy, making it PDHc dependent.^7^ In contrast, the heart—a highly energy-dependent organ—utilizes fatty acids (even under normoglycaemic conditions) and glucose, rendering it less PDHc dependent.^8,9^ These organ systems illustrate the two extremes of a PDHc dependency spectrum.

In most individuals with PDHc deficiency (MIM #312170), the E1α subunit (encoded by *PDHA1,* MIM300502) is affected. The exact prevalence is unknown. *PDHA1* is located on Xp22.1, with both hemizygous males and symptomatic heterozygous females reported.^10^

Individuals with PDHc deficiency regularly present with (congenital) lactic acidosis and neurodevelopmental disorder.^2,11^ Pathomechanism-based supportive treatments have been reported to positively influence the clinical course.^12^ These are high-fat, low-carbohydrate ketogenic diets (KD) that switch metabolism towards ketone body production, providing an alternative energy source circumventing PDHc,^13^ as well as supplementation with thiamine (vitamin B1), the precursor of TPP, a cofactor of the E1 subunit and a chaperone for the PDHc.^1-3^ Furthermore, small molecule therapies [e.g. dichloroacetate (DCA), phenylbutyrate] targeting PDHc activity regulating enzymes^14-16^ have been reported in single cases or small case series. These treatment possibilities mandate timely diagnosis and exclusion of other aetiologies in which a KD is contraindicated, in particular pyruvate carboxylase deficiency (MIM #266150).^17^

This study provides the most comprehensive characterization of *PDHA1*-related PDHc deficiency, describing genotypes and phenotypes in 891 cases.

## Materials and methods

### Study design and data acquisition

**Figure 1:**
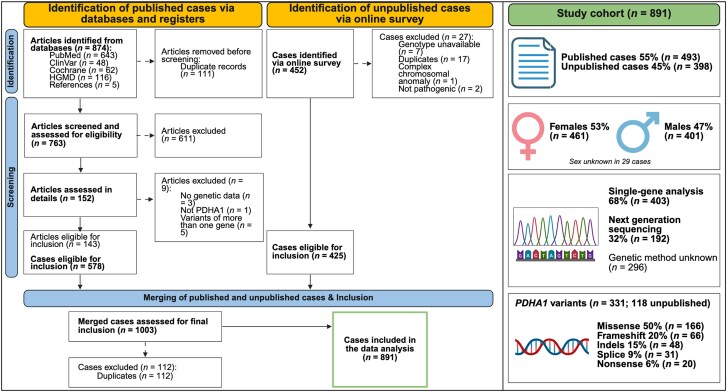
**Flow diagram detailing the inclusion process**. Figure depicts the inclusion of *PDHA1*-related Pyruvate dehydrogenase complex (PDHc) deficiency cases. Both published and unpublished cases that met the inclusion criteria were combined for further analysis. Duplicate control was conducted based on reporting authors, genotype, phenotype and information provided by collaborators, both during the literature search and upon case merging, and any discrepancies were resolved through discussion. Indels = small in-frame insertions or deletions; HGMD = The Human Gene Mutation Database; VUS = variant of unknown significance. Created in BioRender. Merkevicius, K. (2025) https://BioRender.com/3eu8f5b.

This study combines a systematic literature review and a retrospective cohort study, using a two-step approach (Fig. 1). Following Preferred Reporting Items for Systematic reviews and Meta-Analyses (PRISMA) 2020 guidelines,^18^ affected individuals with *PDHA1* variants were identified by two authors independently (search date 12 July 2024) via PubMed, ClinVar, The Human Gene Mutation Database (HGMD) and Cochrane Library electronic databases (keywords ‘PDHD’, ‘PDHc’, ‘Pyruvate dehydrogenase e1’, ‘*PDHA1*’, ‘Pyruvate dehydrogenase deficiency’; inclusion criteria: full text available, English language; exclusion criteria: presence of a second genetic defect including large deletions affecting *PDHA1* and adjacent genes, conference abstracts). This review was not registered, and no protocol was prepared or published prior to this article. A quality assessment of included studies was performed using the Joanna Briggs Institute critical appraisal checklist (Supplementary Tables 1 and 2).^19,20^

Two authors independently extracted data using a data extraction form (Supplementary Table 3), any discrepancies were resolved by discussion. Next, the same data extraction form was used to collect anonymized data from unpublished cases via international collaborators. Data is reported following the STrengthening the Reporting of OBservational studies in Epidemiology (STROBE) statement.^21^ Data from the literature and collaborators were merged and analysed collectively (Fig. 1). Clinical phenotypes were standardized using human phenotype ontology (HPO) terms^22^ (Supplementary Table 3). Clinical, biochemical and neuroimaging data were collected in binary or nominal formats. Cases reported without developmental delay (DD) before 1 year or without intellectual disability (ID) before 6 years were considered non-informative and not assumed free of these conditions.^23-25^ Neuroimaging data were obtained from reports, with no direct image analysis. This study was not intended to evaluate intervention efficacy. Collected data encompassed: whether KD or thiamine supplementation were used and whether treating physicians considered treatment successful. Treatment success criteria were not defined. Treatment regimens, compliance and age at initiation were not collected.

Genetic investigations and enzyme activity measurements of unpublished cases were performed at specialized centres. Enzyme activity was recorded as percentages of normal.

This study was conducted in accordance with the Declaration of Helsinki,^26^ and approved by the Institutional Review Board of the Paracelsus Medical University, Salzburg, Austria (PMU-EK-2024-0043).

### Repeated inclusion control

To avoid duplicate inclusion, extensive cross-checking considering sex, genotype, phenotype (e.g. age at presentation/death, residual PDHc enzyme activity), publishing authors, referring collaborators/centres and cross-referencing with published cases was performed. Supplementary Table 4 lists cases from the literature identified as duplicates, which were pooled accordingly. For published cases with additional data reported by collaborators, data was pooled and cases were marked as published (data not shown).

### Variant interpretation

We classified all *PDHA1* (NM_000284.3) variants according to the American College of Medical Genetics and Genomics and the Association for Molecular Pathology (ACMG/AMP) recommendations (Supplementary Table 5),^27,28^ resolving discrepancies through discussion with metabolic clinicians, geneticists and biochemists. Only cases with (likely) pathogenic variants were included (Supplementary Table 6). Regions between p.Met1 to p.Ala34 and p.Leu319 to p.Ser390 were considered as regions predicted to escape nonsense-mediated decay (NMD-escape) and the remaining as NMD-predicted regions according to Database of Chromosomal Imbalance and Phenotype in Humans Using Ensembl Resources (DECIPHER).^29^ Variant discovery was estimated as the proportion of known disease-causing missense variants (from literature or ClinVar) over all possible likely pathogenic missense variants predicted by AlphaMissense.^30^

### Statistical analysis

Due to uneven reporting, denominators represent cases with available data, excluding missing values. Analysis included cases with available data, imputations were not used. In subgroup analysis, groups with < 10 cases were excluded, unless specified otherwise. Statistical analysis and visualizations were conducted in RStudio (version 2023.09.0 + 463). Differences with *P*-values < 0.05 were considered statistically significant. Distribution was assessed using the Shapiro–Wilk test. Continuous variables are presented as mean [standard deviation (SD)] or median [interquartile range (IQR)] for normal or non-normal distribution, respectively. Where applicable, 95% confidence interval (CI) is provided. Parametric (*t*-test with Levene’s test for variance equality; ANOVA with Bonferroni corrections) and non-parametric (Mann–Whitney U-test; Kruskal–Wallis H with Dunn’s *post hoc*) tests were used for continuous variables. Categorical data are reported as counts and percentages, analysed with chi-square with Yates’s correction for continuity or Fisher’s exact test. Linear and logistic regression with forward/backward stepwise selection was used for association analysis. Variables with a variance inflation factor > 2.5 were not included in regression models to avoid multicollinearity. The Poisson test assessed the significance of Observed-to-Expected (O/E) ratio. The binomial test was used to assess proportion distribution in binary variables. Phenotype distribution homogeneity was defined as 1-Shannon entropy (H).

Cohort’s variant frequency was compared with that of the general population using the gnomAD database.^31^ The filtering of gnomAD variants was based on the following criteria: (i) allele frequency < 0.1%; (ii) ClinVar germline classification not labelled as benign, likely benign or benign/likely benign; (iii) variants located within the canonical transcript (ENST00000422285.7, equal to GenBank NM_000284.4); and (iv) exclusion of variants annotated as untranslated region (UTR) variants. Additional filters: (i) allele count (AC = 1) for private variants; or (ii) exclusion of intronic, splice and synonymous variants for coding variants.

Survival was assessed with the Kaplan–Meier KM estimator and restricted mean survival time (RMST).^32^ Cox proportional hazards (Cox PH) models with Holm–Bonferroni corrections were used for survival covariate analysis. Survival analysis inclusion criteria: cases with known sex, age at presentation and last report, defined status at last report (alive or deceased). Cases reported as alive were considered censored. Cases with undefined status (alive or deceased) and cases with termination of pregnancy were excluded from survival analysis.^33^ Following the 10% rule, survival analysis included 90% of the eligible cases at risk.^34^

## Results

### Study cohort and general characteristics

The final cohort includes 891 cases (461/862, 53% females, sex unknown in 29) of which: 493 previously published, 398 unpublished (Fig. 1 and Supplementary material, References 1–60, 62–144). The individuals resided in 34 countries in Europe, America, Asia and Australia (Supplementary Fig. 1). In 25 families, more than one affected individual was reported, including siblings (*n* = 19) or mother-and-child-pairs (*n* = 6).

### Genotypes

#### Genetic testing strategies

Among 595 individuals (sex unknown in seven), 68% (*n* = 211 females, *n* = 190 males) were diagnosed via single-gene analysis, 10% via gene panels (*n* = 34 females, *n* = 28 males) and 22% (*n* = 68 females, *n* = 57 males) via exome (ES) or genome sequencing (GS). Published cases had more often been diagnosed with single-gene analysis (226/247, 92%), reflecting the era before next-generation sequencing (NGS), while unpublished cases were more often diagnosed via panel or ES/GS (171/348, 49%). Females (110/348, 32%) and males (85/275, 31%) were equally diagnosed via panel, ES or GS.

#### Overview of *PDHA1* variants in the cohort

**Figure 2:**
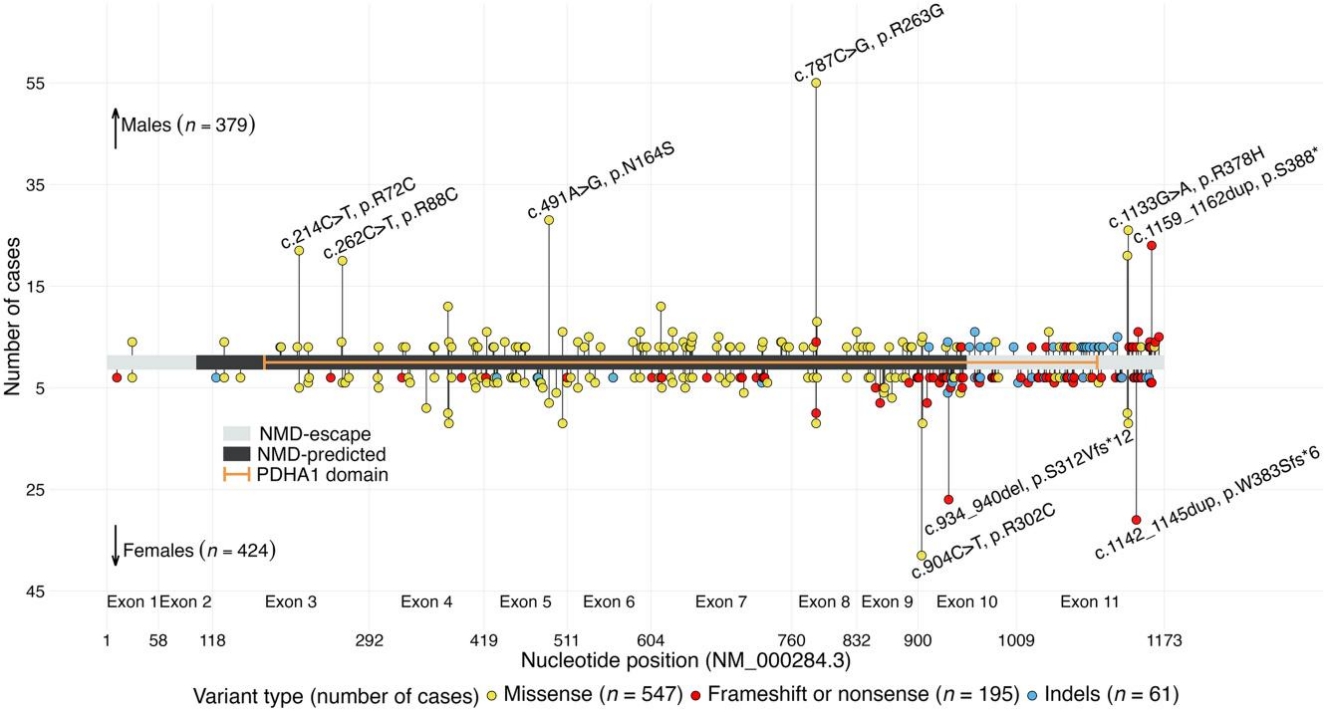
**Distribution of coding variants among males and females (splice variants excluded)**. Figure illustrates the distribution of coding variants (with exception of splice variants) within the cohort in males (*top*) and females (*bottom*) highlighting their nucleotide positions (*x*-axis) and the corresponding number of cases (*y*-axis). Cases *n* = 803, variants *n* = 300. Variants are classified by type: missense (yellow), frameshift or nonsense (red) and indels (blue). Nonsense-mediated decay (NMD)-predicted regions (black) are marked from c.102 to c.955, other regions are considered as NMD-escape regions (grey). The *PDHA1* domain refers to conserved functional domain (TIGR03182). The most frequent recurrent variants are annotated. Indels = small in-frame insertions or deletions.

A total of 331 different *PDHA1* variants were identified (Fig. 2 and Supplementary Table 6) including 118 (36%) unpublished. Of these variants, 50% (166/331) were missense, 20% (66/331) frameshift, 15% (48/331) small in-frame insertions or deletions (indels), 9% (31/331) were splice and 6% (20/331) nonsense variants (Supplementary Fig. 2 and Supplementary Table 6). This exceeds the neutral expectation based on the computed median of 17.9 protein loss-of-function (pLoF) variants per gene in gnomAD.^35^ The present study includes 13% of all possible AlphaMissense computed (likely) pathogenic missense variants in the *PDHA1* gene (Supplementary Table 7). *PDHA1* variants in this study were uploaded to ClinVar (SCV006297015–SCV006297345).

Mutational hotspots, illustrated by variant frequency,^36^ were in exons 5 (0.32 variants/base pair), 7 (0.30), 9 (0.40), 10 (0.44) and 11 (0.50), respectively, no variants were found in exon 2 (Fig. 2 and Supplementary Table 8). Similarly, according to AlphaMissense, likely pathogenic missense variants are predicted to be found mostly in exons 3–10 (Supplementary Fig. 3). Exons 5–7, 9–11 showed significant enrichment of coding variants compared with gnomAD^31^ (O/E ratio > 1.7, *P* < 0.05) (Supplementary Fig. 4 and Supplementary Table 9). FS/N variants were enriched in the NMD-predicted (O/E ratio 2.1, *P* < 0.001, *n* = 38) and NMD-escape (O/E ratio 2.7, *P* = 0.024, *n* = 48) regions.

The 10 most frequent variants [p.Arg263Gly (*n* = 68), p.Arg302Cys (*n* = 40), p.Arg378His (*n* = 35), p.Asn164Ser (*n* = 33), p.Arg378Cys (*n* = 30), p.Trp383SerfsTer6 (*n* = 30), p.Ser312ValfsTer12 (*n* = 25), p.Arg72Cys (*n* = 23), p.Ser388Ter (*n* = 22) and p.Arg88Cys (*n* = 17); Supplementary Table 6] accounted for 36% (323/891) of diagnoses. Overall, 31% (104/331) of all variants recurred in unrelated individuals (Supplementary Table 6), hence, 227/331 (69%) variants were private. Specifically, 58% (97/166) of missense variants, 77% (51/66) of frameshift variants, 83% (40/48) of indels, 71% (22/31) of splice variants and 60% (12/20) of nonsense variants were observed only once.

Inheritance data was available for 405 individuals (200 females, 204 males, sex unknown in one). In 100 cases, variants were inherited maternally, in no case paternally. In six families (six mothers and probands, four additional affected siblings), mothers were reported as symptomatic, but maternal phenotypes were not available for all cases. In most individuals (305/405, 75%) variants had arisen *de novo,* with 156 different variants [22% (33/156) recurring *de novo* variants].

#### Sex-specific genetic findings

Inherited variants were more common in males (74/204, 36%) than in females (26/200, 13%), while *de novo* occurrence was reported more often in females (174/200, 87%) than in males (130/204, 63.7%; *P* < 0.001).

Among variants observed in ≥10 individuals, 10/16 variants showed gender-specific enrichment: 7 variants [6 missense, 1 nonsense (NMD-escape region)] enriched in males and 4 variants (2 missense, 2 frameshift) enriched in females (Supplementary Tables 6 and 9).

**Figure 3:**
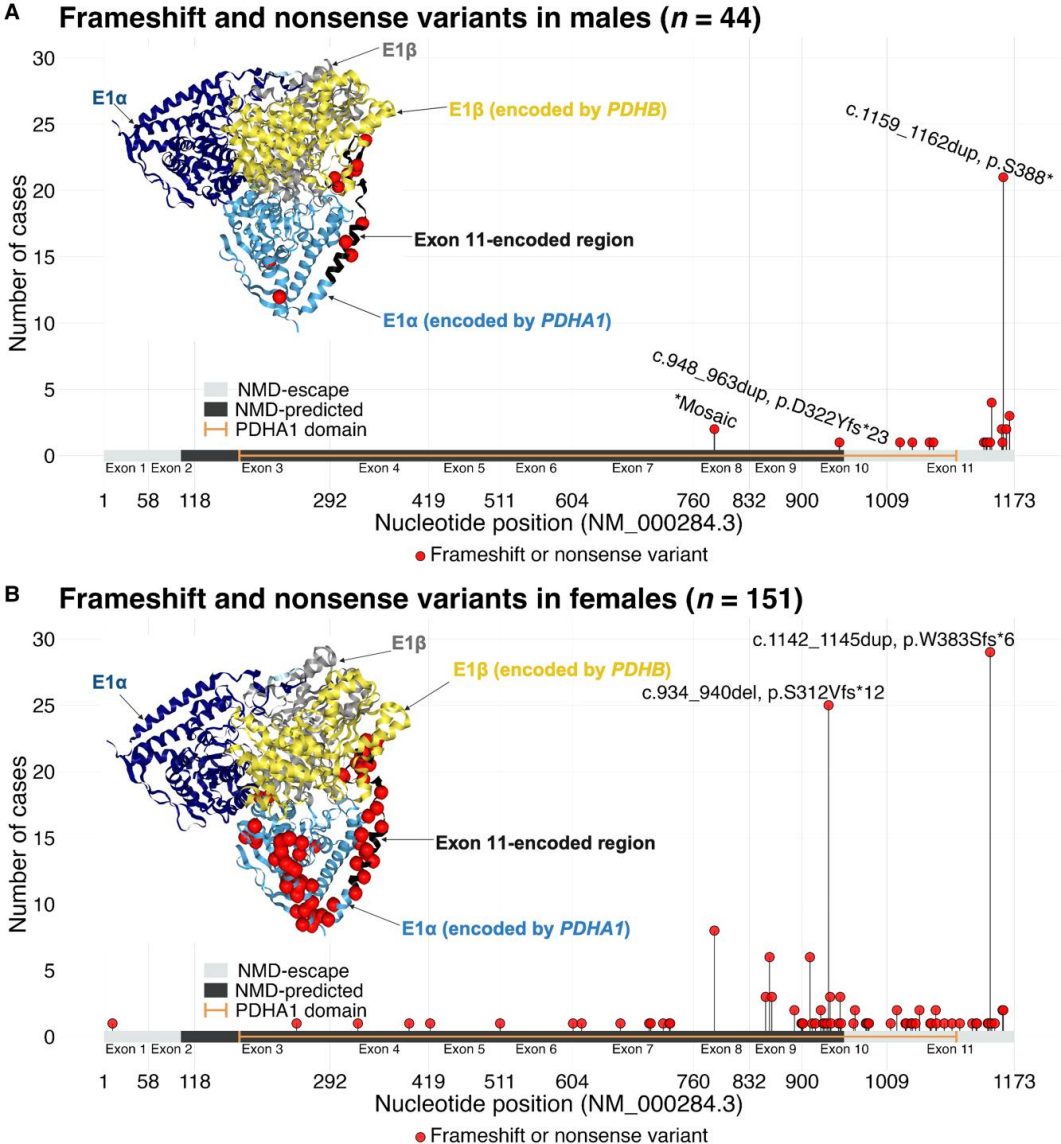
**Distribution of frameshift and nonsense variants in males and females**. Figure shows the distribution of frameshift or nonsense variants in males (**A**) and females (**B**), highlighting their nucleotide positions (*x*-axis) and the corresponding number of cases (*y*-axis). Nonsense-mediated decay (NMD)-predicted regions are marked from c.102 to c.955, other regions are considered as NMD-escape regions. (**A**) Frameshift variant c.948_963dup, p.Asp322Tyrfs*23 escapes NMD as the stop codon emerges outside the NMD-predicted region. The same variants are shown in a representative 3D Pyruvate dehydrogenase complex (PDHc) E1 protein structure, respectively. *Mosaicism confirmed in one case. Created in BioRender. Merkevicius, K. (2025) https://BioRender.com/hu515p4.

Missense variants were more prevalent in males (306/401, 76%) than females (241/461, 52%; *P* < 0.001) and FS/N variants were less frequent in males (44/401, 11%) than in females [151/461, 33%, OR 3.9 (95% CI 2.7–5.8), *P* < 0.001; Supplementary Table 6 and Fig. 3]. FS/N variants in half of exon 10 and entire exon 11 are predicted to escape NMD. These exons collectively harboured the majority of small indels (40/48, 83%) and FS/N (65/86, 76%) variants (Supplementary Table 6 and Fig. 3). More than half of females harboured FS/N variants in NMD-predicted regions (85/151, 56% females; 37/70 variants; Fig. 3). In males, FS/N variants (*n* = 19) were restricted to the NMD-escaping exon 11 (Fig. 3) except for two cases harbouring FS/N variants in the NMD-predicted region [c.787C > T, p.Arg263* (*n* = 2)], with mosaicism confirmed in one. Compared with the neutral expectation of pLoF variants per gene (see variant section),^35^ FS/N variants in the NMD-predicted region were significantly depleted in males (O/E ratio 0.06, *P* < 0.001, *n* = 1) but enriched in females (O/E ratio 2.1, *P* < 0.001, *n* = 35).

Sixty-four unique missense variants were identified exclusively in females (64/166, 38.6% among all missense variants), including 35 *de novo* and three recurring *de novo* variants: p.Arg119Trp, p.Ala236Glu, p.His367Leu. Similarly, 47 missense variants (excluding inherited) were found only in males (47/166, 28.3%), with 22 *de novo* and two recurring *de novo* variants: p.Gly195Ala, p.Tyr227His (Fig. 3 and Supplementary Table 6).

### Delineation of *PDHA1*-related PDHc deficiency clinical phenotypes

#### Age at presentation and at last assessment

The median age at presentation was 4 months (IQR 13 months; *n* = 466, sex unknown in 9). Females presented earlier (2 months, IQR 7.0, *n* = 224) than males (8 months, IQR 16.6, *n* = 233; *P* < 0.001; Supplementary Fig. 5A). In an additional 63 cases, only time frame of presentation was reported. Altogether, subjects presented in the neonatal period (0–28 days; 208/529, 39%), infancy (29 days to 12 months; 197/529, 37%), childhood (1–13 years; 115/529, 22%), adolescence (13–18 years; 6/529, 1%) or adulthood (>18 years; 3/529, 1%). The median age at last assessment was 6 years (IQR 10.5 years, *n* = 622, age range 0–80 years), 14% were older than 18 years (Supplementary Fig. 6A).

#### Survival

The majority of reported deaths came from published cases, while prolonged survival was seen in unpublished cases (Supplementary Table 10). The survival analysis combined published and unpublished cases and was limited to 18 years of age at last report (Supplementary Table 11 and Supplementary Fig. 6).

**Figure 4:**
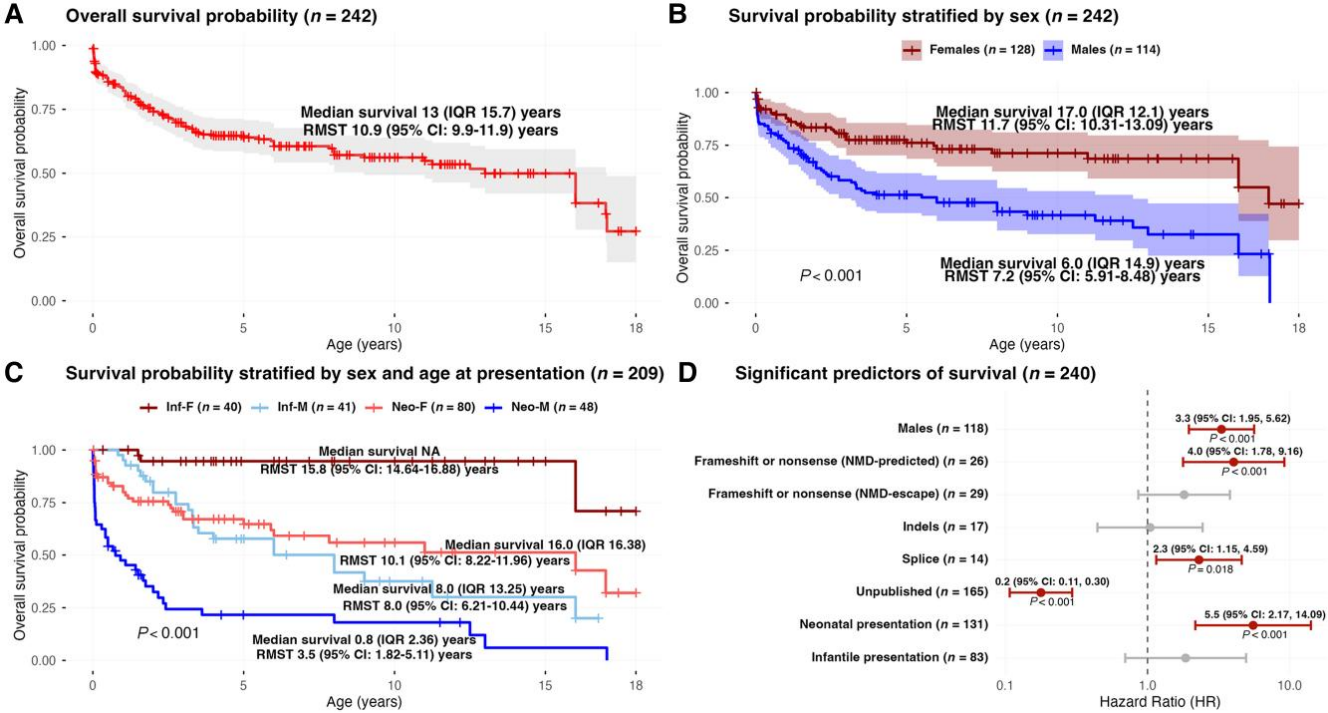
**Survival analysis and predictors of lethal outcomes in patients**. Figure illustrates the overall survival probability using the Kaplan–Meier survival estimate and restricted mean survival time (RMST) (**A**), survival stratified by gender (**B**), gender and age at first presentation (**C**), and predictors of lethal outcomes using Cox Proportional Hazards (PH) regression for the cohort over 18 years (**D**). In survival analysis, birth was used as baseline for all cases, and age at death was considered as time-to-event. The number in brackets represents the number of cases in the given subgroup. Shaded areas indicate the 95% confidence interval (CI); where applicable, median survival in years with interquartile range (IQR) and average survival time (RMST) with 95% CI are given. **A** includes cases with known survival status (alive or deceased), age at last report ≤18 years, sex, age at presentation; median age at censoring 6.2 (IQR 6.94) years (*n* = 142). **B** includes cases with known survival status (alive or deceased), age at last report ≤18 years, sex, age at presentation; median age at censoring: males 7.2 (IQR 5.90) years (*n* = 48), females 5.1 (IQR 7.52) years (*n* = 94). **C** includes cases with known survival status (alive or deceased), age at last report ≤18 years, sex, age at presentation (childhood excluded, *n* < 10 per group); median age at censoring (years): Inf-F 6.4 (IQR 8.42; *n* = 37), Inf-M 4.9 (IQR 5.75; *n* = 18), Neo-F 4.1 (IQR 6.90; *n* = 50), Neo-M 1.6 (IQR 3.57; *n* = 9). Only cases with neonatal or infantile first presentations are shown. **D** includes cases with available sex, outcome (alive or deceased), age at presentation, age at last report ≤18 years. Multivariable stepwise Cox PH model: concordance 0.84 (SE 0.018), logrank test *P* < 0.001. References: missense (*n* = 158) for variant, childhood (*n* = 29) for presentation. Non-significant predictors are shown in grey, while significant predictors are highlighted in red, with odds ratios displayed on a log scale; subanalysis additionally included prenatal and perinatal findings [prenatal movement abnormality (HP:0001557), intrauterine growth retardation (HP:0001511), polyhydramnios (HP:0001561), oligohydramnios (HP:0001562), abnormal fetal MRI and (or) ultrasound findings, birth length, weight or head circumference measurements below the 3rd percentile] not considered in **A–C**; cases with first presentation in adolescence or adulthood were not included. Only significant *P*-values are shown. NA = not available (in cases where median survival or the upper bound of the 95% CI were undefined, as fewer than 50% of cases resulted in death within 30 years follow-up in this subanalysis). Inf-F = females and infantile presentation (29 days–1 year); Inf-M = males and infantile presentation (29 days–1 year); Neo-F = females and neonatal presentation (0–28 days); Neo-M = males and neonatal presentation (0–28 days).

The overall (*n* = 242) mean survival (RMST) was 10.9 years (95% CI 9.9–11.9) and the median survival was 13 years (IQR 15.7). On average, females survived 4.5 years longer than males (95% CI 2.62–6.40; *P* < 0.001). Males also exhibited an earlier survival decline, with median survival of 6 years (IQR 14.9, *n* = 114), compared with 17 years for females (IQR 12.1, *n* = 128, *P* < 0.001; Fig. 4A and B). Males with neonatal presentation had the worst survival, while females with infantile or later presentation—the best (Fig. 4C and D and Supplementary Tables 12 and 13). In a small subset regression analysis, prenatal findings, resuscitation at birth and (or) low Apgar scores 3.1–4.4 times increased lethal outcome risk (Supplementary Table 14).

**Figure 5:**
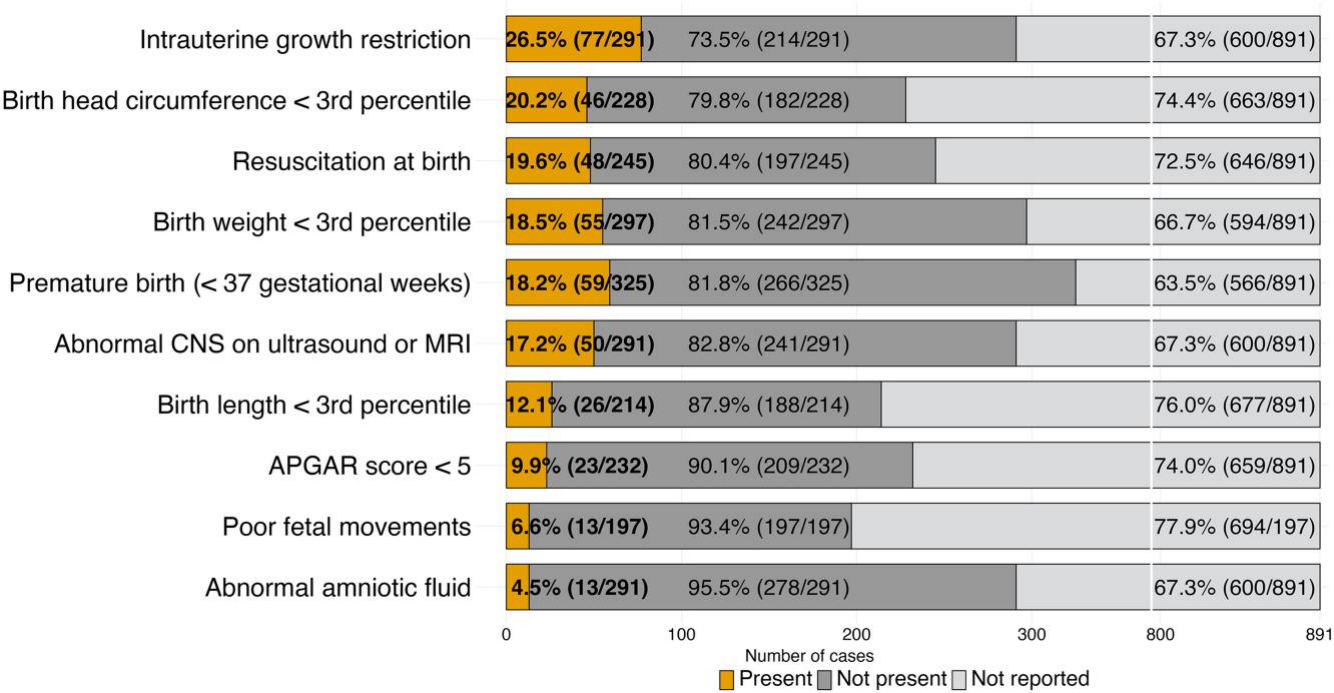
**Fetal and perinatal findings**. Figure presents a summary of fetal and perinatal findings. Apgar score <5 refers to any Apgar score below 5 evaluated at 1, 5 or 10 min after birth; abnormal amniotic fluid refers to either polyhydramnios or oligohydramnios. Findings are listed in decreasing frequency from the top.

**Figure 6:**
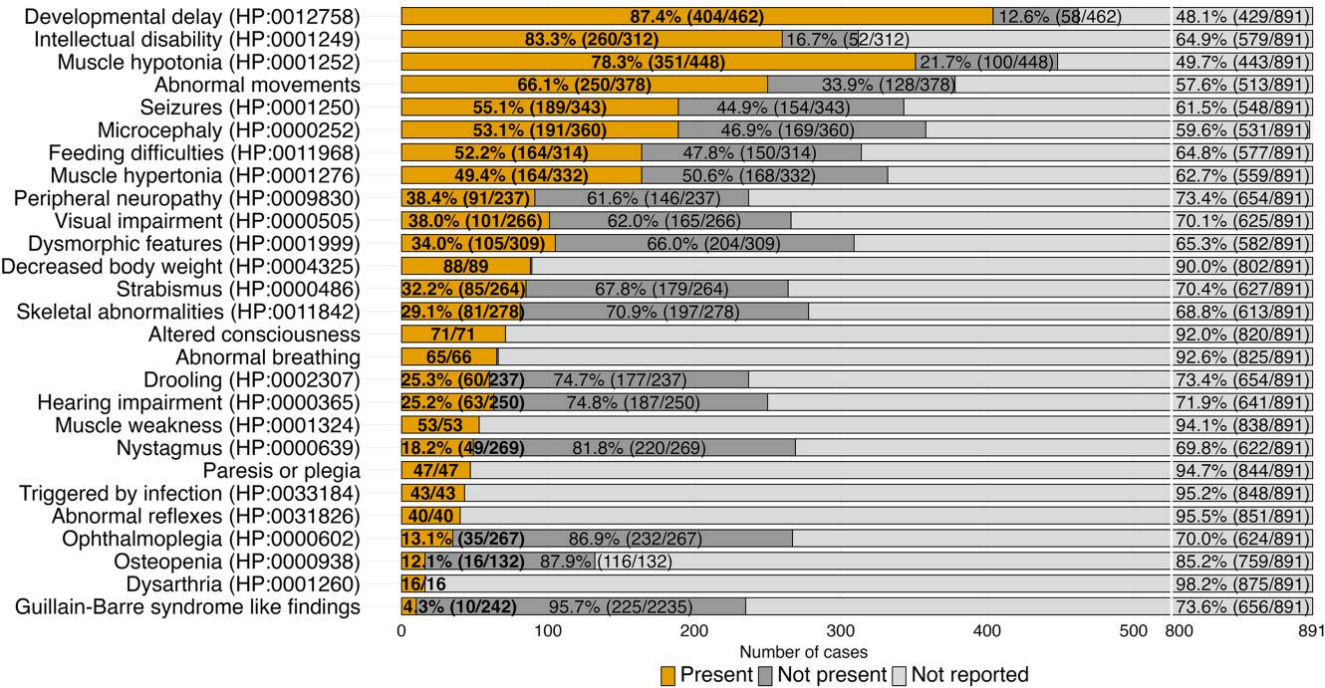
**The most common clinical phenotypes**. Figure shows the most common clinical findings reported in at least 10 cases; where applicable, HPO (human phenotype ontology) codes are provided next to each clinical phenotype. When clinical phenotype is reported only as ‘present’ and percentage of a count yields irrational values (e.g. 100%), data are reported as counts without percentages. Several phenotypes were grouped by similarity: abnormal movements (HP:0004305, HP:0100022, HP:0001288, HP:0100660, HP:0001251, HP:0001332), altered consciousness (HP:0002329, HP:0001254, HP:0001298), abnormal breathing (HP:0002104, HP:0002793, HP:0002878), paresis or plegia (HP:0004374, HP:0030182). Phenotype ‘Guillian–Barre syndrome like findings’ was described as Guillian–Barre syndrome or Guillian–Barre syndrome like. Findings are listed in decreasing frequency from the top.

**Figure 7:**
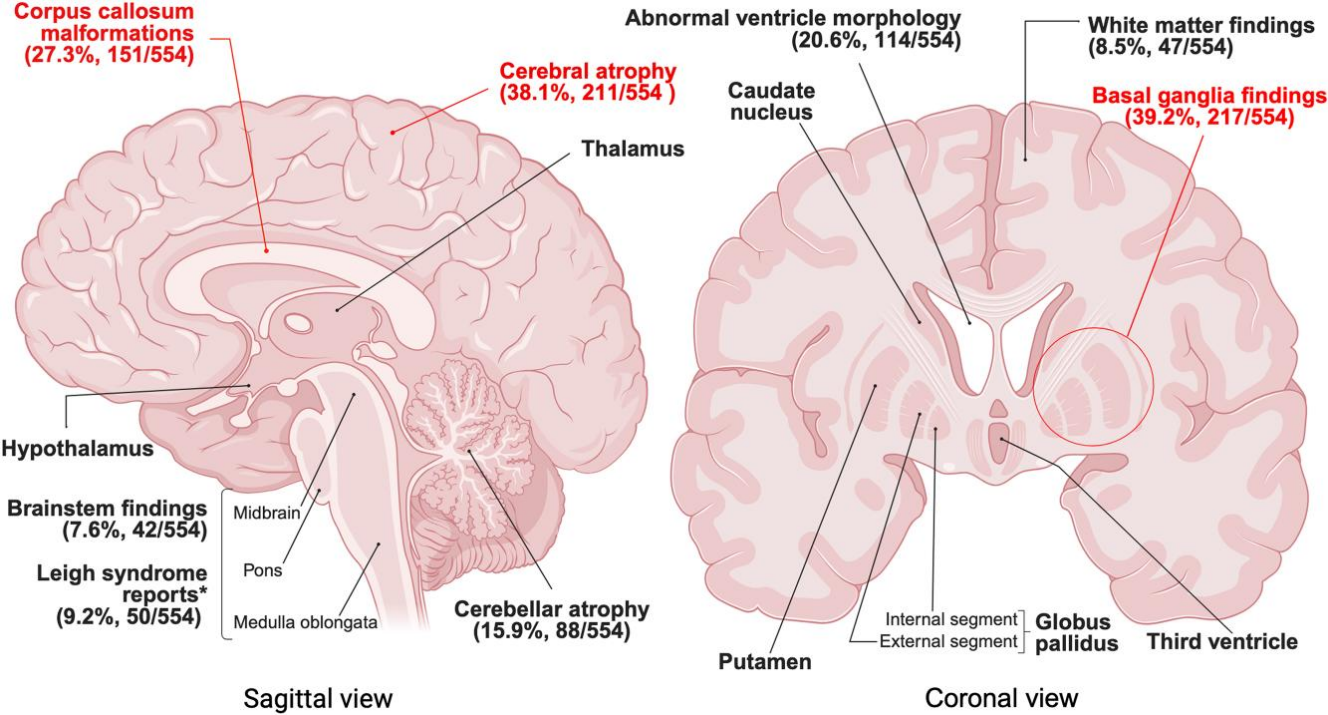
**The most common structural brain abnormalities**. Figure shows the majority of brain structural findings within our cohort in sagittal (*left*) and coronal (*right*) views of the brain. Affected anatomical structures are marked in bold. The frequency and number of cases for the most commonly reported findings are provided (top three are marked in red), compared with all cases that had information on brain structural findings. *Cases where CNS structural changes are reported as Leigh syndrome with limited details or without providing specific details about the abnormalities are also included. Created in BioRender. Merkevicius, K. (2025) https://BioRender.com/lgjswhw.

**Figure 8:**
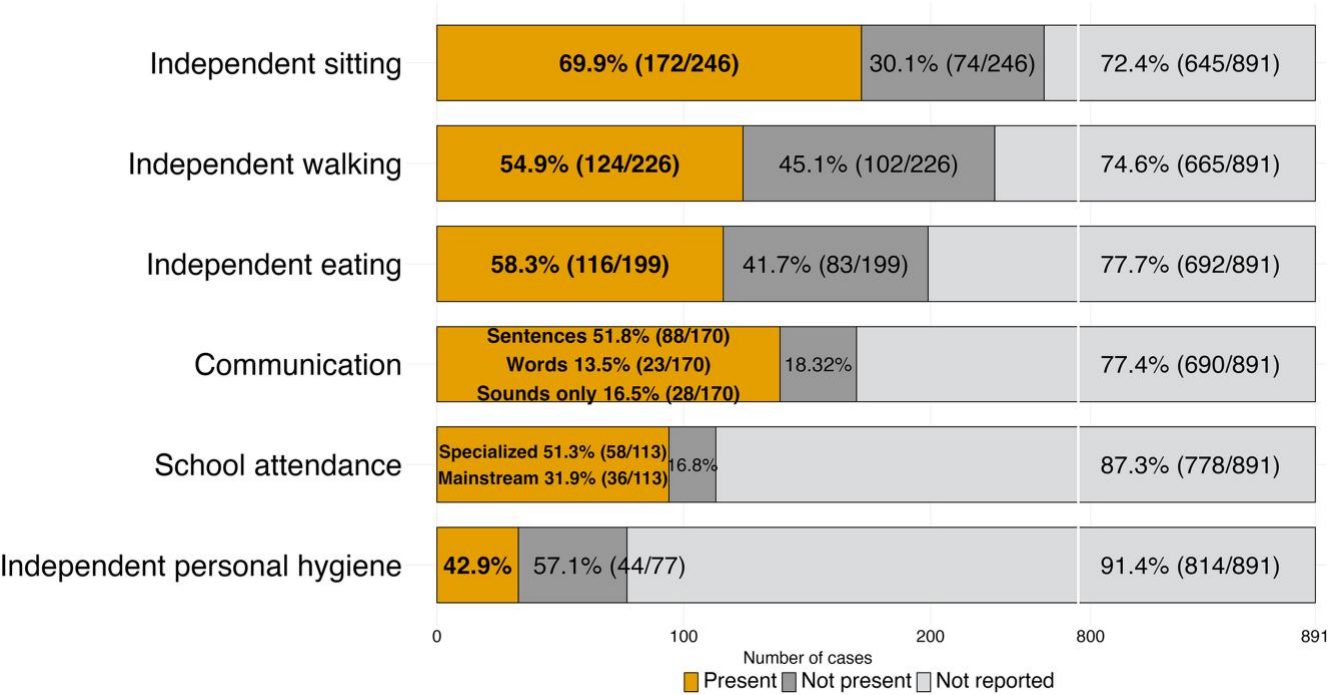
**Affected activities of daily living**. Figure shows the activities of daily living summarized among the included cohort; ‘regular school’ refers to mainstream school. Findings are listed in decreasing frequency from the top. In the subgroup with available data on communication (*n* = 170), 18.3% (31/170) were reported as non-communicating. In the subgroup with available data on school attendance (*n* = 113), 16.8% (19/113) did not attend any school. In the subgroup with available data on personal hygiene (*n* = 77), 42.9% (33/44) were independent with personal hygiene.

Phenotypic information was available for 674/891 (76%) cases and is summarized in Figs 5–8.

#### Pregnancy, delivery, anthropometric data at birth

Fetal and perinatal abnormalities were reported in 47% (159/342) (Fig. 5).

#### Neurological and neuroradiological findings

The most frequent findings were DD, ID, muscle hypotonia, abnormal movements, seizures, feeding difficulties and microcephaly, all found in more than 50% of cases (Fig. 6). Brain MRI, CT, ultrasound or post-mortem findings were reported in 554 cases. Only 27 individuals had unremarkable neuroimaging (CT/MRI). Common anomalies included basal ganglia alterations (217/554, 39%), cerebral atrophy (211/554, 38%) and corpus callosum (CC) anomalies (151/554, 27%) (Fig. 7).

#### Other findings

Non-neurological features were reported (Fig. 6): dysmorphic features (e.g. wide nasal bridge, hypo-/hypertelorism), abnormal skeletal morphology (e.g. rhizomelia, scoliosis) and osteopenia.

#### Activities of daily living

Only cases older than age 1 year for sitting, 2 years for walking and eating, 4 years for communication,^37^ 8 years for school attendance and 11 years for independent hygiene^38^ were considered (data available for 77–246 cases) (Fig. 8). Most individuals could sit unassisted. About half walked unassisted, fed themselves, communicated with sentences or attended a specialized school. Forty per cent were independent with hygiene. One-third communicated with words or only with sounds (Fig. 8). One-third (36/113) attended or completed mainstream school. Overall, males had greater independence with the remaining activities of daily living (ADLs) (Supplementary Table 15).

#### Mild phenotypes

Within our cohort, 4% (36/891) were reported to have no DD or ID (25/36 males; median age at last evaluation: 15.2 years, range 6.2–52 years; 28/36, 77.8% unpublished), with 21 (out 22 with available data) individuals attending or finishing regular school. Compared with those with DD/ID, these individuals had lower frequency of muscular hypo-/hypertonia, feeding difficulties, seizures, visual or hearing impairment (Supplementary Table 16). Exact (clinical) findings prompting diagnostics in this subgroup were not collected. Notably, none had microcephaly (0/30), dysmorphic features (0/28) or abnormal skeletal morphology (0/29). However, 71% (22/31) showed basal ganglia lesions (age range at MRI not available), and only three cases had unremarkable neuroimaging.

### Intervention with ketogenic diet/thiamine

KD use was reported in 226/328 (69%) and thiamine supplementation in 316/356 (89%), of which 201 cases received both KD and thiamine. Physicians considered the KD successful in 160/226 (71%) and thiamine supplementation in 154/316 (49%).

### Genotype-sex-phenotype correlations

#### Correlations for fetal onset, perinatal complications and first presentation

Male sex and variants affecting exon 3 lowered the odds of fetal findings by 47% and 93%, respectively (Supplementary Table 17 and Supplementary Fig. 7). FS/N variants in the NMD-predicted region 3.9 (95% CI 1.54–11.04) times increased the odds of fetal findings. Males were at 3.7 (95% CI 1.71–8.28) times higher risk of prematurity (birth before 37th gestational week) (Supplementary Table 18 and Supplementary Fig. 7). Prenatal findings increased the risk of birth anthropometrics below the 3rd percentile, resuscitation at birth and (or) Apgar scores below five (Supplementary Tables 19 and 20 and Supplementary Fig. 7).

Age at presentation varied by sex, with females presenting earlier (Supplementary Fig. 5). In multivariable logistic regression analysis (*n* = 252), males harbouring variants in exons 3 and 8 had reduced risk of presenting in the neonatal period (Supplementary Table 21 and Supplementary Figs 5 and 8). In a female regression model (*n* = 254), females harbouring FS/N variants in the NMD-predicted region or variants in exon 10 had three times increased risk of neonatal presentation (Supplementary Table 22 and Supplementary Fig. 8).

Among the most common variants (at least 10 cases per variant), data on age at presentation were insufficient to draw any conclusions (Supplementary Tables 23 and 24).

#### Correlations for survival

Overall, published cases reported more deceased cases, which significantly influenced all survival prediction models (Supplementary Tables 10, 13, 14, 25 and 26 and Supplementary Fig. 9). Male sex [hazard ratio (HR) 3.3 (1.95–5.62)], neonatal presentation [HR 5.5 (2.17–14.09)], FS/N variants in the NMD-predicted region [HR 4.0 (1.78, 9.16)] and splice variants [HR 2.3 (1.15, 4.59)] increased the risk of lethal outcome up to 18 years (Fig. 4D and Supplementary Table 13). FS/N variants in the NMD-predicted region were significant predictors for death in female subanalysis (Supplementary Tables 25 and 26).

#### Correlations for phenotype

Mild phenotypes (no DD/ID) were more common among males and unpublished cases (Supplementary Tables 16 and 27–29). None of the mild cases harboured splice variants, compared with 26 cases with DD/ID. Variant type distribution was similar between cases with and without DD/ID. In total, 44% (12/27) of variants in cases without DD/ID were private, compared with 46% (146/318) in cases with DD/ID (*P* = 0.234). Male sex and variants in exon 3 were protective factors for DD, but not for ID. Neonatal and infantile presentation increased the odds of DD by 6.4 and 9.5 times, and the odds of ID by 13.4 and 16.6 times, respectively (Supplementary Tables 27 and 28 and Supplementary Fig. 10).

Among the most common variants (at least 10 cases per variant), males had a tendency of homogeneous phenotypes of the same variants (Supplementary Tables 30 and 31 and Supplementary Fig. 11). Given the small number of cases, conclusions are limited.

Sex and age at presentation were the strongest phenotype predictors (Supplementary Figs 10 and 12–14 and Supplementary Tables 32–50). Male sex increased the odds of peripheral neuropathy and abnormal basal ganglia findings. Female sex increased the odds of DD, muscle hypertonia, microcephaly, seizures, dysmorphic features, visual impairment, abnormal skeletal morphology, cerebral atrophy, CC malformations, and ventriculomegaly or hydrocephalus. Neonatal and infantile presentations increased the odds of DD, ID, microcephaly, seizures, feeding difficulties and dysmorphic features. In contrast, later presentations (childhood) associated with abnormal movements and peripheral neuropathy.

After adjusting for sex, age at presentation and data source, genotype associated with several phenotypes (Supplementary Figs 10 and 12–14 and Supplementary Tables 27, 28 and 32–50). Notably, splice variants increased the odds of muscle hypotonia or hypertonia and seizures. FS/N variants in NMD-predicted regions were linked to microcephaly.

### Effects of mixed case inclusion

Survival analysis was the most affected part by mixed inclusion of published and unpublished cases (see survival sections). Furthermore, cases source influenced prenatal findings, ID, muscle hypotonia, microcephaly, seizures, feeding difficulties, dysmorphic features, abnormal movements, peripheral neuropathy, visual impairment, abnormal skeletal morphology, nystagmus, ophthalmoplegia, cerebral atrophy, basal ganglia findings, and ventriculomegaly or hydrocephalus (Supplementary Tables 17, 27, 28, 32–40, 42, 44, 45, 47, 48 and 50).

### Metabolic and biochemical details

Elevated lactate in serum (509/564, 90%), CSF (262/280, 94%) or urine (129/199, 65%) were common findings (Supplementary Fig. 15). The lactate/pyruvate ratio was not collected.

Residual PDHc enzyme activity (range: 0%–100%) did not significantly differ across tissue types (fibroblasts, lymphocytes and muscle). The median enzyme activity was 32% (IQR 28.4) in fibroblasts (*n* = 305), 31% (IQR 47) in lymphocytes (*n* = 79) and 36% (IQR 39.7) in muscle (*n* = 66). There was considerable variability within each case and tissue type, leading to significant heterogeneity (Supplementary Table 51), with inconclusive association with genetic defects. In females harbouring missense variants, residual enzyme activity (fibroblasts) was higher compared with males harbouring FS/N variants in the NMD-escape region (Supplementary Fig. 16). In males, cases harbouring variants in exon 8 had higher activity compared with exon 11. There were no other significant residual enzyme activity (fibroblasts) differences between subgroups stratified by sex, age at first presentation, variant type and affected exon.

## Discussion

Here, we report the largest single-gene defect cohort of 891 patients with 331 unique variants among all nuclear-encoded genes involved in mitochondrial energy metabolism. This study indicates that *PDHA1*-related PDHc deficiency is among the most frequent mitochondrial disorders.

### The genetic landscape of *PDHA1*-related PDHc deficiency

#### An X-linked disorder with similar female to male ratio

This study supports previous findings of similar affected male and female proportions.^39,40^ Female manifestation is strongly influenced by skewed XCI^41,42^ and possibly other less studied mechanisms (unfavourable cell selection in mosaicism, partial expression of the inactive X chromosome). The proportion of affected females varies across X-linked metabolic conditions: from no affected females in Barth syndrome (MIM #302060),^42^ few symptomatic carrier females in (cerebral) creatine deficiency syndrome 1 (MIM #300352),^43^ to many in ornithine transcarbamylase deficiency (OTC, MIM #311250).^44^ The nearly equal number of females with *PDHA1*-related PDHc deficiency may relate to the high proportion (75%) of *de novo* variants, which was even higher in females (87%) than males (63%). A previous population-based study of *PDHA1*-related PDHc found 86% *de novo* variants in the probands.^45^ This suggests that severe variants are more likely to be symptomatic in females but may be lethal in males. Other X-linked disorders show lower *de novo* variant rates of around 30% [Lesch Nyhan syndrome (MIM #300322),^46^ haemophilia A (MIM #306700),^47^ Duchenne muscular dystrophy (MIM #310200),^48^ Danon disease (MIM #300257)^49^ or 13% in Barth syndrome (MIM #302060)^50^ and 14% in Fabry disease (MIM #301500)].^51^ Often, neurodevelopmental disorders have high *de novo* occurrence as fitness renders transmission down the germ line unlikely.^52,53^ No gene regions were prone to *de novo* variants, and whether a sequence pattern predisposes to *de novo* recurrence is unclear.

#### Sex-specific genotype differences: protein loss-of-function variants are lethal in males

Of the 891 affected individuals, 69% (227/331) harboured private variants. The higher proportion of private variants in *PDHA1* than in gnomAD (51%)^35^ could suggest selection bias, both from evolutionary and study versus general population perspectives. FS/N variants (considered as pLoF) with higher pathogenic potential were enriched due to clinical ascertainment of affected individuals. Half of the FS/N variants in females were in the NMD-predicted region, potentially tolerated due to skewed XCI, or other unidentified mechanisms. Indeed, the XCI ratio is suggested to be related with disease severity in *PDHA1*-related PDHc deficiency.^54^ The remaining variants in females, and the majority in males, occurred in the distal region predicted to escape NMD, leading to a truncated protein (Fig. 3). Truncated *PDHA1-*encoded proteins can still catalyse pyruvate decarboxylation, albeit with reduced efficacy or stability, allowing hemizygous males to survive *in utero*.^3^

Only two male cases with FS/N variants in NMD-predicted regions were found (Fig. 3), one had confirmed mosaicism. This aligns with other X-linked disorders, where pLoF variants are more common in females, as males with a non-functional gene often result in fetal death.^42^ In a PDHc deficiency mouse model, males also die prenatally.^55^ Male mosaicism in *PDHA1*-related PDHc deficiency is likely underdiagnosed, as in other X-linked disorders.^56,57^ These observations suggest a genotype-sex-phenotype correlation: pLoF variants that eliminate protein production are typically lethal but may be detected due to protective mechanisms (e.g. XCI, mosaicism).

#### Genetic defects landscape reflects functional protein regions and post-transcriptional regulations

The E1α subunit terminal region is essential for the interaction with the E1β subunit (encoded by the *PDHB* gene) to form the E1 heterotetramer (Fig. 3B and D).^58-60^ The catalytic centres are located at the subunit interface. Exons 3–9 had the fewest variants, while the N- and C-terminal regions showed greater variant prevalence in gnomAD. In this study, mutational hotspots were from exon 5 forward (Supplementary Table 8), suggesting selection bias. Although coding variants in exons 1–2 are present in the gnomAD database,^35^ there were two in exon 1 and none in exon 2 in this and previous studies.^61^ This may indicate tolerated variants in the N-terminal regions that do not cause a significant phenotype.

Structural and functional studies show that missense variants can completely abolish PDHc activity.^59,61,62^ In the present study, missense variants are the leading cause of *PDHA1*-related PDHc deficiency (50%), especially in males (76%). Missense variant pathogenicity is location dependent, affecting substrate-enzyme affinity, cofactor-ligand conformation, phosphorylation (which regulates PDHc activity) and protein-protein interfaces.^59-62^ Protein-protein interface regions are often enriched for tryptophan, tyrosine and arginine, with conserved buried residues.^61,63^ Replacement of arginine residues accounted for 39% of missense variants in a previous study^61^ and 53% in this study (Supplementary Table 6). As in previous studies,^64^ nearly all cases harbouring p.Arg302Cys were females (36/38, sex unspecified in two), with two male exceptions—one confirmed as mosaic. Other variants at p.Arg302 (Supplementary Table 6) were also predominantly found in females. These findings could be partially explained by p.Arg302 locating near the conserved phosphorylation loop essential for PDHc activity regulation.^59,60,62^ Alanine-169 is located at the E1α-E1β heterodimer interface within the TPP binding region, p.Ala169Val variants were mostly found in females, indicating lethality for males when critical protein regions are affected (Supplementary Table 6).^62,65^ While nonsense variants at p.Arg263 were only found in females (likely lethal for males), missense variants at the same position (p.Arg263Gly, p.Arg263Gln, p.Arg263Pro) were mostly found among males (Supplementary Table 6). Arginine-263 is located near a highly conserved phosphorylation site, interacts with the TPP diphosphate tail and facilitates acetyl group transfer from TPP to the lipoyl domain of E2, crucial for substrate intake, as evidenced by near-zero PDHc activity when replaced.^3,59,61,66-68^ It is thus not surprising that p.Arg263Gly was the most common variant. Furthermore, exon location—adjusted for variant type—associated with fetal findings, presentation and phenotypes. Most mild cases harboured variants in exon 3 (Supplementary Figs 7, 8, 10, 12 and 14). These observations coupled with previous studies support a genotype-sex-phenotype correlation: disease severity is influenced by the affected gene region corresponding to regulatory, structural or enzymatic protein domains.

In contrast to previous studies,^11,39,60^ our results demonstrated that FS/N variants in NMD-predicted, the pLoF variants, associated with fetal onset (Supplementary Fig. 7), worse survival (splice variants as well) (Fig. 4), earlier presentation (Supplementary Fig. 8) and microcephaly (Supplementary Fig. 12). Thus, we propose a genotype-sex-phenotype correlation: more severe genotypes (e.g. the FS/N variants in NMD-predicted regions in females) result in more severe phenotype.

Finally, our results indicate that males harbouring the same variants exhibit more homogeneous phenotypes than females (Supplementary Tables 30 and 31 and Supplementary Fig. 11). While the XCI ratio is suggested to be linked with disease severity in females,^54^ males have only one X chromosome. This suggests that PDHc activity could be consistently affected by the same variants in males. However, interpretability is limited by insufficient data availability.

### Phenotypic spectrum of *PDHA1*-related PDHc deficiency

Our data confirm and extend the previously reported neurological signs and symptoms in this disorder (Figs 6 and 7 and Supplementary Tables 17, 27, 28 and 32–50).^2,39,40,69^ The known CNS dependency on PDHc function explains the clinical phenotype of (nearly) isolated neurological phenotype.^6,7^ Other findings like feeding difficulties, dysmorphic features and skeletal abnormalities may well be secondary to the neurological phenotype.^70,71^ Notably, the multisystemic phenotype typically observed in mitochondrial disorders is absent in *PDHA1*-related PDHc deficiency and should be considered during follow-up.^72^

Early disease presentation negatively correlated with survival both in smaller studies and this one.^39,40^ This aligns with characteristics of mitochondrial diseases: early presentation is linked to a more severe phenotype.^73^ In addition, this study provides clinically relevant age at presentation-based poor survival predictors. Phenotypical sex differences were inconsistent in previous studies,^39,40^ while this study presents significant clinical, age at presentation and survival distinctions. Worse survival in males (Fig. 4) aligns with observations in other X-linked disorders.^42^

#### PDHc deficiency affects brain development

The prenatal and early postnatal periods are critical for brain development, demanding high levels of energy and underscoring the essential role of PDHc.^74,75^ It is hypothesized that energy insufficiency during these stages may disrupt normal neuronal proliferation, migration and differentiation.^76,77^ The structural brain abnormalities identified in our study [abnormal dentate nuclei, cerebellar cortex growth, CC, corticospinal tract abnormalities and microcephaly (Fig. 7)] align with previous findings, supporting this hypothesis.^75-79^ Additionally, early presentation increased the odds of DD, ID, microcephaly, seizures and feeding difficulties (Supplementary Figs 10 and 12), further linking early brain damage to more severe phenotype and worse survival.^80^ Intrauterine growth restriction (27% versus 5%–10%), microcephaly (20% versus 1%) and low weight (19% versus 15%) at birth were more common than in the general population, illustrating prenatal disease onset.^81-83^

A second mechanism resulting from energy failure is cell death, leading to cortical and white matter atrophy, calcified migrating neurons, basal ganglia calcifications or lesions.^76,84,85^ Furthermore, some of the findings may be secondary. Obstructive hydrocephalus can lead to a secondary loss of the CC myelinated fibres or cerebral atrophy.^86,87^ The latter may result in hydrocephalus *ex vacou*. In the current study, cerebral atrophy, CC malformations, hydrocephalus or ventriculomegaly—generally signs of more severe phenotype—were more prominent in females (Supplementary Fig. 14). Severe energy deficiency in early neurogenesis that could result in such findings is likely lethal for males, whereas affected females may survive depending on XCI skewing.^39,76,84^

#### Neurodevelopmental and movement spectrum disorder

Abnormal muscle tone (hypotonia, hypertonia) and abnormal movement (e.g. ataxia, dystonia, various dyskinesias) were present in over two-thirds of patients and reported previously.^2,39,40,69^ These findings may be partially explained by CNS being PDHc dependent and by basal ganglia, cerebellum, cortex or corticospinal tract involvement.^88^ Furthermore, muscle cells utilize both glucose and fatty acids for energy; thus, defective PDHc may contribute to these phenotypes not solely through CNS involvement.^60^ Peripheral neuropathy was observed in 38% of cases. However, nerve conduction studies are not routinely included in the work-up for children with suspected DD/ID in general and PDHc deficiency in particular.^89,90^ Peripheral neuropathy could be similar to other conditions, for example, 10 cases were reported to have Guillain–Barre syndrome-like presentations.^91-95^ Because of its ability to activate PDHc by inhibiting pyruvate kinase (PDK), DCA has been used to treat congenital lactic acidosis, including PDHc deficiency.^14-16,65,96^ DCA is known to induce peripheral neuropathy.^97^ This may be a confounding factor in single cases. DCA use was not addressed in this study.

It remains unclear whether increased lactate may cause neurological damage beyond energy deficiency, or if it is even beneficial. Animal models and case reports have shown exacerbation of ataxia after carbohydrate feeding.^64,98^ In contrast, according to the astrocyte-neuron-lactate shuttle hypothesis, the lactate produced in astrocytes is taken up by neurons to convert to pyruvate as an energy source.^99^ Research has suggested that lactate is a beneficial energy source, although these do not specifically address PDHc deficiency.^100,101^

#### *PDHA1*-related mitochondrial disease phenocopies

In our cohort, premature delivery, resuscitation at birth and low Apgar scores were more frequent than in the general population.^102-104^ All are major predictors for long-lasting neurological complications, including DD,^103-106^ and developing brain injury, resulting in cerebral palsy.^107-109^ Consequently, PDHc deficiency is a known ‘cerebral palsy mimic’ disorder.^110^ Moreover, PDHc deficiency can present as developmental epileptic encephalopathies (DEE).^111-113^ Structural brain changes after hypoxic-ischaemic perinatal damage may overlap with *PDHA1*-related PDHc deficiency as well.^114^

#### Mild phenotype does not exclude the diagnosis

This study further emphasizes mild phenotypes (no DD/ID) in 4% of the cohort^45,94,115^ and highlights clinical differences (e.g. no microcephaly, dysmorphic features or drooling in the absence of DD/ID) (Supplementary Table 16). The lower prevalence of mild phenotypes in females (Supplementary Table 16) likely relates to males with severe phenotype early deaths (most neonatal males died within 1 year) and underdiagnosis of mild phenotypes in females.^45^ A population-based study identified affected females with peripheral neuropathy, *pes cavus* and mild symptoms after diagnosing their offspring.^45^ In this study, one-third was diagnosed via NGS, which may identify milder cases that went unrecognized before the NGS era.^116^ This study confirms that a mild phenotype does not exclude *PDHA1*-related PDHc deficiency. It is recommended to carry out phenotyping of female ‘carriers’ in the patients’ families.

#### *In vitro* PDHc enzyme activity does not reflect disease severity

Enzyme activity reports within this study were retrospectively gathered from multiple sources, different tissues and used varied calculation methods (Supplementary Table 51), resulting in large heterogeneity. In present and previous studies, high variation in PDHc activities were observed, even within the same subject.^39^ In this study, the PDHc activity level in males did not correlate with variant type, contrasting previous reports.^40^ Due to XCI in females, residual enzyme activity in cultured fibroblasts may not reflect variant severity, since cellular heterogeneity is typically uncorrected for.^117^

### Limitations

The retrospective study design relied on data from multiple sources with varying availability, collected over more than 30 years. Cases from the literature, often published in the 1990s and 2000s, were typically diagnosed by single gene analysis and more frequently described severe phenotypes, including more lethal cases. In contrast, unpublished cases were more often diagnosed by NGS, had less lethal cases and more mild phenotype reports. Moreover, the source of data also influenced the detection frequency of several clinical findings. Phenotypes were recorded in a binary manner without age data, excluding the ability to analyse changes over time. This study analysed neuroimaging reports, relying on the source interpretations rather than images themselves. Major limitations include variability in sources, modalities and examiners. The ethnicity was not assessed.

### Treatment options influence future directions

Ketogenic diet and thiamine supplementation are the pathomechanism-based, widely used, interventions. We reiterate that this retrospective study design was not suited to assess treatment outcomes. Consistent with previous reports,^13,118-120^ treating physicians considered KD and thiamine supplementation successful. To date, only one study evaluated the short- and long-term effects of KD.^13^ This underlines the high urgency for prospective clinical trials evaluating the preferred KD modality (e.g. 4:1, modified Adkins diet, low glycaemic index diet), the target ketone body range and the thiamine supplementation dose. In this context, short- and long-term clinical and biochemical outcome measurements need to be defined. Importantly, a murine model showed that maternal KD benefits offspring brain development.^121^ A PDHc deficiency zebrafish model demonstrated improved neurological function and lower embryonic lethality with prenatal KD.^122^ Phenylbutyrate is reported to inhibit PDK and to stimulate PDC activity in fibroblasts.^123^ As of 5 September 2025, the Food and Drug Administration (FDA) declined to approve oral DCA treatment for PDHc deficiency^124^ (NCT02616484). These observations warrant a prospective assessment of early treatment effects and genetic investigations in children with suspected *PDHA1*-related PDHc deficiency.

## Supplementary Material

awaf430_Supplementary_Data

## Data Availability

The data supporting this study are available from the corresponding author upon reasonable request and will be considered on an individual basis.
