# Supplementary material for: The genotypic and phenotypic landscape of PDHA1-related pyruvate dehydrogenase complex deficiency
Source: Brain. 2025 Nov 14;149(7):2344–62. doi: 10.1093/brain/awaf430 (PMC13337232; doi:10.1093/brain/awaf430)
Supplement: awaf430_Supplementary_Data [file awaf430_supplementary_data.zip › brain-2025-01850-File010.pdf]

The genotypic and phenotypic landscape of *PDHA1*-related Pyruvate dehydrogenase complex deficiency

SUPPLEMENTARY MATERIAL

Supplementary Figures 5

Supplementary Figure 1. ***The distribution of participating collaborators across world map*** 5

Supplementary Figure 2. ***Genomic positions of splice and other non-coding variants (variants n = 31, cases n = 59)*** 6

Supplementary Figure 3. ***Distribution of missense variants in this study compared to AlphaMissense predictions across PDHA1 gene*** 7

Supplementary Figure 4. ***Coding variant distribution among PDHA1 exons in the cohort and gnomAD database*** 8

Supplementary Figure 5. ***Age at presentation stratified by gender and variant type*** 10

Supplementary Figure 6. ***Distribution of age at last report and subset selection for survival analysis*** 18

Supplementary Figure 7. ***Significant predictors of prenatal or perinatal findings*** 20

Supplementary Figure 8. ***Significant predictors of neonatal presentation in males and females*** 22

Supplementary Figure 9. ***Multiple imputation sensitivity survival analysis of known and undefined cases with Kaplan-Meier estimator*** 24

Supplementary Figure 10. ***Significant predictors of developmental delay, intellectual disability, muscle hypotonia, and hypertonia*** 26

Supplementary Figure 11. ***Clinical phenotype homogeneity in cases with the most common variants (at least 10 cases per variant)*** 28

Supplementary Figure 12. ***Significant predictors of microcephaly, seizures, feeding difficulties, and dysmorphic features*** 31

Supplementary Figure 13. ***Significant predictors of abnormal movements, peripheral neuropathy, visual impairment, and abnormal skeletal morphology*** 33

Supplementary Figure 14. ***Significant predictors of cerebral atrophy, basal ganglia findings, corpus callosum malformations, and ventriculomegaly or hydrocephalus*** 35

Supplementary Figure 15. ***Summary of the most common laboratory findings*** 37

Supplementary Figure 16. ***Enzyme activity in fibroblasts stratified by sex, variant type, affected exon and age at presentation*** 38

Supplementary Tables 43

Supplementary Table 1. ***Critical appraisal of case reports selected for case inclusion*** 43

Supplementary table 2. ***Critical appraisal of case series selected for case inclusion*** 46

Supplementary Table 3. ***Data extraction form*** 51

Supplementary Table 4. ***List of high risk duplicate cases from the literature*** 55

Supplementary table 5. ***Pathogenicity interpretation details of PDHA1 variants considered to include in the study using ACMG criteria*** 62

Supplementary Table 6. ***PDHA1 variants included in the study in decreasing frequency*** 84

Supplementary Table 7. ***Disease-causing missense variant discovery rate in PDHA1 and other genes.*** 106

Supplementary Table 8. ***Coding variant distribution among PDHA1 exons in the cohort and gnomAD database*** 107

Supplementary Table 9. ***Comparison of sex-related enrichment for the most common variants (10 ≥ cases per variant)*** 108

Supplementary Table 10. ***Comparison of published and unpublished cases by sex, presentation, last report, and survival (n = 316)*** 109

Supplementary Table 11. ***Survival estimates stratified by maximum observation duration*** 110

Supplementary Table 12. ***Pairwise comparisons of sex and age at presentation from Cox PH model with Holm–Bonferroni adjustment*** 111

Supplementary Table 13. ***Univariate and multivariable Cox regression models of survivor up to 18 years (n = 240)*** 112

Supplementary Table 14. ***Univariate and multivariable Cox regression models of survivor up to 18 years including prenatal or perinatal findings (n = 123)*** 113

Supplementary Table 15. ***Sex-related differences in activities of daily living*** 115

Supplementary Table 16. ***Clinical phenotypes among cases with no, mild, and severe developmental delay and (or) intellectual disability*** 116

Supplementary Table 17. ***Univariate and multivariable models of prenatal (fetal) findings (n = 292)*** 117

Supplementary Table 18. ***Univariate and multivariable models of prematurity (n = 246)*** 118

Supplementary Table 20. ***Univariate and multivariable models of resuscitation at birth and (or) APGAR scores ≤ 5 (n = 220)*** 120

Supplementary Table 21. ***Univariate and multivariable models of neonatal presentation in males (n = 252; compared to later presentations)*** 122

Supplementary Table 22. ***Univariate and multivariable models of neonatal presentation in females (n = 254; compared to later presentations)*** 123

Supplementary Table 23. ***Prenatal (fetal), perinatal findings and age at presentation characteristics in males with most common variants (at least 10 cases per variant)*** 124

Supplementary Table 24. ***Prenatal, perinatal findings and age at presentation characteristics in females with most common variants (at least 10 cases per variant)*** .. 125

Supplementary Table 25. ***Univariate and multivariable Cox regression models of survivor in females up to 18 years (n = 106)*** 126
Supplementary Table 26. ***Univariate and multivariable Cox regression models of survivor up to 18 years in males with missense variants (n = 73)*** 127
Supplementary Table 27. ***Univariate and multivariable models of developmental delay (n = 363)*** 128
Supplementary Table 28. ***Univariate and multivariable models of intellectual disability (n = 215)*** 129
Supplementary Table 29. ***Variants in cases with no developmental delay and no intellectual disability (variants n = 27, cases n = 36)*** 130
Supplementary Table 30. ***Clinical phenotype homogeneity in males with the most common variants (at least 10 cases per variant)*** 132
Supplementary Table 31. ***Clinical phenotype homogeneity in females with the most common variants (at least 10 cases per variant)*** 133
Supplementary Table 32. ***Univariate and multivariable models of muscle hypotonia (n = 400)*** 134
Supplementary Table 33. ***Univariate and multivariable models of muscle hypertonia (n = 303)*** 135
Supplementary Table 34. ***Univariate and multivariable models of microcephaly (n = 315)*** 136
Supplementary Table 35. ***Univariate and multivariable models of seizures (n = 305)*** 137
Supplementary Table 36. ***Univariate and multivariable models of feeding difficulties (n = 287)*** 138
Supplementary Table 37. ***Univariate and multivariable models of dysmorphic features (n = 281)*** 139
Supplementary Table 38. ***Univariate and multivariable models of abnormal movements (n = 323)*** 140
Supplementary Table 39. ***Univariate and multivariable models of peripheral neuropathy (n = 281)*** 141
Supplementary Table 40. ***Univariate and multivariable models of visual impairment (n = 243)*** 142
Supplementary table 41. ***Univariate and multivariable models of hearing impairment (n = 219)*** 143
Supplementary Table 42. ***Univariate and multivariable models of abnormal skeletal morphology (n = 258)*** 144
Supplementary Table 43. ***Univariate and multivariable models of strabismus (n = 245)*** 145
Supplementary Table 44. ***Univariate and multivariable models of nystagmus (n = 250)*** 146
Supplementary Table 45. ***Univariate and multivariable models of ophthalmoplegia (n = 207)*** 147
Supplementary Table 46. ***Univariate and multivariable models of drooling (n = 207)*** 148
Supplementary Table 47. ***Univariate and multivariable models of cerebral atrophy (n = 448)*** 149
Supplementary Table 48. ***Univariate and multivariable models of basal ganglia findings (n = 448)*** 150
Supplementary Table 49. ***Univariate and multivariable models of corpus callosum malformations (n = 448)*** 151

Supplementary Table 50. ***Univariate and multivariable models of ventriculomegaly or hydrocephalus (n = 448)*** *152*

Supplementary Table 51. ***Heterogeneity of enzyme activity values among cases with available calculation methods*** *153*

Supplementary references 154

PRISMA 2020 Checklist 169

Supplementary Figures

Supplementary Figure 1. **The distribution of participating collaborators across world map**

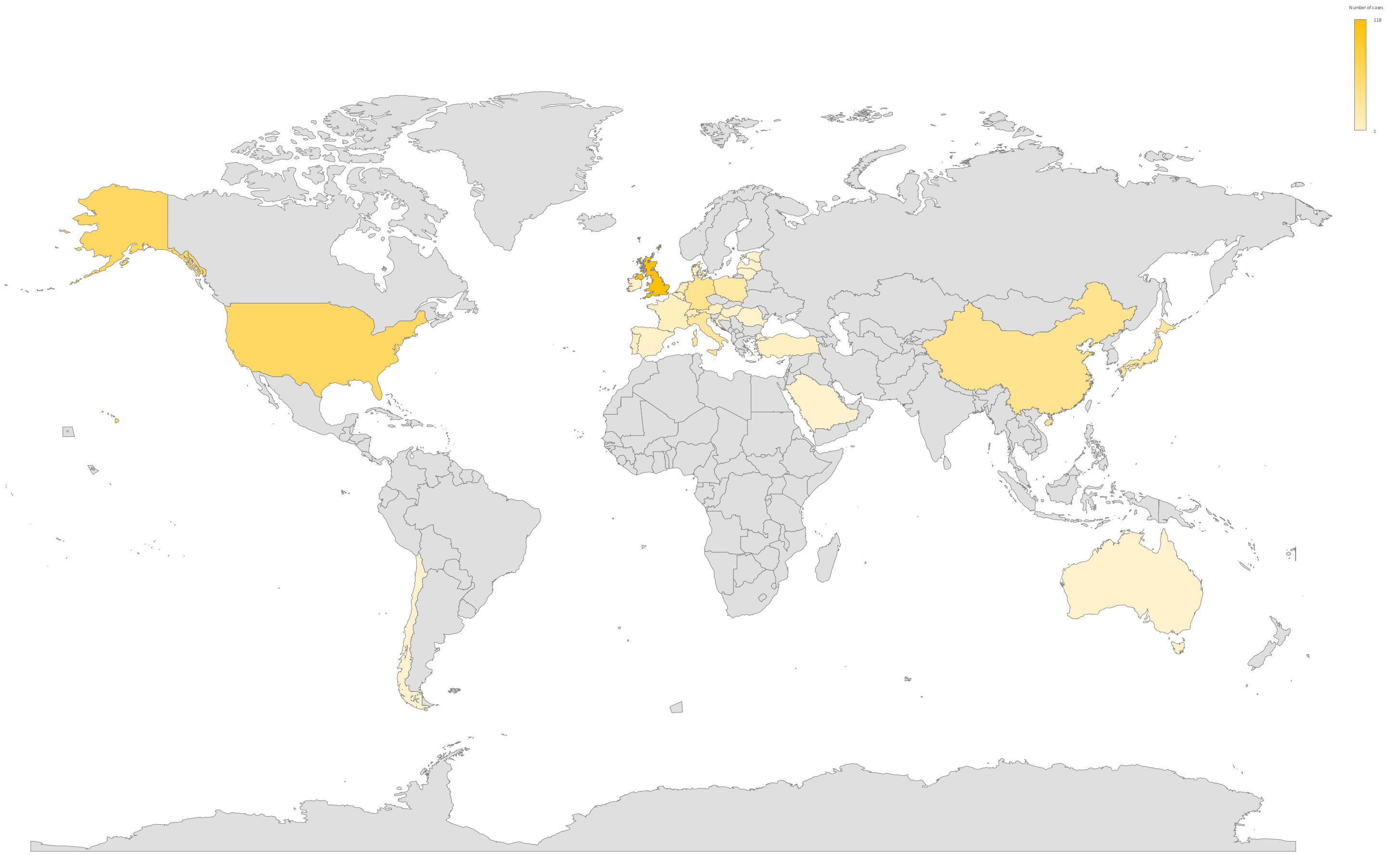

Supplementary figure 1 depicts the countries from which various international collaborators participated in this study. Yellow color depicts number of included cases per country, from light as low to dark as high.

Supplementary Figure 2. **Genomic positions of splice and other non-coding variants (variants *n* = 31, cases *n* = 59)**

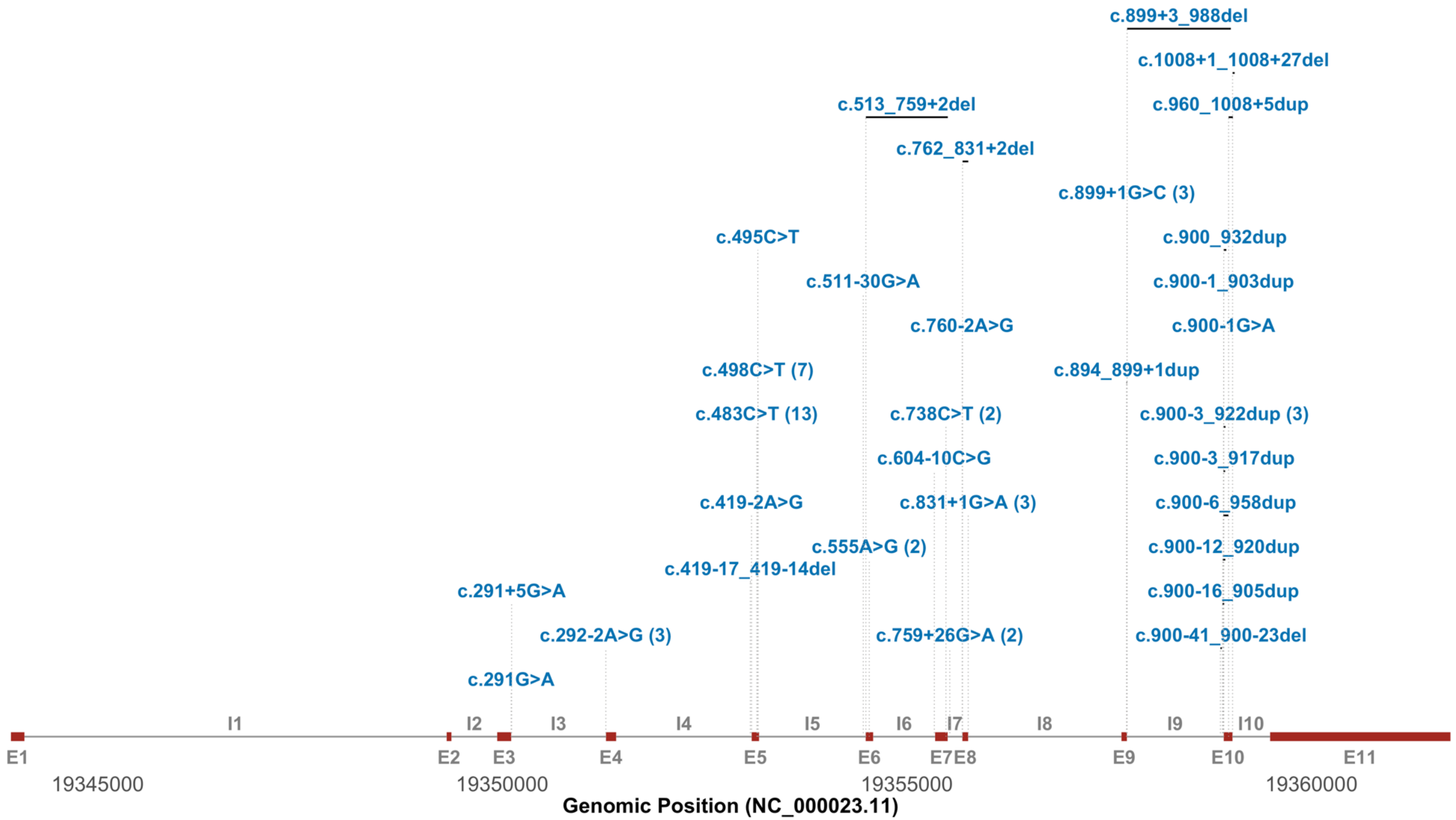

Supplementary figure 2 shows the distribution of splice variants. If more than one case with a specific variant was present, the number in brackets indicates the number of cases. A dotted line marks the genomic position of the first nucleotide. Genomic positions are provided according to the X chromosome reference sequence (NC_000023.11, GRCh38). Exons (dark red) and introns (grey) are represented on the x-axis as E1 to E11 and I1 to I10, respectively.

Supplementary Figure 3. **Distribution of missense variants in this study compared to AlphaMissense predictions across *PDHA1* gene**

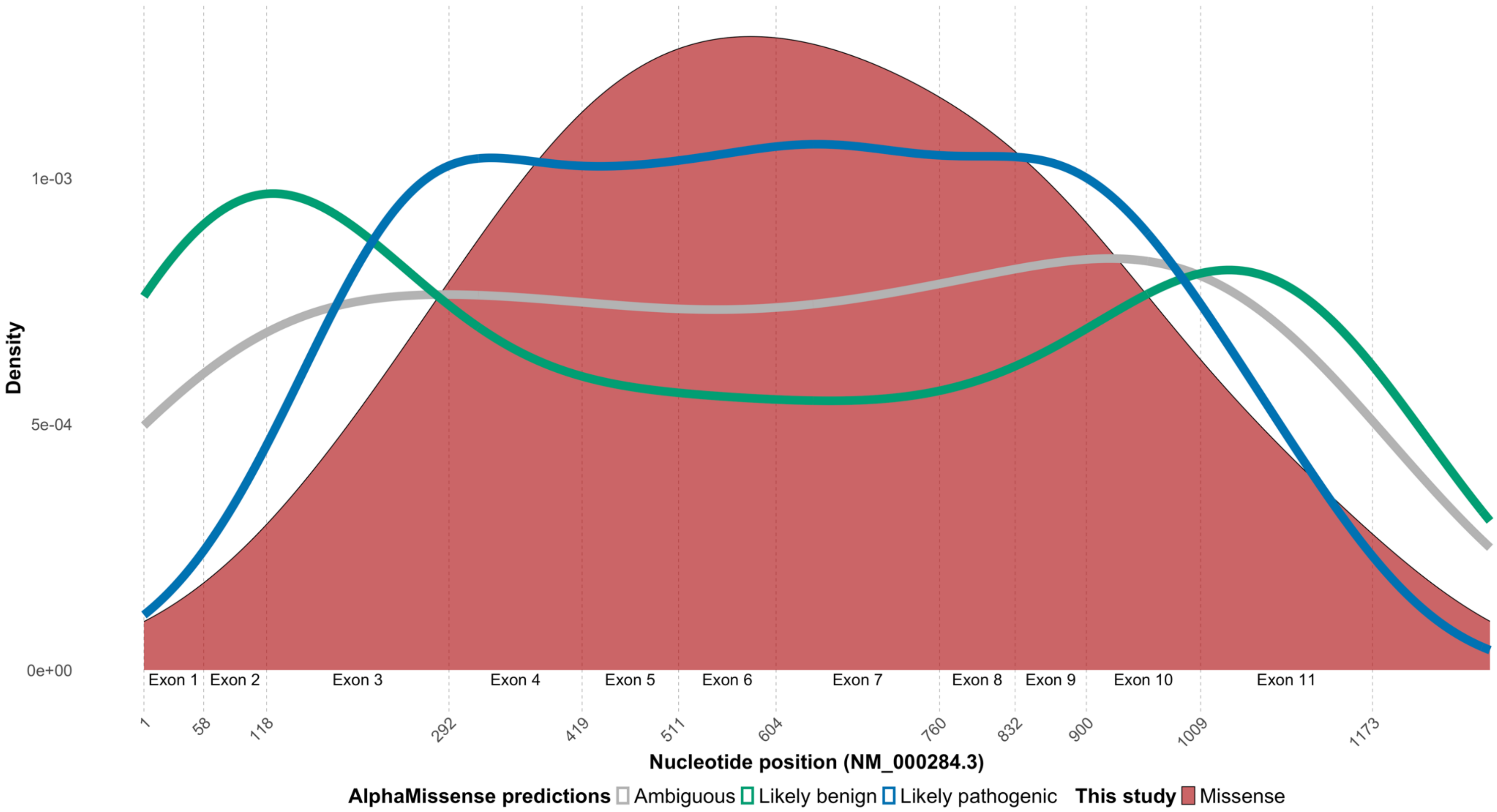

Kernel density plot showing the distribution of missense variants identified in this study (red fill) compared with AlphaMissense predictions of variant pathogenicity (colored lines). Vertical lines indicate exon boundaries based on PDHA1 reference transcript NM_000284.3

Supplementary Figure 4. **Coding variant distribution among *PDHA1* exons in the cohort and gnomAD database**

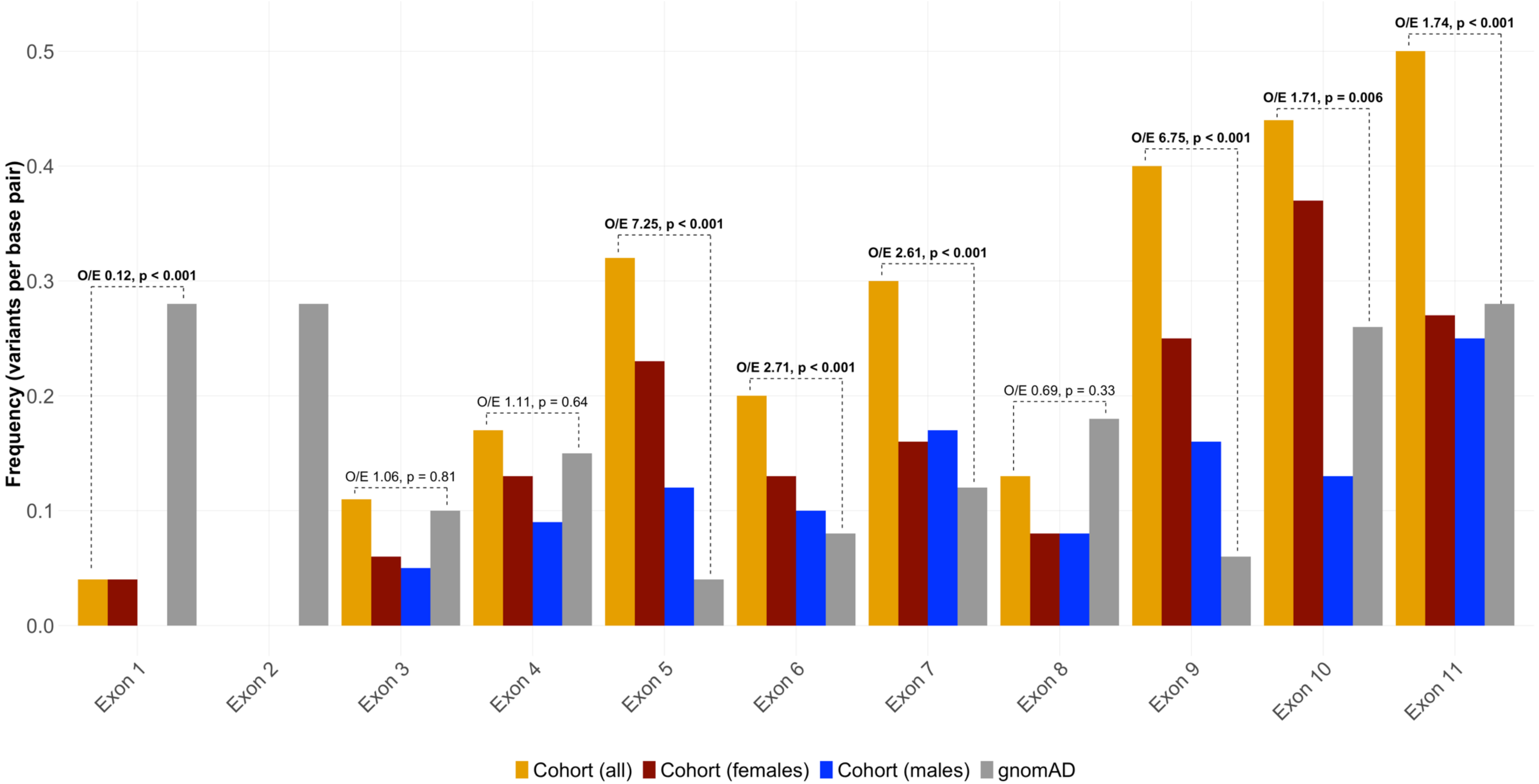

Supplementary figure 4 shows the frequency of coding variants (variants per base pair) for each exon (Exon 1–Exon 11) in the cohort (all, females, and males) compared to the gnomAD database. Observed-to-Expected (O/E) ratios and corresponding p-values of Poisson test are annotated for each exon. The gnomAD dataset was filtered to include only rare variants with an allele frequency < 0.1% and exclude variants classified as “Benign” or “Likely benign” in ClinVar. Additionally, non-coding variants were removed based on VEP annotations, including

UTR variants, intronic or splice variants, and synonymous variants. The variants were assigned to exons based on their genomic positions corresponding to transcript ENST00000422285.7

Supplementary Figure 5. **Age at presentation stratified by gender and variant type**

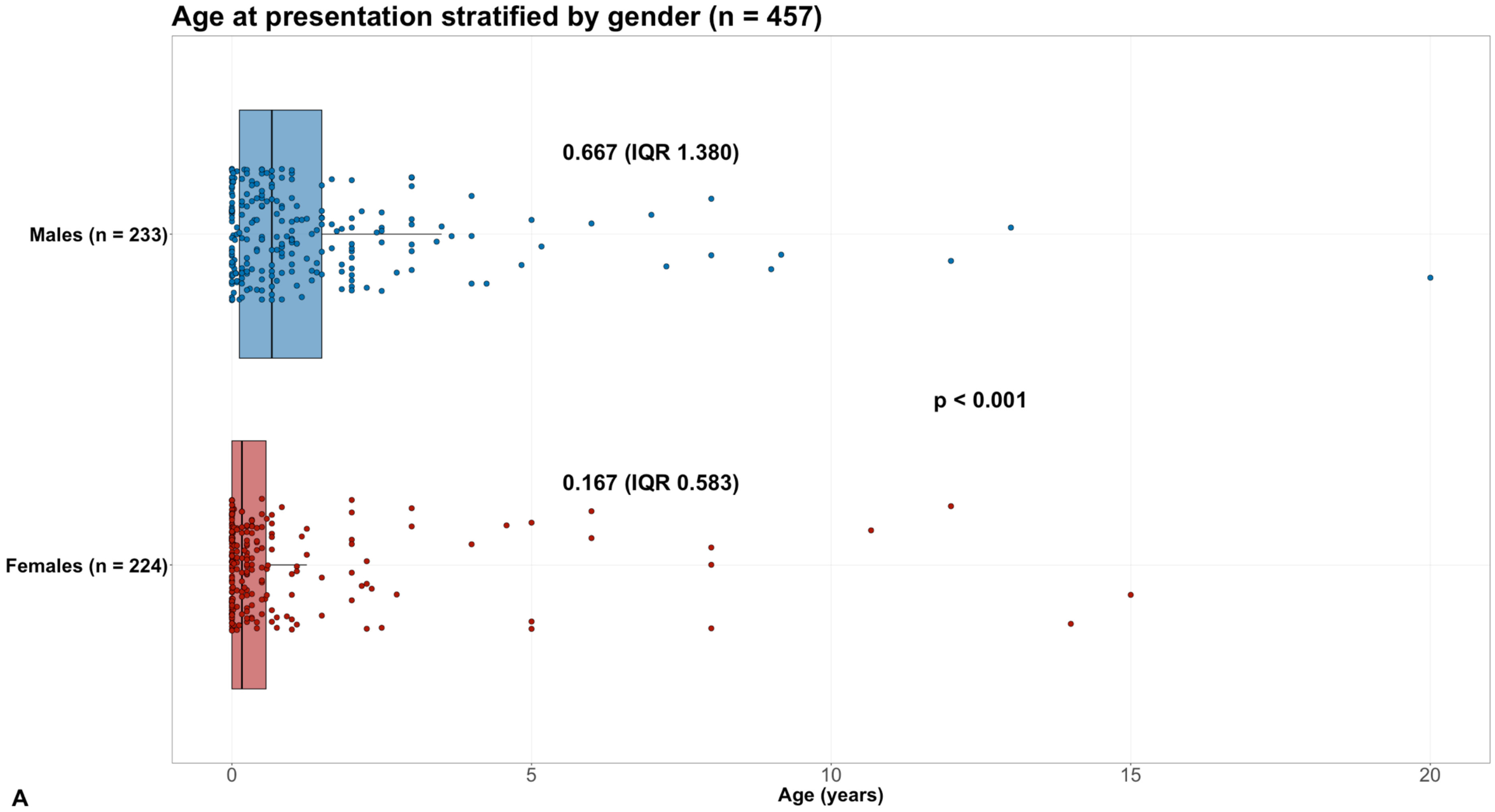

Supplementary Figure 5. **Age at presentation stratified by gender and variant type** *(continued)*

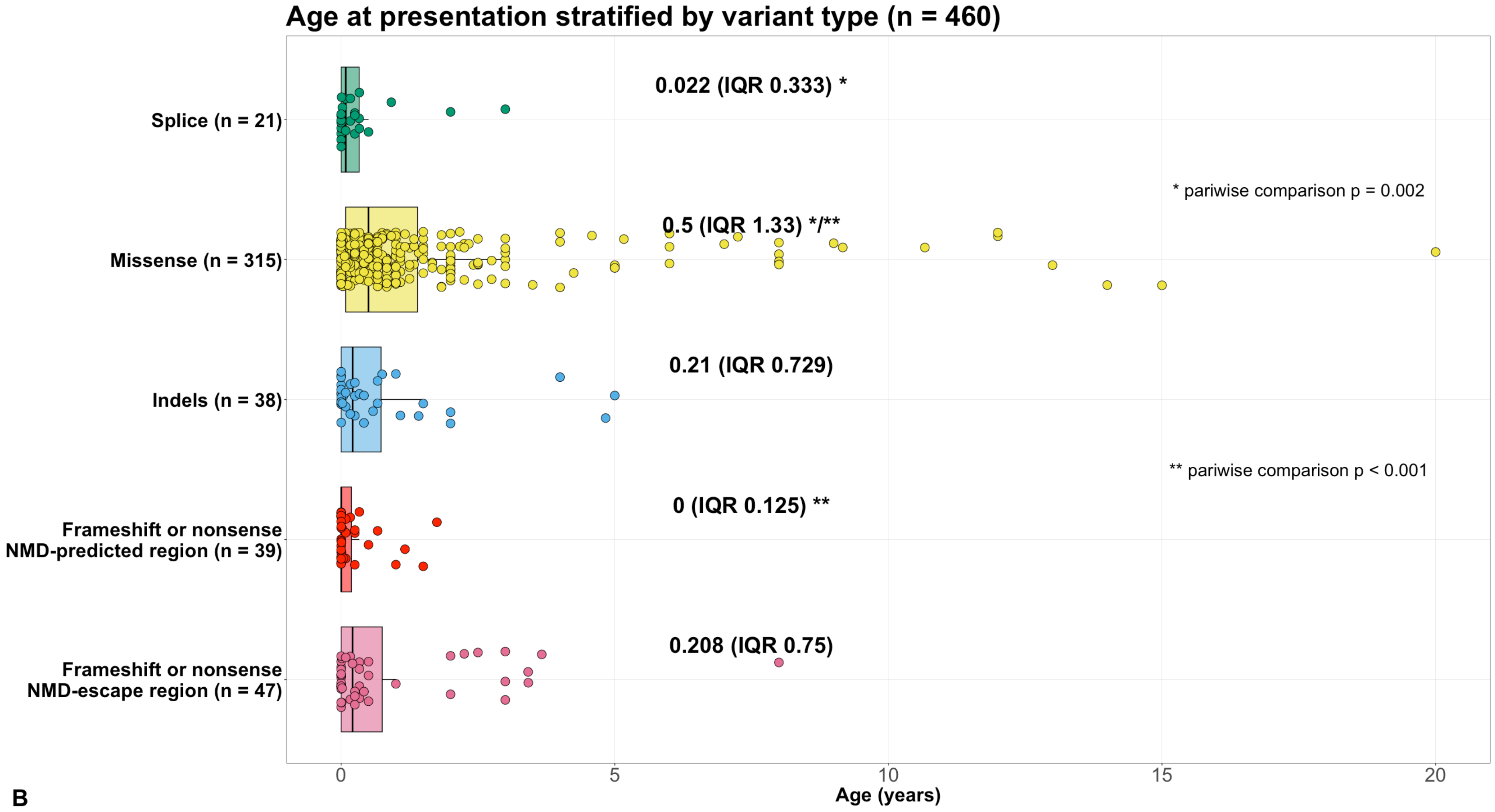

Supplementary Figure 5. **Age at presentation stratified by gender and variant type** *(continued)*

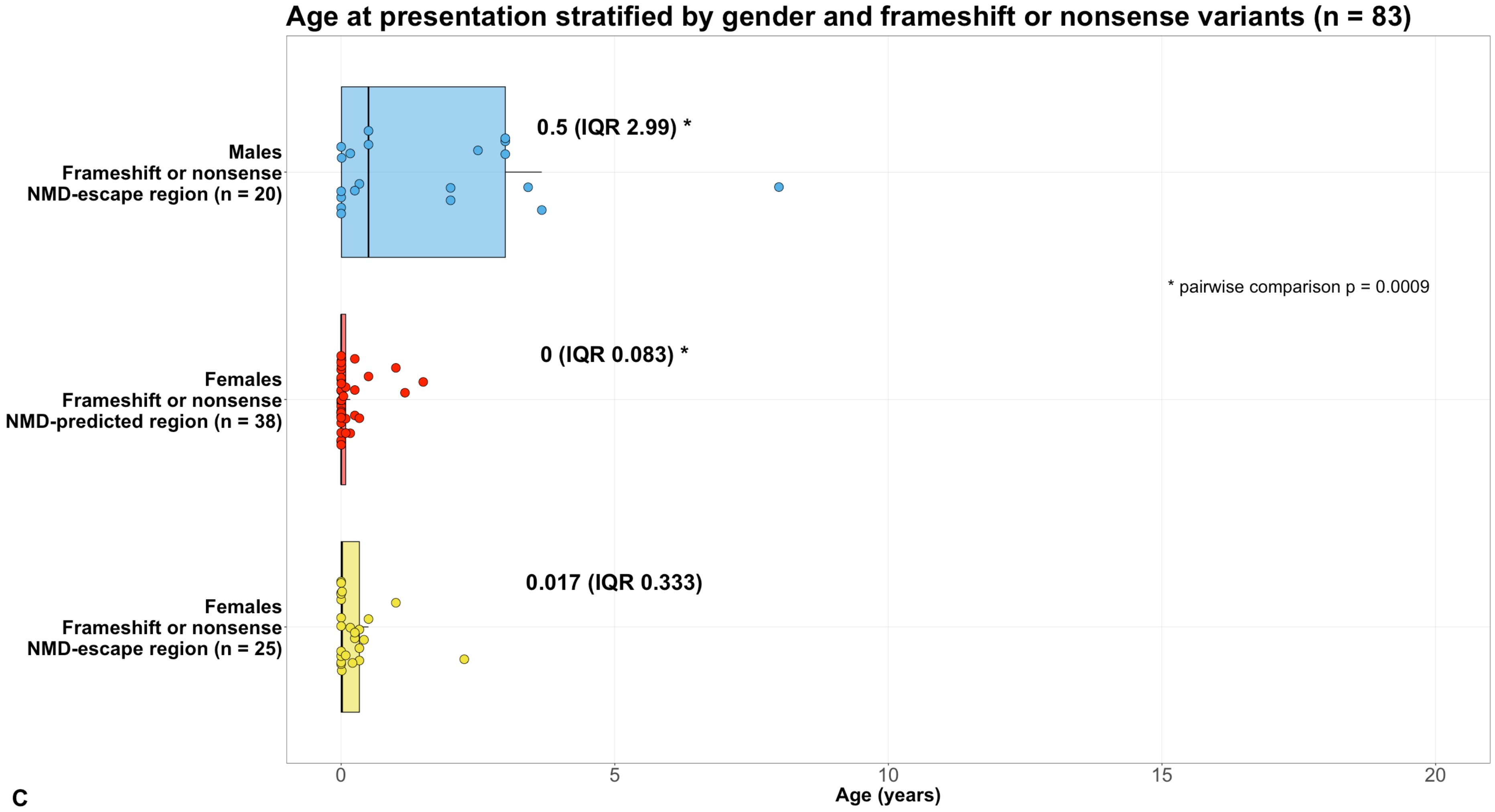

C

Supplementary Figure 5. **Age at presentation stratified by gender and variant type** *(continued)*

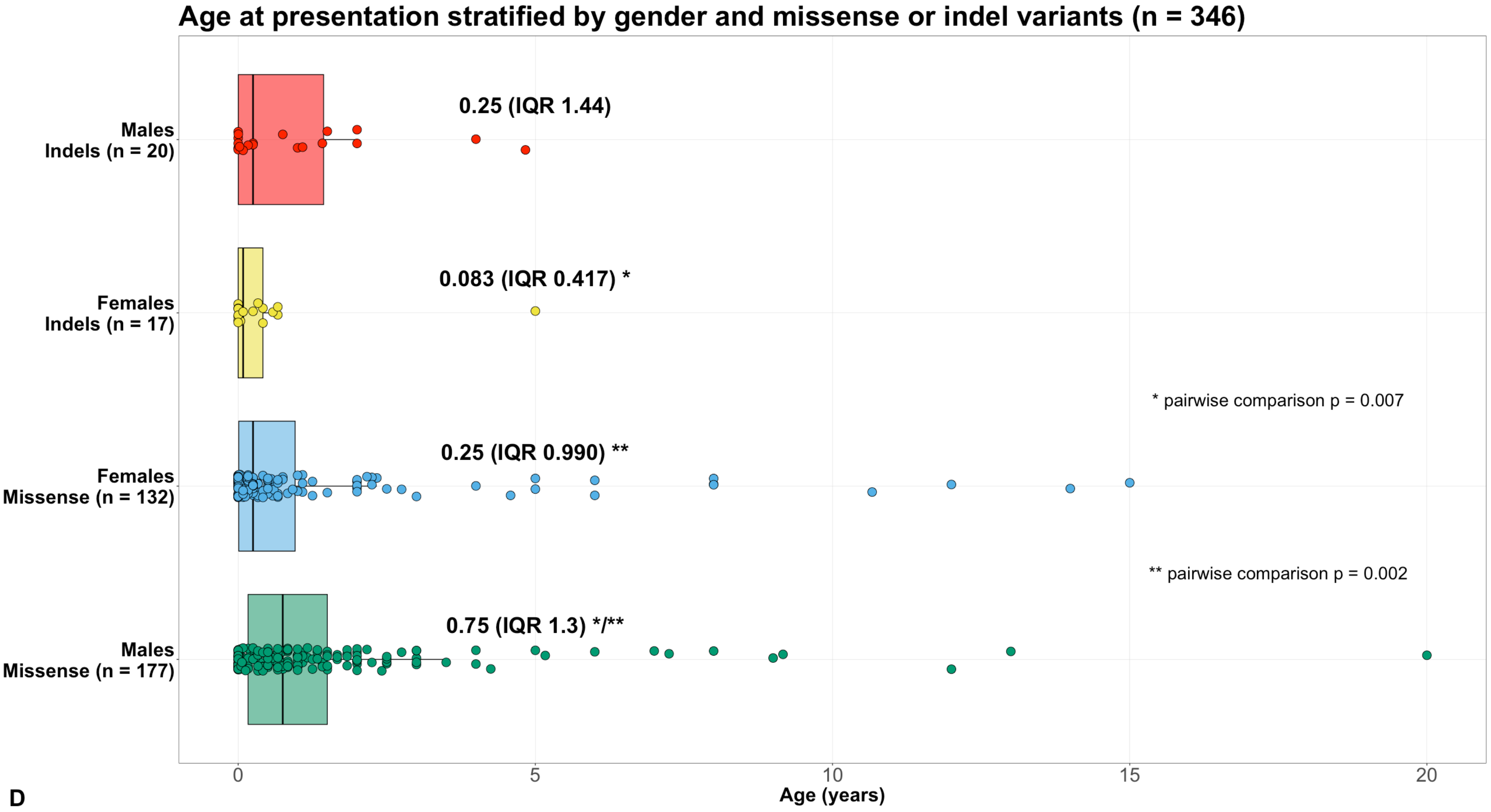

Supplementary Figure 5. **Age at presentation stratified by gender and variant type** *(continued)*

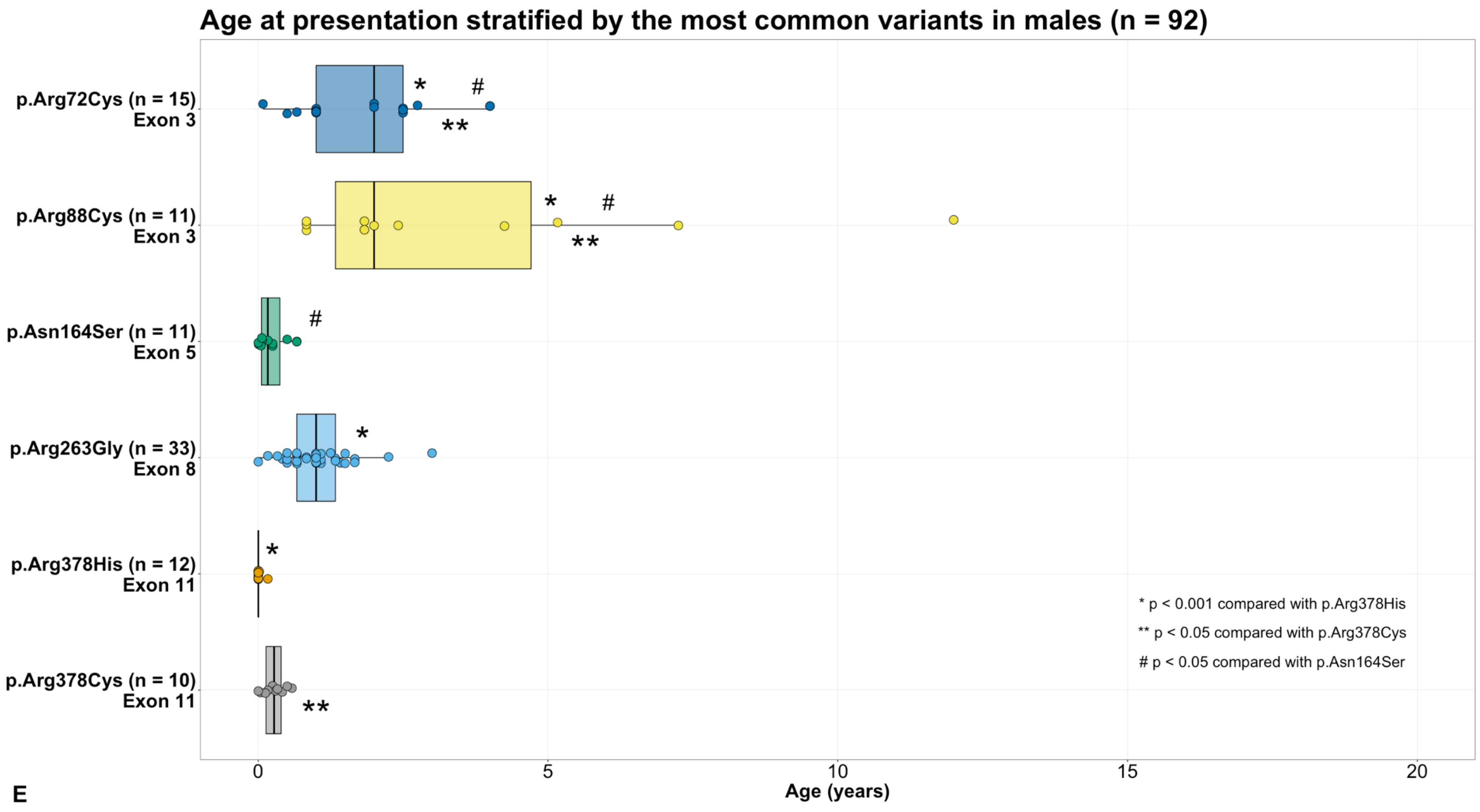

Supplementary Figure 5. **Age at presentation stratified by gender and variant type** *(continued)*

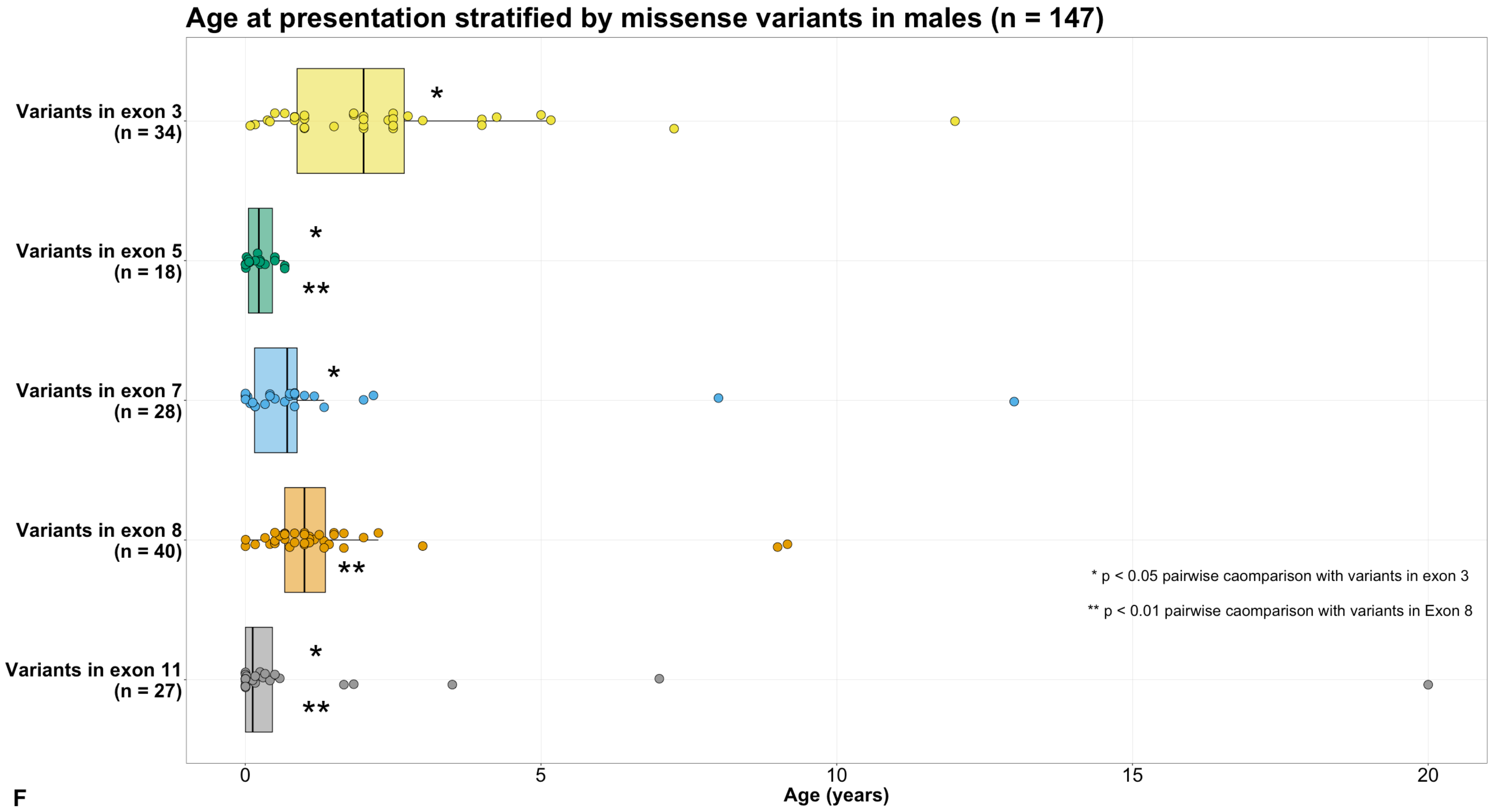

Supplementary Figure 5. **Age at presentation stratified by gender and variant type** *(continued)*

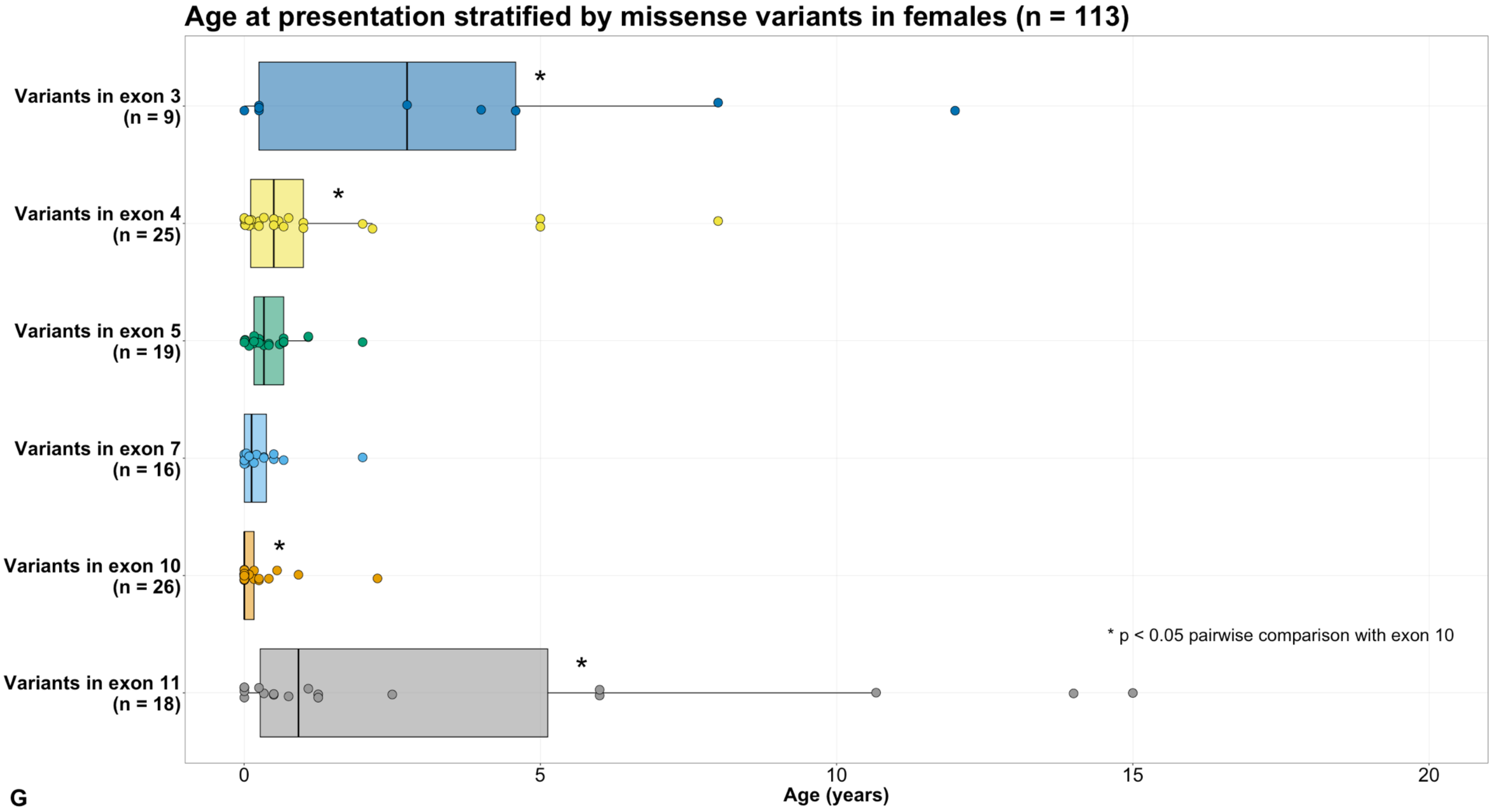

Supplementary figure 5 shows the age at presentation in relation to gender (A), the type of variant (B) and in combination with gender (C-G). Each dot represent a single case. For each subgroup, the median age and interquartile range (IQR) are shown, with only significant p-values displayed.

Dashed lines and neighbouring p-values indicate additional comparisons between subgroups. Regions between p.Met1 to p.Ala34 and p.Leu319 to p.Ser390 were considered as regions predicted to escape nonsense-mediated decay (NMD-escape), the rest as NMD-predicted region. Indels – small in-frame insertions or deletions.

Supplementary Figure 6. **Distribution of age at last report and subset selection for survival analysis**

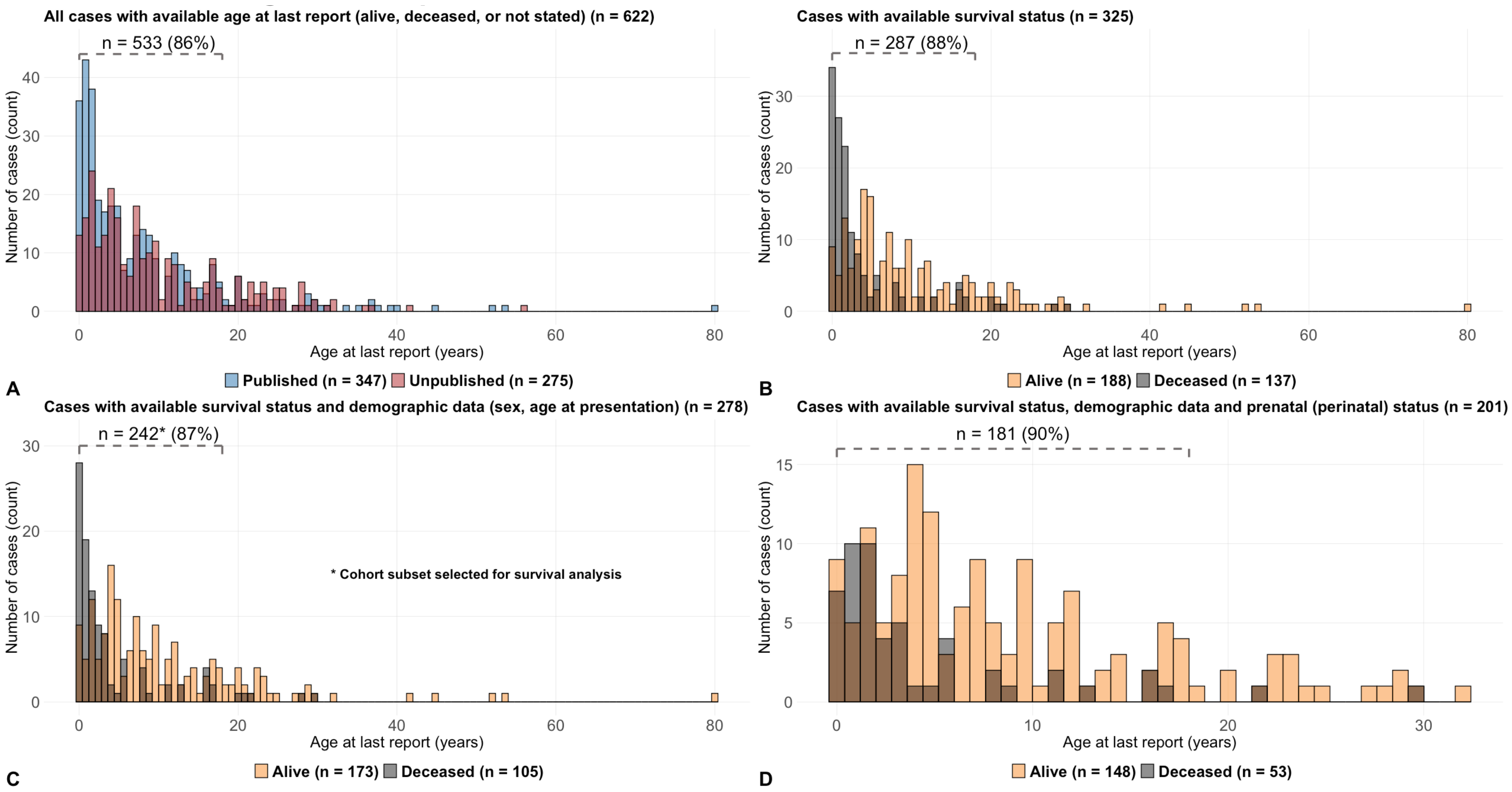

Supplementary figure 6 illustrates the filtering process used to define the cohort for survival analysis. A shows all cases with known age at last report; B restricts to those with known survival status; C applies survival analysis inclusion criteria with known sex, age at presentation; and D

additionally excluded cases without prenatal (perinatal) data. The ≤ 18-year cut-off for final selection was applied to satisfy the 10% rule in Kaplan-Meier analysis across all possible subsets for survival analysis.

Supplementary Figure 7. **Significant predictors of prenatal or perinatal findings**

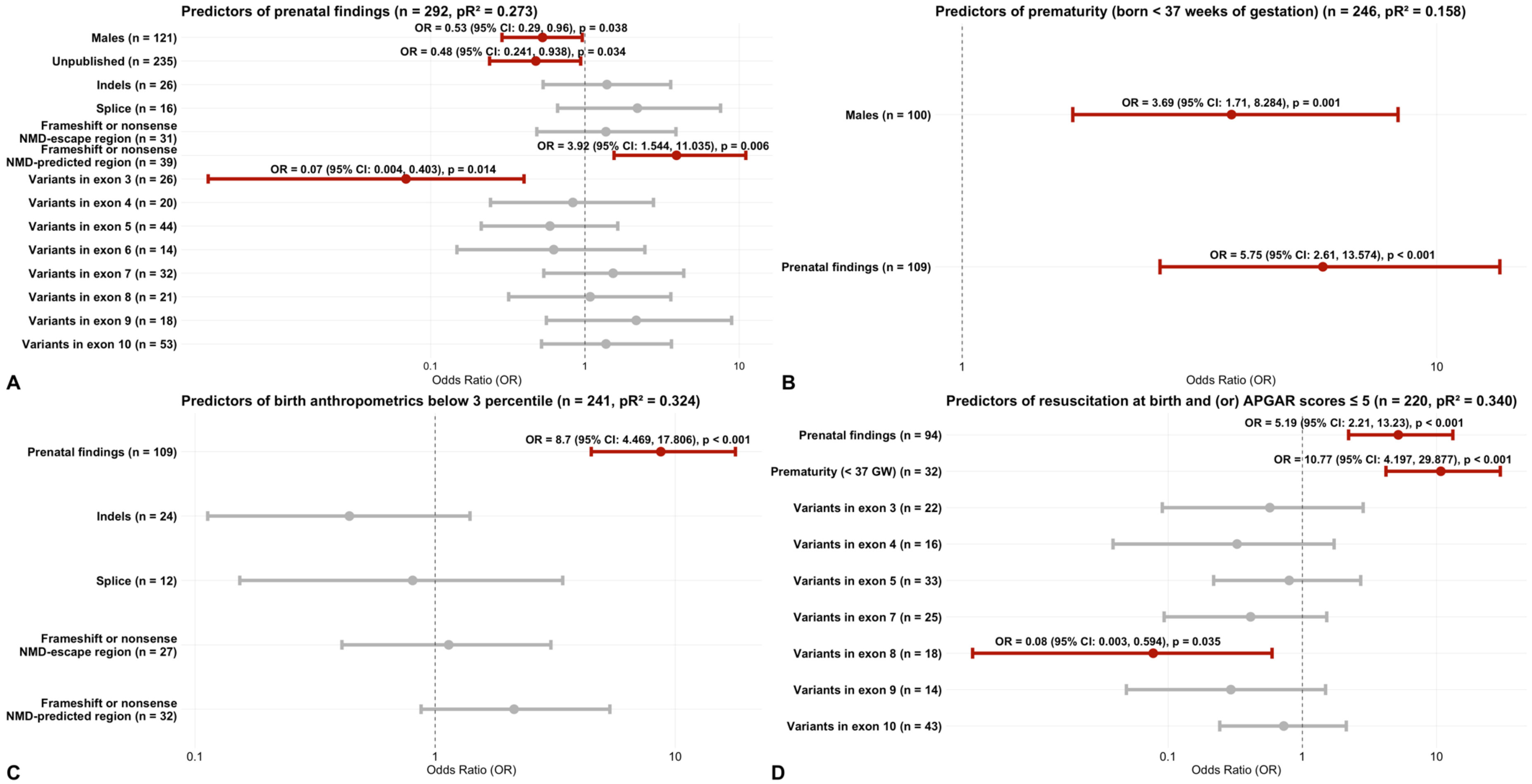

Supplementary figure 7 presents forest plots showing the odds ratios (ORs) from logistic regression analyses of various findings corresponding to supplementary tables 18-21. Significant ORs with their 95% confidence intervals (CIs) are highlighted in red, with corresponding annotations of the OR, CI, and p-value. Non-significant associations are displayed in grey without annotations. Regions between p.Met1 to p.Ala34 and p.Leu319

to p.Ser390 were considered as regions predicted to escape nonsense-mediated decay (NMD-escape), the rest as NMD-predicted region. Dotted line marks OR = 1. Number of cases in a given subanalysis is provided in brackets above the forest plots. pR^2 – Nagelkerke's pseudo R^2.

Supplementary Figure 8. **Significant predictors of neonatal presentation in males and females**

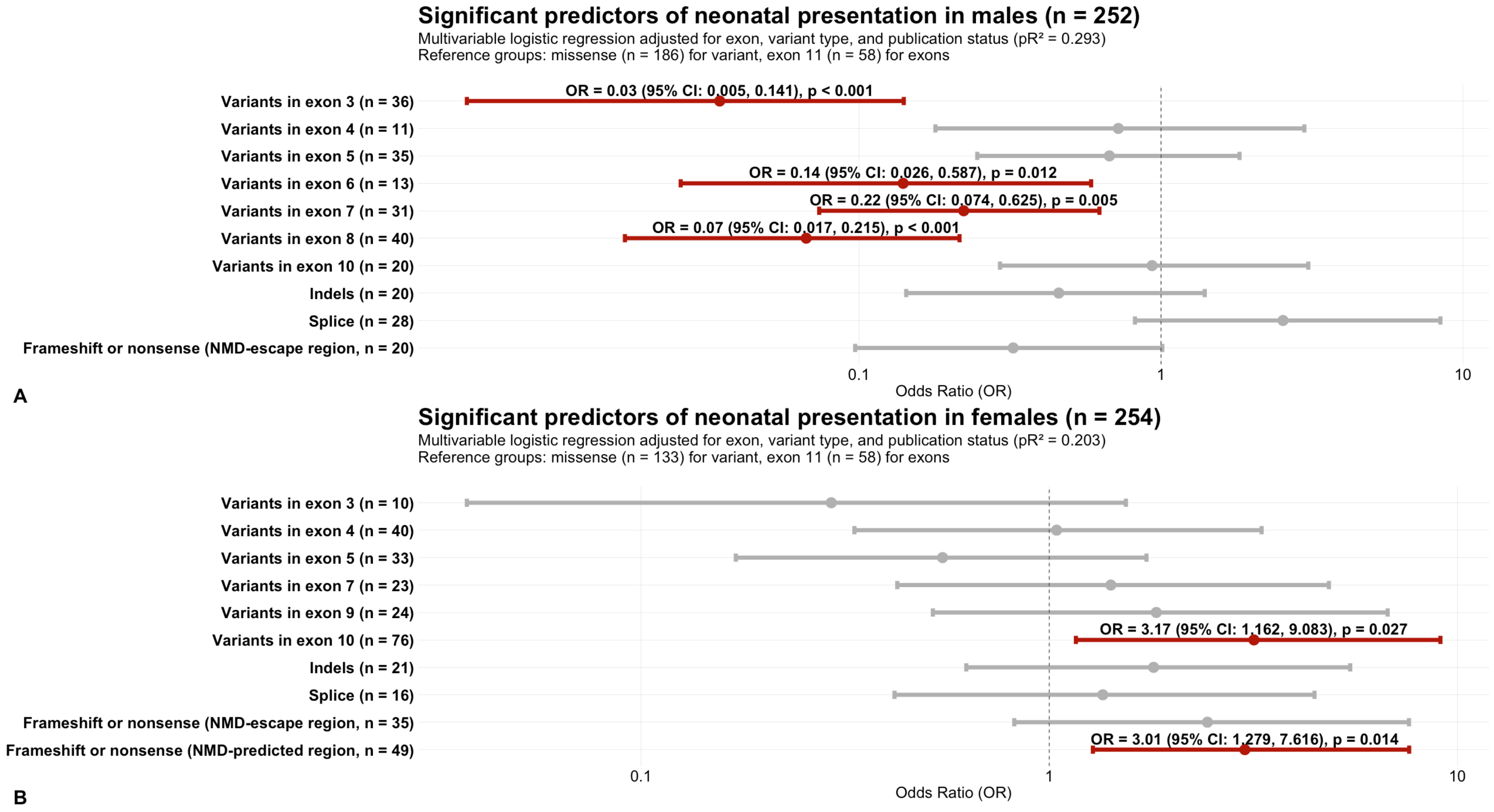

Supplementary figure 8 presents forest plots showing the odds ratios (ORs) from logistic regression analysis of neonatal presentation stratified by sex, corresponding to supplementary tables 22, 23. Significant ORs with their 95% confidence intervals (CIs) are highlighted in red, with

corresponding annotations of the OR, CI, and p-value. Non-significant associations are displayed in grey without annotations. Regions between p.Met1 to p.Ala34 and p.Leu319 to p.Ser390 were considered as regions predicted to escape nonsense-mediated decay (NMD-escape), the rest as NMD-predicted region. Dotted line marks OR = 1. Number of cases in a given subanalysis is provided in brackets above the forest plots. pR^2 – Nagelkerke's pseudo R^2.

Supplementary Figure 9. **Multiple imputation sensitivity survival analysis of known and undefined cases with Kaplan-Meier estimator**

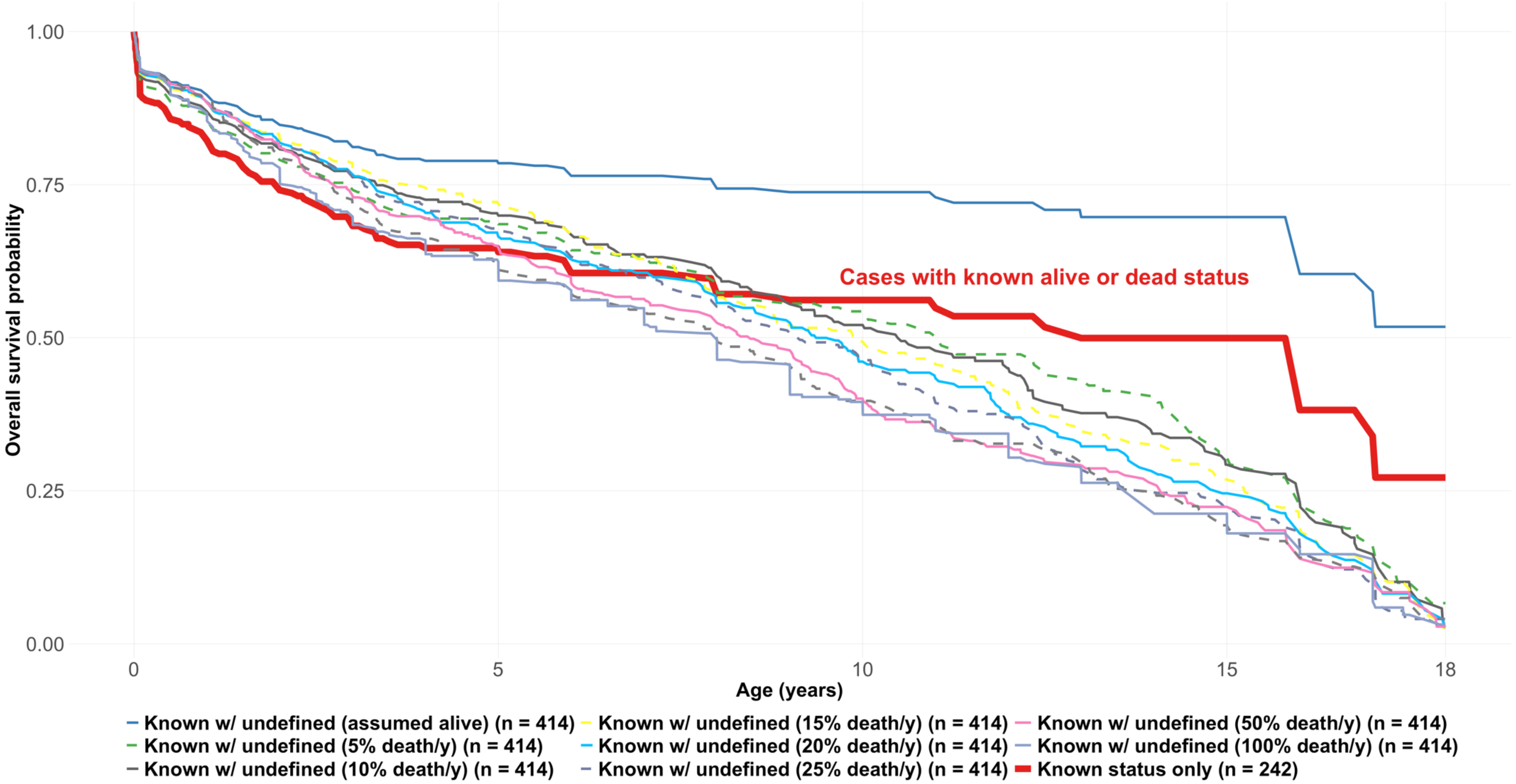

Supplementary figure 9 shows Kaplan-Meier survival curves illustrating the results of a multiple imputation sensitivity analysis for censored cases under varying assumptions. The red curve represents cases with known survival status (either alive or dead) at the last report (n = 242). The blue curve represents cases where unknown outcome cases with available age at last report were assumed to be alive until the end of follow- up and

analysed combined with known cases (n = 414), yielding unrealistic survival. Other curves depict the addition of undefined cases with different annual death probabilities, ranging from 5% (green dashed) to 100% (dark gray), to known cases (n = 242), with high death probabilities drastically reducing survival probabilities. None of the theoretical outcomes of undefined cases matched known cases (red curve), highlighting how survival analysis is fundamentally affected when undefined cases are included with unverified survival data. Survival analysis was confined to 18 years follow-up (supplementary figure 2C). Multiple imputations for undefined cases were performed using a probabilistic simulation approach. For each undefined case, hypothetical survival times were generated in daily intervals, starting from the last known age up to a maximum of 18 years. A predefined daily death probability (0.00014, 0.00029, 0.00045, 0.000611, 0.00079, 0.00189, 0.00379, 1 for an annual probability of 5%, 10%, 15%, 20%, 25%, 50%, 75%, 100%, respectively) was applied, and survival status at each interval was determined using random draws from a binomial distribution. The earliest hypothetical death age was recorded if death occurred; otherwise, cases were retained as censored beyond the follow-up period. This imputed data was combined with known cases, and Kaplan-Meier survival curves were estimated to account for the inclusion of undefined cases under different imputation scenarios. Covariate analysis was not performed.

Supplementary Figure 10. **Significant predictors of developmental delay, intellectual disability, muscle hypotonia, and hypertonia**

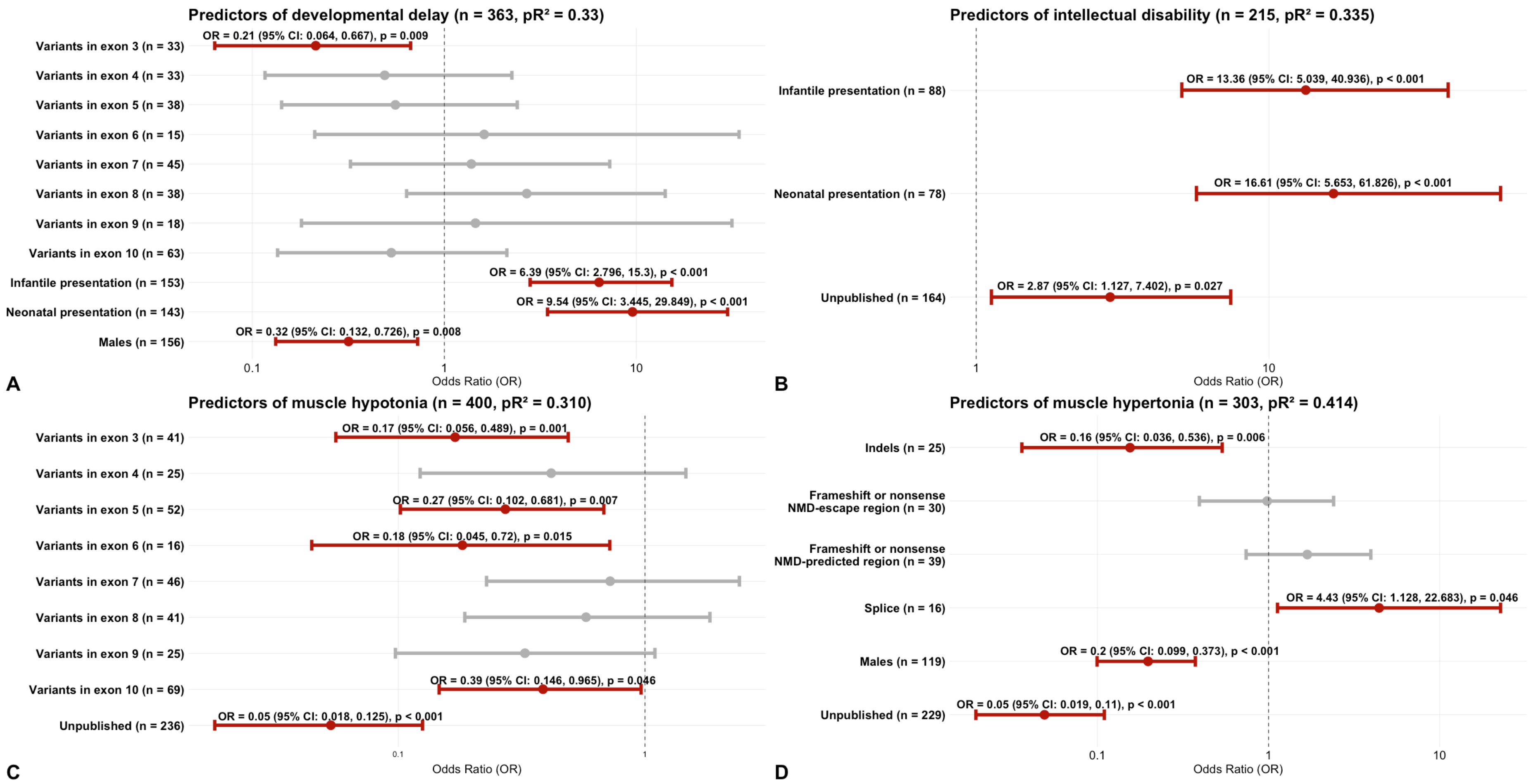

Supplementary figure 10 presents forest plots showing the odds ratios (ORs) from logistic regression analyses of various clinical findings corresponding to supplementary tables 28, 29, 33, 34. Significant ORs with their 95% confidence intervals (CIs) are highlighted in red, with corresponding annotations of the OR, CI, and p-value. Non-significant associations are displayed in grey without annotations. Regions between

p.Met1 to p.Ala34 and p.Leu319 to p.Ser390 were considered as regions predicted to escape nonsense-mediated decay (NMD-escape), the rest as NMD-predicted region. Dotted line marks OR = 1. Number of cases in a given subanalysis is provided in brackets above the forest plots. pR^2 – Nagelkerke's pseudo R^2.

Supplementary Figure 11. **Clinical phenotype homogeneity in cases with the most common variants (at least 10 cases per variant)**

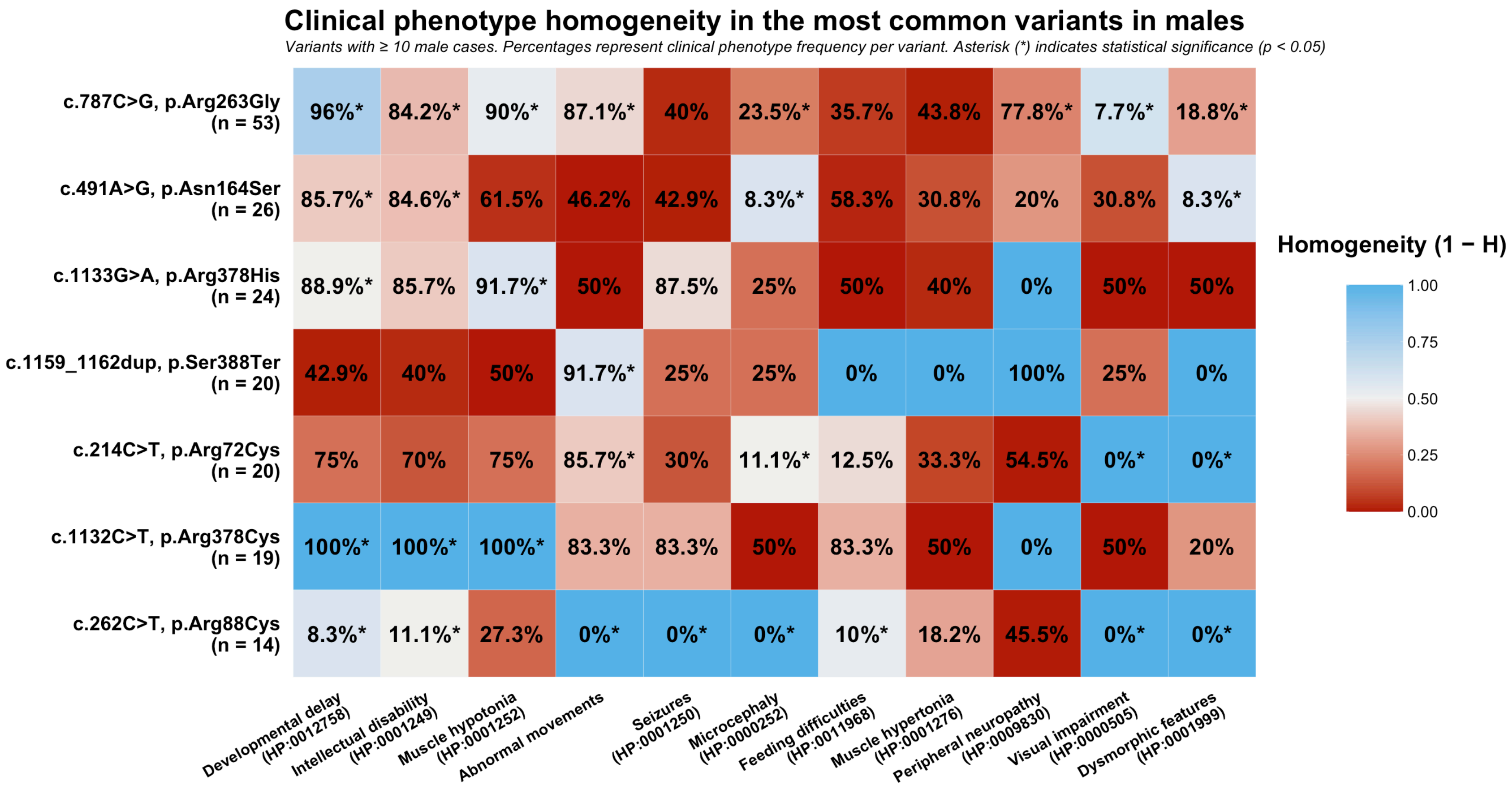

A

Supplementary Figure 11. **Clinical phenotype homogeneity in cases with the most common variants (at least 10 cases per variant)** *(continued)*

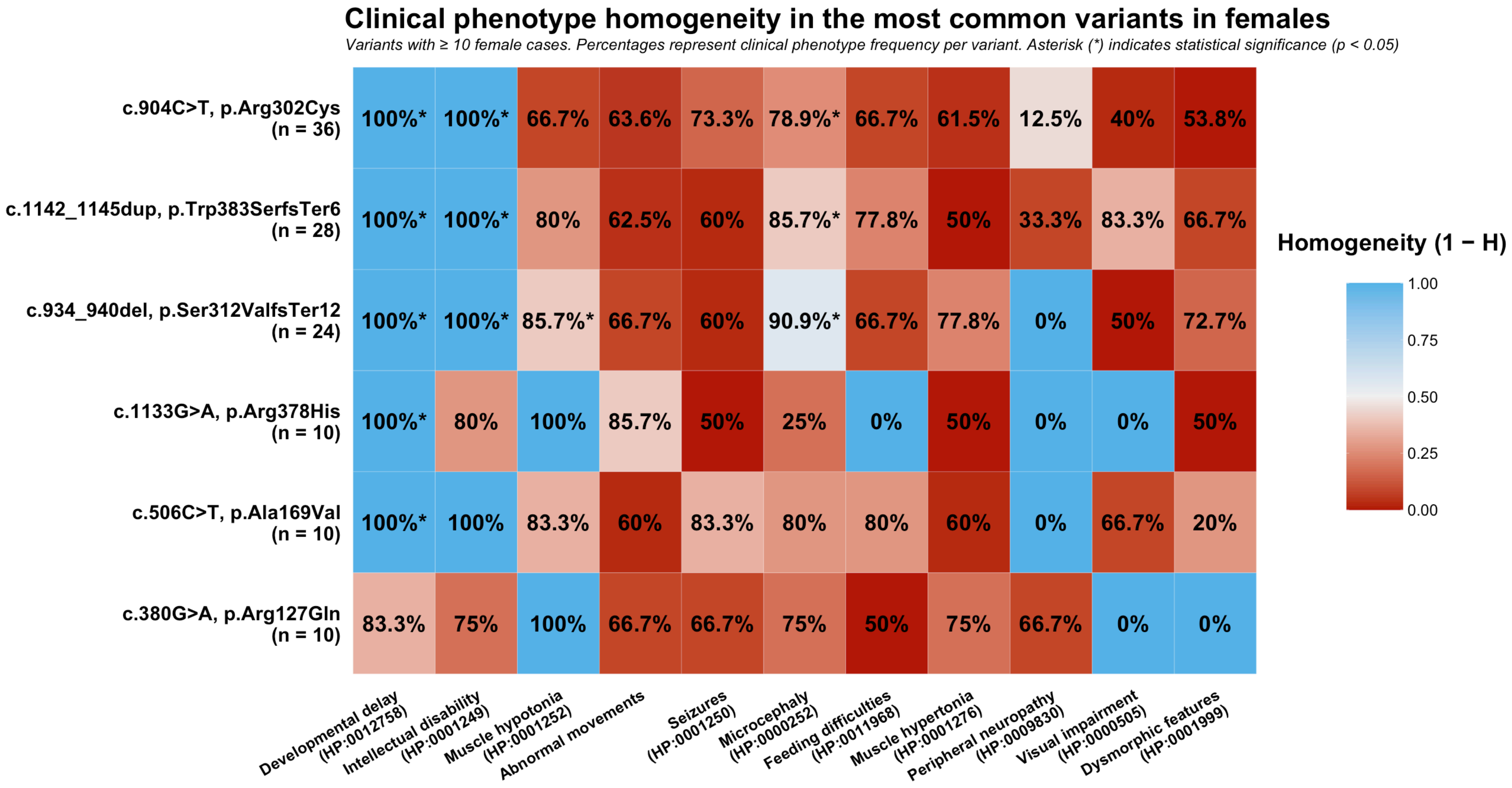

B

The heatmap illustrates clinical phenotype homogeneity across the most common *PDHA1* variants in males (A) and females (B) ($\geq$ 10 cases per variant). Each tile represents the proportion of individuals with a specific clinical feature for a given variant. Homogeneity was measured as 1 − Shannon entropy (H), with darker blue indicating higher clinical uniformity. The percentage indicates how frequently the phenotype occurred within that variant subgroup. Asterisks mark statistically significant ($p < 0.05$) frequency deviation from a theoretical equal distribution (Binomial test). Abnormal movements include these phenotypes: HP:0004305, HP:0100022, HP:0001288, HP:0100660, HP:0001251, HP:0001332.

Supplementary Figure 12. **Significant predictors of microcephaly, seizures, feeding difficulties, and dysmorphic features**

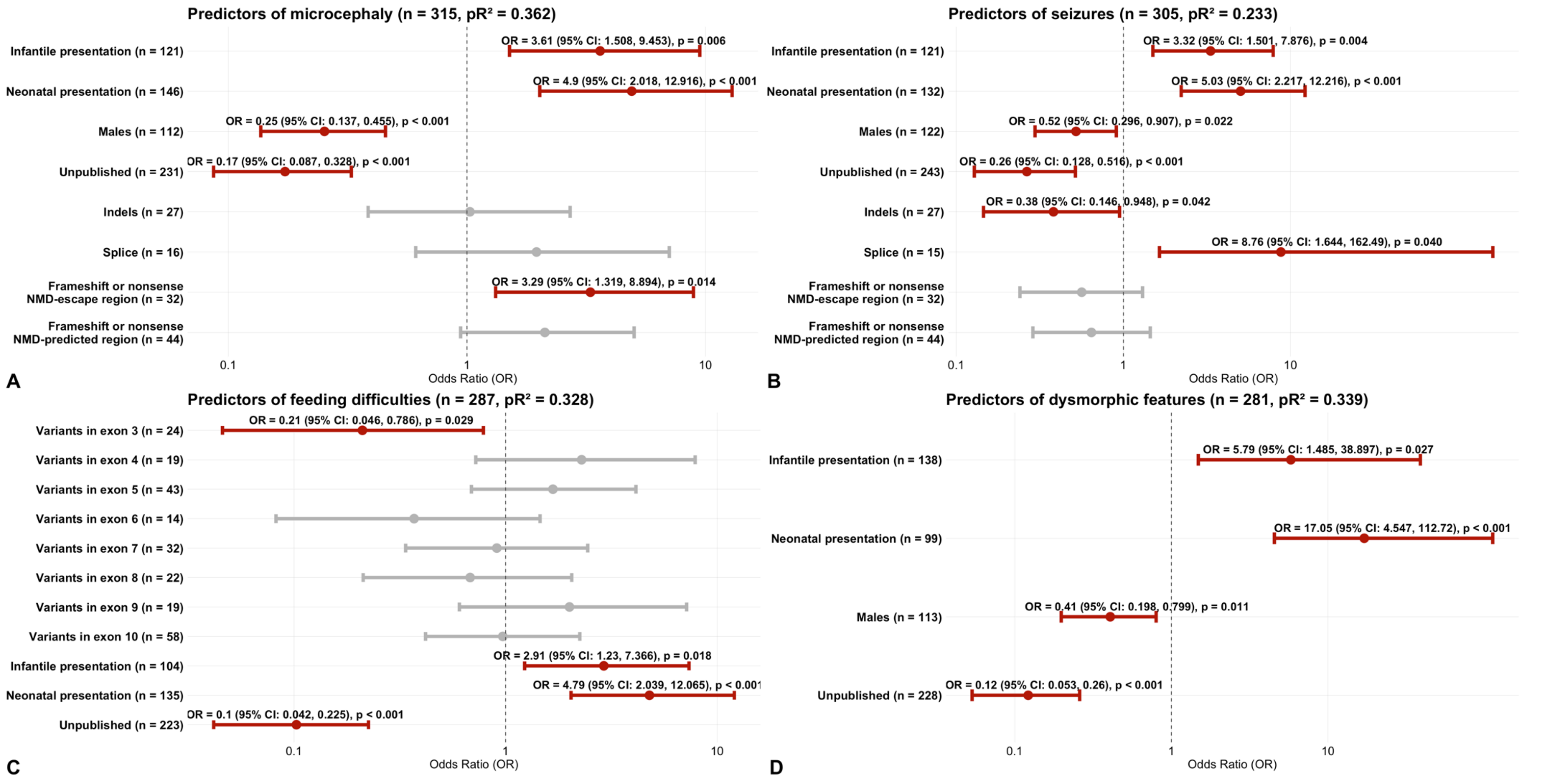

Supplementary figure 12 presents forest plots showing the odds ratios (ORs) from logistic regression analyses of various clinical findings corresponding to supplementary tables 35-38. Significant ORs with their 95% confidence intervals (CIs) are highlighted in red, with corresponding annotations of the OR, CI, and p-value. Non-significant associations are displayed in grey without annotations. Regions between p.Met1 to p.Ala34

and p.Leu319 to p.Ser390 were considered as regions predicted to escape nonsense-mediated decay (NMD-escape), the rest as NMD-predicted region. Dotted line marks OR = 1. Number of cases in a given subanalysis is provided in brackets above the forest plots. pR^2 – Nagelkerke's pseudo R^2.

Supplementary Figure 13. **Significant predictors of abnormal movements, peripheral neuropathy, visual impairment, and abnormal skeletal morphology**

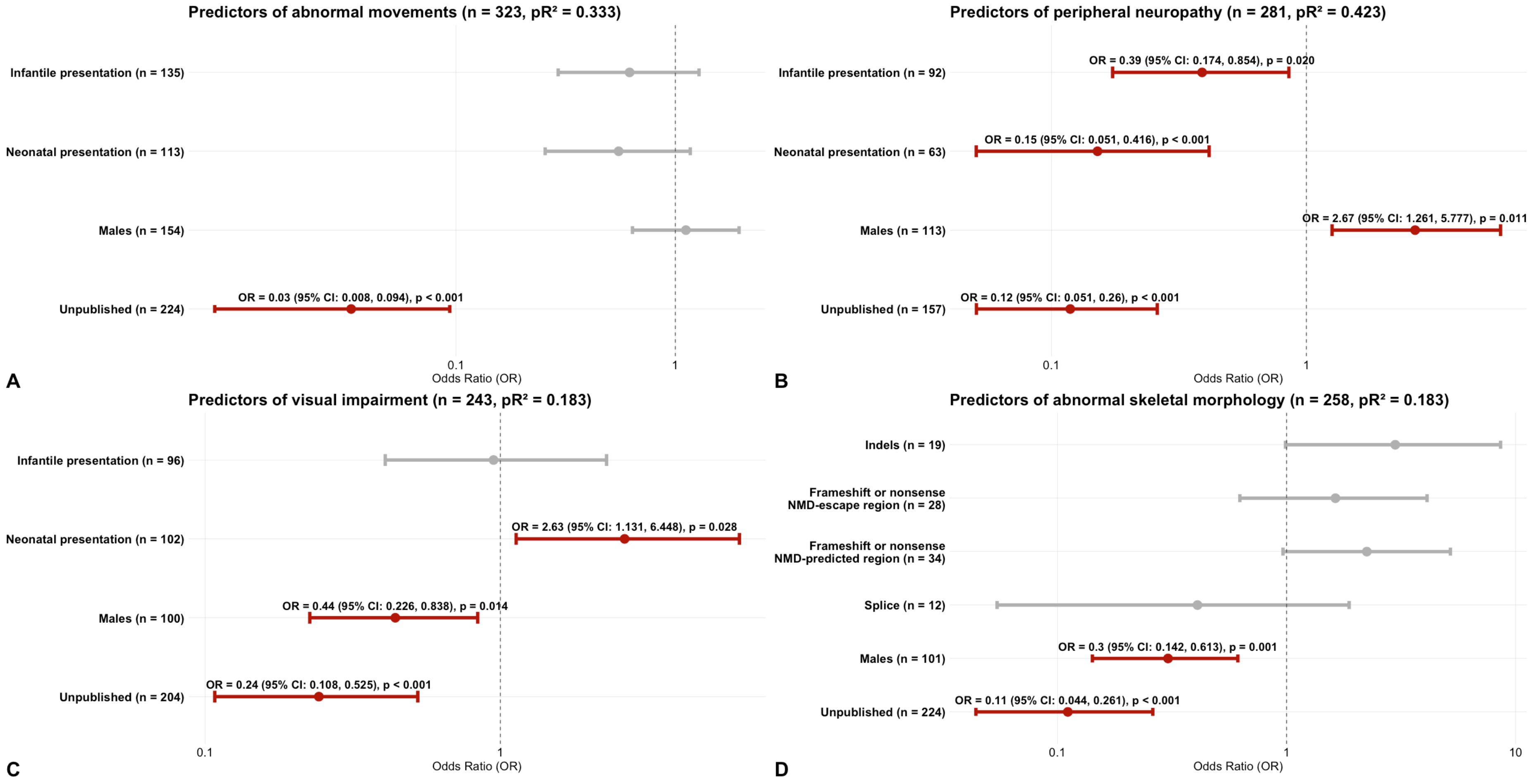

Supplementary figure 13 presents forest plots showing the odds ratios (ORs) from logistic regression analyses of various clinical findings corresponding to supplementary tables 39-41, 43. Significant ORs with their 95% confidence intervals (CIs) are highlighted in red, with

corresponding annotations of the OR, CI, and p-value. Non-significant associations are displayed in grey without annotations. Regions between p.Met1 to p.Ala34 and p.Leu319 to p.Ser390 were considered as regions predicted to escape nonsense-mediated decay (NMD-escape), the rest as NMD-predicted region. Dotted line marks OR = 1. Number of cases in a given subanalysis is provided in brackets above the forest plots. pR^2 – Nagelkerke's pseudo R^2. Abnormal movements include these phenotypes: HP:0004305, HP:0100022, HP:0001288, HP:0100660, HP:0001251,HP:0001332.

Supplementary Figure 14. **Significant predictors of cerebral atrophy, basal ganglia findings, corpus callosum malformations, and ventriculomegaly or hydrocephalus**

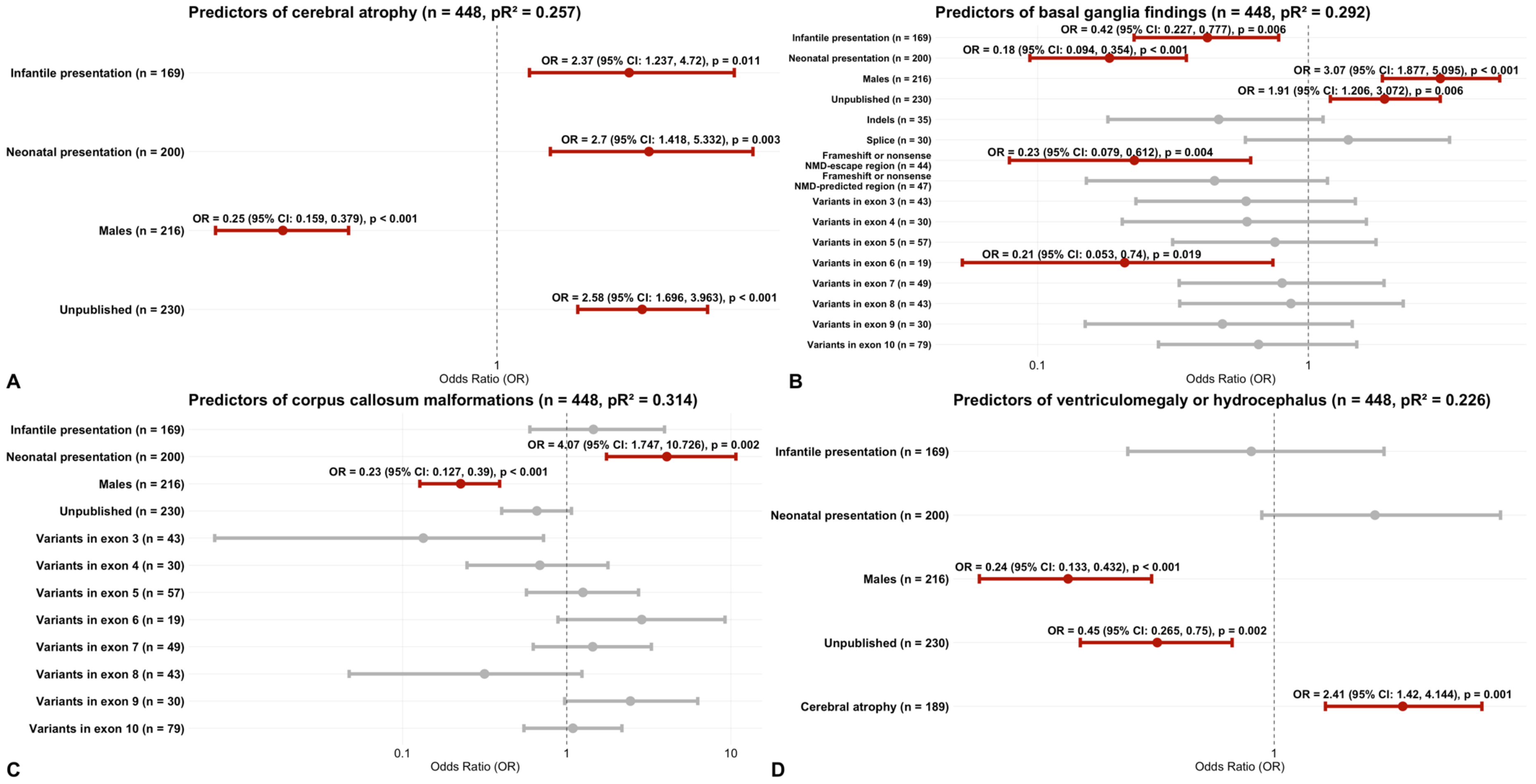

Supplementary figure 14 presents forest plots showing the odds ratios (ORs) from logistic regression analyses of various CNS findings corresponding to supplementary tables 48-51. Significant ORs with their 95% confidence intervals (CIs) are highlighted in red, with corresponding

annotations of the OR, CI, and p-value. Non-significant associations are displayed in grey without annotations. Regions between p.Met1 to p.Ala34 and p.Leu319 to p.Ser390 were considered as regions predicted to escape nonsense-mediated decay (NMD-escape), the rest as NMD-predicted region. Dotted line marks OR = 1. Number of cases in a given subanalysis is provided in brackets above the forest plots. pR^2 – Nagelkerke's pseudo R^2.

Supplementary Figure 15. **Summary of the most common laboratory findings**

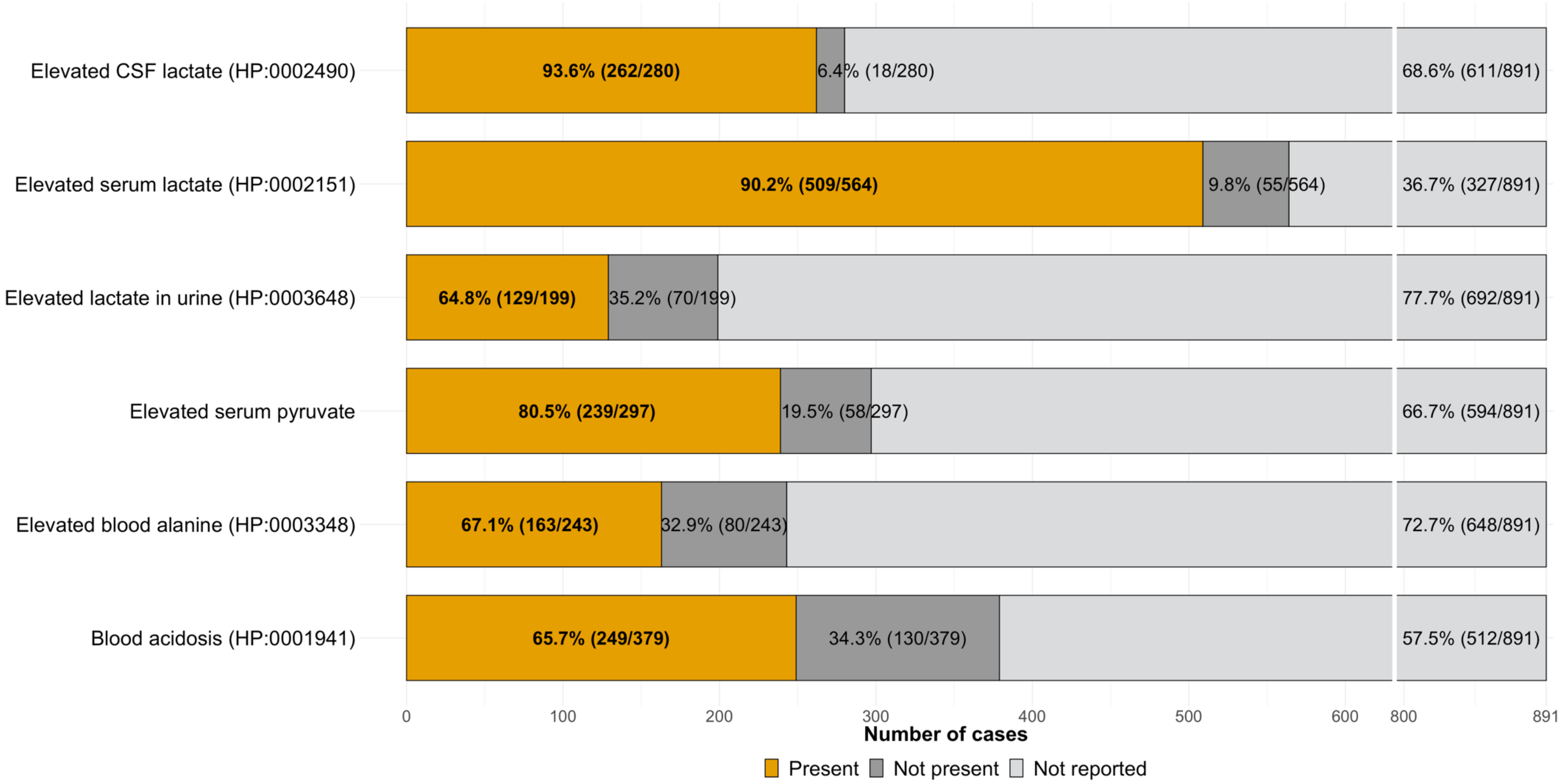

Supplementary figure 15 shows the most common laboratory findings among all cases; where applicable, HPO (Human Phenotype Ontology) codes are provided next to each finding. Findings are listed in decreasing frequency from the top.

Supplementary Figure 16. **Enzyme activity in fibroblasts stratified by sex, variant type, affected exon and age at presentation**

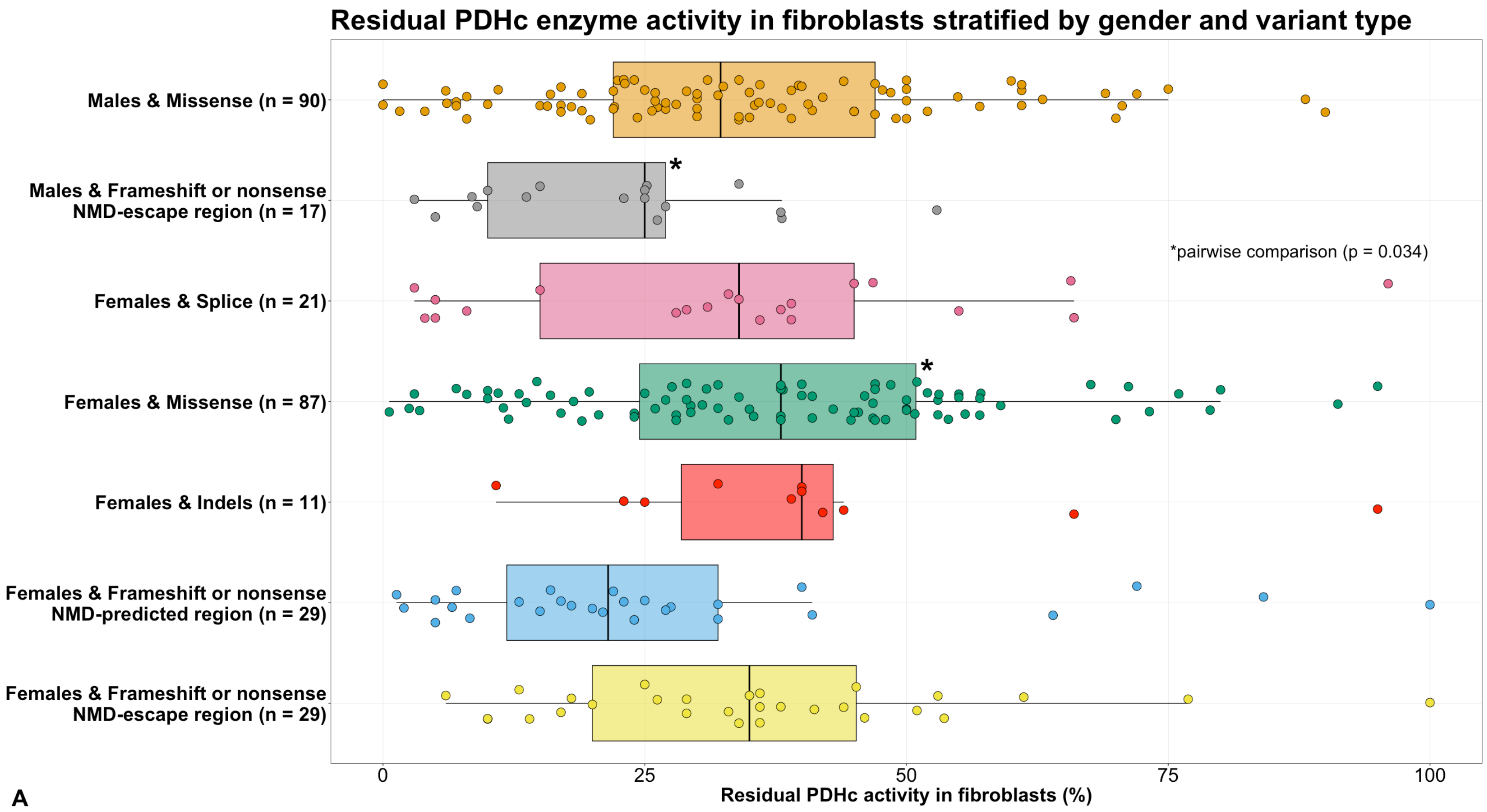

Supplementary Figure 16. **Enzyme activity in fibroblasts stratified by sex, variant type, affected exon and age at presentation** *(continued)*

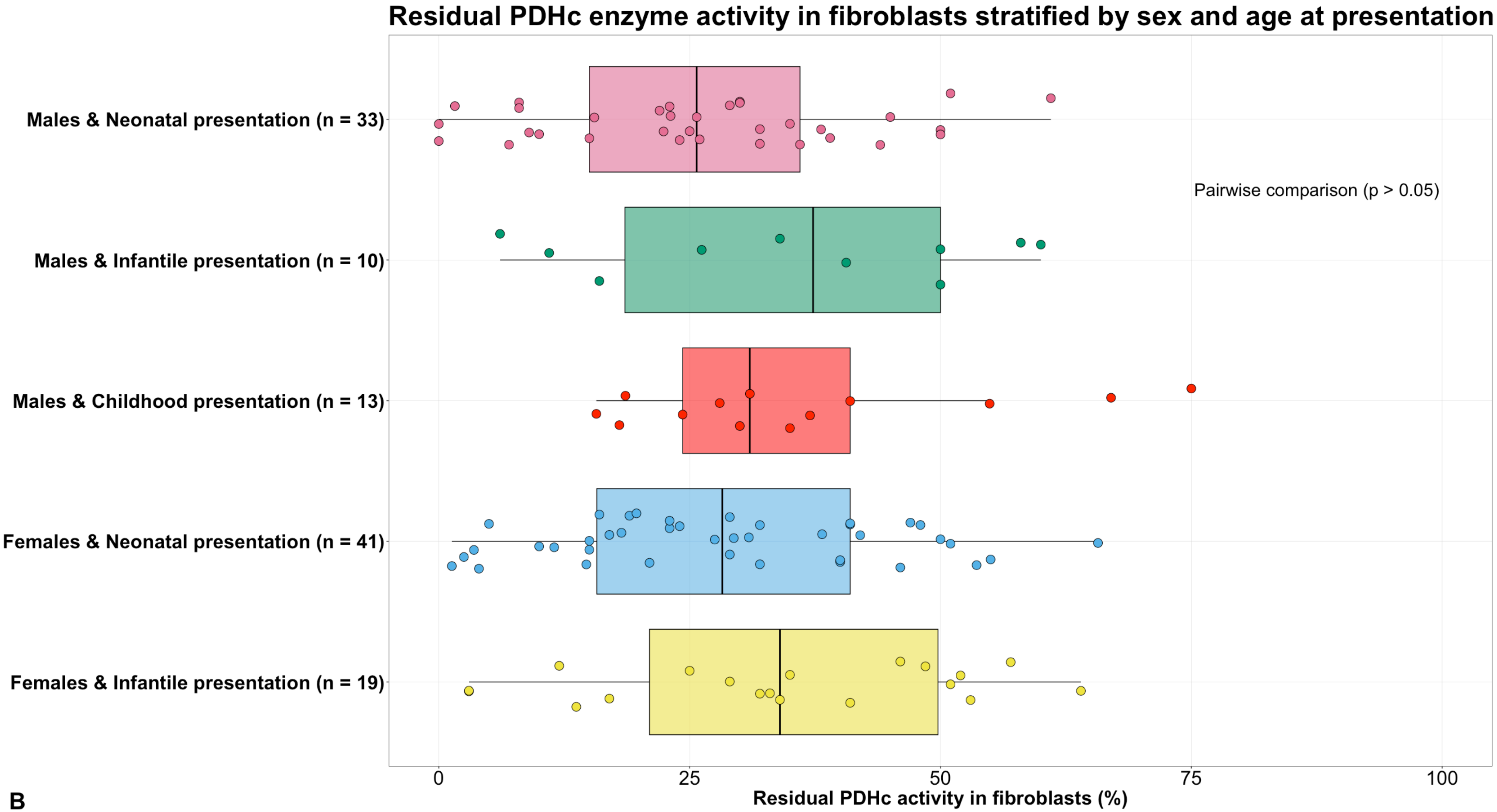

B

Supplementary Figure 16. **Enzyme activity in fibroblasts stratified by sex, variant type, affected exon and age at presentation** *(continued)*

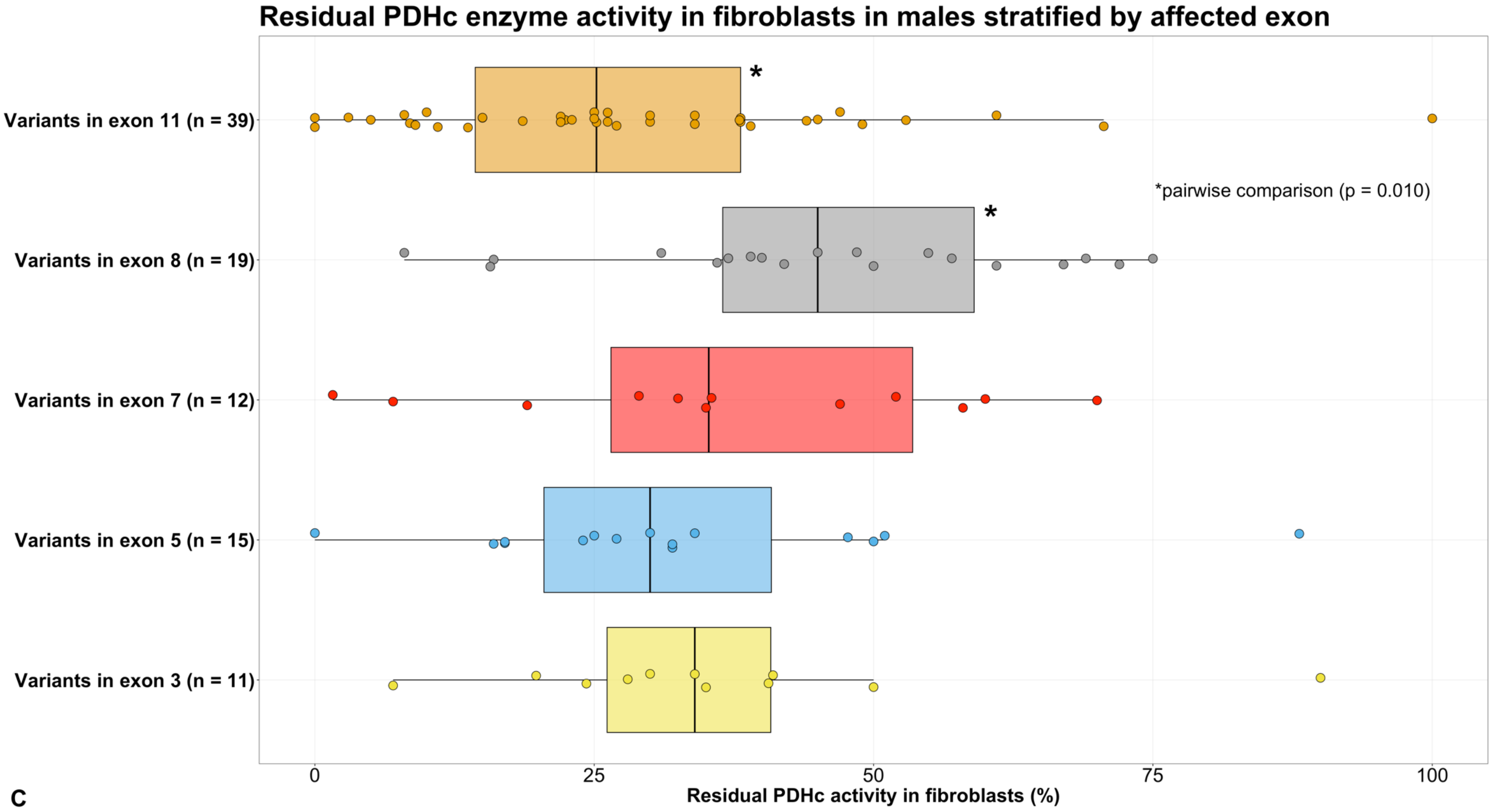

Supplementary Figure 16. **Enzyme activity in fibroblasts stratified by sex, variant type, affected exon and age at presentation** *(continued)*

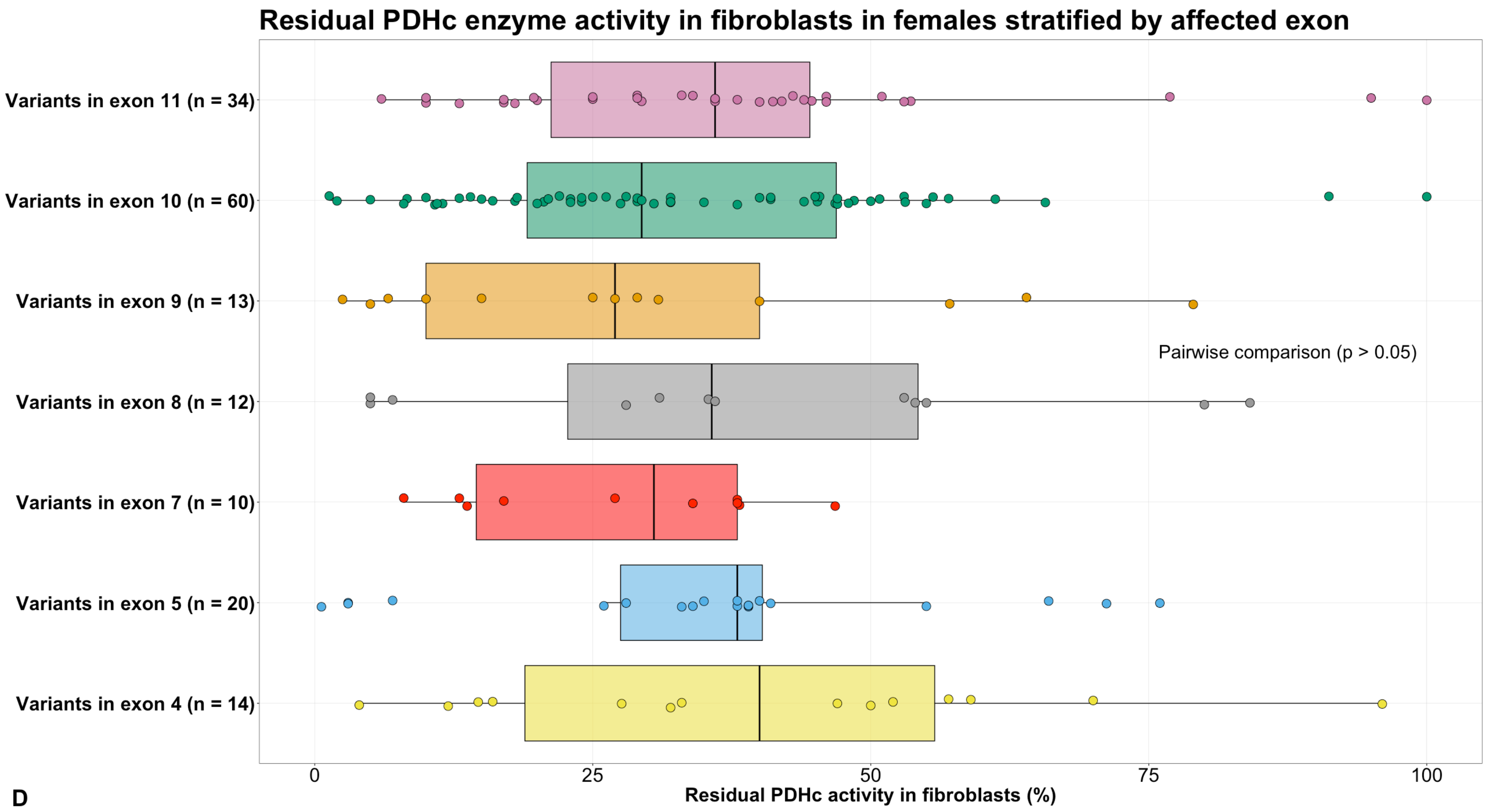

In supplementary figure 16 residual PDHc enzyme activity (range 0-100%) in fibroblasts is shown for: (A) males and females grouped by variant; (B) males and females grouped by age at first presentation presentation (neonatal [0-28 days], infantile [29 days – 12 months], or childhood [1-13

years]); (C) in males grouped by affected exon; (D) in females grouped by affected exon. Only significant comparisons are shown. Regions between p.Met1 to p.Ala34 and p.Leu319 to p.Ser390 were considered as regions predicted to escape nonsense-mediated decay (NMD-escape), the rest as NMD-predicted region. Indels – small in-frame insertions or deletions.

Supplementary Tables

Supplementary Table 1. **Critical appraisal of case reports selected for case inclusion**

Case report (reference)	Case ID	Q1	Q2	Q3	Q4	Q5	Q6	Q7	Q8
Endo H et al., 1989[1]	Pt#21	Yes	No	Yes	Yes	Yes	No	No	Yes
Dahl H et al., 1990[2]	Pt#20	Yes	Yes	Yes	Yes	Unclear	Yes	No	Yes
Chun K et al., 1991[3]	Pt#19	Yes	Yes	Yes	Yes	NA	NA	NA	Yes
De Meirleir L et al., 1991[4]	Pt#194	Yes	Yes	Yes	Yes	NA	NA	NA	Yes
Endo H et al., 1991[5]	Pt#227	Yes	Yes	Yes	Yes	No	NA	NA	Yes
Ito M et al., 1992[6]	Pt#226	Yes	Yes	No	Yes	No	NA	NA	Yes
De Meirleir L et al., 1992[7]	Pt#1023	Yes	Yes	Yes	Yes	NA	NA	NA	Yes
Matthews P et al., 1993[8]	Pt#159	Yes	Yes	Yes	Yes	NA	NA	NA	Yes
Hansen L et al., 1993[9]	Pt#160	Yes	No	Yes	Yes	NA	NA	NA	Yes
Takakubo F et al., 1993[10]	Pt#167	Yes	No	Yes	Yes	No	NA	NA	Yes
Takakubo F et al., 1993[11]	Pt#168	Yes	Yes	Yes	Yes	NA	NA	NA	Yes
De Meirleir L et al., 1993[12]	Pt#217	Yes	Yes	Yes	Yes	No	NA	NA	Yes
Naito E et al., 1994[13]	Pt#1	Yes	Yes	Yes	Yes	Yes	Yes	No	Yes
Naito E et al., 1994[14]	Pt#40	Yes	Yes	Yes	Yes	NA	NA	NA	Yes
Hansen L et al., 1994[15]	Pt#223	Yes	Yes	Yes	Yes	Yes	Yes	No	Yes
Dahl H et al., 1994[16]	Pt#416	Yes	Unclear	Yes	Yes	NA	NA	NA	Yes
Otero L et al., 1995[17]	Pt#41	Yes	Yes	Yes	Yes	No	NA	NA	Yes
Hemalatha S et al., 1995[18]	Pt#181	Yes	No	Yes	Yes	NA	No	No	Yes
Takakubo F et al., 1995[19]	Pt#216	Yes	Unclear	Yes	Unclear	Unclear	Yes	No	Yes
Lissens W et al., 1995[20]	Pt#224	Yes	Unclear	Yes	Yes	NA	NA	NA	Yes
Ito M et al., 1995[21]	Pt#225	Yes	Unclear	Yes	Yes	Unclear	NA	NA	Yes
Naito E et al., 1997[22]	Pt#53	Yes	Yes	Yes	Yes	Yes	Yes	No	Yes

Supplementary table 1. **Critical appraisal of case reports selected for case inclusion** *(continued)*

Case report (reference)	Case ID	Q1	Q2	Q3	Q4	Q5	Q6	Q7	Q8
Takahashi S et al., 1997[23]	Pt#228	Yes	Yes	Yes	Yes	Yes	Yes	No	Yes
Rubio-Gozalbo M et al., 1999[24]	Pt#2	Yes	Yes	Yes	Yes	Yes	Yes	No	Yes
Naito E et al., 1999[25]	Pt#229	Yes	Yes	Yes	Yes	Yes	Yes	No	Yes
Seyda A et al., 2001[26]	Pt#450	Yes	No	Yes	Yes	NA	NA	NA	Yes
Benelli C et al., 2002[27]	Pt#464	Yes	Unclear	Yes	Yes	Yes	Yes	No	Yes
Mine M et al., 2003[28]	Pt#3	Yes	No	Yes	Yes	Yes	No	No	Yes
Brown R et al., 2003[29]	Pt#404	Yes	Unclear	Yes	Yes	NA	NA	NA	Yes
Wada N et al., 2004[30]	Pt#529	Yes	Yes	Yes	Yes	No	Yes	No	Yes
Silva MJ et al., 2004[31]	Pt#533	Yes	Yes	Yes	Yes	NA	NA	NA	Yes
Brivet M et al., 2005[32]	Pt#528	Yes	Yes	Yes	Yes	NA	NA	NA	Yes
Lee E et al., 2006[33]	Pt#380	Yes	Yes	Yes	Yes	Yes	Yes	No	Yes
Okajima K et al., 2006[34]	Pt#387	Yes	Unclear	Yes	Yes	Yes	Yes	No	Yes
Ridout C et al., 2008[35]	Pt#371	Yes	Yes	Yes	Yes	Yes	Yes	No	Yes
Ridout C et al., 2008[36]	Pt#375	Yes	Unclear	Yes	Yes	NA	NA	NA	Yes
Soares-Fernandes J et al., 2008[37]	Pt#376	Yes	Yes	Yes	Unclear	No	Unclear	NA	Yes
Sedel F et al., 2008[38]	Pt#445	Yes	Yes	Yes	Yes	Yes	Yes	No	Yes
Bachmann-Gagescu R et al., 2009[39]	Pt#366	Yes	Yes	Yes	Unclear	Yes	Yes	No	Yes
Silva MJ et al., 2009[40]	Pt#536	Yes	Yes	Yes	Yes	Yes	Yes	No	Yes
Coughlin C et al., 2010[41]	Pt#345	Yes	Unclear	Yes	Yes	NA	NA	NA	Yes
Tamaru S et al., 2012[42]	Pt#161	Yes	Yes	Yes	Yes	Yes	No	No	Yes
Koga Y et al., 2012[43]	Pt#452	Yes	Yes	Yes	Yes	No	Yes	No	Yes
Giribaldi G et al., 2012[44]	Pt#465	Yes	Unclear	Yes	Yes	Yes	Yes	No	Yes
Deeb K et al., 2014[45]	Pt#258	Yes	Yes	Yes	Yes	NA	NA	NA	Yes
Steller J et al., 2014[46]	Pt#277	Yes	Unclear	Yes	Yes	Yes	Yes	No	Yes

Supplementary table 1. **Critical appraisal of case reports selected for case inclusion** *(continued)*

Case report (reference)	Case ID	Q1	Q2	Q3	Q4	Q5	Q6	Q7	Q8
Kim J et al., 2014[47]	Pt#1015	Yes	Unclear	Yes	Yes	NA	NA	NA	Yes
Castiglioni C et al., 2015[48]	Pt#252	Yes	Yes	Yes	Yes	No	Yes	NA	Yes
Jauhari P et al., 2017[49]	Pt#9	Yes	Yes	Yes	Unclear	Yes	Yes	No	Yes
Kara B et al., 2017[50]	Pt#231	Yes	Yes	Yes	Yes	Yes	Yes	No	Yes
Kim J et al., 2019[51]	Pt#1014	Unclear	Yes	Yes	Yes	Yes	Yes	No	Yes
Ma Y et al., 2021[52]	Pt#166	Yes	Yes	Yes	Yes	NA	NA	NA	Yes
Gong K et al., 2021[53]	Pt#173	Yes	Unclear	Yes	Yes	Unclear	Yes	No	Yes
Pavuluri H et al., 2022[54]	Pt#146	Yes	Unclear	Yes	Yes	No	Yes	No	Yes
Hayano S et al., 2023[55]	Pt#164	Yes	Yes	Yes	Yes	Yes	Unclear	No	Yes
Tanner et al., 2023[56]	Pt#165	No	No	Unclear	Yes	NA	NA	NA	Yes
De Gusmao C et al., 2023[57]	Pt#175	Yes	Yes	Yes	Yes	Yes	Yes	No	Yes
Croci C et al., 2023[58]	Pt#172	Yes	Yes	Yes	Yes	NA	NA	NA	Yes
Laxmi V et al., 2023[59]	Pt#1006	Yes	Yes	Yes	Yes	Unclear	Yes	No	Yes
Fecarotta S et al., 2024[60]	Pt#790	Yes	Yes	Yes	Yes	Yes	Yes	No	Yes

Critical appraisal of included case reports was performed using the Joanna Briggs Institute (JBI) critical appraisal checklist:[61] Q1 – "1. Were patient's demographic characteristics clearly described?"; Q2 – "2. Was the patient's history clearly described and presented as a timeline?"; Q3 – "3. Was the current clinical condition of the patient on presentation clearly described?"; Q4 – "4. Were diagnostic tests or methods and the results clearly described?"; Q5 – "5. Was the intervention(s) or treatment procedure(s) clearly described?"; Q6 – "6. Was the post-intervention clinical condition clearly described?"; Q7 – "7. Were adverse events (harms) or unanticipated events identified and described?"; Q8 – "8. Does the case report provide takeaway lessons?". Case ID – unique number of each case in this study's cohort. NA – Not applicable.

Supplementary table 2. **Critical appraisal of case series selected for case inclusion**

Case series (reference)	Case ID	Q1	Q2	Q3	Q4	Q5	Q6	Q7	Q8	Q9	Q10
Hansen L et al., 1991[62]	Pt#195, Pt#196, Pt#197	Yes	Yes	Yes	No	No	Yes	U	NA	No	NA
Wexler I et al., 1992[63]	Pt#17, Pt#18	Yes	Yes	Yes	U	U	Yes	Yes	Yes	NA	NA
Dahl H et al., 1992[64]	Pt#191, Pt#192	Yes	Yes	Yes	No	No	Yes	U	NA	No	NA
Chun K et al., 1993[65]	Pt#198, Pt#199, Pt#200, Pt#201, Pt#202, Pt#203, Pt#204, Pt#205, Pt#206, Pt#207	Yes	Yes	Yes	No	No	Yes	U	NA	No	NA
De Meirleir L et al., 1994[66]	Pt#42, Pt#43	Yes	Yes	Yes	U	U	Yes	Yes	No	NA	NA
Awata H et al., 1994[67]	Pt#189	Yes	Yes	Yes	U	U	Yes	U	NA	NA	NA
Brown R et al., 1994[68]	Pt#213	Yes	Yes	Yes	No	No	Yes	U	NA	No	NA
Matthews P et al., 1994[69]	Pt#218, Pt#219, Pt#220, Pt#222	Yes	Yes	Yes	No	No	Yes	U	NA	No	NA
Takakubo F et al., 1995[70]	Pt#22, Pt#23, Pt#24	Yes	Yes	Yes	U	U	Yes	U	NA	NA	NA
Chun K et al., 1995[71]	Pt#25, Pt#26, Pt#27, Pt#28, Pt#29, Pt#30, Pt#31, Pt#32, Pt#33, Pt#34, Pt#35, Pt#36, Pt#37, Pt#38, Pt#39	Yes	Yes	Yes	U	U	Yes	U	NA	U	NA
Matsuda et al., 1995[72]	Pt#208, Pt#209	Yes	Yes	Yes	No	No	Yes	U	NA	No	NA
Fujii T et al., 1996[73]	Pt#46, Pt#47, Pt#48, Pt#49, Pt#50, Pt#51, Pt#52	Yes	Yes	Yes	No	No	Yes	U	U	U	NA
Lissens W et al., 1996[74]	Pt#151, Pt#152, Pt#153, Pt#154, Pt#155, Pt#156, Pt#157, Pt#158	Yes	Yes	Yes	No	No	Yes	U	U	No	NA
Tripatara A et al., 1996[75]	Pt#178, Pt#179, Pt#180	Yes	Yes	Yes	No	No	Yes	U	U	NA	NA
Marsac C et al., 1997[76]	Pt#147, Pt#148, Pt#149, Pt#150	Yes	Yes	Yes	No	No	Yes	Yes	Yes	No	NA
Wexler I et al., 1997[77]	Pt#182, Pt#183, Pt#184, Pt#185, Pt#186, Pt#187, Pt#188	Yes	Yes	Yes	U	U	Yes	Yes	Yes	NA	NA
De Meirleir L et al., 1998[78]	Pt#54, Pt#55	Yes	Yes	Yes	No	No	Yes	U	U	NA	NA
Otero L et al., 1998[79]	Pt#210, Pt#211, Pt#212	Yes	Yes	Yes	No	No	Yes	U	NA	No	NA
Lissens W et al., 1999[80]	Pt#56, Pt#57	Yes	Yes	Yes	No	No	Yes	No	No	NA	NA
Lissens W et al., 2000[81]	Pt#405, Pt#406, Pt#407, Pt#408, Pt#409, Pt#410, Pt#411, Pt#412, Pt#413, Pt#414, Pt#415, Pt#417, Pt#418, Pt#419, Pt#420, Pt#421, Pt#422, Pt#423, Pt#424, Pt#425, Pt#426, Pt#427, Pt#428, Pt#429, Pt#430, Pt#431, Pt#432, Pt#433, Pt#434, Pt#435, Pt#436, Pt#437, Pt#438, Pt#439, Pt#440, Pt#441	Yes	Yes	Yes	No	No	Yes	Yes	NA	Yes	NA
Naito E et al., 2001[82]	Pt#466, Pt#467, Pt#468, Pt#469	Yes	Yes	Yes	U	U	Yes	Yes	Yes	No	Yes
Naito E et al., 2002[83]	Pt#462, Pt#463	Yes	Yes	Yes	No	No	Yes	Yes	U	NA	Yes
Naito E et al., 2002[84]	Pt#442, Pt#443, Pt#444	Yes	Yes	Yes	No	No	Yes	Yes	Yes	No	Yes
Head R et al., 2004[85]	Pt#388, Pt#389	Yes	Yes	Yes	No	No	Yes	Yes	Yes	No	NA

Supplementary table 2. **Critical appraisal of case series selected for case inclusion** *(continued)*

**Case series (reference)**	**Case ID**	**Q1**	**Q2**	**Q3**	**Q4**	**Q5**	**Q6**	**Q7**	**Q8**	**Q9**	**Q10**
Cameron J et al., 2004[86]	Pt#390, Pt#391, Pt#392, Pt#393, Pt#394, Pt#395, Pt#396, Pt#397, Pt#398, Pt#399, Pt#400, Pt#401, Pt#402, Pt#403	Yes	Yes	Yes	No	No	Yes	Yes	Yes	No	NA
Tulinius M et al., 2005[87]	Pt#448, Pt#449	Yes	Yes	U	U	U	Yes	Yes	Yes	No	NA
Willemsen M et al., 2006[88]	Pt#381, Pt#382, Pt#383, Pt#384	Yes	Yes	Yes	Yes	U	Yes	Yes	NA	No	NA
Debray F et al., 2006[89]	Pt#385, Pt#386	Yes	Yes	Yes	No	No	Yes	U	NA	No	NA
Strassburg H et al., 2006[90]	Pt#470, Pt#471, Pt#472, Pt#473	Yes	Yes	Yes	No	No	Yes	Yes	Yes	No	NA
Debray F et al., 2008[91]	Pt#373, Pt#374	Yes	Yes	Yes	No	No	Yes	U	U	No	NA
Boichard A et al., 2008[92]	Pt#377, Pt#378, Pt#379	Yes	Yes	Yes	No	No	Yes	Yes	Yes	No	NA
Ostergaard E et al., 2009[93]	Pt#367, Pt#368, Pt#369, Pt#370	Yes	Yes	Yes	No	No	Yes	U	U	No	NA
Vasta V et al., 2009[94]	Pt#1013	Yes	Yes	Yes	No	No	Yes	No	NA	No	Yes
Koene S et al., 2009[95]	Pt#372	Yes	Yes	Yes	U	U	Yes	Yes	Yes	U	NA
Rizza T et al., 2009[96]	Pt#1027	Yes	Yes	Yes	No	No	Yes	Yes	NA	No	Yes
Quintana E et al., 2010[97]	Pt#346, Pt#347, Pt#348, Pt#349, Pt#350, Pt#351, Pt#352, Pt#353, Pt#354, Pt#355, Pt#356, Pt#357, Pt#358, Pt#359, Pt#360, Pt#361, Pt#362, Pt#363, Pt#364, Pt#365	Yes	Yes	Yes	No	No	Yes	Yes	U	No	NA
Egel R et al., 2010[98]	Pt#474	Yes	Yes	Yes	No	No	Yes	Yes	Yes	No	NA
Imbard A et al., 2011[99]	Pt#278, Pt#279, Pt#280, Pt#281, Pt#282, Pt#283, Pt#284, Pt#285, Pt#286, Pt#287, Pt#288, Pt#289, Pt#290, Pt#291, Pt#292, Pt#293, Pt#294, Pt#295, Pt#296, Pt#297, Pt#298, Pt#299, Pt#300, Pt#301, Pt#302, Pt#303, Pt#304, Pt#305, Pt#306, Pt#307, Pt#308, Pt#309, Pt#310, Pt#311, Pt#312, Pt#313, Pt#314, Pt#315, Pt#316, Pt#317, Pt#318, Pt#319, Pt#320, Pt#321, Pt#322, Pt#323, Pt#324, Pt#325, Pt#326, Pt#327, Pt#328, Pt#329, Pt#330, Pt#331, Pt#332, Pt#333, Pt#334, Pt#335, Pt#336, Pt#337, Pt#338, Pt#339	Yes	Yes	Yes	No	No	Yes	U	NA	U	Yes
Ah Mew N et al., 2011[100]	Pt#341, Pt#342, Pt#343, Pt#344	Yes	Yes	Yes	No	No	Yes	U	NA	No	NA
Glushakova L et al., 2011[101]	Pt#453, Pt#454, Pt#455, Pt#456, Pt#457, Pt#458, Pt#459, Pt#460, Pt#461	Yes	Yes	No	No	No	Yes	No	NA	U	Yes
Magner M et al., 2011[102]	Pt#537, Pt#538	Yes	Yes	Yes	No	No	No	U	NA	No	NA
Prasad C et al., 2011[103]	Pt#340	Yes	Yes	Yes	No	No	Yes	Yes	Yes	No	U

Supplementary table 2. **Critical appraisal of case series selected for case inclusion** *(continued)*

**Case series (reference)**	**Case ID**	**Q1**	**Q2**	**Q3**	**Q4**	**Q5**	**Q6**	**Q7**	**Q8**	**Q9**	**Q10**
De Ligt J et al., 2012[104]	Pt#447	Yes	Yes	Yes	U	U	Yes	U	NA	Yes	U
DeBrosse S et al., 2012[105]	Pt#475, Pt#476, Pt#477, Pt#478, Pt#479, Pt#480, Pt#481, Pt#482, Pt#483, Pt#484, Pt#485, Pt#486, Pt#487, Pt#488, Pt#489, Pt#490, Pt#491, Pt#492, Pt#493, Pt#494, Pt#495, Pt#496, Pt#497, Pt#498, Pt#499, Pt#500, Pt#501, Pt#502, Pt#503, Pt#504, Pt#505, Pt#506, Pt#507, Pt#508, Pt#509, Pt#510, Pt#511, Pt#512, Pt#513, Pt#514, Pt#515, Pt#516, Pt#517, Pt#518, Pt#519, Pt#520, Pt#521, Pt#522, Pt#523, Pt#524, Pt#525, Pt#526, Pt#527	Yes	Yes	Yes	U	U	Yes	Yes	U	Yes	Yes
Patel K et al., 2012[106]	Pt#1016, Pt#1017, Pt#1018, Pt#1019, Pt#1020, Pt#1021, Pt#1022, Pt#1024, Pt#1025	Yes	NA	NA	U	U	Yes	Yes	U	Yes	Yes
Joost K et al., 2012[107]	Pt#944, Pt#1032	Yes	Yes	Yes	Yes	Yes	No	No	No	U	NA
Ferriero R et al., 2014[108]	Pt#253, Pt#254, Pt#255, Pt#256, Pt#257	Yes	Yes	U	No	No	Yes	No	NA	No	Yes
Zhu X et al., 2015[109]	Pt#176	Yes	Yes	Yes	U	U	Yes	U	NA	U	Yes
Van Dongen S et al., 2015[110]	Pt#259, Pt#260, Pt#261, Pt#262, Pt#263, Pt#264, Pt#265, Pt#266, Pt#267, Pt#268, Pt#269, Pt#270, Pt#271, Pt#272, Pt#273, Pt#274, Pt#275, Pt#276	Yes	Yes	Yes	Yes	Yes	Yes	Yes	Yes	Yes	NA
Alfadhel M et al., 2016[111]	Pt#5, Pt#6	Yes	Yes	U	Yes	Yes	No	No	NA	No	Yes
Pronicka E et al., 2016[112]	Pt#232, Pt#233, Pt#234, Pt#235	Yes	Yes	Yes	Yes	Yes	Yes	U	NA	Yes	U
Ciara E et al., 2016[113]	Pt#236, Pt#237, Pt#238, Pt#239, Pt#240, Pt#241, Pt#242, Pt#243	Yes	Yes	Yes	Yes	Yes	Yes	U	NA	Yes	U
Qin L et al., 2016[114]	Pt#244, Pt#245, Pt#246	Yes	Yes	Yes	No	No	Yes	U	NA	No	U
Pirot N et al., 2016[115]	Pt#247, Pt#248, Pt#250	Yes	Yes	Yes	No	No	Yes	U	NA	No	NA
Wang J et al., 2016[116]	Pt#4	Yes	Yes	Yes	U	U	U	U	NA	U	Yes
Asencio C et al., 2016[117]	Pt#251	Yes	Yes	Yes	No	No	Yes	Yes	NA	No	Yes
Yoshida T et al., 2017[118]	Pt#7, Pt#8	U	Yes	Yes	No	No	Yes	Yes	U	NA	NA
Fang F et al., 2017[119]	Pt#58, Pt#59	Yes	Yes	Yes	Yes	Yes	Yes	Yes	No	Yes	Yes

Supplementary table 2. **Critical appraisal of case series selected for case inclusion** *(continued)*

**Case series (reference)**	**Case ID**	**Q1**	**Q2**	**Q3**	**Q4**	**Q5**	**Q6**	**Q7**	**Q8**	**Q9**	**Q10**
Shin H et al., 2017[120]	Pt#60, Pt#61, Pt#62, Pt#63, Pt#64, Pt#65, Pt#66, Pt#67, Pt#68, Pt#69, Pt#70, Pt#71, Pt#72, Pt#73, Pt#74, Pt#75, Pt#76, Pt#77, Pt#78, Pt#79, Pt#80, Pt#81, Pt#82, Pt#83, Pt#84, Pt#85, Pt#86, Pt#87, Pt#88, Pt#89, Pt#90, Pt#91, Pt#92, Pt#93, Pt#94, Pt#95, Pt#96, Pt#97, Pt#98, Pt#99, Pt#100, Pt#101, Pt#102, Pt#103, Pt#104, Pt#105, Pt#106, Pt#107, Pt#108, Pt#109, Pt#110, Pt#111, Pt#112, Pt#113, Pt#114, Pt#115, Pt#116, Pt#117, Pt#118, Pt#119, Pt#120, Pt#121, Pt#122, Pt#123, Pt#124, Pt#125	Yes	Yes	Yes	Yes	Yes	No	No	No	Yes	Yes
Winters L et al., 2017[121]	Pt#230	Yes	Yes	Yes	No	No	Yes	Yes	NA	No	NA
Jou C et al., 2019[122]	Pt#10, Pt#11, Pt#12, Pt#13, Pt#14	Yes	Yes	Yes	Yes	Yes	Yes	U	NA	Yes	Yes
Horga A et al., 2019[123]	Pt#127, Pt#128	Yes	Yes	Yes	No	No	Yes	U	NA	No	NA
Zouvelou V et al., 2019[124]	Pt#1011	Yes	Yes	Yes	Yes	Yes	No	U	U	U	NA
Dong H et al., 2019[125]	Pt#126	Yes	Yes	Yes	Yes	Yes	Yes	Yes	NA	Yes	Yes
Hu C et al., 2020[126]	Pt#174	Yes	Yes	Yes	Yes	Yes	Yes	U	NA	Yes	Yes
Pavlu-Pereira H et al., 2020[127]	Pt#530, Pt#531, Pt#532, Pt#534, Pt#535	Yes	Yes	Yes	U	U	Yes	Yes	Yes	No	Yes
Ziats M et al., 2020[128]	Pt#16	Yes	Yes	Yes	Yes	Yes	U	U	NA	Yes	Yes
Sen K et al., 2021[129]	Pt#170, Pt#171	Yes	Yes	Yes	U	U	Yes	Yes	Yes	NA	NA
Kose M et al., 2021[130]	Pt#214, Pt#215	Yes	Yes	Yes	Yes	Yes	Yes	U	NA	Yes	Yes
Goergen et al., 2021[131]	Pt#1010	Yes	Yes	Yes	Yes	Yes	U	U	NA	Yes	U
Schon K et al., 2021[132]	Pt#1026	Yes	Yes	Yes	Yes	Yes	Yes	Yes	NA	Yes	Yes
Stenton S et al., 2022[133]	Pt#130, Pt#131, Pt#135, Pt#136, Pt#137, Pt#140, Pt#141, Pt#142	Yes	Yes	Yes	Yes	Yes	No	Yes	Yes	No	Yes
Inui T et al., 2022[134]	Pt#162, Pt#163	Yes	Yes	Yes	No	No	Yes	Yes	Yes	NA	NA
Coste T et al., 2022[135]	Pt#1007, Pt#1008, Pt#1009	Yes	Yes	Yes	U	U	Yes	Yes	NA	Yes	NA
Koh H et al., 2022[136]	Pt#145	Yes	Yes	Yes	Yes	Yes	Yes	Yes	NA	Yes	Yes
Kistol D et al., 2023[137]	Pt#995, Pt#996, Pt#997, Pt#998, Pt#999, Pt#1000, Pt#1001	Yes	Yes	Yes	U	U	Yes	No	No	Yes	NA
Wang Y et al., 2023[138]	Pt#1005	Yes	Yes	Yes	Yes	Yes	Yes	Yes	NA	Yes	Yes
Zhou H et al., 2023[139]	Pt#169	Yes	Yes	Yes	Yes	Yes	No	No	NA	U	U
Savvidou A et al., 2024[140]	Pt#987, Pt#988, Pt#989, Pt#990, Pt#991, Pt#992, Pt#993, Pt#994	Yes	Yes	Yes	Yes	Yes	Yes	Yes	Yes	Yes	NA
Alsehli H et al., 2024[141]	Pt#1002	Yes	Yes	Yes	Yes	Yes	No	Yes	NA	U	Yes

Supplementary table 2. **Critical appraisal of case series selected for case inclusion** *(continued)*

**Case series (reference)**	**Case ID**	**Q1**	**Q2**	**Q3**	**Q4**	**Q5**	**Q6**	**Q7**	**Q8**	**Q9**	**Q10**
Ferreira T et al., 2024[142]	Pt#1003	Yes	Yes	Yes	Yes	Yes	Yes	U	NA	Yes	NA
Westenius E et al., 2024[143]	Pt#1004	Yes	Yes	Yes	Yes	Yes	U	Yes	NA	U	U
Olimpio C et al., 2024[144]	Pt#1012	Yes	Yes	Yes	Yes	Yes	Yes	Yes	NA	Yes	NA

Critical appraisal of included case series was performed using the Joanna Briggs Institute (JBI) critical appraisal checklist:[145] Q1 – "1. Were there clear criteria for inclusion in the case series? "; Q2 – "2. Was the condition measured in a standard, reliable way for all participants included in the case series?"; Q3 – "3. Were valid methods used for identification of the condition for all participants included in the case series?"; Q4 – "4. Did the case series have consecutive inclusion of participants?"; Q5 – "5. Did the case series have complete inclusion of participants?"; Q6 – "6. Was there clear reporting of the demographics of the participants in the study?"; Q7 – "7. Was there clear reporting of clinical information of the participants?"; Q8 – "8. Were the outcomes or follow-up results of cases clearly reported?"; Q9 – "9. Was there clear reporting of the presenting site(s)/clinic(s) demographic information?"; Q10 – "10. Was statistical analysis appropriate?". Case ID – unique number of each case in this study's cohort. NA – Not applicable. U – Unclear.

Supplementary Table 3. **Data extraction form**

**Categories**	**Sub categories**	**Human phenotype ontology (HPO) terms**
General cases characteristics	• Sex (male, female, NA)<br>• Affected family member (yes, no, NA)<br>• Age at presentation/death in days (1 month – 30 days, 1 year – 360 days)<br>• Age at last follow up (1 month – 30 days, 1 year – 360 days)<br>• Last known status (alive, dead, NA)	
Genetic analysis	• Nucleotide change (NM_000284.3)<br>• Amino acid change (p. 1-letter code)<br>• Mode of inheritance (de novo, confirmed in mother, NA)<br>• Type of genetic testing (Sanger sequencing, gene panel, exome, genome, NA)	
Laboratory findings	• Blood: elevated lactate (yes, no, NA); elevated pyruvate (yes, no, NA); elevated alanine (yes, no, NA); lactic acidosis (yes, no, NA)<br>• Cerebrospinal fluid: elevated lactate (yes, no, NA);<br>• Urine: elevated lactate (yes, no, NA)<br>• Enzyme activity (yes, proven in muscle; yes, proven in fibroblasts; yes, proven in lymphocytes; no, unproven; NA; enzyme activity values if available)<br>• Other laboratory findings	• Elevated serum lactate (HP:0002151); blood acidosis (HP:0001941); increased serum pyruvate (HP:0003542); hyperalaninemia (HP:0003348)<br>• Elevated CSF lactate (HP:0002490)<br>• Elevated lactate in urine (HP:0003648)

Supplementary Table 3. **Data extraction form** *(continued)*

Categories	Sub categories	Human phenotype ontology (HPO) terms
Clinical findings	• Seizures (yes, no, NA); age at first seizures (1 month – 30 days, 1 year – 360 days); types of seizures (text); EEG findings (text)<br>• Ataxia (yes, no, NA); dystonia (yes, no, NA); muscular hypertonia (yes, no, NA); muscular hypotonia (yes, no, NA);<br>• Impaired hearing (yes, no, NA); impaired vision (yes, no, NA)<br>• Strabismus (yes, no, NA); nystagmus (yes, no, NA); ophthalmoplegia (yes, no, NA)<br>• Neuropathy (yes, no, NA); *Guillain-Barré* syndrome (yes, no, NA)<br>• Dysphagia/feeding difficulties (yes, no, NA); drooling (yes, no, NA)<br>• Microcephaly (yes, no, NA); intellectual disability (no, mild, moderate, severe, NA); IQ; developmental delay (no, mild, moderate, severe, NA)<br>• Osteopenia (yes, no, NA); dysmorphic features (yes, no, NA); skeletal deformities (yes, no, NA)<br>• Other clinical findings (text)	• Seizures (HP:0001250)<br>• Ataxia (HP:0001251); dystonia (HP:0001332); muscle hypertonia (HP:0001276); muscle hypotonia (HP:0001252); any involuntary abnormal movements (excl. dyskinesia, ataxia, dystonia) (HP:0004305); other gait disturbances not listed before (HP:0001288); other and not specified movements disorders not listed before (HP0100022); muscle weakness (HP:0001324)<br>• Any hearing impairment (HP:0000365); Visual impairment (HP:0000505)<br>• Strabismus (HP:0000486); nystagmus (HP:0000639); ophthalmoplegia (HP:0000602); ptosis (HP:0000508), other and not specified abnormal eye movement not listed before (HP:0000496); abnormal fundus findings (HP:0001098)<br>• Peripheral neuropathy (HP:0009830); myopathy (HP:0003198); any signs of abnormal reflexes (excl. Guilian-Barre syndrome) (HP:0031826)<br>• Feeding difficulties (HP:0011968); drooling (HP:0002307); decreased body weight or failure to thrive (HP:0004325); dysarthria (HP:0001260)<br>• Microcephaly (HP:0000252); any severity intellectual disability (HP:0001249); any severity developmental delay (HP:0012758); specified as motor delay (HP:0001270); specified as global developmental delay (HP:0001263); developmental regression (HP:0002376); any abnormal emotions (HP0100851)<br>• Osteopenia (HP:0000938); any dysmorphic facial features (HP:0001999); any skeletal deformities (HP:0011842)<br>• Apnea (HP:0002104), any signs of abnormal breathing (excluding apnea, respiratory failure) (HP:0002793), any respiratory failure (HP:0002878)<br>• Hemiplegia or hemiparesis (HP:0004374); tetraplegia or tetraparesis (HP:0030182)<br>• Any drowsiness (HP:0002329); any fatigue (HP:0012378); any lethargy (HP:0001254); encephalopathy (HP:0001298)<br>• First presentation or exacerbation after febrile illness (HP:0033184)<br>• Any arrhythmia (HP:0011675)

Supplementary Table 3. **Data extraction form** *(continued)*

Categories	Sub categories	Human phenotype ontology (HPO) terms
Neuroimaging findings	• Basal ganglia lesions (yes, no, NA)<br>• Cerebral atrophy (yes, no, NA)<br>• Cerebellar atrophy (yes, no, NA)<br>• Other findings (text)	• Abnormal basal ganglia (HP:0002134); abnormal substantia nigra (HP:0045007); abnormal dentate nucleus (HP0100321); abnormal putamen (HP:0031982); abnormal caudate nucleus (HP:0002339); abnormal globus pallidus (HP:0002453)<br>• Cerebral atrophy (HP:0002059)<br>• Cerebellar atrophy (HP:0001272)<br>• Corpus callosum hypoplasia (HP:0002079); corpus callosum agenesis (HP:0001274)<br>• Hydrocephalus or ventriculomegaly (HP:0002118)<br>• Periventricular leukomalacia (HP:0006970); abnormal myelination (HP:0012447); abnormal cerebral white matter (HP:0002500)<br>• Abnormal brainstem (HP:0002363)<br>• Abnormal midbrain (HP:0002418), abnormal thalamus (HP:0010663)
Activities of daily living	• Able to sit independently (yes, no, NA); able to walk independently (yes, no, NA); able to eat independently (yes, no, NA)<br>• Able to communicate with sounds (yes, no, NA); able to communicate with words (yes, no, NA); able to communicate with sentences (yes, no, NA)<br>• Able to perform personal hygiene independently (yes, no, NA)<br>• Attending/finished regular school (yes, no, NA)<br>• Attending/finished school for children with special needs (yes, no, NA)	
Pathogenetic or specific treatment	• Ketogenic diet (yes, with perceived clinical benefit, yes, without perceived clinical benefit, no, NA)<br>• Thiamine supplementation (yes, with perceived clinical benefit, yes, without perceived clinical benefit, no, NA)<br>• Other specific treatment	

Supplementary Table 3. **Data extraction form** *(continued)*

**Categories**	**Sub categories**	**Human phenotype ontology (HPO) terms**
Prenatal and delivery characteristics	• Intrauterine growth retardation (yes, no, NA); poor intrauterine foetal movements (yes, no, NA); polyhydramnios (yes, no, NA); oligohydramnios (yes, no, NA); abnormal foetal ultrasound (yes, no, NA)<br>• Natural vaginal delivery (yes, no, NA); assisted delivery (yes, no, NA); caesarean section (yes, no, NA)<br>• Preterm birth (yes, no, NA); delivery at term (yes, no, NA)<br>• Low APGAR scores (< 5) (yes, no, NA); resuscitation at birth (yes, no, NA)<br>• Low birth weight (< 3rd percentile) (yes, no, NA); low birth length (< 3rd percentile) (yes, no, NA); small head circumference (< 3rd percentile) (yes, no, NA)<br>• Other prenatal and delivery findings.	• Intrauterine growth retardation (HP:0001511); any poor intrauterine foetal movements (HP:0001557); polyhydramnios (HP:0001561); oligohydramnios (HP:0001562)<br>• Any premature birth (HP:0001622)<br>• Low APGAR scores (< 5) (HP:0030917)

Supplementary table 3 provides summarized categories and sub categories of data collected in the survey and from published cases. Input choices are listed in the brackets, when applicable. After data collection, phenotypes were converted to the Human phenotype ontology (HPO) terms. NA – not available.

Supplementary Table 4. **List of high risk duplicate cases from the literature**

Variant	Case ID	Sex	Source (reference)	Evidence of duplicate case	Overlapping authors	Final case ID
c.787C>G, p.Arg263Gly	Pt#421	M	Lissens W et al., 2000[81]	Inherited. Enzyme activity: 50%	De Meirleir L. Lissens W	Pt#359
c.787C>G, p.Arg263Gly	Pt#359	M	Quintana E et al., 2010[97]	Inherited. Enzyme activity: 50%		
c.787C>G, p.Arg263Gly	Pt#53	M	Naito E et al., 1997[22]	Enzyme activity: 50%	Naito E. Ito M. Kuroda. Kuroda Y.	Pt#53
c.787C>G, p.Arg263Gly	Pt#422	M	Lissens W et al., 2000[81]	Enzyme activity: 50%		
c.787C>G, p.Arg263Gly	Pt#17	M	Wexler I et al., 1992[63]	First presentation: 5 months	Wexler ID. Hemalatha SG. Berry SA. Patel MS. Kerr DS.	Pt#17
c.787C>G, p.Arg263Gly	Pt#185	M	Wexler I et al., 1997[77]	First presentation: 5 months		
c.787C>G, p.Arg263Gly	Pt#18	M	Wexler I et al., 1992[63]	First presentation: 12 months	Wexler ID. Hemalatha SG. Berry SA. Patel MS. Kerr DS.	Pt#18
c.787C>G, p.Arg263Gly	Pt#186	M	Wexler I et al., 1997[77]	First presentation: 12 months		
c.904C>T, p.Arg302Cys	Pt#192	F	Dahl H et al., 1992[64]	Neonatal first presentation. Enzyme activity: 58% (range 57-68%).	Brown GK	Pt#192
c.904C>T, p.Arg302Cys	Pt#1021	F	Patel K et al., 2012[106]	Neonatal first presentation. Enzyme activity: 68%.		
c.904C>T, p.Arg302Cys	Pt#281	F	Imbard A et al., 2011[99]	Neonatal first presentation. Enzyme activity: 18.2%.	Bucourt M	Pt#250
c.904C>T, p.Arg302Cys	Pt#250	F	Pirot N et al., 2016[115]	Neonatal first presentation. Enzyme activity: 18%		
c.1133G>A, p.Arg378His	Pt#36	M	Chun K et al., 1995[71]	Enzyme activity: 22%	Robinson BH	Pt#36
c.1133G>A, p.Arg378His	Pt#429	M	Lissens W et al., 2000[81]	Enzyme activity: 22%		
c.1133G>A, p.Arg378His	Pt#60	M	Shin H et al., 2017[120]	Enzyme activity: 0.68 (fibroblasts)	Grahame G, Kerr DS	Pt#60
c.1133G>A, p.Arg378His	Pt#518	M	DeBrosse S et al., 2012[105]	Enzyme activity: 0.68 (fibroblasts). Enzyme activity: 5% (lymphocytes). Age at death: 43 months.		
c.1133G>A, p.Arg378His	Pt#184	M	Wexler I et al., 1997[77]	Enzyme activity: 5% (lymphocytes). Age at death: 41 months.	Kerr DS	
c.1133G>A, p.Arg378His	Pt#70	M	Shin H et al., 2017[120]	Enzyme activity: 0.33.	Grahame G, Kerr DS	Pt#70
c.1133G>A, p.Arg378His	Pt#520	M	DeBrosse S et al., 2012[105]	Enzyme activity: 0.31.		

Supplementary Table 4. **List of high risk duplicate cases from the literature** *(continued)*

Variant	Case ID	Sex	Source (reference)	Evidence of duplicate case	Overlapping authors	Final case ID
c.1133G>A, p.Arg378His	Pt#79	M	Shin H et al., 2017[120]	Enzyme activity: 0.42.	Grahame G, Kerr DS	Pt#79
c.1133G>A, p.Arg378His	Pt#521	M	DeBrosse S et al., 2012[105]	Enzyme activity: 0.41.		
c.1133G>A, p.Arg378His	Pt#82	M	Shin H et al., 2017[120]	Enzyme activity: 0.23.	Grahame G, Kerr DS	Pt#82
c.1133G>A, p.Arg378His	Pt#522	M	DeBrosse S et al., 2012[105]	Enzyme activity: 0.24 or 10%.		
c.1133G>A, p.Arg378His	Pt#428	M	Lissens W et al., 2000[81]	Enzyme activity: 10%.	Kerr DS	
c.1133G>A, p.Arg378His	Pt#182	M	Wexler I et al., 1997[77]	Age at death: 13 months. Enzyme activity: 30%	Dahl HH	Pt#182
c.1133G>A, p.Arg378His	Pt#197	NA	Hansen L et al., 1991[62]	Age at death: 13 months. Enzyme activity: 30%		
c.491A>G, p.Asn164Ser	Pt#69	M	Shin H et al., 2017[120]	Enzyme activity: 29%	Kerr DS	Pt#69
c.491A>G, p.Asn164Ser	Pt#415	M	Lissens W et al., 2000[81]	Enzyme activity: 26%		
c.491A>G, p.Asn164Ser	Pt#483	M	DeBrosse S et al., 2012[105]	Enzyme activity: 27%		
c.1132C>T, p.Arg378Cys	Pt#114	F	Shin H et al., 2017[120]	Enzyme activity: 0.37.	Grahame G, Kerr DS	Pt#114
c.1132C>T, p.Arg378Cys	Pt#515	F	DeBrosse S et al., 2012[105]	Enzyme activity: 0.38.		
c.1132C>T, p.Arg378Cys	Pt#77	M	Shin H et al., 2017[120]	Enzyme activity: 0.56.	Grahame G, Kerr DS	Pt#77
c.1132C>T, p.Arg378Cys	Pt#516	M	DeBrosse S et al., 2012[105]	Enzyme activity: 0.56.		
c.1132C>T, p.Arg378Cys	Pt#90	M	Shin H et al., 2017[120]	Enzyme activity: 0 (lymphocytes)	Grahame G, Kerr DS	Pt#90
c.1132C>T, p.Arg378Cys	Pt#517	M	DeBrosse S et al., 2012[105]	Enzyme activity: 0 (lymphocytes)		
c.1142_1145dup, p.Trp383SerfsTer6	Pt#169	NA	Zhou H et al., 2023[139]	DNV	Zhou H, Fu F, Wang Y, Li R, Cheng K, Huang R, Yu Q, Lei T, Yang X, Liao C	Pt#169
c.1142_1145dup, p.Trp383SerfsTer6	Pt#1005	F	Wang Y et al., 2023[138]	DNV		
c.934_940del, p.Ser312ValfsTer12	Pt#107	F	Shin H et al., 2017[120]	Enzyme activity: 1.05.	Grahame G, Kerr DS	Pt#107
c.934_940del, p.Ser312ValfsTer12	Pt#507	F	DeBrosse S et al., 2012[105]	Enzyme activity: 1.04.		
c.214C>T, p.Arg72Cys	Pt#35	M	Chun K et al., 1995[71]	Last known age: 8 years. Enzyme activity: 20%	MacKay N, Robinson BH	Pt#35
c.214C>T, p.Arg72Cys	Pt#392	M	Cameron J et al., 2004[86]	Last known age: 8 years. Enzyme activity: 30%		

Supplementary Table 4. **List of high risk duplicate cases from the literature** *(continued)*

Variant	Case ID	Sex	Source (reference)	Evidence of duplicate case	Overlapping authors	Final case ID
c.262C>T, p.Arg88Cys	Pt#232	M	Pronicka E et al., 2016[112]	Inherited. First presentation: 24 months	Pronicka E. Piekutowska-Abramczuk D. Ciara E. Trubicka J. Rokicki D. Karkucińska-Więckowska A. Pajdowska M. Halat P. Pronicki M. Krajewska-Walasek M. Płoski R. Mayr J. Sperl W	Pt#232
c.262C>T, p.Arg88Cys	Pt#236	M	Ciara E et al., 2016[113]	Inherited. First presentation: 24 months. Last known age: 102 months. Brother of Pt#237		
c.262C>T, p.Arg88Cys	Pt#472	M	Strassburg H et al., 2006[90]	Inherited. Last known age: 108 months. Brother of Pt#471.	Mayr J. Sperl W	
c.262C>T, p.Arg88Cys	Pt#237	M	Ciara E et al., 2016[113]	Inherited. Brother of Pt#236.	Mayr J. Sperl W	Pt#237
c.262C>T, p.Arg88Cys	Pt#471	M	Strassburg H et al., 2006[90]	Inherited. Brother of Pt#472.		
c.483C>T, p.?	Pt#309	M	Imbard A et al., 2011[99]	First presentation: after birth. Age at death: 1 month.	Boutron A. de Lonlay P. de Baulny HO. Brivet M	Pt#309
c.483C>T, p.?	Pt#377	M	Boichard A et al., 2008[92]	First presentation: after birth. Age at death: 1 month.		
c.483C>T, p.?	Pt#313	M	Imbard A et al., 2011[99]	First presentation: 1 month	Boutron A. de Lonlay P. de Baulny HO. Brivet M	Pt#313
c.483C>T, p.?	Pt#379	M	Boichard A et al., 2008[92]	First presentation: 1 month		
c.380G>A, p.Arg127Gln	Pt#66	M	Shin H et al., 2017[120]	Enzyme activity: 0.36.	Grahame G. Kerr DS	Pt#66
c.380G>A, p.Arg127Gln	Pt#478	M	DeBrosse S et al., 2012[105]	Enzyme activity: 0.36.		
c.380G>A, p.Arg127Gln	Pt#78	M	Shin H et al., 2017[120]	Enzyme activity: 0.24.	Grahame G. Kerr DS	Pt#78
c.380G>A, p.Arg127Gln	Pt#479	M	DeBrosse S et al., 2012[105]	Enzyme activity: 0.24.		
c.905G>A, p.Arg302His	Pt#118	F	Shin H et al., 2017[120]	Enzyme activity: 0.64.	Grahame G. Kerr DS	Pt#118
c.905G>A, p.Arg302His	Pt#506	F	DeBrosse S et al., 2012[105]	Enzyme activity: 0.62.		
c.787C>T, p.Arg263Ter	Pt#97	F	Shin H et al., 2017[120]	Enzyme activity: 0.57.	Grahame G. Kerr DS	Pt#97
c.787C>T, p.Arg263Ter	Pt#494	F	DeBrosse S et al., 2012[105]	Enzyme activity: 0.56.		
c.355C>T, p.Arg119Trp	Pt#108	F	Shin H et al., 2017[120]	Enzyme activity: 0.72.	Grahame G. Kerr DS	Pt#108
c.355C>T, p.Arg119Trp	Pt#477	F	DeBrosse S et al., 2012[105]	Enzyme activity: 0.73.		

Supplementary Table 4. **List of high risk duplicate cases from the literature** *(continued)*

Variant	Case ID	Sex	Source (reference)	Evidence of duplicate case	Overlapping authors	Final case ID
c.498C>T, p.?	Pt#300	F	Imbard A et al., 2011[99]	Last known age: 60 months	Boutron A. de Lonlay P. de Baulny HO. Brivet M.	Pt#300
c.498C>T, p.?	Pt#378	F	Boichard A et al., 2008[92]	Last known age: 60 months		
c.628A>G, p.Met210Val	Pt#64	M	Shin H et al., 2017[120]	Enzyme activity: 0.11.	Grahame G.Kerr DS	Pt#64
c.628A>G, p.Met210Val	Pt#178	M	Tripatara A et al., 1996[75]	Enzyme activity: 0.15.	Kerr DS. Lusk MM	
c.628A>G, p.Met210Val	Pt#488	M	DeBrosse S et al., 2012[105]	Enzyme activity: 0.12.	Grahame G.Kerr DS. Lusk-Kopp M	
c.933_935del, p.Arg311del	Pt#63	M	Shin H et al., 2017[120]	Enzyme activity: 0	Kerr DS	Pt#63
c.933_935del, p.Arg311del	Pt#179	NA	Tripatara A et al., 1996[75]	Enzyme activity: 0	Kerr DS	
c.933_935del, p.Arg311del	Pt#234	F	Pronicka E et al., 2016[112]	First presentation: after birth.	Pronicka E. Piekutowska-Abramczuk D. Ciara E. Trubicka J. Rokicki D. Karkucińska-Więckowska A. Pajdowska M. Halat P. Pronicki M. Krajewska-Walasek M. Płoski R	Pt#234
c.933_935del, p.Arg311del	Pt#242	F	Ciara E et al., 2016[113]	First presentation: after birth.		
c.858_861dup, p.Arg288LeufsTer10	Pt#233	F	Pronicka E et al., 2016[112]	First presentation: 3 months.	Pronicka E. Piekutowska-Abramczuk D. Ciara E. Trubicka J. Rokicki D. Karkucińska-Więckowska A. Pajdowska M. Halat P. Pronicki M. Krajewska-Walasek M. Płoski R	Pt#233
c.858_861dup, p.Arg288LeufsTer10	Pt#241	F	Ciara E et al., 2016[113]	First presentation: 3 months.		
c.592G>A, p.Ala198Thr	Pt#387	M	Okajima K et al., 2006[34]	Enzyme activity: 27%	Okajima K. Kerr DS	Pt#387
c.592G>A, p.Ala198Thr	Pt#486	M	DeBrosse S et al., 2012[105]	Enzyme activity: 26%		
c.871G>A, p.Gly291Arg	Pt#122	F	Shin H et al., 2017[120]	Enzyme activity: 0.56.	Grahame G. Kerr DS	Pt#122
c.871G>A, p.Gly291Arg	Pt#499	F	DeBrosse S et al., 2012[105]	Enzyme activity: 0.56.		
c.262C>A, p.Arg88Ser	Pt#150	M	Marsac C et al., 1997[76]	First presentation: 18 months. Enzyme activity: 7%	Marsac C. Saudubray JM	Pt#150
c.262C>A, p.Arg88Ser	Pt#329	M	Imbard A et al., 2011[99]	First presentation: 18 months. Enzyme activity: 8%		

Supplementary Table 4. **List of high risk duplicate cases from the literature** *(continued)*

Variant	Case ID	Sex	Source (reference)	Evidence of duplicate case	Overlapping authors	Final case ID
c.302G>T, p.Cys101Phe	Pt#253	F	Ferriero R et al., 2014[108]	Enzyme activity 47%;	Boutron A. Brivet M.	Pt#253
c.302G>T, p.Cys101Phe	Pt#299	F	Imbard A et al., 2011[99]	Enzyme activity 49%	Boutron A. Brivet M	
c.422G>A, p.Arg141Gln	Pt#98	F	Shin H et al., 2017[120]	Enzyme activity: 0.83.	Grahame G. Kerr DS	Pt#98
c.422G>A, p.Arg141Gln	Pt#481	F	DeBrosse S et al., 2012[105]	Enzyme activity: 0.82.		
c.650C>T, p.Pro217Leu	Pt#61	M	Shin H et al., 2017[120]	Enzyme activity: 0,32	Grahame G. Kerr DS	Pt#61
c.650C>T, p.Pro217Leu	Pt#181	M	Hemalatha S et al., 1995[18]	Enzyme activity: 0,32. Death in neonatal period.	Kerr DS	
c.650C>T, p.Pro217Leu	Pt#490	M	DeBrosse S et al., 2012[105]	Enzyme activity: 0,31. Death in neonatal period.	Grahame G. Kerr DS	
c.963_977dup p.Lys321_Val325dup	Pt#83	M	Shin H et al., 2017[120]	Enzyme activity: 0.78.	Grahame G. Kerr DS	Pt#83
c.963_977dup p.Lys321_Val325dup	Pt#509	M	DeBrosse S et al., 2012[105]	Enzyme activity: 0.77.		
c.383G>A, p.Gly128Asp	Pt#84	M	Shin H et al., 2017[120]	Enzyme activity: 0.50.	Grahame G. Kerr DS	Pt#84
c.383G>A, p.Gly128Asp	Pt#480	M	DeBrosse S et al., 2012[105]	Enzyme activity: 0.48.		
c.499G>A, p.Val167Met	Pt#100	F	Shin H et al., 2017[120]	Enzyme activity: 0.24.	Grahame G. Kerr DS	Pt#100
c.499G>A, p.Val167Met	Pt#484	F	DeBrosse S et al., 2012[105]	Enzyme activity: 0.24.		
c.584G>C, p.Gly195Ala	Pt#373	M	Debray F et al., 2008[91]	Enzyme activity: 35%	MacKay N, Robinson BH	Pt#373
c.584G>C, p.Gly195Ala	Pt#399	M	Cameron J et al., 2004[86]	Enzyme activity: 35%		
c.584G>C, p.Gly195Ala	Pt#374	M	Debray F et al., 2008[91]	Enzyme activity: 18%	MacKay N, Robinson BH	Pt#374
c.584G>C, p.Gly195Ala	Pt#400	M	Cameron J et al., 2004[86]	Enzyme activity: 17%		
c.831+1G>A, p.?	Pt#120	F	Shin H et al., 2017[120]	Enzyme activity: 0.32.	Grahame G. Kerr DS	Pt#120
c.831+1G>A, p.?	Pt#496	F	DeBrosse S et al., 2012[105]	Enzyme activity: 0.31.		
c.862C>T, p.Arg288Cys	Pt#104	F	Shin H et al., 2017[120]	Enzyme activity: 0.72	Grahame G. Kerr DS	Pt#104
c.862C>T, p.Arg288Cys	Pt#498	F	DeBrosse S et al., 2012[105]	Enzyme activity: 0.77		
c.616G>A, p.Glu206Lys	Pt#103	F	Shin H et al., 2017[120]	Enzyme activity: 1.5	Grahame G. Kerr DS	Pt#103
c.616G>A, p.Glu206Lys	Pt#487	F	DeBrosse S et al., 2012[105]	Enzyme activity: 1.5		

Supplementary Table 4. **List of high risk duplicate cases from the literature** *(continued)*

**Variant**	**Case ID**	**Sex**	**Source (reference)**	**Evidence of duplicate case**	**Overlapping authors**	**Final case ID**
c.728A>G, p.Tyr243Cys	Pt#115	F	Shin H et al., 2017[120]	Enzyme activity: 0.48.	Grahame G. Kerr DS	Pt#115
c.728A>G, p.Tyr243Cys	Pt#491	F	DeBrosse S et al., 2012[105]	Enzyme activity: 0.48.		
c.748C>A, p.Pro250Thr	Pt#385	M	Debray F et al., 2006[89]	Enzyme activity: 0.71	MacKay N, Robinson BH	Pt#385
c.748C>A, p.Pro250Thr	Pt#395	M	Cameron J et al., 2004[86]	Enzyme activity: 0.71		
c.847G>A, p.Glu283Lys	Pt#105	F	Shin H et al., 2017[120]	Enzyme activity: 0.39.	Grahame G. Kerr DS	Pt#105
c.847G>A, p.Glu283Lys	Pt#497	F	DeBrosse S et al., 2012[105]	Enzyme activity: 0.39.		
c.899+1G>C, p.?	Pt#121	F	Shin H et al., 2017[120]	Enzyme activity: 0.71.	Grahame G. Kerr DS	Pt#121
c.899+1G>C, p.?	Pt#501	F	DeBrosse S et al., 2012[105]	Enzyme activity: 0.77.		
c.224A>C, p.Glu75Ala	Pt#335	M	Imbard A et al., 2011[99]	Enzyme activity: 13% (lymphocytes)	Boutron A. Saudubray JM. Brivet M.	Pt#335
c.224A>C, p.Glu75Ala	Pt#445	M	Sedel F et al., 2008[38]	Enzyme activity: 13% (lymphocytes)		
c.265G>A, p.Gly89Ser	Pt#208	F	Matsuda J et al., 1995[72]	Enzyme activity: 18%. Last known age: 60 months.	Naito E. Ito M. Yokota I. Kuroda Y.	Pt#208
c.265G>A, p.Gly89Ser	Pt#229	F	Naito E et al., 1999 (25)	Enzyme activity: 15%. Last known age: 60 months.		
c.269T>C, p.Phe90Ser	Pt#112	F	Shin H et al., 2017[120]	Enzyme activity: 0.59	Grahame G. Kerr DS	Pt#112
c.269T>C, p.Phe90Ser	Pt#475	F	DeBrosse S et al., 2012[105]	Enzyme activity: 0.58		
c.301T>C, p.Cys101Arg	Pt#111	F	Shin H et al., 2017[120]	Enzyme activity: 0.92.	Grahame G. Kerr DS	Pt#111
c.301T>C, p.Cys101Arg	Pt#476	F	DeBrosse S et al., 2012[105]	Enzyme activity: 0.92.		
c.530T>C, p.Ile177Thr	Pt#65	M	Shin H et al., 2017[120]	Enzyme activity: 10%	Grahame G. Kerr DS	Pt#65
c.530T>C, p.Ile177Thr	Pt#485	M	DeBrosse S et al., 2012[105]	Enzyme activity: 9%		
c.629T>C, p.Met210Thr	Pt#81	M	Shin H et al., 2017[120]	Enzyme activity: 0.42.	Grahame G. Kerr DS	Pt#81
c.629T>C, p.Met210Thr	Pt#489	M	DeBrosse S et al., 2012[105]	Enzyme activity: 0.41.		
c.728A>C, p.Tyr243Ser	Pt#305	M	Imbard A et al., 2011[99]	Enzyme: 7%	Marsac C	Pt#305
c.728A>C, p.Tyr243Ser	Pt#464	M	Benelli C et al., 2002[27]	Enzyme: 7%		
c.905G>T, p.Arg302Leu	Pt#424	F	Lissens W et al., 2000[81]	Last known age: 12 months	Ito M. Naito E. Kuroda Y	Pt#424
c.905G>T, p.Arg302Leu	Pt#467	F	Naito E et al., 2001[82]	Last known age: 12 months		

Supplementary Table 4. **List of high risk duplicate cases from the literature** *(continued)*

**Variant**	**Case ID**	**Sex**	**Source (reference)**	**Evidence of duplicate case**	**Overlapping authors**	**Final case ID**
c.986_998dup, p.Glu333AspfsTer11	Pt#106	F	Shin H et al., 2017[120]	Enzyme activity: 0.59	Grahame G. Kerr DS	Pt#106
c.986_998dup, p.Glu333AspfsTer11	Pt#511	F	DeBrosse S et al., 2012[105]	Enzyme activity: 0.58		
c.1083_1102dup, p.Ile368ArgfsTer63	Pt#94	F	Shin H et al., 2017[120]	Enzyme activity: 0.29.	Grahame G. Kerr DS	Pt#94
c.1083_1102dup, p.Ile368ArgfsTer63	Pt#513	F	DeBrosse S et al., 2012[105]	Enzyme activity: 0.29.		
c.1121_1159dup, p.Phe386_Lys387ins13	Pt#71	M	Shin H et al., 2017[120]	Enzyme activity: 0.47.	Grahame G. Kerr DS	Pt#71
c.1121_1159dup, p.Phe386_Lys387ins13	Pt#514	M	DeBrosse S et al., 2012[105]	Enzyme activity: 0.41.		
c.1137_1159dup, p.Lys387MetfsTer45	Pt#76	M	Shin H et al., 2017[120]	Enzyme activity: 0.48.	Grahame G. Kerr DS	Pt#76
c.1137_1159dup, p.Lys387MetfsTer45	Pt#523	M	DeBrosse S et al., 2012[105]	Enzyme activity: 0.48.		

Supplementary table 4 lists cases from the literature that were identified as possible duplicates after each published cases was cross-referenced with other cases of the same genotype, matching sex, and quantitative data (e.g., age at presentation, age at death, residual PDHc enzyme activity), along with overlapping authorship. In supplementary table 4 enzyme activity refers to residual PDHc activity in fibroblasts, unless specified otherwise, results are presented as percentages (range 0-100%) or absolute values (measurement units not provided). DNV – confirmed *de novo* inheritance. F – female. M – male. NA – not available.

Supplementary table 5. **Pathogenicity interpretation details of *PDHA1* variants considered to include in the study using ACMG criteria**

**Variant**	**ACMG**	**Points**	**PVS1**	**PS1**	**PS2**	**PS3**	**PS4**	**PS5**	**PM1**	**PM2**	**PM3**	**PM4**	**PM5**	**PM6**	**PM7**	**PP1**	**PP2**	**PP3**	**PP4**	**PP5**	**PP6**	**BA1**	**BS1**	**BS2**	**BS3**	**BS4**	**BS5**	**BP1**	**BP2**	**BP3**	**BP4**	**BP5**	**BP6**	**BP7**	**BP8**
c.12_13insACTT, p.Leu5ThrfsTer26	P	10	8							2																									
c.29G>C, p.Arg10Pro	LP	9				4				2					2			1																	
c.122_124del, p.Cys41_Asp42delins Tyr	P	10			4				2	2		2																							
c.131A>G, p.His44Arg	LP	8							2	2					2			1	1																
c.148C>T, p.Pro50Ser	LP	6							2	2								1	1																
c.149C>G, p.Pro50Arg	LP	6							2	2								1	1																
c.193_195delinsCAA, p.Tyr65Gln	P	14			4	4				2		2			2																				
c.194A>C, p.Tyr65Ser	LP	7		4						2								1																	
c.212T>C, p.Val71Ala	LP	9			4					2					2			1																	
c.214C>T, p.Arg72Cys	P	12				4			2	2					2			1		1															
c.224A>C, p.Glu75Ala	P	11				4			2	2					2			1																	
c.224A>G, p.Glu75Gly	LP	6								2			2				1	1																	
c.224A>T, p.Glu75Val	LP	9							2	2			2		2			1																	
c.225G>T, p.Glu75Asp	LP	9							2	2			2		2			1																	
c.249dup, p.Gln84ThrfsTer12	P	20	8		4	4				2					2																				
c.261T>G, p.Ile87Met	P	11				4			2	2					2			1																	
c.262C>T, p.Arg88Cys	P	11				4			2	2					2			1																	

Supplementary table 5. **Pathogenicity interpretation details of *PDHA1* variants considered to include in the study using ACMG criteria *(continued)***

Variant	ACMG	Points	PVS1	PS1	PS2	PS3	PS4	PS5	PM1	PM2	PM3	PM4	PM5	PM6	PM7	PP1	PP2	PP3	PP4	PP5	PP6	BA1	BS1	BS2	BS3	BS4	BS5	BP1	BP2	BP3	BP4	BP5	BP6	BP7	BP8
c.262C>A, p.Arg88Ser	P	13				4			2	2			2		2			1																	
c.265G>A, p.Gly89Ser	LP	7				4				2								1																	
c.269T>C, p.Phe90Ser	LP	9				4				2					2			1																	
c.272G>C, p.Cys91Ser	LP	6							2	2								1	1																
c.291G>A, p.?	LP	6			4					2																									
c.291+5G>A, p.?	LP	6			4					2								1													1				
c.292-2A>G, p.?	P	16	8			4			2						2																				
c.301T>C, p.Cys101Arg	LP	9							2	2			2		2			1																	
c.302G>T, p.Cys101Phe	P	11				4			2	2					2			1																	
c.328delinsAGA, p.Pro110ArgfsTer71	P	14	8		4					2																									
c.329C>A, p.Pro110His	P	10			4				2	2					2																				
c.332C>T, p.Thr111Ile	LP	6							2	2								1	1																
c.335A>G, p.Asp112Gly	LP	7								2					2		1	1	1																
c.337C>G, p.His113Asp	LP	9				4				2					2			1																	
c.355C>T, p.Arg119Trp	LP	7							2	2					2			1																	
c.363C>A, p.His121Gln	P	11			4				2	2					2			1																	
c.364G>A, p.Gly122Ser	LP	7							2	2					2			1																	

Supplementary table 5. **Pathogenicity interpretation details of *PDHA1* variants considered to include in the study using ACMG criteria** *(continued)*

Variant	ACMG	Points	PVS1	PS1	PS2	PS3	PS4	PS5	PM1	PM2	PM3	PM4	PM5	PM6	PM7	PP1	PP2	PP3	PP4	PP5	PP6	BA1	BS1	BS2	BS3	BS4	BS5	BP1	BP2	BP3	BP4	BP5	BP6	BP7	BP8
c.364G>C, p.Gly122Arg	LP	7							2	2			2					1																	
c.379C>T, p.Arg127Trp	LP	7							2	2					2			1																	
c.380G>A, p.Arg127Gln	P	11				4			2	2					2			1																	
c.383G>A, p.Gly128Asp	LP	7							2	2					2			1																	
c.394C>T, p.Arg132Ter	P	14	8		4					2																									
c.406G>A, p.A136T	LB	-3																1						4											
c.407C>T, p.Ala136Val	LP	8							2	2					2			1	1																
c.409G>A, p.Glu137Lys	P	11			4				2	2			2					1																	
c.409G>C, p.Glu137Gln	LP	6							2	2								1	1																
c.410A>G, p.Glu137Gly	LP	7			4					2								1																	
c.412C>T, p.Leu138Phe	P	11			4				2	2					2			1																	
c.416C>G, p.Thr139Arg	LP	6							2	2								1	1																
c.419-17_419-14del, p.?	LP	7				4				2					2																1				
c.419-2A>G, p.?	P	13	8							2					2			1																	
c.421C>G, p.Arg141Gly	LP	7							2	2			2					1																	
c.421C>T, p.Arg141*	P	14	8		4					2																									
c.422G>A, p.Arg141Gln	LP	7							2	2					2			1																	

Supplementary table 5. **Pathogenicity interpretation details of *PDHA1* variants considered to include in the study using ACMG criteria** *(continued)*

Variant	ACMG	Points	PVS1	PS1	PS2	PS3	PS4	PS5	PM1	PM2	PM3	PM4	PM5	PM6	PM7	PP1	PP2	PP3	PP4	PP5	PP6	BA1	BS1	BS2	BS3	BS4	BS5	BP1	BP2	BP3	BP4	BP5	BP6	BP7	BP8
c.422G>T, p.Arg141Leu	P	13			4				2	2			2		2			1																	
c.429_431del, p.Gly144del	LP	8							2	2		2			2																				
c.430G>A, p.Gly144Ser	LP	9							2	2			2		2			1																	
c.430G>C, p.Gly144Arg	LP	9							2	2			2		2			1																	
c.431G>A, p.Gly144Asp	LP	7							2	2					2			1																	
c.433_435del, p.Cys145del	P	10			4				2	2		2																							
c.434G>A, p.Cys145Tyr	LP	9			4				2	2								1																	
c.442G>A, p.Gly148Arg	P	11				4			2	2					2			1																	
c.449G>A, p.Gly150Glu	P	11			4				2	2					2			1																	
c.451G>A, p.Gly151Arg	LP	9			4				2	2								1																	
c.454T>C, p.Ser152Pro	LP	6							2	2								1	1																
c.454T>A, p.Ser152Thr	LP	9			4					2					2			1																	
c.455C>T, p.Ser152Leu	LP	7							2	2					2			1																	
c.457A>G, p.Met153Val	P	11			4				2	2					2			1																	
c.464T>C, p.Met155Thr	P	11			4				2	2					2			1																	

Supplementary table 5. **Pathogenicity interpretation details of *PDHA1* variants considered to include in the study using ACMG criteria** *(continued)*

Variant	ACMG	Points	PVS1	PS1	PS2	PS3	PS4	PS5	PM1	PM2	PM3	PM4	PM5	PM6	PM7	PP1	PP2	PP3	PP4	PP5	PP6	BA1	BS1	BS2	BS3	BS4	BS5	BP1	BP2	BP3	BP4	BP5	BP6	BP7	BP8
c.465G>T, p.Met155Ile	LP	7							2	2			2					1																	
c.472A>G, p.K158E	VUS	5							2	2									1																
c.478_479delTTinsAA, p.Phe160Asn	LP	8					4		2	2																									
c.479_481del, p.Phe160del	P	10		4	4					2																									
c.481_483del, p.Phe160del	LP	8							2	2		2			2																				
c.482A>G, p.Tyr161Cys	LP	7							2	2					2			1																	
c.483C>T, p.?	LP	8				4				2					2			1													1				
c.484G>A, p.Gly162Arg	P	11		4					2	2					2			1																	
c.491A>G, p.Asn164Ser	LP	7							2	2					2			1																	
c.495C>T, p.?	LP	9			4					2					2				1																
c.498C>T, p.?	P	11			4	4				2					2			1													1			1	
c.499G>A, p.Val167Met	LP	7							2	2					2			1																	
c.499G>T, p.Val167Leu	LP	7				4				2								1																	
c.506C>T, p.Ala169Val	LP	8							2	2					2			1		1															
c.511G>A, p.Val171Met	LP	7							2	2					2			1																	
c.511-30G>A, p.?	P	12			4	4				2					2			1													1				
c.511G>C, p.Val171Leu	P	11			4				2	2			2					1																	
c.511-414_899+584del, p.?	LP	8	8							2		2													4										

Supplementary table 5. **Pathogenicity interpretation details of *PDHA1* variants considered to include in the study using ACMG criteria** *(continued)*

**Variant**	**ACMG**	**Points**	**PVS1**	**PS1**	**PS2**	**PS3**	**PS4**	**PS5**	**PM1**	**PM2**	**PM3**	**PM4**	**PM5**	**PM6**	**PM7**	**PP1**	**PP2**	**PP3**	**PP4**	**PP5**	**PP6**	**BA1**	**BS1**	**BS2**	**BS3**	**BS4**	**BS5**	**BP1**	**BP2**	**BP3**	**BP4**	**BP5**	**BP6**	**BP7**	**BP8**
c.513_759+2del, p.?	P	18	8			4				2		2			2																				
c.515C>T, p.Pro172Leu	LP	9			4				2	2								1																	
c.523G>A, p.Ala175Thr	LP	7							2	2					2			1																	
c.523G>C, p.Ala175Pro	LP	7							2	2					2			1																	
c.530T>C, p.Ile177Thr	P	11				4			2	2					2			1																	
c.535C>G, p.Leu179Val	LP	8							2	2					2			1	1																
c.536T>G, p.Leu179Arg	LP	6							2	2								1	1																
c.542G>A, p.Cys181Tyr	P	11			4				2	2					2			1																	
c.548A>G, p.Tyr183Cys	LP	7							2	2					2			1																	
c.555A>G, p.?	LP	9				4				2					2					1															
c.562_858dup, p.Glu188_Thr286dup	P	20	8			4			2	2		2			2																				
c.562G>A, p.Glu188Lys	P	13			4	4				2					2			1																	
c.584G>C, p.Gly195Ala	P	13			4	4				2					2			1																	
c.586G>A, p.Asp196Asn	LP	7							2	2			2					1																	
c.592G>A, p.Ala198Thr	P	19	8			4			2	2					2			1																	
c.593C>T, p.Ala198Val	LP	9				4				2					2			1																	

Supplementary table 5. **Pathogenicity interpretation details of *PDHA1* variants considered to include in the study using ACMG criteria *(continued)***

Variant	ACMG	Points	PVS1	PS1	PS2	PS3	PS4	PS5	PM1	PM2	PM3	PM4	PM5	PM6	PM7	PP1	PP2	PP3	PP4	PP5	PP6	BA1	BS1	BS2	BS3	BS4	BS5	BP1	BP2	BP3	BP4	BP5	BP6	BP7	BP8
c.595G>A, p.Ala199Thr	P	14			4	4				2					2			1		1															
c.599A>C, p.Asn200Thr	LP	9				4				2					2			1																	
c.604-10C>G, p.?	LP	9				4				2					2			1																	
c.606_609del, p.Gln203TyrfsTer49	P	16	8			4				2					2																				
c.613T>C, p.Phe205Leu	LP	9		4					2	2								1																	
c.615C>G, p.Phe205Leu	P	11				4			2	2					2			1																	
c.615C>A, p.Phe205Leu	P	15		4		4			2	2					2			1																	
c.616G>A, p.Glu206Lys	P	11				4			2	2					2			1																	
c.616G>T, p.Glu206Ter	P	12	8						2	2																									
c.616G>C, p.Glu206Gln	P	11			4				2	2			2					1																	
c.619G>C, p.Ala207Pro	LP	6							2	2								1	1																
c.626A>G, p.Asn209Ser	LP	6							2	2								1	1																
c.628A>G, p.Met210Val	P	11				4			2	2					2			1																	
c.629T>C, p.Met210Thr	P	17		4		4			2	2			2		2			1																	
c.640T>C, p.Trp214Arg	P	11				4			2	2					2			1																	
c.640T>G, p.Trp214Gly	P	11		4					2	2			2					1																	

Supplementary table 5. **Pathogenicity interpretation details of *PDHA1* variants considered to include in the study using ACMG criteria** *(continued)*

Variant	ACMG	Points	PVS1	PS1	PS2	PS3	PS4	PS5	PM1	PM2	PM3	PM4	PM5	PM6	PM7	PP1	PP2	PP3	PP4	PP5	PP6	BA1	BS1	BS2	BS3	BS4	BS5	BP1	BP2	BP3	BP4	BP5	BP6	BP7	BP8
c.642G>T, p.Trp214Cys	P	11				4			2	2					2			1																	
c.643A>C, p.Lys215Gln	LP	6								2					2			1	1																
c.647T>C, p.Leu216Ser	P	11				4			2	2					2			1																	
c.648A>C, p.Leu216Phe	LP	9				4			2	2								1																	
c.649C>A, p.Pro217Thr	LP	7							2	2			2					1																	
c.649C>G, p.Pro217Ala	LP	7								2			2		2			1																	
c.650C>T, p.Pro217Leu	P	11				4			2	2					2			1																	
c.650C>G, p.Pro217Arg	P	13			4	4			2	2								1																	
c.666_667del, p.Cys222Ter	P	14	8		4					2																									
c.677G>A, p.Arg226His	LP	9				4			2	2								1																	
c.679T>C, p.Tyr227His	LP	9			4				2	2								1																	
c.680A>G, p.Tyr227Cys	LP	7							2	2			2					1																	
c.687G>A, p.Met229Ile	P	13		4		4			2	2								1																	
c.688G>A, p.Gly230Arg	LP	6							2	2								1	1																
c.691A>G, p.Thr231Ala	P	11				4			2	2					2			1																	

Supplementary table 5. **Pathogenicity interpretation details of *PDHA1* variants considered to include in the study using ACMG criteria** *(continued)*

Variant	ACMG	Points	PVS1	PS1	PS2	PS3	PS4	PS5	PM1	PM2	PM3	PM4	PM5	PM6	PM7	PP1	PP2	PP3	PP4	PP5	PP6	BA1	BS1	BS2	BS3	BS4	BS5	BP1	BP2	BP3	BP4	BP5	BP6	BP7	BP8
c.692C>G, p.Thr231Arg	LP	9							2	2			2		2			1																	
c.692C>A, p.Thr231Lys	LP	6							2	2								1	1																
c.703_706del, p.Ala236GlnfsTer16	P	10	8							2																									
c.703A>G, p.Arg235Gly	P	15			4	4			2	2					2			1																	
c.705_706del, p.Arg235SerfsTer6	P	10	8							2																									
c.707C>A, p.Ala236Glu	P	11				4			2	2					2			1																	
c.721_724dup, p.Tyr242Ter	P	12	8							2					2																				
c.727_729del, p.Tyr243del	P	14			4	4				2		2			2																				
c.727T>A, p.Tyr243Asn	P	13				4			2	2			2		2			1																	
c.728A>G, p.Tyr243Cys	P	11				4			2	2					2			1																	
c.728A>C, p.Tyr243Ser	P	13				4			2	2			2		2			1																	
c.729C>A, p.Tyr243Ter	P	16	8			4				2					2																				
c.730_731del, p.Lys244GlufsTer30	P	14	8		4					2																									
c.733A>G, p.Arg245Gly	P	13				4			2	2			2		2			1																	
c.738C>T, p.?	P	10				4				2					2					1	1														
c.748C>A, p.Pro250Thr	P	11				4			2	2					2			1																	

Supplementary table 5. **Pathogenicity interpretation details of *PDHA1* variants considered to include in the study using ACMG criteria** *(continued)*

Variant	ACMG	Points	PVS1	PS1	PS2	PS3	PS4	PS5	PM1	PM2	PM3	PM4	PM5	PM6	PM7	PP1	PP2	PP3	PP4	PP5	PP6	BA1	BS1	BS2	BS3	BS4	BS5	BP1	BP2	BP3	BP4	BP5	BP6	BP7	BP8
c.749C>T, p.Pro250Leu	P	13				4			2	2			2		2			1																	
c.754C>G, p.Leu252Val	LP	8				4			2	2								1													1				
c.757A>G, p.Arg253Gly	P	11				4			2	2					2			1																	
c.759+26G>A, p.?	P	11			4	4				2					2																1				
c.762_831+2del, p.?	LP	9				4				2					2			1																	
c.760-2A>G, p.?	P	16	8			4				2					2																				
c.773A>C, p.Asp258Ala	P	11				4			2	2					2			1																	
c.778C>G, p.Leu260Val	P	11				4			2	2					2			1																	
c.784G>C, p.Val262Leu	LP	7							2	2			2					1																	
c.784G>T, p.Val262Phe	P	15			4	4			2	2					2			1																	
c.787C>G, p.Arg263Gly	P	11				4			2	2					2			1																	
c.787C>T, p.Arg263Ter	P	16	8			4				2					2																				
c.788G>A, p.Arg263Gln	LP	9							2	2			2		2			1																	
c.788G>C, p.Arg263Pro	P	13				4			2	2			2		2			1																	
c.821G>C, p.Arg274Thr	LP	7				4				2								1																	
c.831+1G>A, p.?	P	16	8			4				2					2																				
c.832G>A, p.Gly278Arg	LP	6								2			2					1	1																

Supplementary table 5. **Pathogenicity interpretation details of *PDHA1* variants considered to include in the study using ACMG criteria** *(continued)*

Variant	ACMG	Points	PVS1	PS1	PS2	PS3	PS4	PS5	PM1	PM2	PM3	PM4	PM5	PM6	PM7	PP1	PP2	PP3	PP4	PP5	PP6	BA1	BS1	BS2	BS3	BS4	BS5	BP1	BP2	BP3	BP4	BP5	BP6	BP7	BP8
c.832G>C, p.Gly278Arg	LP	9		4						2					2			1																	
c.833G>A, p.Gly278Glu	LP	6								2			2					1	1																
c.836C>T, p.P279L	VUS	4								2								1	1																
c.839T>G, p.Ile280Ser	LP	6							2	2								1		1															
c.844A>C, p.M282L	B	-14																				8			4						1		1		
c.844A>G, p.Met282Val	P	11				4			2	2					2			1																	
c.845_846insTCT, p.Met282delinsIleLeu	LP	6							2	2		2																							
c.845T>G, p.Met282Arg	P	11			4				2	2			2					1																	
c.847G>A, p.Glu283Lys	P	11				4			2	2					2			1																	
c.853C>T, p.Gln285Ter	P	16	8			4				2					2																				
c.853_865dup, p.Tyr289SerfsTer12	P	10	8							2																									
c.858_861dup, p.Arg288LeufsTer10	P	16	8		4					2					2																				
c.861_862insT, p.Arg288SerfsTer9	P	10	8							2																									
c.862C>T, p.Arg288Cys	P	11				4			2	2					2			1																	
c.862C>A, p.Arg288Ser	P	11			4				2	2			2					1																	
c.863G>A, p.Arg288His	P	13				4			2	2			2		2			1																	

Supplementary table 5. **Pathogenicity interpretation details of *PDHA1* variants considered to include in the study using ACMG criteria** *(continued)*

Variant	ACMG	Points	PVS1	PS1	PS2	PS3	PS4	PS5	PM1	PM2	PM3	PM4	PM5	PM6	PM7	PP1	PP2	PP3	PP4	PP5	PP6	BA1	BS1	BS2	BS3	BS4	BS5	BP1	BP2	BP3	BP4	BP5	BP6	BP7	BP8
c.863G>T, p.Arg288Leu	LP	7							2	2			2					1																	
c.868C>T, p.His290Tyr	P	11				4			2	2					2			1																	
c.869A>C, p.His290Pro	LP	9			4				2	2								1																	
c.871G>A, p.Gly291Arg	LP	7							2	2					2			1																	
c.875A>T, p.His292Leu	P	11				4			2	2					2			1																	
c.883A>G, p.Ser295Gly	P	11				4			2	2					2			1																	
c.886G>A, p.Asp296Asn	P	11			4				2	2			2					1																	
c.888C>G, p.Asp296Glu	P	11				4			2	2					2			1																	
c.890dup, p.Gly298TrpfsTer16	P	16	8			4				2					2																				
c.892G>A, p.Gly298Arg	LP	9				4				2			2					1																	
c.893G>A, p.Gly298Glu	P	13			4	4				2					2			1																	
c.894_899+1dup, p.?	P	16	8			4				2					2																				
c.899+1G>C, p.?	P	16	8			4				2					2																				
c.899_918del, p.Ser300AsnfsTer7	P	16	8			4				2					2																				
c.899+3_988del, p.?	LP	7			4					2								1																	
c.900-3_922dup, p.?	LP	8				4				2					2																				
c.900-1_903dup, p.?	P	16	8			4				2					2																				
c.900_903dup, p.Arg302LeufsTer13	P	10	8		4					2															4										

Supplementary table 5. **Pathogenicity interpretation details of *PDHA1* variants considered to include in the study using ACMG criteria** *(continued)*

Variant	ACMG	Points	PVS1	PS1	PS2	PS3	PS4	PS5	PM1	PM2	PM3	PM4	PM5	PM6	PM7	PP1	PP2	PP3	PP4	PP5	PP6	BA1	BS1	BS2	BS3	BS4	BS5	BP1	BP2	BP3	BP4	BP5	BP6	BP7	BP8
c.900_932dup, p.?	LP	9				4				2					2			1																	
c.900-16_905dup, p.?	LP	9				4				2					2			1																	
c.900-1G>A, p.?	P	16	8			4				2					2																				
c.900-41_900-23del, p.?	LP	9				4				2					2			1																	
c.900-6_958dup, p.?	LP	8				4				2					2																				
c.900-3_917dup, p.?	LP	9				4				2					2			1																	
c.900-12_920dup, p.?	LP	9				4				2					2			1																	
c.901_1004dup, p.Lys336ThrfsTer10	P	16	8			4				2					2																				
c.904C>T, p.Arg302Cys	P	10				4				2					2		1	1																	
c.905G>A, p.Arg302His	P	11				4			2	2					2			1																	
c.905G>T, p.Arg302Leu	P	13				4			2	2			2		2			1																	
c.905_927inv, p.Arg302_Glu309delinsLeuProGluPheLeuLeuValTyr	LP	8				4				2					2																				
c.910C>T, p.Arg304Ter	P	16	8			4				2					2																				
c.914_915insGATAGTTACCGTACACGAGAA, p.Arg304_Glu305insAspSerTyrArgThrArgGlu	LP	8				4				2					2																				
c.913_929dup, p.Arg311LysfsTer6	P	14	8			4				2																									

Supplementary table 5. **Pathogenicity interpretation details of *PDHA1* variants considered to include in the study using ACMG criteria** *(continued)*

Variant	ACMG	Points	PVS1	PS1	PS2	PS3	PS4	PS5	PM1	PM2	PM3	PM4	PM5	PM6	PM7	PP1	PP2	PP3	PP4	PP5	PP6	BA1	BS1	BS2	BS3	BS4	BS5	BP1	BP2	BP3	BP4	BP5	BP6	BP7	BP8
c.917_924dup, p.Glu309LysfsTer5	P	14	8		4					2																									
c.924_930del, p.Ser312ValfsTer12	P	20	8	4		4				2					2																				
c.924G>T, p.Gln308His	LP	9				4				2					2			1																	
c.927dup, p.Val310SerfsTer4	P	16	8		4					2					2																				
c.929_932del, p.Val310GlufsTer15	P	16	8			4				2					2																				
c.931A>G, p.Arg311Gly	LP	7							2	2					2			1																	
c.932_935del, p.Arg311IlefsTer14	P	16	8			4				2					2																				
c.933_935del, p.Arg311del	P	12				4			2	2		2			2																				
c.933_989dup, p.Lys313_Ser331dup	LP	7			4					2								1																	
c.934_940del, p.Ser312ValfsTer12	P	16	8			4				2					2																				
c.934_992dup, p.Ser331ArgfsTer15	P	10	8							2																									
c.936_939del, p.Ser312ArgfsTer13	P	14	8			4				2																									
c.937_940dup, p.Ser314LysfsTer3	P	10	8							2																									
c.937_942dup, p.Lys313_Ser314dup	P	16			4	4			2	2		2			2																				
c.938_940del, p.Lys313del	P	13				4			2	2		2			2					1															

Supplementary table 5. **Pathogenicity interpretation details of *PDHA1* variants considered to include in the study using ACMG criteria** *(continued)*

Variant	ACMG	Points	PVS1	PS1	PS2	PS3	PS4	PS5	PM1	PM2	PM3	PM4	PM5	PM6	PM7	PP1	PP2	PP3	PP4	PP5	PP6	BA1	BS1	BS2	BS3	BS4	BS5	BP1	BP2	BP3	BP4	BP5	BP6	BP7	BP8
c.939_950del, p.Lys313_Ile317delins Asn	P	10			4				2	2		2																							
c.940A>T, p.Ser314Cys	LP	7							2	2					2			1																	
c.943G>A, p.Asp315Asn	P	11				4			2	2					2			1																	
c.947C>T, p.Pro316Leu	P	11				4			2	2					2			1																	
c.947dup, p.Ile317TyrfsTer23	P	14	8			4				2																									
c.948_963dup, p.Asp322TyrfsTer23	P	16	8			4				2					2																				
c.949_952dup, p.Met318AsnfsTer23	P	14	8			4				2																									
c.949_950del, p.Ile317TyrfsTer22	P	16	8			4				2					2																				
c.950_962dup, p.Lys321AsnfsTer23	P	16	8			4				2					2																				
c.957_959dup, p.Leu320dup	P	10			4				2	2		2																							
c.960_1008+5dup, p.?	P	13			4	4				2					2			1																	
c.963_977dup, p.Lys321_Val325dup	P	14		4		4			2	2		2																							
c.966_1011dup, p.Ile338_Ser390delins GlnAspGlyGluGlnGln SerCysGlnCysGlyArg ThrLysGlyThrValThr CysSerTrpTrpPheGlu GlyTrpLeu	P	10	8							2																									

Supplementary table 5. **Pathogenicity interpretation details of *PDHA1* variants considered to include in the study using ACMG criteria *(continued)***

Variant	ACMG	Points	PVS1	PS1	PS2	PS3	PS4	PS5	PM1	PM2	PM3	PM4	PM5	PM6	PM7	PP1	PP2	PP3	PP4	PP5	PP6	BA1	BS1	BS2	BS3	BS4	BS5	BP1	BP2	BP3	BP4	BP5	BP6	BP7	BP8
c.966_969del, p.Asp322GlufsTer3	P	16	8			4				2					2																				
c.968_980dup, p.Ser327ArgfsTer17	P	16	8			4				2					2																				
c.968_976del, p.Arg323_Val325del	LP	9			4					2		2						1																	
c.969_1004dup, p.Met324_Leu335dup	LP	7							2	2		2						1																	
c.978_1004dup, p.Ser327_Leu335dup	LP	7							2	2		2						1																	
c.982_985dup, p.Leu329GlnfsTer12	P	16	8			4				2					2																				
c.983_986dup, p.Ala330SerfsTer11	P	16	8			4				2					2																				
c.985_998dup, p.Glu333AspfsTer8	P	20	8		4	4				2					2																				
c.986_998dup, p.Glu333AspfsTer11	P	16	8			4				2					2																				
c.986T>G, p.Leu329Arg	LP	9				4				2					2			1																	
c.989C>G, p.Ala330Gly	LP	6								2					2			1			1														
c.1006_1008dup, p.Lys336dup	P	10			4					2		2			2																				
c.1008+1_1008+27del, p.?	P	14	8		4					2																									
c.1011_1031dup, p.Ile338_Lys344dup	P	12			4				2	2		2			2																				
c.1011_1040dup, p.Ile338_Glu347dup	LP	6							2	2		2																							

Supplementary table 5. **Pathogenicity interpretation details of *PDHA1* variants considered to include in the study using ACMG criteria** *(continued)*

Variant	ACMG	Points	PVS1	PS1	PS2	PS3	PS4	PS5	PM1	PM2	PM3	PM4	PM5	PM6	PM7	PP1	PP2	PP3	PP4	PP5	PP6	BA1	BS1	BS2	BS3	BS4	BS5	BP1	BP2	BP3	BP4	BP5	BP6	BP7	BP8
c.1014_1032dup, p.Glu345Ter	P	12	8							2					2																				
c.1022_1034del, p.Glu341GlyfsTer79	P	14	8		4					2																									
c.1026_1051dup, p.Gln351ArgfsTer82	P	12	8							2					2																				
c.1026_1130dup, p.Lys344_Arg378dup	P	10			4				2	2		2																							
c.1033_1078dup, p.Pro360ArgfsTer3	P	10	8							2																									
c.1034_1037dup, p.Glu347AspfsTer2	P	14	8		4					2																									
c.1040_1046dup, p.Ala350GlyfsTer11	P	10	8							2																									
c.1040_1063dup, p.Glu347_Thr354dup	LP	8							2	2		2			2																				
c.1042_1045dup, p.Ala349GlyfsTer11	P	12	8							2					2																				
c.1045G>A, p.Ala349Thr	LP	7							2	2					2			1																	
c.1046_1047insCAAT, p.Arg349SerfsTer4	P	14	8		4					2																									
c.1050_1133dup, p.Gln351_Arg378dup	P	12			4				2	2		2			2																				
c.1051C>T, p.Gln351Ter	P	12	8							2					2																				
c.1052A>C, p.Gln351Pro	LP	9			4				2	2								1																	
c.1054_1057del, p.Phe352ProfsTer71	P	10	8							2																									

Supplementary table 5. **Pathogenicity interpretation details of *PDHA1* variants considered to include in the study using ACMG criteria *(continued)***

Variant	ACMG	Points	PVS1	PS1	PS2	PS3	PS4	PS5	PM1	PM2	PM3	PM4	PM5	PM6	PM7	PP1	PP2	PP3	PP4	PP5	PP6	BA1	BS1	BS2	BS3	BS4	BS5	BP1	BP2	BP3	BP4	BP5	BP6	BP7	BP8
c.1057G>A, p.Ala353Thr	LP	6							2	2								1	1																
c.1057G>C, p.Ala353Pro	LP	7							2	2					2			1																	
c.1062_1124dup, p.Pro374_Phe375insLeuAlaAspProGluProProLeuGluGluLeuGlyTyrHisIleTyrSerSerAspProPro	P	12			4				2	2		2			2																				
c.1063_1068del, p.Ala355_Asp356del	LP	8							2	2		2			2																				
c.1064_1065insTAAG, p.Asp356LysfsTer4	P	10	8							2																									
c.1065del, p.Asp356IlefsTer68	P	14	8		4					2																									
c.1066_1090dup, p.Leu364delinsArgSerTer	P	14	8		4					2																									
c.1069_1114dup, p.Asp372delinsAlaTer	P	12	8							2					2																				
c.1071_1088dup, p.Glu362_Glu363insAspGluProProLeuGlu	LP	6							2	2		2																							
c.1073_1092del, p.Glu358GlyfsTer12	P	16	8		4					2					2																				
c.1072G>A, p.Glu358Lys	LP	8							2	2					2			1	1																
c.1073_1094del, p.Glu358AlafsTer59	P	14	8		4					2																									

Supplementary table 5. **Pathogenicity interpretation details of *PDHA1* variants considered to include in the study using ACMG criteria** *(continued)*

**Variant**	**ACMG**	**Points**	**PVS1**	**PS1**	**PS2**	**PS3**	**PS4**	**PS5**	**PM1**	**PM2**	**PM3**	**PM4**	**PM5**	**PM6**	**PM7**	**PP1**	**PP2**	**PP3**	**PP4**	**PP5**	**PP6**	**BA1**	**BS1**	**BS2**	**BS3**	**BS4**	**BS5**	**BP1**	**BP2**	**BP3**	**BP4**	**BP5**	**BP6**	**BP7**	**BP8**
c.1081_1125dup, p.Leu361_Phe375dup	P	10			4				2	2		2																							
c.1083_1102dup, p.Ile368ArgfsTer63	P	12	8							2					2																				
c.1083_1124del, p.Leu361_Pro374del	LP	6							2	2		2																							
c.1083_1124dup, p.Leu361_Pro374dup	LP	6							2	2		2																							
c.1085_1093dup, p.Glu362_Leu364dup	P	10			4				2	2		2																							
c.1087_1119dup, p.Glu363_Pro373dup	P	12			4				2	2		2			2																				
c.1090_1155dup, p.Leu364_Lys385dup 22	P	10			4				2	2		2																							
c.1091T>G, p.Leu364Arg	LP	6		4						2																									
c.1093_1112dup, p.Ser371ArgfsTer60	P	16	8			4				2					2																				
c.1095_1118dup, p.Tyr366_Pro373dup	LP	6							2	2		2																							
c.1100A>T, p.His367Leu	P	11				4			2	2					2			1																	
c.1100A>G, p.His367Arg	LP	6							2	2					2																				
c.1101_1154dup, p.Ile384_Lys385insAsnIleTyrSerSerAspProProPheGluValArgGlyAlaAsnGlnTrpIle	LP	8							2	2		2			2																				

Supplementary table 5. **Pathogenicity interpretation details of *PDHA1* variants considered to include in the study using ACMG criteria** *(continued)*

Variant	ACMG	Points	PVS1	PS1	PS2	PS3	PS4	PS5	PM1	PM2	PM3	PM4	PM5	PM6	PM7	PP1	PP2	PP3	PP4	PP5	PP6	BA1	BS1	BS2	BS3	BS4	BS5	BP1	BP2	BP3	BP4	BP5	BP6	BP7	BP8
c.1103_1116dup, p.Pro373SerfsTer56	P	12	8							2					2																				
c.1106_1108del, p.Tyr369del	P	12			4				2	2		2			2																				
c.1105T>C, p.Tyr369His	LP	9			4				2	2								1																	
c.1116_1154dup, p.Ile384_Lys385insAsnProProPheGluValArgGlyAlaAsnGlnTrpIle	LP	8							2	2		2			2																				
c.1119_1123del, p.Pro374_Phe375delinsTer	P	12	8							2					2																				
c.1121_1159dup, p.Phe386_Lys387ins13	LP	8							2	2		2			2																				
c.1121_1144dup, p.Pro374_Asn381dup	P	12			4				2	2		2			2																				
c.1124_1125dup, p.Glu376LeufsTer49	P	12	8							2					2																				
c.1124_1132del, p.Phe375_Arg378delinsCys	P	10			4				2	2		2																							
c.1125del, p.Phe375LeufsTer49	P	14	8		4					2																									
c.1126_1131dup, p.Glu376_Val377dup	LP	6							2	2		2																							
c.1132C>T, p.Arg378Cys	P	14			4		4		2	2								1		1															
c.1133G>A, p.Arg378His	P	15			4		4		2	2					2			1																	

c.1133G>T, p.Arg378Leu	LP	9							2	2			2		2			1																	
c.1134_1159dup, p.Lys387MetfsTer46	P	10	8							2																									
c.1137_1159dup, p.Lys387MetfsTer45	P	12	8							2					2																				
c.1138_1157dup, p.Phe386LeufsTer45	P	12	8							2					2																				
c.1139_1142dup, p.Trp383SerfsTer5	P	12	8							2					2																				
c.1140_1150dup, p.Ile384ThrfsTer44	P	10	8							2																									
c.1142_1145dup, p.Trp383SerfsTer6	P	17	8		4					2					2					1															
c.1142_1164del, p.Asn381SerfsTer43	P	12	8							2					2																				
c.1143_1154del, p.Asn381_Ile384del	P	12			4				2	2		2			2																				
c.1144_1159dup, p.Lys387ThrfsTer6	P	16	8		4					2					2																				
c.1144_1147dup, p.Trp383SerfsTer6	P	18	8	4	4					2																									
c.1144C>T, p.Gln382Ter	P	14	8		4					2																									
c.1147_1158dup, p.Trp383_Phe386dup	P	10			4				2	2		2																							
c.1147T>C, p.Trp383Arg	P	11				4			2	2					2			1																	
c.1148G>A, p.Trp383Ter	P	20	8		4	4				2					2																				
c.1153_1155dup, p.Lys385dup	LP	8							2	2		2			2																				
c.1157_1162del, p.Phe386_Lys387del	P	16			4	4			2	2		2			2																				
c.1157_1158dup, p.Lys387LeufsTer38	P	12	8							2					2																				
c.1157_1162dup, p.Phe386_Lys387dup	P	11			4				2	2		2								1															

c.1158dup, p.Lys387Ter	P	12	8							2					2																				
c.1159_1162dup, p.Ser388Ter	P	12	8							2					2																				
c.1159_1160del, p.Lys387ValfsTer44	P	12	8							2					2																				
c.1162T>C, p.Ser388Pro	LP	6							2	2									1		1														
c.1163_1164del, p.Ser388CysfsTer43	P	16	8		4					2					2																				
c.1163C>A, p.Ser388Ter	P	12	8							2					2																				
c.1167_1170del, p.Ser390LysfsTer33	P	12	8							2					2																				

ACMG – variant pathogenicity interpretation according to the recommendations of the American College of Medical Genetics and Genomics and the Association for Molecular Pathology (ACMG/AMP).[146] Variant interpretation was done using naturally scaled point system to the ACMG/AMP variant classification guidelines.[147] Two authors independently evaluated the pathogenicity of each variant, all discrepancies were resolved unanimously. Score is the sum of all points given for specific criteria based on evidence strength. Point values used for different categories of evidence strength: a) indeterminate = 0, b) supporting = 1 or -1, c) moderate = 2 or -2, d) strong = 4 or -4, e) very strong = 8 or -8. Negative values were given for benign evidence. Point based variant classification categories: a.) Pathogenic (P) ≥ 10, b) Likely Pathogenic (LP) ranged 6 – 9, c) Variant of unknown significance (VUS) ranged 0 – 5, d) Likely Benign (LB) ranged −1 – −6, e) Benign (B) ≤ −7. Criteria for classifying pathogenic variants (PVS1, PS1, PS2, PS3, PS4, PS5, PM1, PM2, PM3, PM4, PM5, PM6, PM7, PP1, PP2, PP3, PP4, PP5, PP6) and benign variants (BA1, BS1, BS2, BS3, BS4, BS5, BP1, BP2, BP3, BP4, BP5, BP6, BP7, BP8) are defined according to 2015 ACMG/AMP guidelines.[146] VUS, LB and B variants are marked in red and were excluded from the cohort.

Supplementary Table 6. ***PDHA1* variants included in the study in decreasing frequency**

Variant	Classification	No. of cases (M/F/NA)	Case ID	DNV	INH	UP	P	References
c.787C>G, p.Arg263Gly	Missense	68 (53/10/5)	Pt#13, Pt#17, Pt#18, Pt#34, Pt#38, Pt#53, Pt#59, Pt#67, Pt#86, Pt#110, Pt#147, Pt#148, Pt#155, Pt#187, Pt#188, Pt#207, Pt#238, Pt#239, Pt#240, Pt#273, Pt#274, Pt#302, Pt#303, Pt#322, Pt#326, Pt#328, Pt#331, Pt#334, Pt#359, Pt#366, Pt#420, Pt#444, Pt#461, Pt#492, Pt#493, Pt#539, Pt#545, Pt#549, Pt#566, Pt#601, Pt#605, Pt#606, Pt#607, Pt#609, Pt#648, Pt#653, Pt#683, Pt#690, Pt#699, Pt#718, Pt#755, Pt#762, Pt#764, Pt#774, Pt#797, Pt#845, Pt#886, Pt#895, Pt#897, Pt#908, Pt#916, Pt#966, Pt#995, Pt#996, Pt#997, Pt#998, Pt#1013, Pt#1024	17	14	27	41	22,22,39,63,65,71,74,76,77,81,84,94,97,99,101,105,106,110,113,119,120,122,137
c.904C>T, p.Arg302Cys	Missense	40 (2/36/2)	Pt#52, Pt#93, Pt#109, Pt#116, Pt#152, Pt#191, Pt#192, Pt#210, Pt#211, Pt#215, Pt#217, Pt#248, Pt#250, Pt#254, Pt#282, Pt#352, Pt#360, Pt#401, Pt#423, Pt#456, Pt#502, Pt#503, Pt#504, Pt#505, Pt#555, Pt#685, Pt#723, Pt#778, Pt#788, Pt#875, Pt#888, Pt#914, Pt#917, Pt#944, Pt#946, Pt#981, Pt#1010, Pt#1017, Pt#1020, Pt#1032	11	3	12	28	12,64,73,74,79,81,86,99,101,106,108,115,120,130,131
c.1133G>A, p.Arg378His	Missense	35 (24/10/1)	Pt#29, Pt#36, Pt#60, Pt#62, Pt#70, Pt#79, Pt#82, Pt#182, Pt#183, Pt#220, Pt#259, Pt#298, Pt#304, Pt#306, Pt#307, Pt#310, Pt#381, Pt#389, Pt#519, Pt#533, Pt#577, Pt#612, Pt#619, Pt#629, Pt#429, Pt#650, Pt#697, Pt#738, Pt#739, Pt#740, Pt#802, Pt#876, Pt#904, Pt#956, Pt#976	17	1	14	21	31,62,69,31,71,77,80,85,88,99,105,120,150
c.491A>G, p.Asn164Ser	Missense	33 (26/6/1)	Pt#69, Pt#85, Pt#87, Pt#393, Pt#413, Pt#414, Pt#466, Pt#482, Pt#542, Pt#573, Pt#576, Pt#584, Pt#587, Pt#588, Pt#590, Pt#591, Pt#617, Pt#657, Pt#662, Pt#715, Pt#716, Pt#717, Pt#770, Pt#798, Pt#860, Pt#873, Pt#894, Pt#903, Pt#923, Pt#953, Pt#968, Pt#984, Pt#1031	9	6	25	8	81,82,86,105,120

Supplementary Table 6. ***PDHA1* variants included in the study in decreasing frequency** *(continued)*

Variant	Classification	No. of cases (M/F/NA)	Case ID	DNV	INH	UP	P	References
c.1132C>T, p.Arg378Cys	Missense	30 (19/8/3)	Pt#6, Pt#51, Pt#58, Pt#77, Pt#90, Pt#114, Pt#145, Pt#269, Pt#296, Pt#312, Pt#340, Pt#362, Pt#363, Pt#382, Pt#427, Pt#530, Pt#531, Pt#532, Pt#578, Pt#595, Pt#639, Pt#806, Pt#807, Pt#905, Pt#936, Pt#974, Pt#999, Pt#1000, Pt#1001, Pt#1015	14	–	8	22	47,73,81,88,97,99,103,105,111,119,120,127,136,137,150,151
c.1142_1145dup, p.Trp383SerfsTer6	Frameshift	30 (1/28/1)	Pt#5, Pt#101, Pt#167, Pt#169, Pt#202, Pt#226, Pt#227, Pt#247, Pt#354, Pt#355, Pt#356, Pt#438, Pt#439, Pt#524, Pt#525, Pt#565, Pt#678, Pt#693, Pt#745, Pt#786, Pt#812, Pt#814, Pt#819, Pt#915, Pt#929, Pt#938, Pt#945, Pt#960, Pt#970, Pt#971	13	1	15	15	5,6,10,65,81,97,105,111,115,120,138,139
c.934_940del, p.Ser312ValfsTer12	Frameshift	25 (0/24/1)	Pt#20, Pt#26, Pt#27, Pt#50, Pt#107, Pt#163, Pt#203, Pt#243, Pt#257, Pt#353, Pt#435, Pt#468, Pt#469, Pt#529, Pt#569, Pt#615, Pt#742, Pt#750, Pt#751, Pt#760, Pt#767, Pt#840, Pt#883, Pt#983, Pt#1018	12	–	10	15	2,30,65,71,73,81,82,105,108,113,120,97,134
c.214C>T, p.Arg72Cys	Missense	23 (20/3/0)	Pt#35, Pt#151, Pt#166, Pt#175, Pt#176, Pt#327, Pt#332, Pt#388, Pt#405, Pt#406, Pt#557, Pt#585, Pt#603, Pt#709, Pt#710, Pt#772, Pt#773, Pt#804, Pt#842, Pt#866, Pt#867, Pt#892, Pt#896	7	6	13	10	52,57,65,74,81,85,86,99,109
c.1159_1162dup, p.Ser388Ter	Nonsense	22 (20/2/0)	Pt#32, Pt#33, Pt#40, Pt#126, Pt#146, Pt#174, Pt#314, Pt#337, Pt#391, Pt#403, Pt#440, Pt#441, Pt#458, Pt#459, Pt#526, Pt#527, Pt#562, Pt#661, Pt#672, Pt#733, Pt#796, Pt#934	7	2	6	16	14,54,71,81,86,99,101,105,125,126
c.262C>T, p.Arg88Cys	Missense	17 (14/2/1)	Pt#172, Pt#232, Pt#237, Pt#386, Pt#544, Pt#567, Pt#769, Pt#800, Pt#843, Pt#844, Pt#854, Pt#855, Pt#858, Pt#951, Pt#961, Pt#990, Pt#991	2	10	11	6	58,89,90,112,113,140
c.379C>T, p.Arg127Trp	Missense	17 (9/8/0)	Pt#283, Pt#384, Pt#407, Pt#408, Pt#409, Pt#410, Pt#561, Pt#564, Pt#598, Pt#631, Pt#701, Pt#702, Pt#703, Pt#734, Pt#805, Pt#931, Pt#937	5	2	11	6	81,88,99
c.506C>T, p.Ala169Val	Missense	14 (4/10/0)	Pt#289, Pt#357, Pt#553, Pt#586, Pt#681, Pt#707, Pt#727, Pt#728, Pt#777, Pt#787, Pt#791, Pt#813, Pt#821, Pt#847	6	1	12	2	99,97
c.483C>T, p.?	Splice	13 (7/6/0)	Pt#262, Pt#301, Pt#309, Pt#313, Pt#379, Pt#596, Pt#641, Pt#700, Pt#731, Pt#803, Pt#825, Pt#955, Pt#986	5	1	8	5	92,99,110

Supplementary Table 6. ***PDHA1* variants included in the study in decreasing frequency** *(continued)*

Variant	Classification	No. of cases (M/F/NA)	Case ID	DNV	INH	UP	P	References
c.380G>A, p.Arg127Gln	Missense	12 (2/10/0)	Pt#66, Pt#78, Pt#287, Pt#288, Pt#347, Pt#411, Pt#670, Pt#824, Pt#846, Pt#975, Pt#993, Pt#994	4	2	4	8	81,99,120,140,97,105
c.905G>A, p.Arg302His	Missense	11 (3/8/0)	Pt#118, Pt#212, Pt#286, Pt#376, Pt#535, Pt#543, Pt#687, Pt#735, Pt#736, Pt#1003, Pt#1012	3	2	4	7	37,79,81,99,120,127,142,144,105
c.787C>T, p.Arg263Ter	Nonsense	10 (2/8/0)	Pt#97, Pt#113, Pt#330, Pt#552, Pt#554, Pt#581, Pt#643, Pt#695, Pt#793, Pt#909	3	–	7	3	99,120,105
c.355C>T, p.Arg119Trp	Missense	7 (0/7/0)	Pt#108, Pt#292, Pt#781, Pt#811, Pt#933, Pt#978, Pt#1008	3	–	4	3	99,120,135,105
c.498C>T, p.?	Splice	7 (1/6/0)	Pt#12, Pt#230, Pt#294, Pt#300, Pt#348, Pt#613, Pt#636	4	–	2	5	92,99,121,122,97
c.615C>G, p.Phe205Leu	Missense	7 (6/1/0)	Pt#319, Pt#321, Pt#462, Pt#534, Pt#795, Pt#865, Pt#963	4	–	3	4	83,99,127
c.628A>G, p.Met210Val	Missense	6 (4/2/0)	Pt#64, Pt#268, Pt#559, Pt#828, Pt#839, Pt#849	1	–	4	2	75,81,110,120,105
c.858_861dup, p.Arg288LeufsTer10	Frameshift	6 (0/6/0)	Pt#233, Pt#272, Pt#390, Pt#600, Pt#926, Pt#927	4	–	3	3	112,113,150
c.788G>A, p.Arg263Gln	Missense	6 (6/0/0)	Pt#7, Pt#91, Pt#189, Pt#801, Pt#836, Pt#872	3	1	3	3	67,118,120
c.910C>T, p.Arg304Ter	Nonsense	6 (0/6/0)	Pt#284, Pt#827, Pt#829, Pt#880, Pt#939, Pt#1009	4	–	4	2	99,135
c.933_935del, p.Arg311del	Indel	5 (2/3/0)	Pt#28, Pt#63, Pt#234, Pt#541, Pt#831	1	1	2	3	99,135
c.592G>A, p.Ala198Thr	Missense	5 (4/1/0)	Pt#73, Pt#246, Pt#350, Pt#387, Pt#558	3	–	1	4	34,114,120,97,105
c.871G>A, p.Gly291Arg	Missense	5 (0/5/0)	Pt#122, Pt#209, Pt#768, Pt#826, Pt#885	1	–	3	2	72,120,105
c.947C>T, p.Pro316Leu	Missense	5 (1/4/0)	Pt#214, Pt#216, Pt#265, Pt#394, Pt#732	–	–	1	4	19,86,130,150
c.963_977dup, p.Lys321_Val325dup	Indel	5 (4/1/0)	Pt#46, Pt#83, Pt#575, Pt#792, Pt#851	–	3	3	2	73,105,120
c.262C>A, p.Arg88Ser	Missense	4 (4/0/0)	Pt#150, Pt#333, Pt#537, Pt#1022	–	–	–	4	99,102,106,76
c.302G>T, p.Cys101Phe	Missense	4 (1/3/0)	Pt#253, Pt#372, Pt#443, Pt#741	2	–	1	3	84,95,99,108

Supplementary Table 6. ***PDHA1* variants included in the study in decreasing frequency** *(continued)*

Variant	Classification	No. of cases (M/F/NA)	Case ID	DNV	INH	UP	P	References
c.422G>A, p.Arg141Gln	Missense	4 (2/2/0)	Pt#98, Pt#412, Pt#664, Pt#692	–	–	2	2	81,105
c.410A>G, p.Glu137Gly	Missense	4 (1/3/0)	Pt#74, Pt#987, Pt#988, Pt#989	–	2	–	4	120,140
c.523G>A, p.Ala175Thr	Missense	4 (2/2/0)	Pt#25, Pt#258, Pt#621, Pt#759	1	–	2	2	45,71,81
c.707C>A, p.Ala236Glu	Missense	4 (0/4/0)	Pt#579, Pt#618, Pt#655, Pt#913	3	–	4	–	This study#
c.883A>G, p.Ser295Gly	Missense	4 (2/1/1)	Pt#623, Pt#624, Pt#625, Pt#671	–	–	4	–	This study
c.1045G>A, p.Ala349Thr	Missense	4 (4/0/0)	Pt#68 Pt#465 Pt#512, Pt#979	–	–	1	3	44,120,105
c.29G>C, p.Arg10Pro	Missense	3 (2/1/0)	Pt#22, Pt#23, Pt#24	1	2	–	3	70
c.131A>G, p.His44Arg	Missense	3 (2/1/0)	Pt#1, Pt#9, Pt#977	–	–	1	2	13,49
c.148C>T, p.Pro50Ser	Missense	3 (0/0/3)	Pt#130, Pt#136, Pt#137	–	–	–	3	49
c.292-2A>G, p.?	Splice	3 (0/3/0)	Pt#278, Pt#725, Pt#726	–	–	2	1	06/11/2025 06:52:00
c.407C>T, p.Ala136Val	Missense	3 (2/1/0)	Pt#448, Pt#449, Pt#992	–	2	–	3	87,140
c.464T>C, p.Met155Thr	Missense	3 (1/2/0)	Pt#776, Pt#785, Pt#830	2	1	3	–	This study
c.484G>A, p.Gly162Arg	Missense	3 (0/3/0)	Pt#154, Pt#642, Pt#679	–	–	2	1	74
c.499G>A, p.Val167Met	Missense	3 (0/3/0)	Pt#100, Pt#198, Pt#199	1	–	–	3	65,120
c.535C>G, p.Leu179Val	Missense	3 (3/0/0)	Pt#899, Pt#906, Pt#907	–	2	3	–	This study#
c.615C>A, p.Phe205Leu	Missense	3 (3/0/0)	Pt#37, Pt#416, Pt#611	1	–	1	2	16,71
c.642G>T, p.Trp214Cys	Missense	3 (0/3/0)	Pt#453, Pt#454, Pt#964	–	–	1	2	101

Supplementary Table 6. ***PDHA1* variants included in the study in decreasing frequency** *(continued)*

Variant	Classification	No. of cases (M/F/NA)	Case ID	DNV	INH	UP	P	References
c.650C>T, p.Pro217Leu	Missense	3 (2/1/0)	Pt#61, Pt#852, Pt#949	2	–	2	1	18,105,120
c.679T>C, p.Tyr227His	Missense	3 (3/0/0)	Pt#245, Pt#345, Pt#583	3	–	1	2	41,114
c.831+1G>A, p.?	Splice	3 (0/3/0)	Pt#120, Pt#712, Pt#1016	–	–	1	2	
c.832G>A, p.Gly278Arg	Missense	3 (3/0/0)	Pt#14, Pt#251, Pt#580	1	1	2	1	117,122
c.862C>T, p.Arg288Cys	Missense	3 (0/3/0)	Pt#104, Pt#658, Pt#823	1	–	2	1	120,105
c.861_862insT, p.Arg288SerfsTer9	Frameshift	3 (0/3/0)	Pt#222, Pt#784, Pt#789	1	–	2	1	69
c.900-3_922dup, p.?	Splice	3 (0/3/0)	Pt#119, Pt#550, Pt#551	–	2	2	1	120
c.936_939del, p.Ser312ArgfsTer13	Frameshift	3 (0/3/0)	Pt#224, Pt#280, Pt#958	–	–	1	2	20,99
c.1144_1159dup, p.Lys387ThrfsTer6	Nonsense	3 (3/0/0)	Pt#365, Pt#180, Pt#799	1	1	1	2	75,97
c.1158dup, p.Lys387Ter	Nonsense	3 (1/2/0)	Pt#270, Pt#271, Pt#275	–	–	–	3	1,110
c.1167_1170del, p.Ser390LysfsTer33	Frameshift	3 (3/0/0)	Pt#21, Pt#173, Pt#859	1	1	1	2	1,53
c.261T>G, p.Ile87Met	Missense	2 (2/0/0)	Pt#276, Pt#622	–	–	1	1	110
c.265G>A, p.Gly89Ser	Missense	2 (0/2/0)	Pt#208, Pt#229	1	–	–	2	25,72
c.328delinsAGA, p.Pro110ArgfsTer71	Frameshift	2 (0/1/1)	Pt#447, Pt#744	1	–	1	1	104
c.337C>G, p.His113Asp	Missense	2 (0/2/0)	Pt#153, Pt#346	1	–	–	2	74,97
c.364G>A, p.Gly122Ser	Missense	2 (1/1/0)	Pt#689, Pt#810	–	–	2	–	This study#
c.383G>A, p.Gly128Asp	Missense	2 (1/1/0)	Pt#84, Pt#861	–	1	1	1	120,105

Supplementary Table 6. ***PDHA1* variants included in the study in decreasing frequency** *(continued)*

Variant	Classification	No. of cases (M/F/NA)	Case ID	DNV	INH	UP	P	References
c.421C>G, p.Arg141Gly	Missense	2 (0/0/2)	Pt#141, Pt#142	–	–	–	2	133
c.422G>T, p.Arg141Leu	Missense	2 (2/0/0)	Pt#450, Pt#1025	1	–	–	2	26,106
c.430G>A, p.Gly144Ser	Missense	2 (1/1/0)	Pt#749, Pt#775	–	2	2	–	This study
c.434G>A, p.Cys145Tyr	Missense	2 (0/2/0)	Pt#924, Pt#1026	1	–	1	1	132
c.442G>A, p.Gly148Arg	Missense	2 (2/0/0)	Pt#42, Pt#43	–	2	–	2	66
c.481_483del, p.Phe160del	Indel	2 (0/2/0)	Pt#548, Pt#656	1	–	2	–	This study
c.482A>G, p.Tyr161Cys	Missense	2 (0/2/0)	Pt#380, Pt#570	1	–	1	1	33
c.542G>A, p.Cys181Tyr	Missense	2 (0/2/0)	Pt#870, Pt#901	1	–	2	–	This study
c.555A>G, p.?	Splice	2 (2/0/0)	Pt#315, Pt#316	–	2	–	2	99
c.584G>C, p.Gly195Ala	Missense	2 (2/0/0)	Pt#373, Pt#374	2	–	–	2	86,91
c.613T>C, p.Phe205Leu	Missense	2 (1/1/0)	Pt#832, Pt#833	–	2	2	–	This study#
c.616G>A, p.Glu206Lys	Missense	2 (0/2/0)	Pt#103, Pt#369	1	–	–	2	93,105,120
c.640T>C, p.Trp214Arg	Missense	2 (2/0/0)	Pt#2, Pt#417	1	–	–	2	24,81
c.647T>C, p.Leu216Ser	Missense	2 (1/1/0)	Pt#252, Pt#473	2	–	–	2	48,90
c.687G>A, p.Met229Ile	Missense	2 (0/2/0)	Pt#890, Pt#935	1	–	2	–	This study#
c.727_729del, p.Tyr243del	Indel	2 (0/2/0)	Pt#351, Pt#433	2	–	–	2	81,97
c.728A>G, p.Tyr243Cys	Missense	2 (1/1/0)	Pt#115, Pt#339	1	–	–	2	99,120,105

Supplementary Table 6. ***PDHA1* variants included in the study in decreasing frequency** *(continued)*

Variant	Classification	No. of cases (M/F/NA)	Case ID	DNV	INH	UP	P	References
c.733A>G, p.Arg245Gly	Missense	2 (0/2/0)	Pt#290, Pt#1027	1	–	–	2	96,99
c.738C>T, p.?	Splice	2 (0/2/0)	Pt#117, Pt#973	–	–	1	1	120
c.748C>A, p.Pro250Thr	Missense	2 (2/0/0)	Pt#385, Pt#460	1	–	–	2	86,89,101
c.749C>T, p.Pro250Leu	Missense	2 (2/0/0)	Pt#324, Pt#538	–	1	–	2	99,102
c.759+26G>A, p.?	Splice	2 (2/0/0)	Pt#3, Pt#318	1	1	–	2	28,99
c.821G>C, p.Arg274Thr	Missense	2 (1/1/0)	Pt#170, Pt#171	–	1	–	2	129
c.847G>A, p.Glu283Lys	Missense	2 (1/1/0)	Pt#75, Pt#105	–	–	–	2	120,105
c.853C>T, p.Gln285Ter	Nonsense	2 (0/2/0)	Pt#267, Pt#589	–	1	1	1	110
c.863G>A, p.Arg288His	Missense	2 (0/2/0)	Pt#56, Pt#677	–	–	1	1	80
c.890dup, p.Gly298TrpfsTer16	Frameshift	2 (0/2/0)	Pt#96, Pt#500	–	–	–	2	120,105
c.899+1G>C, p.?	Splice	2 (0/2/0)	Pt#121, Pt#341	1	–	–	2	100,105,120
c.924_930del, p.Ser312ValfsTer12	Frameshift	2 (0/2/0)	Pt#660, Pt#706	–	–	2	–	This study
c.924G>T, p.Gln308His	Missense	2 (0/2/0)	Pt#255, Pt#743	1	–	1	1	108
c.931A>G, p.Arg311Gly	Missense	2 (1/1/0)	Pt#665, Pt#666	–	–	2	–	This study#
c.938_940del, p.Lys313del	Indel	2 (0/2/0)	Pt#195, Pt#721	1	–	1	1	62
c.949_952dup, p.Met318AsnfsTer23	Frameshift	2 (0/2/0)	Pt#508, Pt#95	–	–	–	2	105,120
c.968_980dup, p.Ser327ArgfsTer17	Frameshift	2 (0/2/0)	Pt#30, Pt#711	–	–	1	1	71

Supplementary Table 6. ***PDHA1* variants included in the study in decreasing frequency** *(continued)*

Variant	Classification	No. of cases (M/F/NA)	Case ID	DNV	INH	UP	P	References
c.969_1004dup, p.Met324_Leu335dup	Indel	2 (1/1/0)	Pt#54, Pt#55	–	2	–	2	78
c.986T>G, p.Leu329Arg	Missense	2 (2/0/0)	Pt#425, Pt#761	1	–	1	1	81
c.1022_1034del, p.Glu341GlyfsTer79	Frameshift	2 (0/2/0)	Pt#925, Pt#952	2	–	2	–	This study
c.1042_1045dup, p.Ala349GlyfsTer11	Frameshift	2 (1/1/0)	Pt#627, Pt#696	–	–	2	–	This study
c.1051C>T, p.Gln351Ter	Nonsense	2 (0/2/0)	Pt#594 Pt#704	1	–	2	–	This study
c.1073_1092del, p.Glu358GlyfsTer12	Frameshift	2 (0/2/0)	Pt#19, Pt#1019	1	–	–	2	3,106
c.1100A>T, p.His367Leu	Missense	2 (0/2/0)	Pt#127 Pt#128	2	–	–	2	123
c.1121_1144dup, p.Pro374_Asn381dup	Indel	2 (2/0/0)	Pt#10, Pt#364	1	–	–	2	122,97
c.1157_1158dup, p.Lys387LeufsTer38	Frameshift	2 (2/0/0)	Pt#72, Pt#80	–	–	–	2	120
c.1157_1162del, p.Phe386_Lys387del	Indel	2 (1/1/0)	Pt#279, Pt#1014	1	1	–	2	51,99
c.12_13insACTT, p.Leu5ThrfsTer26	Frameshift	1 (0/1/0)	Pt#568	–	–	1	–	This study
c.122_124del, p.Cys41_Asp42delinsTyr	Indel	1 (0/1/0)	Pt#893	1	–	1	–	This study
c.149C>G, p.Pro50Arg	Missense	1 (0/1/0)	Pt#616	–	–	1	–	This study
c.193_195delinsCAA, p.Tyr65Gln	Indel	1 (1/0/0)	Pt#747	1	–	1	–	This study
c.194A>C, p.Tyr65Ser	Missense	1 (1/0/0)	Pt#982	–	1	1	–	This study#
c.212T>C, p.Val71Ala	Missense	1 (1/0/0)	Pt#442	1	–	–	1	84

Supplementary Table 6. ***PDHA1* variants included in the study in decreasing frequency** *(continued)*

Variant	Classification	No. of cases (M/F/NA)	Case ID	DNV	INH	UP	P	References
c.224A>C, p.Glu75Ala	Missense	1 (1/0/0)	Pt#335	–	1	–	1	38,99
c.224A>T, p.Glu75Val	Missense	1 (0/1/0)	Pt#652	–	–	1	–	This study
c.224A>G, p.Glu75Gly	Missense	1 (0/1/0)	Pt#753	1	–	1	–	This study
c.225G>T, p.Glu75Asp	Missense	1 (0/1/0)	Pt#125	–	–	–	1	120
c.249dup, p.Gln84ThrfsTer12	Frameshift	1 (0/1/0)	Pt#297	1	–	–	1	99
c.269T>C, p.Phe90Ser	Missense	1 (0/1/0)	Pt#112	–	–	–	1	105,120
c.272G>C, p.Cys91Ser	Missense	1 (0/0/1)	Pt#766	–	–	1	–	This study
c.291G>A, p.?	Splice	1 (1/0/0)	Pt#235	1	–	–	1	112
c.291+5G>A, p.?	Splice	1 (0/1/0)	Pt#1028	1	–	1	–	This study
c.301T>C, p.Cys101Arg	Missense	1 (0/1/0)	Pt#111	–	–	–	1	105,120
c.329C>A, p.Pro110His	Missense	1 (1/0/0)	Pt#748	1	–	1	–	This study
c.332C>T, p.Thr111Ile	Missense	1 (1/0/0)	Pt#164	–	–	–	1	55
c.335A>G, p.Asp112Gly	Missense	1 (0/1/0)	Pt#757	–	–	1	–	This study
c.363C>A, p.His121Gln	Missense	1 (1/0/0)	Pt#396	1	–	–	1	86
c.364G>C, p.Gly122Arg	Missense	1 (0/0/1)	Pt#746	–	–	1	–	This study
c.394C>T, p.Arg132Ter	Nonsense	1 (0/1/0)	Pt#868	1	–	1	–	This study
c.409G>A, p.Glu137Lys	Missense	1 (0/1/0)	Pt#574	1	–	1	–	This study#

Supplementary Table 6. ***PDHA1* variants included in the study in decreasing frequency** *(continued)*

Variant	Classification	No. of cases (M/F/NA)	Case ID	DNV	INH	UP	P	References
c.409G>C, p.Glu137Gln	Missense	1 (0/1/0)	Pt#11	–	–	–	1	122
c.412C>T, p.Leu138Phe	Missense	1 (1/0/0)	Pt#398	1	–	–	1	86
c.416C>G, p.Thr139Arg	Missense	1 (0/1/0)	Pt#972	–	–	1	–	This study
c.419-17_419-14del, p.?	Splice	1 (0/1/0)	Pt#260	–	–	–	1	110
c.419-2A>G, p.?	Splice	1 (0/1/0)	Pt#1002	–	–	–	1	141
c.421C>T, p.Arg141*	Nonsense	1 (0/1/0)	Pt#604	1	–	1	–	This study
c.429_431del, p.Gly144del	Indel	1 (1/0/0)	Pt#367	–	–	–	1	93
c.430G>C, p.Gly144Arg	Missense	1 (0/1/0)	Pt#719	–	–	1	–	This study
c.431G>A, p.Gly144Asp	Missense	1 (0/1/0)	Pt#295	–	–	–	1	99
c.433_435del, p.Cys145del	Indel	1 (0/1/0)	Pt#8	1	–	–	1	118
c.449G>A, p.Gly150Glu	Missense	1 (0/1/0)	Pt#342	1	–	–	1	100
c.451G>A, p.Gly151Arg	Missense	1 (0/1/0)	Pt#244	1	–	–	1	114
c.454T>C, p.Ser152Pro	Missense	1 (1/0/0)	Pt#871	–	1	1	–	This study
c.454T>A, p.Ser152Thr	Missense	1 (0/1/0)	Pt#1029	1	–	1	–	This study
c.455C>T, p.Ser152Leu	Missense	1 (0/1/0)	Pt#765	–	–	1	–	This study#
c.457A>G, p.Met153Val	Missense	1 (1/0/0)	Pt#370	1	–	–	1	93
c.465G>T, p.Met155Ile	Missense	1 (1/0/0)	Pt#969	–	–	1	–	This study#

Supplementary Table 6. ***PDHA1* variants included in the study in decreasing frequency** *(continued)*

Variant	Classification	No. of cases (M/F/NA)	Case ID	DNV	INH	UP	P	References
c.478_479delTTinsAA, p.Phe160Asn	Missense	1 (0/1/0)	Pt#881	–	–	1	–	This study
c.479_481del, p.Phe160del	Indel	1 (0/1/0)	Pt#980	1	–	1	–	This study
c.495C>T, p.?	Splice	1 (1/0/0)	Pt#863	1	–	1	–	This study
c.499G>T, p.Val167Leu	Missense	1 (0/1/0)	Pt#900	–	–	1	–	This study#
c.511-414_899+584del, p.?	Frameshift	1 (0/1/0)	Pt#528	–	–	–	1	32
c.511-30G>A, p.?	Splice	1 (1/0/0)	Pt#358	1	–	–	1	97
c.511G>C, p.Val171Leu	Missense	1 (0/1/0)	Pt#884	1	–	1	–	This study#
c.511G>A, p.Val171Met	Missense	1 (0/1/0)	Pt#264	–	–	–	1	110
c.513_759+2del, p.?	Splice	1 (0/1/0)	Pt#431	–	–	–	1	81
c.515C>T, p.Pro172Leu	Missense	1 (0/1/0)	Pt#349	1	–	–	1	97
c.523G>C, p.Ala175Pro	Missense	1 (0/1/0)	Pt#168	–	–	–	1	11
c.530T>C, p.Ile177Thr	Missense	1 (1/0/0)	Pt#65	–	–	–	1	105,120
c.536T>G, p.Leu179Arg	Missense	1 (0/1/0)	Pt#572	1	–	1	–	This study#
c.548A>G, p.Tyr183Cys	Missense	1 (1/0/0)	Pt#822	–	1	1	–	This study#
c.562_858dup, p.Glu188_Thr286dup	Indel	1 (0/1/0)	Pt#632	–	–	1	–	This study
c.562G>A, p.Glu188Lys	Missense	1 (0/1/0)	Pt#397	1	–	–	1	86
c.586G>A, p.Asp196Asn	Missense	1 (0/1/0)	Pt#930	–	–	1	–	This study
c.593C>T, p.Ala198Val	Missense	1 (1/0/0)	Pt#675	–	–	1	–	This study

Supplementary Table 6. ***PDHA1* variants included in the study in decreasing frequency** *(continued)*

Variant	Classification	No. of cases (M/F/NA)	Case ID	DNV	INH	UP	P	References
c.595G>A, p.Ala199Thr	Missense	1 (1/0/0)	Pt#206	1	–	–	1	65
c.599A>C, p.Asn200Thr	Missense	1 (1/0/0)	Pt#452	–	1	–	1	43
c.604-10C>G, p.?	Splice	1 (1/0/0)	Pt#263	–	–	–	1	110
c.606_609del, p.Gln203TyrfsTer49	Frameshift	1 (0/1/0)	Pt#455	–	–	–	1	101
c.616G>T, p.Glu206Ter	Nonsense	1 (0/1/0)	Pt#162	1	–	–	1	134
c.616G>C, p.Glu206Gln	Missense	1 (0/1/0)	Pt#918	1	–	1	–	This study#
c.619G>C, p.Ala207Pro	Missense	1 (1/0/0)	Pt#782	1	–	1	–	This study
c.626A>G, p.Asn209Ser	Missense	1 (0/1/0)	Pt#779	1	–	1	–	This study
c.629T>C, p.Met210Thr	Missense	1 (1/0/0)	Pt#81	–	–	–	1	105,120
c.640T>G, p.Trp214Gly	Missense	1 (0/1/0)	Pt#763	–	–	1	–	This study#
c.643A>C, p.Lys215Gln	Missense	1 (1/0/0)	Pt#1030	–	–	1	–	This study
c.648A>C, p.Leu216Phe	Missense	1 (1/0/0)	Pt#463	–	1	–	1	83
c.649C>G, p.Pro217Ala	Missense	1 (1/0/0)	Pt#1006	–	–	–	1	59,135
c.649C>A, p.Pro217Thr	Missense	1 (1/0/0)	Pt#794	–	–	1	–	This study
c.650C>G, p.Pro217Arg	Missense	1 (1/0/0)	Pt#323	1	–	–	1	99
c.666_667del, p.Cys222Ter	Nonsense	1 (0/1/0)	Pt#432	1	–	–	1	81
c.677G>A, p.Arg226His	Missense	1 (1/0/0)	Pt#88	–	–	–	1	120

Supplementary Table 6. ***PDHA1* variants included in the study in decreasing frequency** *(continued)*

Variant	Classification	No. of cases (M/F/NA)	Case ID	DNV	INH	UP	P	References
c.680A>G, p.Tyr227Cys	Missense	1 (0/1/0)	Pt#344	–	–	–	1	100
c.688G>A, p.Gly230Arg	Missense	1 (0/0/1)	Pt#1011	–	–	–	1	124
c.691A>G, p.Thr231Ala	Missense	1 (1/0/0)	Pt#205	–	–	–	1	65
c.692C>G, p.Thr231Arg	Missense	1 (0/1/0)	Pt#540	1	–	1	–	This study#
c.692C>A, p.Thr231Lys	Missense	1 (1/0/0)	Pt#418	–	–	–	1	81
c.703_706del, p.Ala236GlnfsTer16	Frameshift	1 (0/1/0)	Pt#771	–	–	1	–	This study
c.703A>G, p.Arg235Gly	Missense	1 (1/0/0)	Pt#320	1	–	–	1	99
c.705_706del, p.Arg235SerfsTer6	Frameshift	1 (0/1/0)	Pt#1007	–	–	–	1	135
c.721_724dup, p.Tyr242Ter	Nonsense	1 (0/1/0)	Pt#691	–	–	1	–	This study#
c.727T>A, p.Tyr243Asn	Missense	1 (1/0/0)	Pt#218	–	–	–	1	69
c.728A>C, p.Tyr243Ser	Missense	1 (1/0/0)	Pt#305	1	–	–	1	27,99
c.729C>A, p.Tyr243Ter	Nonsense	1 (0/1/0)	Pt#375	–	–	–	1	36
c.730_731del, p.Lys244GlufsTer30	Frameshift	1 (0/1/0)	Pt#848	1	–	1	–	This study
c.754C>G, p.Leu252Val	Missense	1 (1/0/0)	Pt#614	–	–	1	–	This study#
c.757A>G, p.Arg253Gly	Missense	1 (1/0/0)	Pt#536	1	–	–	1	40
c.760-2A>G, p.?	Splice	1 (0/1/0)	Pt#630	–	–	1	–	This study
c.762_831+2del, p.?	Splice	1 (0/1/0)	Pt#434	–	–	–	1	81

Supplementary Table 6. ***PDHA1* variants included in the study in decreasing frequency** *(continued)*

Variant	Classification	No. of cases (M/F/NA)	Case ID	DNV	INH	UP	P	References
c.773A>C, p.Asp258Ala	Missense	1 (1/0/0)	Pt#213	1	–	–	1	68
c.778C>G, p.Leu260Val	Missense	1 (0/1/0)	Pt#720	–	–	1	–	This study
c.784G>C, p.Val262Leu	Missense	1 (1/0/0)	Pt#950	–	1	1	–	This study
c.784G>T, p.Val262Phe	Missense	1 (0/1/0)	Pt#402	1	–	–	1	86
c.788G>C, p.Arg263Pro	Missense	1 (0/1/0)	Pt#495	–	–	–	1	105
c.832G>C, p.Gly278Arg	Missense	1 (1/0/0)	Pt#336	–	1	–	1	99
c.833G>A, p.Gly278Glu	Missense	1 (1/0/0)	Pt#856	–	1	1	–	This study
c.839T>G, p.Ile280Ser	Missense	1 (1/0/0)	Pt#474	–	1	–	1	98
c.844A>G, p.Met282Val	Missense	1 (1/0/0)	Pt#338	–	–	–	1	99
c.845_846insTCT, p.Met282delinsIleLeu	Indel	1 (0/0/1)	Pt#135	–	–	–	1	133
c.845T>G, p.Met282Arg	Missense	1 (0/1/0)	Pt#874	1	–	1	–	This study
c.853_865dup, p.Tyr289SerfsTer12	Frameshift	1 (0/1/0)	Pt#1004	–	–	–	1	143
c.862C>A, p.Arg288Ser	Missense	1 (0/1/0)	Pt#16	1	–	–	1	128
c.863G>T, p.Arg288Leu	Missense	1 (0/1/0)	Pt#758	1	–	1	–	This study
c.868C>T, p.His290Tyr	Missense	1 (1/0/0)	Pt#688	–	–	1	–	This study#
c.869A>C, p.His290Pro	Missense	1 (0/1/0)	Pt#790	1	–	1	–	60

Supplementary Table 6. ***PDHA1* variants included in the study in decreasing frequency** *(continued)*

Variant	Classification	No. of cases (M/F/NA)	Case ID	DNV	INH	UP	P	References
c.875A>T, p.His292Leu	Missense	1 (0/1/0)	Pt#201	–	–	–	1	65
c.888C>G, p.Asp296Glu	Missense	1 (1/0/0)	Pt#404	1	–	–	1	29
c.886G>A, p.Asp296Asn	Missense	1 (0/1/0)	Pt#783	1	–	1	–	This study
c.892G>A, p.Gly298Arg	Missense	1 (1/0/0)	Pt#850	–	–	–	1	This study
c.893G>A, p.Gly298Glu	Missense	1 (1/0/0)	Pt#311	1	–	–	1	99
c.894_899+1dup, p.?	Splice	1 (0/1/0)	Pt#635	–	–	1	–	This study
c.899+3_988del, p.?	Splice	1 (0/1/0)	Pt#954	1	–	1	–	This study
c.899_918del, p.Ser300AsnfsTer7	Frameshift	1 (0/1/0)	Pt#159	–	–	–	1	8
c.900-41_900-23del, p.?	Splice	1 (1/0/0)	Pt#620	–	–	1	–	This study
c.900-16_905dup, p.?	Splice	1 (1/0/0)	Pt#682	–	–	1	–	This study
c.900-12_920dup, p.?	Splice	1 (0/1/0)	Pt#223	–	–	–	1	15
c.900-6_958dup, p.?	Splice	1 (1/0/0)	Pt#368	–	1	–	1	93
c.900-3_917dup, p.?	Splice	1 (1/0/0)	Pt#194	–	–	–	1	4
c.900-1_903dup, p.?	Splice	1 (0/1/0)	Pt#160	–	–	–	1	9
c.900-1G>A, p.?	Splice	1 (0/1/0)	Pt#371	–	1	–	1	35
c.900_932dup, p.?	Splice	1 (0/1/0)	Pt#640	–	–	1	–	This study
c.900_903dup, p.Arg302LeufsTer13	Frameshift	1 (0/1/0)	Pt#943	1	–	1	–	This study
c.901_1004dup, p.Lys336ThrfsTer10	Frameshift	1 (0/1/0)	Pt#41	–	–	–	1	17
c.905G>T, p.Arg302Leu	Missense	1 (0/1/0)	Pt#424	1	–	–	1	81,82
c.905_927inv, p.Arg302_Glu309delinsLeuProGluPheLeuLeuValTyr	Missense	1 (0/1/0)	Pt#887	–	–	1	–	This study

Supplementary Table 6. ***PDHA1* variants included in the study in decreasing frequency** *(continued)*

Variant	Classification	No. of cases (M/F/NA)	Case ID	DNV	INH	UP	P	References
c.913_929dup, p.Arg311LysfsTer6	Frameshift	1 (0/1/0)	Pt#891	–	–	1	–	This study
c.914_915insGATAGTTACCGTACACGAGAA, p.Arg304_Glu305insAspSerTyrArgThrArgGlu	Indel	1 (1/0/0)	Pt#1023	–	–	–	1	7
c.917_924dup, p.Glu309LysfsTer5	Frameshift	1 (0/1/0)	Pt#599	1	–	1	–	This study
c.927dup, p.Val310SerfsTer4	Frameshift	1 (0/1/0)	Pt#383	1	–	–	1	88
c.929_932del, p.Val310GlufsTer15	Frameshift	1 (0/1/0)	Pt#261	–	–	–	1	110
c.932_935del, p.Arg311IlefsTer14	Frameshift	1 (0/1/0)	Pt#680	–	–	1	–	This study
c.933_989dup, p.Lys313_Ser331dup	Indel	1 (0/1/0)	Pt#837	1	–	1	–	This study
c.934_992dup, p.Ser331ArgfsTer15	Frameshift	1 (0/1/0)	Pt#816	–	–	1	–	This study#
c.937_940dup, p.Ser314LysfsTer3	Frameshift	1 (0/1/0)	Pt#571	–	–	1	–	This study#
c.937_942dup, p.Lys313_Ser314dup	Indel	1 (0/1/0)	Pt#285	1	–	–	1	99
c.939_950del, p.Lys313_Ile317delinsAsn	Indel	1 (0/1/0)	Pt#928	1	–	1	–	This study
c.940A>T, p.Ser314Cys	Missense	1 (0/1/0)	Pt#102	–	–	–	1	120
c.943G>A, p.Asp315Asn	Missense	1 (1/0/0)	Pt#219	–	–	–	1	69
c.947dup, p.Ile317TyrfsTer23	Frameshift	1 (0/1/0)	Pt#592	1	–	1	–	This study

Supplementary Table 6. ***PDHA1*** **variants included in the study in decreasing frequency** *(continued)*

Variant	Classification	No. of cases (M/F/NA)	Case ID	DNV	INH	UP	P	References
c.948_963dup, p.Asp322TyrfsTer23	Frameshift	1 (1/0/0)	Pt#158	1	–	–	1	74
c.949_950del, p.Ile317TyrfsTer22	Frameshift	1 (0/1/0)	Pt#200	–	–	–	1	65
c.950_962dup, p.Lys321AsnfsTer23	Frameshift	1 (0/1/0)	Pt#724	–	–	1	–	This study
c.957_959dup, p.Leu320dup	Indel	1 (1/0/0)	Pt#608	1	–	1	–	This study
c.960_1008+5dup, p.?	Splice	1 (1/0/0)	Pt#325	1	–	–	1	99
c.966_969del, p.Asp322GlufsTer3	Frameshift	1 (0/1/0)	Pt#698	–	–	1	–	This study
c.966_1011dup, p.Ile338_Ser390delinsGlnAspGlyGluGlnGlnSerCysGlnCysGlyArgThrLysGlyThrValThrCysSerTrpTrpPheGluGlyTrpLeu	Frameshift	1 (0/0/1)	Pt#140	–	–	–	1	133
c.968_976del, p.Arg323_Val325del	Indel	1 (0/1/0)	Pt#808	1	–	1	–	This study
c.978_1004dup, p.Ser327_Leu335dup	Indel	1 (1/0/0)	Pt#4	–	–	–	1	116
c.982_985dup, p.Leu329GlnfsTer12	Frameshift	1 (0/1/0)	Pt#99	–	–	–	1	120
c.983_986dup, p.Ala330SerfsTer11	Frameshift	1 (0/1/0)	Pt#510	–	–	–	1	105
c.985_998dup, p.Glu333AspfsTer8	Frameshift	1 (0/1/0)	Pt#809	1	–	1	–	This study#
c.986_998dup, p.Glu333AspfsTer11	Frameshift	1 (0/1/0)	Pt#106	–	–	–	1	105,120
c.989C>G, p.Ala330Gly	Missense	1 (0/1/0)	Pt#124	–	–	–	1	120

Supplementary Table 6. ***PDHA1* variants included in the study in decreasing frequency** *(continued)*

Variant	Classification	No. of cases (M/F/NA)	Case ID	DNV	INH	UP	P	References
c.1006_1008dup, p.Lys336dup	Indel	1 (1/0/0)	Pt#877	1	–	1	–	This study
c.1008+1_1008+27del, p.?	Splice	1 (1/0/0)	Pt#593	1	–	1	–	This study#
c.1011_1031dup, p.Ile338_Lys344dup	Indel	1 (0/1/0)	Pt#343	1	–	–	1	100
c.1011_1040dup, p.Ile338_Glu347dup	Indel	1 (0/1/0)	Pt#597	–	–	1	–	This study
c.1014_1032dup, p.Glu345Ter	Nonsense	1 (0/1/0)	Pt#647	–	–	1	–	This study
c.1026_1051dup, p.Gln351ArgfsTer82	Frameshift	1 (1/0/0)	Pt#714	–	–	1	–	This study
c.1026_1130dup, p.Lys344_Arg378dup	Indel	1 (0/1/0)	Pt#437	1	–	–	1	81
c.1033_1078dup, p.Pro360ArgfsTer3	Frameshift	1 (0/1/0)	Pt#31	–	–	–	1	71
c.1034_1037dup, p.Glu347AspfsTer2	Frameshift	1 (0/1/0)	Pt#817	1	–	1	–	This study#
c.1040_1046dup, p.Ala350GlyfsTer11	Frameshift	1 (0/1/0)	Pt#882	–	–	1	–	This study
c.1040_1063dup, p.Glu347_Thr354dup	Indel	1 (0/1/0)	Pt#654	–	–	1	–	This study
c.1046_1047insCAAT, p.Arg349SerfsTer4	Frameshift	1 (0/1/0)	Pt#780	1	–	1	–	This study
c.1050_1133dup, p.Gln351_Arg378dup	Indel	1 (1/0/0)	Pt#308	1	–	–	1	99
c.1052A>C, p.Gln351Pro	Missense	1 (0/1/0)	Pt#947	1	–	1	–	This study
c.1054_1057del, p.Phe352ProfsTer71	Frameshift	1 (0/0/1)	Pt#131	–	–	–	1	133
c.1057G>A, p.Ala353Thr	Missense	1 (1/0/0)	Pt#231	–	–	–	1	50

Supplementary Table 6. ***PDHA1* variants included in the study in decreasing frequency** *(continued)*

Variant	Classification	No. of cases (M/F/NA)	Case ID	DNV	INH	UP	P	References
c.1057G>C, p.Ala353Pro	Missense	1 (0/1/0)	Pt#228	–	–	–	1	23
c.1062_1124dup, p.Pro374_Phe375insLeuAlaAspProGluProProLeuGluGluLeuGlyTyrHisIleTyrSerSerAspProPro	Indel	1 (1/0/0)	Pt#869	1	–	1	–	This study
c.1063_1068del, p.Ala355_Asp356del	Indel	1 (0/1/0)	Pt#457	–	–	–	1	101
c.1064_1065insTAAG, p.Asp356LysfsTer4	Frameshift	1 (1/0/0)	Pt#857	1	–	1	–	This study
c.1065del, p.Asp356IlefsTer68	Frameshift	1 (0/1/0)	Pt#820	1	–	1	–	This study
c.1066_1090dup, p.Leu364delinsArgSerTer	Nonsense	1 (0/1/0)	Pt#602	1	–	1	–	This study
c.1069_1114dup, p.Asp372delinsAlaTer	Nonsense	1 (1/0/0)	Pt#626	–	–	1	–	This study
c.1071_1088dup, p.Glu362_Glu363insAspGluProProLeuGlu	Indel	1 (0/1/0)	Pt#225	–	–	–	1	21
c.1072G>A, p.Glu358Lys	Missense	1 (1/0/0)	Pt#470	–	–	–	1	90
c.1073_1094del, p.Glu358AlafsTer59	Frameshift	1 (0/1/0)	Pt#752	1	–	1	–	This study
c.1081_1125dup, p.Leu361_Phe375dup	Indel	1 (1/0/0)	Pt#853	1	–	1	–	This study
c.1083_1102dup, p.Ile368ArgfsTer63	Frameshift	1 (0/1/0)	Pt#94	–	–	–	1	105,120

Supplementary Table 6. ***PDHA1* variants included in the study in decreasing frequency** *(continued)*

Variant	Classification	No. of cases (M/F/NA)	Case ID	DNV	INH	UP	P	References
c.1083_1124del, p.Leu361_Pro374del	Indel	1 (0/1/0)	Pt#940	–	–	1	–	This study
c.1083_1124dup, p.Leu361_Pro374dup	Indel	1 (1/0/0)	Pt#436	–	1	–	1	81
c.1085_1093dup, p.Glu362_Leu364dup	Indel	1 (1/0/0)	Pt#47	1	–	–	1	73
c.1087_1119dup, p.Glu363_Pro373dup	Indel	1 (1/0/0)	Pt#277	1	–	–	1	46
c.1090_1155dup, p.Leu364_Lys385dup 22	Indel	1 (1/0/0)	Pt#563	1	–	1	–	This study
c.1091T>G, p.Leu364Arg	Missense	1 (0/1/0)	Pt#985	–	–	1	–	This study#
c.1093_1112dup, p.Ser371ArgfsTer60	Frameshift	1 (0/1/0)	Pt#157	–	–	–	1	74
c.1095_1118dup, p.Tyr366_Pro373dup	Indel	1 (1/0/0)	Pt#92	–	–	–	1	120
c.1100A>G, p.His367Arg	Missense	1 (1/0/0)	Pt#646	–	–	1	–	This study
c.1101_1154dup, p.Ile384_Lys385insAsnIleTyrSerSerAspProProPheGluValArgGlyAlaAsnGlnTrpIle	Indel	1 (1/0/0)	Pt#547	–	1	1	–	This study
c.1103_1116dup, p.Pro373SerfsTer56	Frameshift	1 (0/1/0)	Pt#673	–	–	1	–	This study
c.1106_1108del, p.Tyr369del	Indel	1 (1/0/0)	Pt#426	1	–	–	1	81
c.1105T>C, p.Tyr369His	Missense	1 (1/0/0)	Pt#361	1	–	–	1	97

Supplementary Table 6. ***PDHA1* variants included in the study in decreasing frequency** *(continued)*

Variant	Classification	No. of cases (M/F/NA)	Case ID	DNV	INH	UP	P	References
c.1116_1154dup, p.Ile384_Lys385insAsnProProPheGluValArgGlyAlaAsnGlnTrpIle	Indel	1 (1/0/0)	Pt#156	–	–	–	1	74
c.1119_1123del, p.Pro374_Phe375delinsTer	Nonsense	1 (0/1/0)	Pt#686	–	–	1	–	This study
c.1121_1159dup, p.Phe386_Lys387ins13	Indel	1 (1/0/0)	Pt#71	–	–	–	1	105,120
c.1124_1125dup, p.Glu376LeufsTer49	Frameshift	1 (0/1/0)	Pt#204	–	–	–	1	65
c.1124_1132del, p.Phe375_Arg378delinsCys	Indel	1 (0/1/0)	Pt#922	1	–	1	–	This study
c.1125del, p.Phe375LeufsTer49	Frameshift	1 (0/1/0)	Pt#815	1	–	1	–	This study
c.1126_1131dup, p.Glu376_Val377dup	Indel	1 (0/1/0)	Pt#560	–	–	1	–	This study
c.1133G>T, p.Arg378Leu	Missense	1 (0/0/1)	Pt#754	–	–	1	–	This study#
c.1134_1159dup, p.Lys387MetfsTer46	Frameshift	1 (1/0/0)	Pt#48	–	–	–	1	73
c.1137_1159dup, p.Lys387MetfsTer45	Frameshift	1 (1/0/0)	Pt#76	–	–	–	1	105,120
c.1138_1157dup, p.Phe386LeufsTer45	Frameshift	1 (1/0/0)	Pt#637	–	–	1	–	This study
c.1139_1142dup, p.Trp383SerfsTer5	Frameshift	1 (0/1/0)	Pt#266	–	–	–	1	110
c.1140_1150dup, p.Ile384ThrfsTer44	Frameshift	1 (0/1/0)	Pt#161	–	–	–	1	42
c.1142_1164del, p.Asn381SerfsTer43	Frameshift	1 (0/1/0)	Pt#256	–	–	–	1	108

Supplementary Table 6. ***PDHA1* variants included in the study in decreasing frequency** *(continued)*

Variant	Classification	No. of cases (M/F/NA)	Case ID	DNV	INH	UP	P	References
c.1143_1154del, p.Asn381_Ile384del	Indel	1 (0/1/0)	Pt#149	1	–	–	1	97
c.1144_1147dup, p.Trp383SerfsTer6	Frameshift	1 (0/1/0)	Pt#737	1	–	1	–	This study
c.1144C>T, p.Gln382Ter	Nonsense	1 (1/0/0)	Pt#165	1	–	–	1	56
c.1147_1158dup, p.Trp383_Phe386dup	Indel	1 (0/1/0)	Pt#57	1	–	–	1	80
c.1147T>C, p.Trp383Arg	Missense	1 (1/0/0)	Pt#89	–	–	–	1	120
c.1148G>A, p.Trp383Ter	Nonsense	1 (0/1/0)	Pt#291	1	–	–	1	99
c.1153_1155dup, p.Lys385dup	Indel	1 (0/1/0)	Pt#123	–	–	–	1	120
c.1157_1162dup, p.Phe386_Lys387dup	Indel	1 (1/0/0)	Pt#49	1	–	–	1	73
c.1159_1160del, p.Lys387ValfsTer44	Frameshift	1 (1/0/0)	Pt#196	–	–	–	1	62
c.1162T>C, p.Ser388Pro	Missense	1 (1/0/0)	Pt#889	–	–	1	–	This study
c.1163_1164del, p.Ser388CysfsTer43	Frameshift	1 (1/0/0)	Pt#835	1	–	1	–	This study
c.1163C>A, p.Ser388Ter	Nonsense	1 (1/0/0)	Pt#430	–	–	–	1	81

All *PDHA1* variants included in this study were (re)classified according to NM_000284.3 reference transcript. ACMG – variant pathogenicity interpretation according to the recommendations of the American College of Medical Genetics and Genomics and the Association for Molecular Pathology.[146] Reference column contains references to the records of published cases. Indel – small in-frame insertion or deletion. DNV – number of confirmed *de novo* cases. INH – number of confirmed maternally inherited cases. P – published. UP – unpublished (this study). M – males. F – Females. NA – sex not available. # – listed on ClinVar (search date 24.03.2025).

Supplementary Table 7. **Disease-causing missense variant discovery rate in *PDHA1* and other genes**.

**Gene**	***PDHA1***	***OTC***	***SLC6A8***	***TAFAZZIN***	***GLA***	***GAA***	***CFTR***	***PAH***
Associated disease	PDHc defiency	Urea cycle disorders	CCDS	Barth syndrome	Fabry disease	Pompe disease	Cystic fibrosis	Phenylketonuria
Gene location (CHCh38)	Xp22.1	Xp11.4	Xp14	Xq28	Xq22.1	17q25.3	7q31.2	12q23.2
Coding region lenght (nucleotides)	17792	68843	8663	10188	10123	18301	188641	80356
Protein length (amino acids)	390	354	635	262	429	952	1480	452
Missense variants among pathogenic variants	50%	63%	18%	0	61%	51%	39%	58%
Variant discovery (available / AlphaMissense)	13% (166/1277)	7% (264/3834)	0.4% (32/7970)	0	18% (605/3328)	4% (297/7342)	5% (819/13744)	15% (691/4565)
Reference	This study	148	ClinVar	ClinVar	149	150	151	152

Missense variant discovery was estimated as the proportion of known missense variants (from literature or ClinVar) over all possible missense variants predicted by AlphaMissense.[153] ClinVar data was accessed on 2025-03-23. Gene names (e.g., *PDHA1*, *GAA*) were used as search terms, and only variants classified as pathogenic or likely pathogenic and directly affecting the gene of interest were included (e.g., complex chromosomal rearrangements were excluded). AlphaMissense-predicted missense variants for each gene (e.g., *GLA*: UniProt ID P06280) were filtered for variants classified as likely pathogenic. Unique amino acid substitutions were included in the final count. "Missense variants among pathogenic variants" represents the proportion of missense mutations among all pathogenic variant types reported for each gene.
CCDS – Cerebral creatine deficiency syndrome.

Supplementary Table 8. **Coding variant distribution among *PDHA1* exons in the cohort and gnomAD database**

**Exon**	**1**	**2**	**3**	**4**	**5**	**6**	**7**	**8**	**9**	**10**	**11**
Base pairs (NM_000284.4)	1-57	58-117	118-291	292-418	419-510	511-603	604-759	760-831	832-899	900-1008	1009-1173
Number of cases	4	0	66	66	89	31	78	91	49	148	211
Number of variants	2	0	19	21	29	19	47	9	27	48	82
Nonsense	0	0	0	1	1	0	4	1	1	1	11
Frameshift	1	0	1	1	0	1	4	0	5	24	29
Missense	1	0	16	19	24	17	38	8	20	11	14
Indels	0	0	2	0	4	1	1	0	1	12	28
Frequency (all)	0.04	0	0.11	0.17	0.32	0.20	0.30	0.13	0.40	0.44	0.50
Frequency (males)	0	0	0.05	0.09	0.12	0.10	0.17	0.08	0.16	0.13	0.25
Frequency (females)	0.04	0	0.06	0.13	0.23	0.13	0.16	0.08	0.25	0.37	0.27
Number of variants (gnomAD)	16	17	18	19	4	7	18	13	4	28	47
Frequency (gnomAD)	0.28	0.28	0.10	0.15	0.04	0.08	0.12	0.18	0.06	0.26	0.28

Supplementary table 8 shows the distribution of coding variants among *PDHA1* exons. Frequency – variant frequency defined as variants/base pair.[154] The gnomAD dataset was filtered to include only rare variants with an allele frequency < 0.001 and exclude variants classified as “Benign” or “Likely benign” in ClinVar.[155] Additionally, non-coding variants were removed based on VEP annotations, including UTR variants, intronic or splice variants, and synonymous variants. The variants were assigned to exons based on their genomic positions corresponding to transcript ENST00000422285.7. Indels – small in-frame insertions or deletions.

Supplementary Table 9. **Comparison of sex-related enrichment for the most common variants (10 ≥ cases per variant)**

**Variant**	**Classification**	***No.* of cases (M/F/NA)**	**Expected (males)**	**Expected (females)**	**O/E ratio (males)**	**O/E ratio (females)**	**O/E male-to-female ratio**	***p*-value**
c.787C>G, p.Arg263Gly	Missense	68 (53/10/5)	30.603	35.183	1.732	0.284	6.093	< 0.001
c.904C>T, p.Arg302Cys	Missense	40 (2/36/2)	18.002	20.696	0.111	1.739	0.064	< 0.001
c.1133G>A, p.Arg378His	Missense	35 (24/10/1)	15.752	18.109	1.524	0.552	2.759	0.005
c.491A>G, p.Asn164Ser	Missense	33 (26/6/1)	14.852	17.074	1.751	0.351	4.982	< 0.001
c.1132C>T, p.Arg378Cys	Missense	30 (19/8/3)	13.502	15.522	1.407	0.515	2.730	0.017
c.1142_1145dup, p.Trp383SerfsTer6	Frameshift	30 (1/28/1)	13.502	15.522	0.074	1.804	0.041	< 0.001
c.934_940del, p.Ser312ValfsTer12	Frameshift	25 (0/24/1)	11.251	12.935	0	1.855	0	–
c.214C>T, p.Arg72Cys	Missense	23 (20/3/0)	10.351	11.9	1.932	0.252	7.664	< 0.001
c.1159_1162dup, p.Ser388Ter	Nonsense	22 (20/2/0)	9.901	11.383	2.019	0.176	11.496	< 0.001
c.262C>T, p.Arg88Cys	Missense	17 (14/2/1)	7.651	8.796	1.829	0.227	8.047	0.0014
c.379C>T, p.Arg127Trp	Missense	17 (9/8/0)	7.651	8.796	1.176	0.909	1.293	0.630
c.506C>T, p.Ala169Val	Missense	14 (4/10/0)	6.301	7.244	0.635	1.381	0.459	0.279
c.483C>T, p.?	Splice	13 (7/6/0)	5.851	6.726	1.196	0.892	1.341	0.781
c.380G>A, p.Arg127Gln	Missense	12 (2/10/0)	5.401	6.209	0.370	1.611	0.229	0.043
c.905G>A, p.Arg302His	Missense	11 (3/8/0)	4.951	5.691	0.605	1.406	0.431	0.236
c.787C>T, p.Arg263Ter	Nonsense	10 (2/8/0)	4.501	5.174	0.444	1.546	0.287	0.116

The table shows observed and expected counts of the variants (with ≥ 10 cases) in males and females. Expected counts were calculated as: total No. of variant × (proportion of cases in sex subgroups). Observed-to-expected (O/E) ratios reflect observed counts divided by expected counts, and the male-to-female O/E ratio highlights enrichment between sex subgroups.

Supplementary Table 10. **Comparison of published and unpublished cases by sex, presentation, last report, and survival (*n* = 316)**

**Characteristic**	**Published cases (*n* = 133)** (known alive or dead status and age at last report)	**Unpublished cases (*n* = 183)** (known alive or dead status and age at last report)	***p*-value**
Males, count	79 (59.4%)	65 (35.5%)	< 0.001
Age at last report, years (IQR)	1.8 (8.5)	6 (9.0)	< 0.001
Neonatal presentation, count	56/96 (58.3%)	82/180 (45.6%)	0.058
Infantile presentation, count	28/96 (29.2%)	64/180 (35.6%)	0.348
Childhood presentation, count	12/96 (12.5%)	34/180 (18.8%)	0.235
Deceased cases, count	106 (79.7%)	31 (16.9%)	< 0.001

Neonatal presentation – first presentation between 0-28 days. Infantile presentation – first presentation between 29 days - 12 months. Childhood presentation – first presentation between 1-13 years. CI – confidence interval. IQR – interquartile range. RMST – restricted mean survival time.

Supplementary Table 11. **Survival estimates stratified by maximum observation duration**

**Censoring threshold (years)**	**Number of cases**	**Percentage of selected subset (*n* = 278)**	**Deceased cases, count (%)**	**Median survival, years (IQR)**	**Restricted mean survival time, years (95% CI)**
[0-20]	251	90.3	101 (40.2)	16 (17.83)	11.7 (10.52 – 12.92)
[0-19]	245	88.1	100 (40.8)	16 (15.04)	11.3 (10.21 – 12.45)
[0-18]	242	87.1	100 (41.3)	13 (15.67)	10.9 (9.88 – 11.99)
[0-17]	237	85.3	99 (41.8)	12.5 (15.25)	10.5 (9.55 – 11.53)
[0-16]	231	83.1	98 (42.4)	11.2 (14.25)	10.1 (9.16 – 11.02)
[0-15]	224	80.6	94 (41.9)	11 (13.42)	9.6 (8.70 – 10.42)
[0-14]	219	78.8	94 (42.9)	9 (11.92)	9.0 (8.24 – 9.82)
[0-13]	216	77.7	94 (43.5)	8 (11.42)	8.5 (7.77 – 9.23)
[0-12]	210	75.5	92 (43.8)	8 (10.50)	7.9 (7.28 – 8.61)
[0-11]	201	72.3	91 (45.3)	7.8 (9.58)	7.4 (6.78 – 7.98)
[0-10]	196	70.5	90 (45.9)	6 (8.67)	6.8 (6.26 – 7.34)

Supplementary table 11 presents the number of cases, proportion of deaths, and survival metrics (median survival with interquartile range (IQR) and restricted mean survival time with 95% confidence intervals (CI) across different censoring thresholds from 10 to 20 years in selected cohort subset (includes cases with known survival status (alive or deceased), age at last report, sex, age at presentation). As the censoring threshold increases, the sample size and median survival rise, while the proportion of deceased cases remains relatively stable (~40–46%). Censoring up to 19 or 20 years inflates the median survival due to a small number of long-term survivors, as indicated by the large discrepancy between the median and restricted mean survival time (RMST). Censoring below 15 years leads to a substantial loss of cases (>20%), limiting the representativeness of the cohort. A censoring threshold of 18 years retains 87% of the selected cohort and 100 events (deaths), ensuring sufficient statistical power and compliance with the 10% rule for Kaplan–Meier analysis. Importantly, it marks the highest observation duration beyond which no substantial changes in median survival are observed, minimizing upward bias in survival estimates.

Supplementary Table 12. **Pairwise comparisons of sex and age at presentation from Cox PH model with Holm–Bonferroni adjustment**

**Pairwise comparisons**	**HR**	**1/HR**	**Estimate**	**SE**	***p*-value**
Infantile & Females (*n* = 40) vs. Infantile & Males (*n* = 41)	0.68	1.47	-0.39	0.225	0.173
Infantile & Females (*n* = 40) vs. Neonatal & Females (*n* = 80)	0.61	1.63	-0.49	0.195	0.036
Infantile & Females (*n* = 40) vs. Neonatal & Males (*n* = 48)	0.33	3.02	-1.11	0.218	< 0.001
Infantile & Males (*n* = 41) vs. Neonatal & Females (*n* = 80)	0.90	1.11	-0.10	0.193	0.597
Infantile & Males (*n* = 41) vs. Neonatal & Males (*n* = 48)	0.49	2.06	-0.72	0.215	0.004
Neonatal & Females (*n* = 80) vs. Neonatal & Males (*n* = 48)	0.54	1.86	-0.62	0.185	0.004

Supplementary table 12 provides details of survival multiple covariate analysis using Cox Proportional hazards (Cox PH) model with Holm–Bonferroni corrections (Concordance = 0.647 (standard error 0.02); Wald test = 27.23, $p<0.001$; Logrank test = 28.79, $p<0.001$). Model includes cases with known survival status (alive or deceased), age at last report ≤ 18 years, sex, age at presentation (childhood excluded, $n < 10$ per group). HR – Hazard ratio. 1/HR – reciprocal hazard ratio. SE – Standard error. Significant results are marked in bold. Infantile – age of presentation between 29 days - 12 months. Neonatal – age of presentation between 0-28 days.

Supplementary Table 13. **Univariate and multivariable Cox regression models of survivor up to 18 years (*n* = 240)**

Variable	Univariate model		Multivariable model (unadjusted)		Multivariable model (stepwise selection)	
	**HR (95% CI)**	***p*-value**	**HR (95% CI)**	***p*-value**	**HR (95% CI)**	***p*-value**
Exon (Exon 11 (*n* = 55) as reference)						
Exon 3 (*n* = 21)	0.22 (0.067, 0.731)	0.013	0.42 (0.114, 1.527)	0.187	Excluded	NA
Exon 4 (*n* = 14)	0.55 (0.192, 1.582)	0.269	0.83 (0.247, 2.78)	0.760	Excluded	NA
Exon 5 (*n* = 32)	0.62 (0.308, 1.26)	0.188	0.93 (0.397, 2.2)	0.877	Excluded	NA
Exon 6 (*n* = 10)	0.8 (0.279, 2.298)	0.679	0.53 (0.129, 2.171)	0.376	Excluded	NA
Exon 7 (*n* = 23)	0.86 (0.416, 1.778)	0.684	0.29 (0.121, 0.671)	0.004	Excluded	NA
Exon 8 (*n* = 23)	0.95 (0.491, 1.853)	0.889	0.58 (0.26, 1.273)	0.172	Excluded	NA
Exon 9 (*n* = 19)	1 (0.432, 2.304)	0.996	1.05 (0.39, 2.827)	0.924	Excluded	NA
Exon 10 (*n* = 43)	0.94 (0.52, 1.691)	0.831	0.68 (0.323, 1.423)	0.304	Excluded	NA
Variant type (Missense (*n* = 158) as reference)						
Indels (*n* = 17)	0.75 (0.323, 1.763)	0.515	0.75 (0.307, 1.837)	0.530	1.04 (0.444, 2.44)	0.926
Frameshift or Nonsense (NMD-escape) (*n* = 27)	0.87 (0.434, 1.762)	0.708	1.24 (0.506, 3.022)	0.642	1.81 (0.861, 3.797)	0.118
Frameshift or Nonsense (NMD-predicted) (*n* = 25)	1.4 (0.713, 2.735)	0.330	4.37 (1.856, 10.277)	0.001	4.04 (1.777, 9.163)	0.001
Splice (*n* = 13)	3.52 (1.786, 6.954)	< 0.001	2.76 (1.134, 6.708)	0.025	2.3 (1.152, 4.595)	0.018
Source (Published (*n* = 81) as reference)						
Unpublished (*n* = 159)	0.13 (0.086, 0.21)	< 0.001	0.15 (0.09, 0.265)	< 0.001	0.18 (0.107, 0.295)	< 0.001
Presentation (Childhood presentation (*n* = 29) as reference)						
Neonatal (*n* = 127)	5.5 (2.211, 13.68)	< 0.001	4.96 (1.786, 13.75)	0.002	5.53 (2.169, 14.087)	< 0.001
Infantile (*n* = 80)	2.09 (0.801, 5.476)	0.132	1.58 (0.563, 4.414)	0.387	1.85 (0.697, 4.931)	0.216
Sex (Females (*n* = 127) as reference)						
Males (*n* = 113)	2.53 (1.664, 3.86)	< 0.001	3.72 (2.005, 6.894)	< 0.001	3.31 (1.953, 5.617)	< 0.001

Analysis includes selected cohort subset (Supplementary Fig. 6) which includes cases with known survival status (alive or deceased), age at last report up to 18 years, sex, age at presentation (*n* = 240). Analysis excludes cases with variants in Exon 1 (does not fulfil ≥ 10 cases per comparison group, Exon, criteria). Longrank test of Multivariate model with stepwise selection $p < 0.001$. HR – hazard ratio. SE – standard error.

Supplementary Table 14. **Univariate and multivariable Cox regression models of survivor up to 18 years including prenatal or perinatal findings (*n* = 123)**

**Parameter**	**Univariate model**		**Multivariable model** (unadjusted)		**Multivariable model** (stepwise selection)	
	**HR (95% CI)**	***p*-value**	**HR (95% CI)**	***p*-value**	**HR (95% CI)**	***p*-value**
Exon (Exon 11 (*n* = 33) as reference)						
Exon 3 (excluded)						
Exon 4 (*n* = 10)	1.33 (0.274, 6.495)	0.721	1.75 (0.244, 12.603)	0.578	Excluded	NA
Exon 5 (*n* = 20)	0.69 (0.177, 2.668)	0.588	0.14 (0.026, 0.758)	0.023	Excluded	NA
Exon 6 (excluded)						
Exon 7 (*n* = 13)	0.49 (0.06, 4.007)	0.507	0.33 (0.03, 3.663)	0.370	Excluded	NA
Exon 8 (*n* = 12)	1.61 (0.471, 5.521)	0.447	0.56 (0.122, 2.609)	0.464	Excluded	NA
Exon 9 (*n* = 10)	0.74 (0.09, 6.095)	0.779	0.27 (0.02, 3.663)	0.327	Excluded	NA
Exon 10 (*n* = 25)	1.34 (0.448, 3.994)	0.602	0.3 (0.051, 1.745)	0.179	Excluded	NA
Variant type (Missense (*n* = 74) as reference)						
Indels (*n* = 11)	0.36 (0.048, 2.763)	0.329	0.05 (0.004, 0.784)	0.033	0.16 (0.018, 1.34)	0.090
Frameshift or Nonsense (NMD-escape) (*n* = 19)	0.44 (0.101, 1.926)	0.277	0.4 (0.057, 2.746)	0.349	0.73 (0.16, 3.32)	0.682
Frameshift or Nonsense (NMD-predicted) (*n* = 19)	1.77 (0.639, 4.891)	0.273	5.18 (1.104, 24.276)	0.037	3.01 (0.822, 11.048)	0.096
Splice (excluded, *n* < 10)						
Presentation (Childhood (*n* = 12) as reference)						
Neonatal (*n* = 66)	4.71 (0.619, 35.773)	0.134	6.69 (0.602, 74.42)	0.122	Excluded	NA
Infantile (*n* = 44)	2.86 (0.356, 22.884)	0.323	7.73 (0.808, 73.982)	0.076	Excluded	NA
Sex (Females (*n* = 83) as reference)						
Males (*n* = 40)	3.08 (1.366, 6.946)	0.007	16.46 (3.724, 72.785)	< 0.001	9.45 (3.047, 29.282)	< 0.001
Prenatal findings (Absent (*n* = 66) as reference)						
Prenatal findings (*n* = 57)	2.79 (1.205, 6.461)	0.017	2.03 (0.45, 9.127)	0.358	3.08 (1.044, 9.103)	0.042
Prematurity (Term (*n* = 106) as reference)						
Premature (*n* = 17)	4.59 (1.843, 11.436)	0.001	1.66 (0.506, 5.461)	0.402	Excluded	NA

Supplementary Table 14. **Univariate and multivariable Cox regression models of survivor up to 18 years including prenatal or perinatal findings (*n* = 123)** *(continued)*

**Parameter**	**Univariate model**		**Multivariable model** (unadjusted) (Concordance 0.84 (SE 0.027))		**Multivariable model** (stepwise selection) (Concordance 0.85 (SE 0.049))	
	**HR (95% CI)**	***p*-value**	**HR (95% CI)**	***p*-value**	**HR (95% CI)**	***p*-value**
Birth anthropometrics (> 3 *p*. (*n* = 83) as reference)						
Below 3 percentile (*n* = 40)	3.17 (1.38, 7.263)	0.007	0.9 (0.251, 3.241)	0.875	Excluded	NA
Resuscitation / APGAR < 5 (Absent (n =96) as reference)						
Present (*n* = 27)	4.24 (1.871, 9.62)	0.001	6.51 (1.796, 23.581)	0.004	4.38 (1.58, 12.121)	0.005

Analysis includes selected cohort subset (Supplementary Fig. 6) which includes cases with known survival status (alive or deceased), age at last report up to 18 years, sex, age at presentation. In addition, analysis included only cases with known prenatal findings, prematurity (born < 37 weeks) status, birth anthropometrics (weight, length, and (or) head circumference) status, resuscitation and (or) APGAR scores < 5 status, and excluded cases with variants in exons 1 and 6 (does not fulfil ≥ 10 cases per comparison group criteria). Cases with variants in exon 3 were all censored, thus were excluded from this sub-analysis. The following findings were considered as prenatal: prenatal movement abnormality (HP:0001557), intrauterine growth retardation (HP:0001511), polyhydramnios (HP:0001561), oligohydramnios (HP:0001562), abnormal fetal ultrasound or MRI. Longrank test of Multivariate model with stepwise selection p < 0.001. HR – hazard ratio. Data source (published or unpublished) was not included as covariate, as in this subset there were only seven unpublished cases. SE – standard error.

Supplementary Table 15. **Sex-related differences in activities of daily living**

**Activity of daily living**	**Unpublished among all cases with available data**	**Females**	**Males**	***p*-value (females *vs.* males)**
Independent sitting, % (*n*)	81.3% (200/246)	60.6% (83/137)	74.8% (89/119)	0.0006
Independent walking, % (*n*)	78.8% (178/226)	41.9% (52/124)	70.3% (71/101)	< 0.001
Independent eating, % (*n*)	87.4% (174/199)	40% (46/115)	71.4% (55/77)	< 0.001
Communication, % (*n*)	87.6% (149/170)	–	–	–
No communication, % (*n*)	–	27.1% (26/96)	6.8% (5/73)	0.002
Sounds only, % (*n*)	–	25.0% (24/96)	5.5% (4/73)	0.002
With words, % (*n*)	–	14.6% (14/96)	12.3% (9/73)	0.844
With sentences, % (*n*)	–	33.3% (32/96)	75.3% (55/73)	< 0.001
Independent personal hygiene, % (*n*)	77.9% (60/77)	32.6% (14/43)	55.9% (19/34)	0.068
School attendance, % (*n*)	76.9% (87/113)	–	–	–
Mainstream school, % (*n*)	–	18.0% (11/61)	49.0% (25/51)	0.001
School for children with special needs, % (*n*)	–	60.7% (37/61)	39.2% (20/51)	0.038

Table shows comparison of distribution of different activities of daily living between females and males. Analysis included cases with available sex and activities of daily living. Only cases with last known age of at least one year for sitting, two years for walking or eating, four years for communication,[156] eight years for school attendance, and 11 years for independent hygiene[157] were included.

Supplementary Table 16. **Clinical phenotypes among cases with no, mild, and severe developmental delay and (or) intellectual disability**

**Characteristics**	**No DD/ID (*n* = 36)**	**DD/ID (*n* = 464)**	***p*-value (no DD/ID *vs.* DD/ID)**	**Mild DD/ID (*n* = 39)**	**Moderate-severe DD/ID (*n* = 123)**	***p*-value (mild *vs.* severe)**
Sex (males), count	25/36 (69.4%)	176/456 (38.6%)	0.0006	23/39 (59.0%)	38/123 (30.9%)	0.002
Unpublished cases, count	28/36 (77.8%)	228/464 (49.1%)	0.002	34/39 (87.2%)	112/123 (91.1%)	0.539
Hypotonia (HP:0001252), count	12/31 (38.7%)	258/319 (80.9%)	< 0.001	26/39 (66.7%)	90/121 (74.4%)	0.41
Abnormality of movement, count	16/34 (47.1%)	179/272 (65.8%)	0.051	26/39 (66.7%)	59/112 (52.7%)	0.139
Feeding difficulties (HP:0011968), count	4/30 (13.3%)	131/240 (54.6%)	< 0.001	4/37 (10.8%)	72/121 (59.5%)	< 0.001
Seizures (HP:0001250), count	4/29 (13.8%)	166/273 (60.8%)	< 0.001	13/38 (34.2%)	74/121 (64.5%)	0.005
Microcephaly (HP:0000252), count	0/30	173/284 (61.3%)	–	6/34 (17.6%)	75/122 (61.5%)	< 0.001
Hypertonia (HP:0001276), count	4/30 (13.3%)	137/256 (53.5%)	< 0.001	11/37 (29.7%)	58/120 (48.3%)	0.04
Peripheral neuropathy (HP:0009830), count	9/30 (30%)	56/172 (32.6%)	0.948	11/34 (32.4%)	16/87 (18.4%)	0.143
Dysmorphic features (HP:0001999), count	0/28	88/241 (36.5%)	–	2/36 (5.6%)	43/118 (36.4%)	0.0003
Visual impairment (HP:0000505), count	2/29 (6.9%)	90/217 (41.5%)	0.0001	2/36 (5.6%)	49/111 (44.1%)	< 0.001
Strabismus (HP:0000486), count	5/29 (17.2%)	77/208 (37%)	0.059	10/36 (27.8%)	44/116 (37.9%)	0.321
Abnormal skeletal morphology (HP:0011842), count	0/29	71/219 (35.6%)	–	6/35 (17.1%)	38/118 (32.2%)	0.093
Hearing impairment (HP:0000365), count	1/29 (3.4%)	58/202 (28.7%)	0.002	1/34 (2.9%)	34/108 (31.5%)	0.0004
Drooling (HP:0002307), count	0/20	60/197 (30.5%)	–	2/34 (5.9%)	38/112 (33.9%)	0.0008
Nystagmus (HP:0000639), count	2/29 (6.9%)	39/206 (18.9%)	0.125	5/36 (13.9%)	19/116 (16.4%)	0.8
Ophthalmoplegia (HP:0000602), count	3/30 (10%)	28/209 (13.4%)	0.776	4/38 (10.5%)	12/113 (10.6%)	1

Supplementary table 16 provides clinical phenotypes distribution in cases with or without developmental delay (DD) and intellectual disability (ID), and cases with mild or moderate-severe DD/ID. Where applicable, HPO (Human Phenotype Ontology) codes are provided next to each clinical phenotype. Fisher's exact test was used for clinical phenotype frequency comparison in no DD/ID *vs.* DD/ID subgroups and in mild DD/ID *vs.* moderate-severe DD/ID (mild *vs.* severe). Phenotypes were grouped by similarity: abnormality of movement (HP:0004305, HP:0100022, HP:0001288, HP:0100660, HP:0001251, HP:0001332).

Supplementary Table 17. **Univariate and multivariable models of prenatal (fetal) findings (*n* = 292)**

**Parameter**	**Univariate model**		**Multivariable model** (unadjusted)		**Multivariable model** (stepwise selection)	
	**OR (95% CI)**	***P*-value**	**OR (95% CI)**	***P*-value**	**OR (95% CI)**	***P*-value**
Exon (Exon 11 (*n* = 64) as reference)						
Exon 3 (*n* = 26)	0.05 (0.002, 0.235)	0.003	0.07 (0.004, 0.403)	0.014	0.07 (0.004, 0.403)	0.014
Exon 4 (*n* = 20)	0.76 (0.264, 2.076)	0.590	0.83 (0.244, 2.781)	0.770	0.83 (0.244, 2.781)	0.770
Exon 5 (*n* = 44)	0.53 (0.233, 1.167)	0.120	0.59 (0.213, 1.632)	0.312	0.59 (0.213, 1.632)	0.312
Exon 6 (*n* = 14)	0.63 (0.177, 2.033)	0.449	0.63 (0.148, 2.446)	0.509	0.63 (0.148, 2.446)	0.509
Exon 7 (*n* = 32)	1.46 (0.623, 3.464)	0.387	1.52 (0.541, 4.371)	0.429	1.52 (0.541, 4.371)	0.429
Exon 8 (*n* = 21)	0.85 (0.307, 2.288)	0.749	1.08 (0.32, 3.604)	0.898	1.08 (0.32, 3.604)	0.898
Exon 9 (*n* = 18)	2.95 (0.986, 10.078)	0.064	2.15 (0.562, 8.929)	0.272	2.15 (0.562, 8.929)	0.272
Exon 10 (*n* = 53)	2.2 (1.049, 4.735)	0.039	1.37 (0.523, 3.638)	0.524	1.37 (0.523, 3.638)	0.524
Variant type (Missense (*n* = 180) as reference)						
Indels (*n* = 26)	1.55 (0.668, 3.566)	0.298	1.39 (0.535, 3.598)	0.497	1.39 (0.535, 3.598)	0.497
Frameshift or Nonsense (NMD-escape) (*n* = 31)	1.93 (0.895, 4.202)	0.092	1.37 (0.487, 3.89)	0.553	1.37 (0.487, 3.89)	0.553
Frameshift or Nonsense (NMD-predicted) (*n* = 39)	8.29 (3.649, 21.414)	< 0.001	3.92 (1.544, 11.035)	0.006	3.92 (1.544, 11.035)	0.006
Splice (*n* = 16)	2.33 (0.83, 6.802)	0.109	2.19 (0.665, 7.57)	0.202	2.19 (0.665, 7.57)	0.202
Source (Published (*n* = 57) as reference)						
Unpublished (*n* = 235)	0.49 (0.27, 0.883)	0.018	0.48 (0.241, 0.938)	0.034	0.48 (0.241, 0.938)	0.034
Sex (Females (*n* = 171) as reference)						
Males (*n* = 121)	0.32 (0.196, 0.526)	< 0.001	0.53 (0.29, 0.96)	0.038	0.53 (0.29, 0.96)	0.038

Analysis includes cases with available sex and prenatal status (present or absent). Cases with variants in Exons 1 were excluded (does not fulfil ≥ 10 cases per comparison group criteria). The following findings were considered as prenatal: prenatal movement abnormality (HP:0001557), intrauterine growth retardation (HP:0001511), polyhydramnios (HP:0001561), oligohydramnios (HP:0001562), abnormal fetal ultrasound or MRI. Multivariable model with stepwise selection was acceptable (Hosmer–Lemeshow p = 0.524) and had explained approximately 27% of variance (Nagelkerke pR^2 = 0.273). NA – not applicable.

Supplementary Table 18. **Univariate and multivariable models of prematurity (*n* = 246)**

Parameter	Univariate model		Multivariable model (unadjusted)		Multivariable model (stepwise selection)	
	**OR (95% CI)**	***P*-value**	**OR (95% CI)**	***P*-value**	**OR (95% CI)**	***P*-value**
Exon (Exon 11 (*n* = 56) as reference)						
Exon 3 (*n* = 24)	1.2 (0.293, 4.283)	0.785	1.33 (0.248, 6.961)	0.730	Excluded	NA
Exon 4 (*n* = 18)	1.2 (0.24, 4.768)	0.805	1.11 (0.168, 6.56)	0.907	Excluded	NA
Exon 5 (*n* = 37)	1.4 (0.449, 4.294)	0.553	1.08 (0.244, 4.83)	0.922	Excluded	NA
Exon 6 (excluded, *p*-10)						
Exon 7 (*n* = 27)	1.36 (0.376, 4.576)	0.620	0.82 (0.169, 3.874)	0.798	Excluded	NA
Exon 8 (*n* = 20)	1.06 (0.213, 4.154)	0.938	0.57 (0.089, 3.136)	0.528	Excluded	NA
Exon 9 (*n* = 15)	3 (0.774, 11.094)	0.100	2.16 (0.379, 12.293)	0.381	Excluded	NA
Exon 10 (*n* = 49)	0.68 (0.194, 2.2)	0.528	0.43 (0.091, 1.937)	0.270	Excluded	NA
Variant type (Missense (*n* = 148) as reference)						
Indels (*n* = 24)	0.98 (0.269, 2.878)	0.978	1.11 (0.247, 4.258)	0.879	Excluded	NA
Frameshift or Nonsense (NMD-escape) (*n* = 28)	0.38 (0.059, 1.383)	0.205	0.4 (0.048, 2.461)	0.349	Excluded	NA
Frameshift or Nonsense (NMD-predicted) (*n* = 34)	1.05 (0.364, 2.674)	0.916	1.1 (0.303, 3.778)	0.878	Excluded	NA
Splice (*n* = 12)	1.64 (0.346, 5.955)	0.481	1.78 (0.283, 9.101)	0.508	Excluded	NA
Source (Published (*n* = 37) as reference)						
Unpublished (*n* = 209)	0.8 (0.341, 2.125)	0.635	0.72 (0.27, 2.143)	0.537	Excluded	NA
Sex (Females (*n* = 146) as reference)						
Males (*n* = 100)	2.01 (1.015, 4.013)	0.046	3.44 (1.407, 8.848)	0.008	3.69 (1.71, 8.284)	0.001
Prenatal findings (Absent (*n* = 137) as reference)						
Prenatal findings (*n* = 109)	3.6 (1.769, 7.733)	0.001	6.62 (2.763, 17.322)	< 0.001	5.75 (2.61, 13.574)	< 0.001

Analysis includes cases with available sex, age at presentation, prematurity (born < 37 weeks of gestation) status (present or absent).). Cases with variants in exons 1 or 6 were excluded (does not fulfil ≥ 10 cases per comparison group criteria). The following findings were considered as prenatal: prenatal movement abnormality (HP:0001557), intrauterine growth retardation (HP:0001511), polyhydramnios (HP:0001561), oligohydramnios (HP:0001562), abnormal fetal ultrasound or MRI. Multivariable model with stepwise selection was acceptable (Hosmer–Lemeshow p = 0.094) and had explained approximately 16% of variance (Nagelkerke pR^2 = 0.158). NA – not applicable.

Supplementary Table 19. **Univariate and multivariable models of birth anthropometrics below 3rd percentile (*n* = 241)**

**Parameter**	**Univariate model**		**Multivariable model** (unadjusted)		**Multivariable model** (stepwise selection)	
	**OR (95% CI)**	***P*-value**	**OR (95% CI)**	***P*-value**	**OR (95% CI)**	***P*-value**
Exon (Exon 11 (*n* = 54) as reference)						
Exon 3 (*n* = 25)	0.3 (0.064, 1.01)	0.075	0.43 (0.07, 2.159)	0.320	Excluded	NA
Exon 4 (*n* = 17)	0.47 (0.098, 1.667)	0.276	0.29 (0.046, 1.571)	0.165	Excluded	NA
Exon 5 (*n* = 37)	0.42 (0.138, 1.151)	0.105	0.28 (0.063, 1.181)	0.088	Excluded	NA
Exon 6 (excluded, *n* < 10)						
Exon 7 (*n* = 26)	2.18 (0.835, 5.757)	0.112	1.26 (0.32, 5.122)	0.740	Excluded	NA
Exon 8 (*n* = 19)	0.78 (0.222, 2.409)	0.673	0.3 (0.056, 1.531)	0.156	Excluded	NA
Exon 9 (*n* = 15)	1.9 (0.582, 6.178)	0.279	0.46 (0.087, 2.415)	0.362	Excluded	NA
Exon 10 (*n* = 48)	1.43 (0.632, 3.247)	0.394	0.53 (0.148, 1.884)	0.326	Excluded	NA
Variant type (Missense (*n* = 146) as reference)						
Indels (*n* = 24)	0.59 (0.163, 1.68)	0.362	0.33 (0.073, 1.233)	0.120	0.44 (0.113, 1.397)	0.191
Frameshift or Nonsense (NMD-escape) (*n* = 27)	1.47 (0.587, 3.495)	0.390	0.66 (0.162, 2.669)	0.561	1.14 (0.409, 3.034)	0.797
Frameshift or Nonsense (NMD-predicted) (*n* = 32)	4.91 (2.221, 11.284)	< 0.001	2.6 (0.933, 7.526)	0.071	2.13 (0.874, 5.34)	0.099
Splice (*n* = 12)	0.98 (0.21, 3.494)	0.979	1.23 (0.217, 5.858)	0.798	0.81 (0.154, 3.399)	0.779
Source (Published (*n* = 34) as reference)						
Unpublished (*n* = 207)	1.24 (0.566, 2.954)	0.601	1.69 (0.651, 4.723)	0.295	Excluded	NA
Sex (Females (*n* = 145) as reference)						
Males (*n* = 96)	0.55 (0.301, 0.975)	0.044	1.54 (0.67, 3.592)	0.314	Excluded	NA
Prenatal findings (Absent (*n* = 136) as reference)						
Prenatal findings (*n* = 105)	9.95 (5.262, 19.83)	< 0.001	9.26 (4.455, 20.553)	< 0.001	8.7 (4.469, 17.806)	< 0.001

Analysis includes cases with available sex, age at presentation, birth anthropometrics below 3 percentile (included birth weight, length, or (and) head circumference < 3 percentile) status (present or absent).). Cases with variants in exons 1 or 6 were excluded (does not fulfil ≥ 10 cases per comparison group criteria). The following findings were considered as prenatal: prenatal movement abnormality (HP:0001557), intrauterine growth retardation (HP:0001511), polyhydramnios (HP:0001561), oligohydramnios (HP:0001562), abnormal fetal ultrasound or MRI. Multivariable model with stepwise selection was acceptable (Hosmer–Lemeshow p = 0.741) and had explained approximately 16% of variance (Nagelkerke $pR^2 = 0.324$). NA – not applicable.

Supplementary Table 20. **Univariate and multivariable models of resuscitation at birth and (or) APGAR scores ≤ 5 (*n* = 220)**

**Parameter**	**Univariate model**		**Multivariable model** (unadjusted)		**Multivariable model** (stepwise selection)	
	**OR (95% CI)**	***P*-value**	**OR (95% CI)**	***P*-value**	**OR (95% CI)**	***P*-value**
Exon (Exon 11 (*n* = 49) as reference)						
Exon 3 (*n* = 22)	0.42 (0.088, 1.478)	0.209	0.41 (0.054, 2.555)	0.351	0.57 (0.09, 2.838)	0.518
Exon 4 (*n* = 16)	0.4 (0.057, 1.683)	0.259	0.25 (0.023, 1.849)	0.200	0.33 (0.039, 1.724)	0.231
Exon 5 (*n* = 33)	0.75 (0.25, 2.086)	0.583	0.46 (0.084, 2.41)	0.364	0.8 (0.218, 2.721)	0.722
Exon 6 (excluded, *n* < 10)						
Exon 7 (*n* = 25)	0.53 (0.135, 1.715)	0.313	0.34 (0.058, 1.726)	0.200	0.41 (0.093, 1.519)	0.204
Exon 8 (*n* = 18)	0.15 (0.008, 0.868)	0.082	0.05 (0.002, 0.536)	0.028	0.08 (0.003, 0.594)	0.035
Exon 9 (*n* = 14)	1.11 (0.267, 3.982)	0.879	0.3 (0.034, 2.187)	0.255	0.29 (0.049, 1.489)	0.156
Exon 10 (*n* = 43)	0.95 (0.369, 2.424)	0.918	0.73 (0.173, 3.081)	0.669	0.73 (0.242, 2.125)	0.561
Variant type (Missense (*n* = 132) as reference)						
Indels (*n* = 23)	1.34 (0.41, 3.766)	0.597	0.65 (0.143, 2.555)	0.548	Excluded	NA
Frameshift or Nonsense (NMD-escape) (*n* = 24)	1.61 (0.536, 4.32)	0.364	0.7 (0.138, 3.446)	0.659	Excluded	NA
Frameshift or Nonsense (NMD-predicted) (*n* = 30)	1.47 (0.531, 3.701)	0.431	0.54 (0.131, 2.059)	0.383	Excluded	NA
Splice (*n* = 11)	2.76 (0.677, 9.934)	0.128	2.74 (0.439, 15.358)	0.258	Excluded	NA
Source (Published (*n* = 21) as reference)						
Unpublished (*n* = 199)	1.09 (0.379, 3.939)		1.46 (0.32, 8.522)	0.644	Excluded	NA
Sex (Females (*n* = 133) as reference)						
Males (*n* = 87)	1.02 (0.518, 1.988)	0.944	1.46 (0.538, 3.957)	0.456	Excluded	NA
Prenatal findings (Absent (*n* = 126) as reference)						
Prenatal findings (*n* = 94)	5.14 (2.536, 11.039)	< 0.001	6.06 (2.489, 16.213)	< 0.001	5.44 (2.415, 12.955)	< 0.001
Prematurity (Term (*n* = 188) as reference)						
Premature (*n* = 32)	9.11 (4.067, 21.094)	< 0.001	10.66 (4.006, 30.513)	< 0.001	8.92 (3.689, 22.578)	< 0.001
Birth anthropometrics (> 3 *p*. (*n* = 154) as reference)						
Below 3 percentile (*n* = 154)	2.53 (1.28, 4.985)	0.007	0.78 (0.296, 1.972)	0.599	Excluded	NA

Analysis includes cases with available sex, age at presentation, resuscitation at birth and (or) APGAR scores ≤ 5 status (present or absent).). Cases with variants in exons 1 or 6 were excluded (does not fulfil ≥ 10 cases per comparison group criteria). The following findings were considered as

prenatal: prenatal movement abnormality (HP:0001557), intrauterine growth retardation (HP:0001511), polyhydramnios (HP:0001561), oligohydramnios (HP:0001562), abnormal fetal ultrasound or MRI. Multivariable model with stepwise selection was acceptable (Hosmer–Lemeshow $p = 0.359$) and had explained approximately 34% of variance (Nagelkerke $pR^2 = 0.340$). NA – not applicable.

Supplementary Table 21. **Univariate and multivariable models of neonatal presentation in males (*n* = 252; compared to later presentations)**

**Parameter**	**Univariate model**		**Multivariable model** (unadjusted)		**Multivariable model** (stepwise selection)	
	**OR (95% CI)**	***P*-value**	**OR (95% CI)**	***P*-value**	**OR (95% CI)**	***P*-value**
Exon (Exon 11 (*n* = 58) as reference)						
Exon 3 (*n* = 36)	0.06 (0.009, 0.218)	< 0.001	0.04 (0.005, 0.148)	< 0.001	0.03 (0.005, 0.141)	< 0.001
Exon 4 (*n* = 11)	1.2 (0.326, 4.585)	0.782	0.75 (0.184, 3.098)	0.679	0.72 (0.179, 2.982)	0.644
Exon 5 (*n* = 35)	1.33 (0.575, 3.136)	0.504	0.75 (0.263, 2.094)	0.579	0.67 (0.246, 1.819)	0.438
Exon 6 (*n* = 13)	0.3 (0.062, 1.098)	0.089	0.15 (0.027, 0.617)	0.014	0.14 (0.026, 0.587)	0.012
Exon 7 (*n* = 31)	0.41 (0.155, 1.015)	0.060	0.22 (0.074, 0.632)	0.006	0.22 (0.074, 0.625)	0.005
Exon 8 (*n* = 40)	0.11 (0.03, 0.321)	< 0.001	0.07 (0.017, 0.222)	< 0.001	0.07 (0.017, 0.215)	< 0.001
Exon 9 (excluded, *n* < 10)						
Exon 10 (*n* = 20)	1.22 (0.441, 3.458)	0.700	0.92 (0.287, 3.038)	0.887	0.93 (0.293, 3.074)	0.908
Variant type (Missense (*n* = 186) as reference)						
Frameshift or Nonsense (NMD-escape) (*n* = 20)	1.19 (0.427, 3.061)	0.727	0.33 (0.099, 1.041)	0.064	0.32 (0.097, 1.009)	0.057
Frameshift or Nonsense (NMD-predicted) (excluded, *n* < 10)						
Indels (*n* = 20)	1.47 (0.55, 3.752)	0.424	0.49 (0.151, 1.505)	0.216	0.46 (0.143, 1.396)	0.175
Splice (*n* = 28)	3.47 (1.3, 9.855)	0.015	2.4 (0.771, 8.002)	0.138	2.53 (0.82, 8.414)	0.113
Source (Published (*n* = 152) as reference)						
Unpublished (*n* = 92)	0.75 (0.428, 1.297)	0.308	0.78 (0.4, 1.505)	0.464	Excluded	NA

Analysis includes males with available age at presentation. Cases with frameshift or nonsense variants in NMD-predicted region and with variants in Exons 1 and 9 were excluded (does not fulfil ≥ 10 cases per comparison group criteria). Model compared neonatal vs later presentation (infantile or at childhood). Multivariable model with stepwise selection was acceptable (Hosmer–Lemeshow p = 0.967) and had explained approximately 29% of variance (Nagelkerke $pR^2 = 0.293$). NA – not applicable.

Supplementary Table 22. **Univariate and multivariable models of neonatal presentation in females (*n* = 254; compared to later presentations)**

**Parameter**	**Univariate model**		**Multivariable model** (unadjusted)		**Multivariable model** (stepwise selection)	
	**OR (95% CI)**	***P*-value**	**OR (95% CI)**	***P*-value**	**OR (95% CI)**	***P*-value**
Exon (Exon 11 (*n* = 58) as reference)						
Exon 3 (*n* = 10)	0.2 (0.029, 0.896)	0.056	0.29 (0.037, 1.541)	0.176	0.29 (0.037, 1.541)	0.177
Exon 4 (*n* = 30)	0.62 (0.252, 1.504)	0.294	1.04 (0.333, 3.313)	0.944	1.04 (0.333, 3.313)	0.943
Exon 5 (*n* = 33)	0.35 (0.138, 0.856)	0.024	0.55 (0.168, 1.745)	0.307	0.55 (0.171, 1.731)	0.305
Exon 6 (excluded, *n* < 10)						
Exon 7 (*n* = 23)	0.89 (0.335, 2.357)	0.807	1.41 (0.419, 4.866)	0.580	1.42 (0.424, 4.843)	0.573
Exon 8 (excluded, *n* < 10)						
Exon 9 (*n* = 24)	1.62 (0.613, 4.563)	0.338	1.83 (0.517, 6.748)	0.355	1.83 (0.519, 6.746)	0.353
Exon 10 (*n* = 76)	2.82 (1.347, 6.044)	0.007	3.17 (1.16, 9.081)	0.027	3.17 (1.162, 9.083)	0.027
Variant type (Missense (*n* = 133) as reference)						
Frameshift or Nonsense (NMD-escape) (*n* = 35)	2 (0.94, 4.391)	0.077	2.44 (0.816, 7.619)	0.116	2.44 (0.821, 7.609)	0.114
Frameshift or Nonsense (NMD-predicted) (*n* = 49)	5.25 (2.451, 12.318)	< 0.001	3.01 (1.279, 7.619)	0.014	3.01 (1.279, 7.616)	0.014
Indels (*n* = 21)	1.92 (0.758, 5.133)	0.177	1.8 (0.626, 5.461)	0.282	1.8 (0.626, 5.461)	0.282
Splice (*n* = 16)	1.18 (0.411, 3.389)	0.754	1.36 (0.414, 4.547)	0.612	1.35 (0.418, 4.465)	0.613
Source (Published (*n* = 152) as reference)						
Unpublished (*n* = 102)	0.92 (0.556, 1.534)	0.762	1.01 (0.574, 1.786)	0.964	Excluded	NA

Analysis includes females with available age at presentation. Cases with variants in Exons 1, 6, and 8 were excluded (does not fulfil ≥ 10 cases per comparison group criteria). Model compared neonatal vs later presentation (infantile or childhood). Multivariable model with stepwise selection was acceptable (Hosmer–Lemeshow p = 0.5275) and had explained approximately 20% of variance (Nagelkerke $pR^2 = 0.203$). NA – not applicable.

Supplementary Table 23. **Prenatal (fetal), perinatal findings and age at presentation characteristics in males with most common variants (at least 10 cases per variant)**

**Variant**	**Age at presentation (months), median (IQR, n)**	**RCV_Q (age at presentation)**	**Age at presentation periods**			**Prenatal or perinatal findings, n (%)**
			**Neonatal, n (% of all)**	**Infantile, n (% of all)**	**Childhood, n (% of all)**	
c.787C>G, p.Arg263Gly (*n* = 53)	12 (8, *n* = 33)	0.503	3/35 (8.6%)	19/35 (54.3%)	13/35 (37.1%)	5/16 (31.2%)
c.491A>G, p.Asn164Ser (*n* = 26)	2 (3.82, *n* = 11)	1.432	7/15 (46.7%)	8/15 (53.3%)	0	1/12 (8.3%)
c.1133G>A, p.Arg378His (*n* = 24)	0.03 (0, *n* = 12)	0	14/15 (93.3%)	1/15 (6.7%)	0	2/8 (25%)
c.214C>T, p.Arg72Cys (*n* = 20)	24 (18, *n* = 15)	0.562	1/16 (6.2%)	7/16 (43.8%)	8/16 (50%)	1/8 (12.5%)
c.1159_1162dup, p.Ser388Ter (*n* = 20)	NA, *n* < 10	NA	1/9 (11.1%)	3/9 (33.3%)	5/9 (55.6%)	0/4
c.1132C>T, p.Arg378Cys (*n* = 19)	3.3 (3.12, *n* = 10)	0.709	3/11 (27.3%)	8/11 (72.7%)	0	5/7 (71.4%)
c.262C>T, p.Arg88Cys (*n* = 14)	24 (40.5, *n* = 11)	1.266	0	3/8 (37.5%)	5/8 (62.5%)	0/8

Analysis includes males with most common variants (at least 10 cases per variant). RCV_Q – Robust Coefficient of Variation based on IQR (inter-quartile range) ($RCV_Q = 0.75 * (IQR/median)$). RCV_Q values: 0-0.5 highly homogeneous; 0.5-1.0 – moderate heterogeneity; > 1.0 – high heterogeneity of age at presentation. The following findings were considered as prenatal: prenatal movement abnormality (HP:0001557), intrauterine growth retardation (HP:0001511), polyhydramnios (HP:0001561), oligohydramnios (HP:0001562), abnormal fetal ultrasound or MRI. The following findings were considered as perinatal: birth anthropometrics below 3 percentile (included birth weight, length, or (and) head circumference < 3 percentile), resuscitation at birth and (or) APGAR scores ≤ 5 status.

Supplementary Table 24. **Prenatal, perinatal findings and age at presentation characteristics in females with most common variants (at least 10 cases per variant)**

**Variant**	**Age at presentation (months), median (IQR, n)**	**RCV_Q (age at presentation)**	**Age at presentation periods (P)**			**Prenatal or perinatal findings, n (%)**
			**Neonatal, n (% of all)**	**Infantile, n (% of all)**	**Childhood, n (% of all)**	
c.904C>T, p.Arg302Cys (*n* = 36)	0.067 (2.967, *n* = 19)	33.213	14/21 (66.7%)	6/21 (28.6%)	1/21 (4.8%)	11/16 (68.8%)
c.1142_1145dup, p.Trp383SerfsTer6 (*n* = 28)	NA, *n* < 10	NA	10/15 (66.7%)	5/15 (33.3%)	0	5/10 (50%)
c.934_940del, p.Ser312ValfsTer12 (*n* = 24)	0.05 (0.592, *n* = 12)	8.88	12/14 (85.7%)	1/14 (7.1%)	1/14 (7.1%)	10/11 (90.9%)
c.506C>T, p.Ala169Val (*n* = 10)	NA, *n* < 10	NA	1/7 (14.3%)	6/7 (85.7%)	0	2/5 (40%)
c.787C>G, p.Arg263Gly (*n* = 10)	NA, *n* < 10	NA	0	0	6/6 (100%)	1/1 (100%)
c.1133G>A, p.Arg378His (*n* = 10)	NA, *n* < 10	NA	1/5 (20%)	2/5 (40%)	2/5 (40%)	1/5 (20%)

Analysis includes females with most common variants (at least 10 cases per variant). RCV_Q – Robust Coefficient of Variation based on IQR (inter-quartile range) (RCV_Q = 0.75 * (IQR/median)).[158] RCV_Q values: 0-0.5 highly homogeneous; 0.5-1.0 – moderate heterogeneity; > 1.0 – high heterogeneity of age at presentation. The following findings were considered as prenatal: prenatal movement abnormality (HP:0001557), intrauterine growth retardation (HP:0001511), polyhydramnios (HP:0001561), oligohydramnios (HP:0001562), abnormal fetal ultrasound or MRI. The following findings were considered as perinatal: birth anthropometrics below 3 percentile (included birth weight, length, or (and) head circumference < 3 percentile), resuscitation at birth and (or) APGAR scores ≤ 5 status.

Supplementary Table 25. **Univariate and multivariable Cox regression models of survivor in females up to 18 years (*n* = 106)**

Variable	Univariate model		Multivariable model (unadjusted)		Multivariable model (stepwise selection)	
	**HR (95% CI)**	***p*-value**	**HR (95% CI)**	***p*-value**	**HR (95% CI)**	***p*-value**
Source (Published (*n* = 26) as reference)						
Unpublished (*n* = 97)	0.18 (0.086, 0.381)	< 0.001	0.13 (0.057, 0.313)	< 0.001	0.13 (0.057, 0.313)	< 0.001
Variant type (Missense (*n* = 64) as reference)						
Indels (excluded, *n* < 10)						
Frameshift or Nonsense (NMD-escape) (*n* = 19)	1.14 (0.412, 3.166)	0.799	1.49 (0.529, 4.208)	0.449	1.49 (0.529, 4.208)	0.449
Frameshift or Nonsense (NMD-predicted) (*n* = 23)	2.49 (1.074, 5.776)	0.033	3.7 (1.394, 9.822)	0.009	3.7 (1.394, 9.822)	0.009
Splice (excluded, *n* < 10)						
Presentation (Infantile presentation (*n* = 34) as reference)						
Neonatal (*n* = 72)	5.75 (1.734, 19.042)	0.004	4.07 (1.179, 14.029)	0.026	4.07 (1.179, 14.029)	0.026

Analysis includes selected cohort subset (supplementary figure 6) which includes cases with known survival status (alive or deceased), age at last report up to 18 years, sex, age at presentation. In addition, analysis included only females and excluded cases with indels and splice variants, presentation in childhood or later (does not fulfil ≥ 10 cases per comparison group criteria) (*n* = 106). Longrank test of Multivariate model with stepwise selection p < 0.001. HR – hazard ratio. SE – standard error.

Supplementary Table 26. **Univariate and multivariable Cox regression models of survivor up to 18 years in males with missense variants (*n* = 73)**

Variable	Univariate model		Multivariable model (unadjusted)		Multivariable model (stepwise selection)	
	**HR (95% CI)**	***p*-value**	**HR (95% CI)**	***p*-value**	**HR (95% CI)**	***p*-value**
Exon (Exon 11 (*n* = 16) as reference)						
Exon 3 (*n* = 13)	0.05 (0.01, 0.211)	< 0.001	0.19 (0.036, 1.002)	0.050	0.19 (0.036, 1.002)	0.050
Exon 4 (excluded, *n* < 10)						
Exon 5 (*n* = 14)	0.21 (0.073, 0.579)	0.003	0.67 (0.178, 2.528)	0.556	0.67 (0.178, 2.528)	0.556
Exon 6 (excluded, *n* < 10)						
Exon 7 (*n* = 10)	0.36 (0.135, 0.976)	0.045	0.3 (0.109, 0.823)	0.019	0.3 (0.109, 0.823)	0.019
Exon 8 (*n* = 20)	0.24 (0.102, 0.542)	0.001	0.35 (0.138, 0.866)	0.023	0.35 (0.138, 0.866)	0.023
Exon 9 (excluded, *n* < 10)						
Exon 10 (excluded, *n* < 10)						
Source (Published (*n* = 33) as reference)						
Unpublished (*n* = 40)	0.19 (0.093, 0.388)	< 0.001	0.22 (0.081, 0.611)	0.004	0.22 (0.081, 0.611)	0.004
Presentation (Childhood presentation (*n* = 15) as reference)						
Neonatal (*n* = 29)	7.55 (2.556, 22.306)	< 0.001	3.03 (0.873, 10.541)	0.081	3.03 (0.873, 10.541)	0.081
Infantile (*n* = 29)	2.98 (0.988, 8.975)	0.053	1.63 (0.49, 5.396)	0.427	1.63 (0.49, 5.396)	0.427

Analysis includes selected cohort subset (supplementary figure 6) which includes cases with known survival status (alive or deceased), age at last report up to 18 years, sex, age at presentation. In addition, analysis included only males and excluded cases with variants in Exons 1, 4, 6, 9, 10, splice, indels, and frameshift or nonsense variants (does not fulfil ≥ 10 cases per comparison group criteria) (*n* = 73). Longrank test of Multivariate model with stepwise selection p < 0.001. HR – hazard ratio. SE – standard error.

Supplementary Table 27. **Univariate and multivariable models of developmental delay (*n* = 363)**

**Parameter**	**Univariate model**		**Multivariable model** (unadjusted)		**Multivariable model** (stepwise selection)	
	**OR (95% CI)**	***P*-value**	**OR (95% CI)**	***P*-value**	**OR (95% CI)**	***P*-value**
Exon (Exon 11 (*n* = 80) as reference)						
Exon 3 (*n* = 33)	0.1 (0.034, 0.276)	< 0.001	0.14 (0.025, 0.61)	0.014	0.21 (0.064, 0.667)	0.009
Exon 4 (*n* = 33)	0.7 (0.195, 2.818)	0.584	0.31 (0.047, 1.821)	0.199	0.49 (0.116, 2.246)	0.333
Exon 5 (*n* = 38)	0.82 (0.23, 3.284)	0.757	0.36 (0.057, 2.048)	0.254	0.56 (0.142, 2.396)	0.404
Exon 6 (*n* = 15)	1.34 (0.214, 26.112)	0.790	1.03 (0.094, 24.78)	0.985	1.61 (0.211, 34.254)	0.689
Exon 7 (*n* = 45)	1.34 (0.353, 6.483)	0.681	0.89 (0.132, 5.83)	0.897	1.38 (0.324, 7.262)	0.675
Exon 8 (*n* = 38)	1.12 (0.292, 5.429)	0.876	1.79 (0.265, 12.082)	0.539	2.68 (0.636, 14.13)	0.199
Exon 9 (*n* = 18)	1.63 (0.264, 31.526)	0.658	1.02 (0.091, 24.691)	0.991	1.45 (0.18, 31.45)	0.758
Exon 10 (*n* = 63)	1.11 (0.338, 3.928)	0.862	0.51 (0.089, 2.763)	0.439	0.53 (0.135, 2.116)	0.355
Variant type (Missense (*n* = 256) as reference)						
Indels (*n* = 24)	0.54 (0.202, 1.726)	0.255	0.49 (0.115, 2.298)	0.343	Excluded	NA
Frameshift or Nonsense (NMD-escape) (*n* = 38)	1.21 (0.446, 4.263)	0.729	0.47 (0.075, 2.775)	0.406	Excluded	NA
Frameshift or Nonsense (NMD-predicted) (*n* = 45)	2 (0.675, 8.586)	0.269	0.54 (0.115, 3.044)	0.449	Excluded	NA
Splice	All cases (*n* = 20) had developmental delay. Excluded from sub-analysis					
Source (Published (*n* = 137) as reference)						
Unpublished (*n* = 226)	0.94 (0.477, 1.78)	0.841	1.03 (0.454, 2.326)	0.934	Excluded	NA
Sex (Females (*n* = 207) as reference)						
Males (*n* = 156)	0.24 (0.s114, 0.469)	< 0.001	0.31 (0.121, 0.728)	0.009	0.32 (0.132, 0.726)	0.008
Presentation (Childhood (*n* = 67) as reference)						
Neonatal (*n* = 143)	13.59 (5.543, 38.62)	< 0.001	9.16 (3.212, 29.573)	< 0.001	9.54 (3.445, 29.849)	< 0.001
Infantile (*n* = 153)	6.41 (3.067, 13.979)	< 0.001	6.19 (2.665, 15.026)	< 0.001	6.39 (2.796, 15.3)	< 0.001

Analysis includes cases with available sex, age at presentation, developmental delay status (present or absent).). Cases with variants in Exons 1 were excluded (does not fulfil ≥ 10 cases per comparison group criteria). Cases with splice variants were exclude, as all had developmental delay. Multivariable model with stepwise selection was acceptable (Hosmer–Lemeshow p = 0.252) and had explained approximately 33% of variance (Nagelkerke $pR^2 = 0.33$). NA – not applicable.

Supplementary Table 28. **Univariate and multivariable models of intellectual disability (*n* = 215)**

**Parameter**	**Univariate model**		**Multivariable model** (unadjusted)		**Multivariable model** (stepwise selection)	
	**OR (95% CI)**	***P*-value**	**OR (95% CI)**	***P*-value**	**OR (95% CI)**	***P*-value**
Exon (Exon 11 (*n* = 50) as reference)						
Exon 3 (*n* = 24)	0.11 (0.03, 0.359)	< 0.001	0.18 (0.027, 0.957)	0.055	Excluded	NA
Exon 4 (*n* = 18)	0.89 (0.172, 6.634)	0.894	0.72 (0.077, 7.751)	0.772	Excluded	NA
Exon 5 (*n* = 28)	1.44 (0.288, 10.586)	0.673	0.45 (0.048, 4.576)	0.472	Excluded	NA
Exon 6 (excluded, *n* < 10)						
Exon 7 (*n* = 23)	0.74 (0.165, 3.89)	0.700	0.39 (0.048, 2.943)	0.358	Excluded	NA
Exon 8 (*n* = 22)	0.7 (0.156, 3.705)	0.652	1.11 (0.136, 8.994)	0.920	Excluded	NA
Exon 9 (*n* = 13)	0.61 (0.114, 4.657)	0.585	0.75 (0.074, 8.471)	0.805	Excluded	NA
Exon 10 (*n* = 37)	0.92 (0.226, 3.95)	0.902	0.53 (0.072, 3.569)	0.510	Excluded	NA
Variant type (Missense (*n* = 149) as reference)						
Indels (*n* = 17)	0.66 (0.211, 2.473)	0.490	0.63 (0.126, 3.47)	0.579	Excluded	NA
Frameshift or Nonsense (NMD-escape) (*n* = 26)	2.42 (0.658, 15.654)	0.250	1.03 (0.113, 11.316)	0.979	Excluded	NA
Frameshift or Nonsense (NMD-predicted) (*n* = 23)	2.12 (0.57, 13.761)	0.331	0.31 (0.045, 2.756)	0.249	Excluded	NA
Splice (excluded, *n* < 10)						
Source (Published (*n* = 51) as reference)						
Unpublished (*n* = 164)	2.46 (1.105, 5.366)	0.024	3.69 (1.312, 10.936)	0.015	2.87 (1.127, 7.402)	0.027
Sex (Females (*n* = 128) as reference)						
Males (*n* = 87)	0.33 (0.146, 0.693)	0.004	0.88 (0.297, 2.602)	0.810	Excluded	NA
Presentation (Childhood (*n* = 49) as reference)						
Neonatal (*n* = 78)	16.37 (5.674, 59.876)	< 0.001	18.6 (4.475, 99.976)	< 0.001	16.61 (5.653, 61.826)	< 0.001
Infantile (*n* = 88)	12.09 (4.694, 35.721)	< 0.001	13.34 (4.394, 47.1)	< 0.001	13.36 (5.039, 40.936)	< 0.001

Analysis includes cases with available sex, age at presentation, intellectual disability status (present or absent). Cases with variants in exons 1, 6 or splice variants were excluded (does not fulfil ≥ 10 cases per comparison group criteria). Multivariable model with stepwise selection was acceptable (Hosmer–Lemeshow p = 0.456) and had explained approximately 34% of variance (Nagelkerke $pR^2 = 0.335$). NA – not applicable.

Supplementary Table 29. **Variants in cases with no developmental delay and no intellectual disability (variants *n* = 27, cases *n* = 36)**

Variant	Type	Gene region	No. of cases with no DD/ID	No. of cases in the cohort	ACMG	CADD	Residual enzyme activity in fibroblasts, median (IQR, n)
c.193_195delinsCAA, p.Tyr65Gln	Indel	Exon 3	1	1	P	26.0	NA
c.194A>C, p.Tyr65Ser	Missense	Exon 3	1	1	LP	27.3	NA
c.212T>C, p.Val71Ala	Missense	Exon 3	1	1	LP	25.5	28 (*n* = 1)
c.214C>T, p.Arg72Cys	Missense	Exon 3	1	23	P	29.0	32 (IQR 13.2, *n* = 8)
c.262C>T, p.Arg88Cys	Missense	Exon 3	8	17	P	31.0	40 (*n* = 1)
c.380G>A, p.Arg127Gln	Missense	Exon 4	1	12	P	26.8	50 (IQR 42.2, *n* = 7)
c.407C>T, p.Ala136Val	Missense	Exon 4	1	3	LP	25.7	NA
c.478_479delTTinsAA, p.Phe160Asn	Missense	Exon 5	1	1	LP	27.4	NA
c.491A>G, p.Asn164Ser	Missense	Exon 5	1	33	LP	26.1	25 (IQR 36.8, *n* = 11)
c.506C>T, p.Ala169Val	Missense	Exon 5	1	14	LP	26.0	36.5 (IQR 18.2, *n* = 4)
c.628A>G, p.Met210Val	Missense	Exon 7	1	6	P	24.8	Min-max, 14.8-40.2 (*n* = 2)
c.647T>C, p.Leu216Ser	Missense	Exon 7	1	2	P	27.7	NA
c.687G>A, p.Met229Ile	Missense	Exon 7	1	2	P	27.7	NA
c.787C>G, p.Arg263Gly	Missense	Exon 8	2	68	P	23.5	43.5 (IQR 35.8, *n* = 30)
c.821G>C, p.Arg274Thr	Missense	Exon 8	1	2	LP	24.8	NA
c.784G>C, p.Val262Leu	Missense	Exon 8	1	1	LP	25.5	NA
c.833G>A, p.Gly278Glu	Missense	Exon 9	1	1	LP	33	NA
c.892G>A, p.Gly298Arg	Missense	Exon 9	1	1	LP	33	NA
c.910C>T, p.Arg304Ter	Nonsense (NMD-predicted)	Exon 10	1	6	P	38	32 (*n* = 1)
c.963_977dup, p.Lys321_Val325dup	Indel	Exon 10	1	5	P	18.4	61.9 (*n* = 1)
c.913_929dup, p.Arg311LysfsTer6	Frameshift (NMD-predicted)	Exon 10	1	1	P	34	NA
c.1006_1008dup, p.Lys336dup	Indel	Exon 10	1	1	P	17.0	NA
c.1159_1162dup, p.Ser388Ter	Nonsense (NMD-escape)	Exon 11	2	22	P	34.0	25 (IQR 13, *n* = 13)
c.1167_1170del, p.Ser390LysfsTer33	Frameshift (NMD-escape)	Exon 11	1	3	P	33.0	NA
c.1064_1065insTAAG, p.Asp356LysfsTer4	Frameshift (NMD-escape)	Exon 11	1	1	P	33.0	NA
c.1087_1119dup, p.Glu363_Pro373dup	Indel	Exon 11	1	1	P	22.5	18.6 (*n* = 1)
c.1162T>C, p.Ser388Pro	Missense	Exon 11	1	1	LP	25.5	NA

The most commonly affected gene regions in cases with no DD/ID: exon 3 (12/36, 33.3%) and exon 11 (6/36, 16.7%). None of the case with no DD/ID harbored splice variants, compared with 26 cases with DD/ID. Variant type distribution was similar between cases with and without DD/ID ($p = 0.517$). In total, 44.4% (12/27) of variants in cases without DD/ID were private (vs. 45.9% (146/318) in cases with DD/ID, $p = 0.234$). Regions between p.Met1 to p.Ala34 and p.Leu319 to p.Ser390 were considered as regions predicted to escape nonsense-mediated decay (NMD-escape) and the remaining as NMD-predicted regions.

Supplementary Table 30. **Clinical phenotype homogeneity in males with the most common variants (at least 10 cases per variant)**

**Clinical findings**	**c.787C>G, p.Arg263Gly (*n* = 53)**	**c.491A>G, p.Asn164Ser (*n* = 26)**	**c.1133G>A, p.Arg378His (*n* = 24)**	**c.214C>T, p.Arg72Cys (*n* = 20)**	**c.1159_1162dup, p.Ser388Ter (*n* = 20)**	**c.1132C>T, p.Arg378Cys (*n* = 19)**	**c.262C>T, p.Arg88Cys (*n* = 14)**
Developmental delay (HP:0012758), count (%)	24/25 (96%); H = 0.242; p < 0.001	12/14 (85.7%); H = 0.592; p = 0.013	8/9 (88.9%); H = 0.503; p = 0.039	9/12 (75%); H = 0.811; p = 0.146	3/7 (42.9%); H = 0.985; p = 1	9/9 (100%); H = 0; p = 0.004	1/12 (8.3%); H = 0.414; p = 0.006
Intellectual disability (HP:0001249), count (%)	16/19 (84.2%); H = 0.629; p = 0.004	11/13 (84.6%); H = 0.619; p = 0.022	6/7 (85.7%); H = 0.592; p = 0.125	7/10 (70%); H = 0.881; p = 0.344	4/10 (40%); H = 0.971; p = 0.754	8/8 (100%); H = 0; p = 0.008	1/9 (11.1%); H = 0.503; p = 0.039
Muscle hypotonia (HP:0001252), count (%)	27/30 (90%); H = 0.469; p < 0.001	8/13 (61.5%); H = 0.961; p = 0.581	11/12 (91.7%); H = 0.414; p = 0.006	9/12 (75%); H = 0.811; p = 0.146	2/4 (50%); H = 1; p = 1	10/10 (100%); H = 0; p = 0.002	3/11 (27.3%); H = 0.845; p = 0.227
Abnormal movements, count (%)	27/31 (87.1%); H = 0.555; p < 0.001	6/13 (46.2%); H = 0.996; p = 1	2/4 (50%); H = 1; p = 1	12/14 (85.7%); H = 0.592; p = 0.013	11/12 (91.7%); H = 0.414; p = 0.006	5/6 (83.3%); H = 0.65; p = 0.219	0/9 (0%); H = 0; p = 0.004
Seizures (HP:0001250), count (%)	6/15 (40%); H = 0.971; p = 0.607	6/14 (42.9%); H = 0.985; p = 0.791	7/8 (87.5%); H = 0.544; p = 0.07	3/10 (30%); H = 0.881; p = 0.344	1/4 (25%); H = 0.811; p = 0.625	5/6 (83.3%); H = 0.65; p = 0.219	0/9 (0%); H = 0; p = 0.004
Microcephaly (HP:0000252), count (%)	4/17 (23.5%); H = 0.787; p = 0.049	1/12 (8.3%); H = 0.414; p = 0.006	1/4 (25%); H = 0.811; p = 0.625	1/9 (11.1%); H = 0.503; p = 0.039	1/4 (25%); H = 0.811; p = 0.625	3/6 (50%); H = 1; p = 1	0/10 (0%); H = 0; p = 0.002
Feeding difficulties (HP:0011968), count (%)	5/14 (35.7%); H = 0.94; p = 0.424	7/12 (58.3%); H = 0.98; p = 0.774	3/6 (50%); H = 1; p = 1	1/8 (12.5%); H = 0.544; p = 0.07	0/3 (0%); H = 0; p = 0.25	5/6 (83.3%); H = 0.65; p = 0.219	1/10 (10%); H = 0.469; p = 0.021
Muscle hypertonia (HP:0001276), count (%)	7/16 (43.8%); H = 0.989; p = 0.804	4/13 (30.8%); H = 0.89; p = 0.267	2/5 (40%); H = 0.971; p = 1	3/9 (33.3%); H = 0.918; p = 0.508	0/3 (0%); H = 0; p = 0.25	3/6 (50%); H = 1; p = 1	2/11 (18.2%); H = 0.684; p = 0.065
Peripheral neuropathy (HP:0009830), count (%)	14/18 (77.8%); H = 0.764; p = 0.031	2/10 (20%); H = 0.722; p = 0.109	0/2 (0%); H = 0; p = 0.5	6/11 (54.5%); H = 0.994; p = 1	2/2 (100%); H = 0; p = 0.5	0/5 (0%); H = 0; p = 0.062	5/11 (45.5%); H = 0.994; p = 1
Visual impairment (HP:0000505), count (%)	1/13 (7.7%); H = 0.391; p = 0.003	4/13 (30.8%); H = 0.89; p = 0.267	2/4 (50%); H = 1; p = 1	0/8 (0%); H = 0; p = 0.008	1/4 (25%); H = 0.811; p = 0.625	3/6 (50%); H = 1; p = 1	0/9 (0%); H = 0; p = 0.004
Dysmorphic features (HP:0001999), count (%)	3/16 (18.8%); H = 0.696; p = 0.021	1/12 (8.3%); H = 0.414; p = 0.006	3/6 (50%); H = 1; p = 1	0/8 (0%); H = 0; p = 0.008	0/3 (0%); H = 0; p = 0.25	1/5 (20%); H = 0.722; p = 0.375	0/9 (0%); H = 0; p = 0.004

Supplementary table 30 corresponds to supplementary figure 11A. Only males with the most common variants were included (≥ 10 cases per variant). Each row shows the phenotype frequency within males with the same variant (represented in columns). For each phenotype prevalence with variant subgroup Shannon entropy (H, as log2 values) was determined. *P*-value represents Binomial test results, which indicate whether deviation from a theoretical equal distribution of a phenotype was significant. Abnormal movements include these phenotypes: HP:0004305, HP:0100022, HP:0001288, HP:0100660, HP:0001251, HP:0001332.

Supplementary Table 31. **Clinical phenotype homogeneity in females with the most common variants (at least 10 cases per variant)**

**Clinical findings**	**c.904C>T, p.Arg302Cys (*n* = 36)**	**c.1142_1145dup, p.Trp383SerfsTer 6 (*n* = 28)**	**c.934_940del, p.Ser312ValfsTer 12 (*n* = 24)**	**c.380G>A, p.Arg127Gln (*n* = 10)**	**c.506C>T, p.Ala169Val (*n* = 10)**	**c.1133G>A, p.Arg378His (*n* = 10)**
Developmental delay (HP:0012758), count (%)	19/19 (100%); H = 0; p < 0.001	13/13 (100%); H = 0; p < 0.001	14/14 (100%); H = 0; p < 0.001	5/6 (83.3%); H = 0.65; p = 0.219	6/6 (100%); H = 0; p = 0.031	9/9 (100%); H = 0; p = 0.004
Intellectual disability (HP:0001249), count (%)	14/14 (100%); H = 0; p < 0.001	9/9 (100%); H = 0; p = 0.004	9/9 (100%); H = 0; p = 0.004	3/4 (75%); H = 0.811; p = 0.625	4/4 (100%); H = 0; p = 0.125	4/5 (80%); H = 0.722; p = 0.375
Muscle hypotonia (HP:0001252), count (%)	10/15 (66.7%); H = 0.918; p = 0.302	8/10 (80%); H = 0.722; p = 0.109	12/14 (85.7%); H = 0.592; p = 0.013	3/3 (100%); H = 0; p = 0.25	5/6 (83.3%); H = 0.65; p = 0.219	5/5 (100%); H = 0; p = 0.062
Abnormal movements, count (%);	7/11 (63.6%); H = 0.946; p = 0.549	5/8 (62.5%); H = 0.954; p = 0.727	4/6 (66.7%); H = 0.918; p = 0.687	4/6 (66.7%); H = 0.918; p = 0.687	3/5 (60%); H = 0.971; p = 1	6/7 (85.7%); H = 0.592; p = 0.125
Seizures (HP:0001250), count (%)	11/15 (73.3%); H = 0.837; p = 0.118	6/10 (60%); H = 0.971; p = 0.754	6/10 (60%); H = 0.971; p = 0.754	2/3 (66.7%); H = 0.918; p = 1	5/6 (83.3%); H = 0.65; p = 0.219	2/4 (50%); H = 1; p = 1
Microcephaly (HP:0000252), count (%)	15/19 (78.9%); H = 0.742; p = 0.019	12/14 (85.7%); H = 0.592; p = 0.013	10/11 (90.9%); H = 0.439; p = 0.012	3/4 (75%); H = 0.811; p = 0.625	4/5 (80%); H = 0.722; p = 0.375	1/4 (25%); H = 0.811; p = 0.625
Feeding difficulties (HP:0011968), count (%)	10/15 (66.7%); H = 0.918; p = 0.302	7/9 (77.8%); H = 0.764; p = 0.18	6/9 (66.7%); H = 0.918; p = 0.508	1/2 (50%); H = 1; p = 1	4/5 (80%); H = 0.722; p = 0.375	0/4 (0%); H = 0; p = 0.125
Muscle hypertonia (HP:0001276), count (%)	8/13 (61.5%); H = 0.961; p = 0.581	4/8 (50%); H = 1; p = 1	7/9 (77.8%); H = 0.764; p = 0.18	3/4 (75%); H = 0.811; p = 0.625	3/5 (60%); H = 0.971; p = 1	3/6 (50%); H = 1; p = 1
Peripheral neuropathy (HP:0009830), count (%)	1/8 (12.5%); H = 0.544; p = 0.07	1/3 (33.3%); H = 0.918; p = 1	0/5 (0%); H = 0; p = 0.062	2/3 (66.7%); H = 0.918; p = 1	0/2 (0%); H = 0; p = 0.5	0/4 (0%); H = 0; p = 0.125
Visual impairment (HP:0000505), count (%)	4/10 (40%); H = 0.971; p = 0.754	5/6 (83.3%); H = 0.65; p = 0.219	3/6 (50%); H = 1; p = 1	0/2 (0%); H = 0; p = 0.5	4/6 (66.7%); H = 0.918; p = 0.687	0/4 (0%); H = 0; p = 0.125
Dysmorphic features (HP:0001999), count (%)	7/13 (53.8%); H = 0.996; p = 1	6/9 (66.7%); H = 0.918; p = 0.508	8/11 (72.7%); H = 0.845; p = 0.227	0/2 (0%); H = 0; p = 0.5	1/5 (20%); H = 0.722; p = 0.375	2/4 (50%); H = 1; p = 1

Supplementary table 31 corresponds to supplementary figure 11B. Only females with the most common variants were included (≥ 10 cases per variant). Each row shows the phenotype frequency within males with the same variant (represented in columns). For each phenotype prevalence with variant subgroup Shannon entropy (H, as log2 values) was determined. *P*-value represents Binomial test results, which indicate whether deviation from a theoretical equal distribution of a phenotype was significant. Abnormal movements include these phenotypes: HP:0004305, HP:0100022, HP:0001288, HP:0100660, HP:0001251, HP:0001332.

Supplementary Table 32. **Univariate and multivariable models of muscle hypotonia (*n* = 400)**

**Parameter**	**Univariate model**		**Multivariable model** (unadjusted)		**Multivariable model** (stepwise selection)	
	**OR (95% CI)**	***P*-value**	**OR (95% CI)**	***P*-value**	**OR (95% CI)**	***P*-value**
Exon (Exon 11 (*n* = 85) as reference)						
Exon 3 (*n* = 41)	0.26 (0.095, 0.655)	0.005	0.1 (0.017, 0.43)	0.004	0.17 (0.056, 0.489)	0.001
Exon 4 (*n* = 25)	0.38 (0.12, 1.236)	0.094	0.21 (0.036, 1.062)	0.069	0.42 (0.123, 1.465)	0.161
Exon 5 (*n* = 52)	0.21 (0.081, 0.49)	0.001	0.11 (0.021, 0.421)	0.003	0.27 (0.102, 0.681)	0.007
Exon 6 (*n* = 16)	0.2 (0.058, 0.692)	0.009	0.09 (0.013, 0.496)	0.008	0.18 (0.045, 0.72)	0.015
Exon 7 (*n* = 46)	0.79 (0.265, 2.501)	0.674	0.34 (0.06, 1.627)	0.194	0.72 (0.228, 2.415)	0.584
Exon 8 (*n* = 41)	0.58 (0.198, 1.729)	0.310	0.3 (0.052, 1.433)	0.149	0.58 (0.186, 1.834)	0.340
Exon 9 (*n* = 25)	0.3 (0.1, 0.952)	0.036	0.17 (0.028, 0.813)	0.033	0.33 (0.097, 1.098)	0.067
Exon 10 (*n* = 69)	0.36 (0.144, 0.857)	0.024	0.21 (0.042, 0.763)	0.028	0.39 (0.146, 0.965)	0.046
Variant type (Missense (*n* = 260) as reference)						
Indels (*n* = 30)	0.84 (0.37, 2.104)	0.697	0.7 (0.222, 2.181)	0.528	Excluded	NA
Frameshift or Nonsense (NMD-escape) (*n* = 37)	1.31 (0.578, 3.386)	0.539	0.38 (0.07, 1.673)	0.221	Excluded	NA
Frameshift or Nonsense (NMD-predicted) (*n* = 46)	0.98 (0.48, 2.118)	0.947	1.17 (0.461, 3.066)	0.749	Excluded	NA
Splice (*n* = 27)	2.45 (0.821, 10.565)	0.154	2.27 (0.619, 10.972)	0.248	Excluded	NA
Sex (Females (*n* = 211) as reference)						
Males (*n* = 189)	1.55 (0.965, 2.52)	0.072	1.39 (0.735, 2.642)	0.316	Excluded	NA
Source (Published (*n* = 164) as reference)						
Unpublished (*n* = 236)	0.06 (0.019, 0.128)	< 0.001	0.06 (0.02, 0.146)	< 0.001	0.05 (0.018, 0.125)	< 0.001
Presentation (Childhood (*n* = 67) as reference)						
Neonatal (*n* = 175)	1.89 (1, 3.526)	0.047	1.97 (0.84, 4.627)	0.118	Excluded	NA
Infantile (*n* = 158)	1.92 (1.008, 3.647)	0.045	2.15 (0.965, 4.829)	0.062	Excluded	NA

Analysis includes cases with available sex, age at presentation, muscle hypotonia status (present or absent). Cases with variants in Exons 1 were excluded (does not fulfil ≥ 10 cases per comparison group criteria). Source of the cases (published or unpublished) was not included as a variable, as only 5/164 (3%) had no hypotonia in published subgroup, compared to 85/236 (36%) in unpublished subgroup. Multivariable model with stepwise selection was acceptable (Hosmer–Lemeshow p = 0.443) and had explained approximately 31% of variance (Nagelkerke pR^2 = 0.310). NA – not applicable.

Supplementary Table 33. **Univariate and multivariable models of muscle hypertonia (*n* = 303)**

**Parameter**	**Univariate model**		**Multivariable model** (unadjusted)		**Multivariable model** (stepwise selection)	
	**OR (95% CI)**	***P*-value**	**OR (95% CI)**	***P*-value**	**OR (95% CI)**	***P*-value**
Exon (Exon 11 (*n* = 62) as reference)						
Exon 3 (*n* = 25)	0.41 (0.144, 1.098)	0.086	1.08 (0.243, 4.658)	0.921	Excluded	NA
Exon 4 (*n* = 22)	0.74 (0.269, 1.963)	0.546	0.42 (0.095, 1.738)	0.234	Excluded	NA
Exon 5 (*n* = 45)	0.78 (0.357, 1.686)	0.528	1.19 (0.351, 4.092)	0.778	Excluded	NA
Exon 6 (*n* = 14)	1.42 (0.443, 4.779)	0.555	2.13 (0.428, 11.121)	0.358	Excluded	NA
Exon 7 (*n* = 30)	1.22 (0.509, 2.946)	0.657	1.25 (0.357, 4.478)	0.725	Excluded	NA
Exon 8 (*n* = 27)	1.15 (0.464, 2.863)	0.764	2.63 (0.675, 10.546)	0.166	Excluded	NA
Exon 9 (*n* = 22)	1.87 (0.697, 5.272)	0.222	1.3 (0.317, 5.476)	0.719	Excluded	NA
Exon 10 (*n* = 56)	1.65 (0.796, 3.456)	0.181	1.65 (0.51, 5.469)	0.407	Excluded	NA
Variant type (Missense (*n* = 193) as reference)						
Indels (*n* = 25)	0.28 (0.09, 0.724)	0.014	0.13 (0.028, 0.504)	0.006	0.16 (0.036, 0.536)	0.006
Frameshift or Nonsense (NMD-escape) (*n* = 30)	1.12 (0.516, 2.435)	0.771	1.08 (0.306, 3.842)	0.906	0.98 (0.394, 2.399)	0.968
Frameshift or Nonsense (NMD-predicted) (*n* = 39)	2.52 (1.232, 5.436)	0.014	1.12 (0.428, 2.985)	0.812	1.68 (0.739, 3.958)	0.221
Splice (*n* = 16)	4.86 (1.508, 21.673)	0.016	4.04 (0.922, 22.896)	0.082	4.43 (1.128, 22.683)	0.046
Source (Published (*n* = 74) as reference)						
Unpublished (*n* = 229)	0.08 (0.037, 0.168)	< 0.001	0.04 (0.017, 0.102)	< 0.001	0.05 (0.019, 0.11)	< 0.001
Sex (Females (*n* = 184) as reference)						
Males (*n* = 119)	0.33 (0.204, 0.536)	< 0.001	0.18 (0.08, 0.361)	< 0.001	0.2 (0.099, 0.373)	< 0.001
Presentation (Childhood (*n* = 47) as reference)						
Neonatal (*n* = 127)	1.87 (0.999, 3.567)	0.052	1.47 (0.575, 3.812)	0.426	Excluded	NA
Infantile (*n* = 119)	1.36 (0.72, 2.598)	0.347	1.09 (0.454, 2.627)	0.853	Excluded	NA

Analysis includes cases with available sex, age at presentation, muscle hypertonia status (present or absent). Cases with variants in Exons 1 were excluded (does not fulfil ≥ 10 cases per comparison group criteria). Multivariable model with stepwise selection was acceptable (Hosmer–Lemeshow p = 0.464) and had explained approximately 41% of variance (Nagelkerke pR^2 = 0.414). NA – not applicable.

Supplementary Table 34. **Univariate and multivariable models of microcephaly (*n* = 315)**

**Parameter**	**Univariate model**		**Multivariable model** (unadjusted)		**Multivariable model** (stepwise selection)	
	**OR (95% CI)**	***P*-value**	**OR (95% CI)**	***P*-value**	**OR (95% CI)**	***P*-value**
Exon (Exon 11 (*n* = 60) as reference)						
Exon 3 (*n* = 28)	0.25 (0.089, 0.665)	0.007	0.68 (0.173, 2.574)	0.573	Excluded	NA
Exon 4 (*n* = 24)	0.76 (0.293, 1.986)	0.580	0.82 (0.225, 2.991)	0.758	Excluded	NA
Exon 5 (*n* = 40)	0.51 (0.223, 1.141)	0.104	1.22 (0.392, 3.864)	0.733	Excluded	NA
Exon 6 (*n* = 14)	0.76 (0.234, 2.495)	0.652	1.67 (0.373, 7.754)	0.502	Excluded	NA
Exon 7 (*n* = 31)	1.06 (0.441, 2.577)	0.898	1.78 (0.545, 6.045)	0.343	Excluded	NA
Exon 8 (*n* = 26)	0.28 (0.097, 0.744)	0.014	0.95 (0.24, 3.656)	0.939	Excluded	NA
Exon 9 (*n* = 21)	1.24 (0.454, 3.554)	0.676	1.32 (0.327, 5.534)	0.702	Excluded	NA
Exon 10 (*n* = 71)	1.95 (0.947, 4.072)	0.072	1.82 (0.639, 5.361)	0.268	Excluded	NA
Variant type (Missense (*n* = 196) as reference)						
Indels (*n* = 27)	1.02 (0.448, 2.296)	0.956	0.97 (0.336, 2.772)	0.958	1.03 (0.385, 2.706)	0.954
Frameshift or Nonsense (NMD-escape) (*n* = 32)	3.27 (1.483, 7.792)	0.005	4.05 (1.198, 14.731)	0.028	3.29 (1.319, 8.894)	0.014
Frameshift or Nonsense (NMD-predicted) (*n* = 44)	3.84 (1.885, 8.358)	< 0.001	1.92 (0.792, 4.846)	0.156	2.12 (0.94, 5.017)	0.077
Splice (*n* = 16)	2.81 (0.984, 9.211)	0.064	1.87 (0.542, 7.179)	0.337	1.96 (0.61, 7.045)	0.274
Source (Published (*n* = 84) as reference)						
Unpublished (*n* = 231)	0.24 (0.135, 0.421)	< 0.001	0.17 (0.082, 0.331)	< 0.001	0.17 (0.087, 0.328)	< 0.001
Sex (Females (*n* = 203) as reference)						
Males (*n* = 112)	0.2 (0.118, 0.324)	< 0.001	0.27 (0.139, 0.517)	< 0.001	0.25 (0.137, 0.455)	< 0.001
Presentation (Childhood (*n* = 48) as reference)						
Neonatal (*n* = 146)	8.32 (3.882, 19.578)	< 0.001	3.99 (1.58, 10.867)	0.005	4.9 (2.018, 12.916)	0.001
Infantile (*n* = 121)	4.26 (1.972, 10.076)	< 0.001	3.3 (1.347, 8.729)	0.012	3.61 (1.508, 9.453)	0.006

Analysis includes cases with available sex, age at presentation, microcephaly status (present or absent). Cases with variants in Exons 1 were excluded (does not fulfil ≥ 10 cases per comparison group criteria). Multivariable model with stepwise selection was acceptable (Hosmer–Lemeshow p = 0.688) and had explained approximately 36% of variance (Nagelkerke $pR^2 = 0.362$). NA – not applicable.

Supplementary Table 35. **Univariate and multivariable models of seizures (*n* = 305)**

**Parameter**	**Univariate model**		**Multivariable model** (unadjusted)		**Multivariable model** (stepwise selection)	
	**OR (95% CI)**	***P*-value**	**OR (95% CI)**	***P*-value**	**OR (95% CI)**	***P*-value**
Exon (Exon 11 (*n* = 65) as reference)						
Exon 3 (*n* = 28)	0.36 (0.136, 0.891)	0.031	0.32 (0.087, 1.092)	0.074	Excluded	NA
Exon 4 (*n* = 18)	1.19 (0.414, 3.597)	0.750	0.62 (0.168, 2.317)	0.472	Excluded	NA
Exon 5 (*n* = 46)	0.9 (0.42, 1.932)	0.788	0.49 (0.167, 1.375)	0.179	Excluded	NA
Exon 6 (*n* = 10)	0.76 (0.193, 2.965)	0.682	0.64 (0.129, 3.183)	0.580	Excluded	NA
Exon 7 (*n* = 37)	1.24 (0.547, 2.88)	0.605	0.54 (0.176, 1.604)	0.269	Excluded	NA
Exon 8 (*n* = 25)	0.59 (0.231, 1.501)	0.273	0.7 (0.206, 2.331)	0.565	Excluded	NA
Exon 9 (*n* = 21)	0.83 (0.308, 2.261)	0.716	0.37 (0.098, 1.363)	0.136	Excluded	NA
Exon 10 (*n* = 55)	1.14 (0.548, 2.366)	0.733	0.68 (0.237, 1.913)	0.470	Excluded	NA
Variant type (Missense (*n* = 195) as reference)						
Indels (*n* = 27)	0.5 (0.213, 1.139)	0.106	0.29 (0.1, 0.794)	0.018	0.38 (0.146, 0.948)	0.042
Frameshift or Nonsense (NMD-escape) (*n* = 30)	0.75 (0.343, 1.623)	0.464	0.33 (0.103, 0.997)	0.052	0.56 (0.24, 1.302)	0.180
Frameshift or Nonsense (NMD-predicted) (*n* = 38)	1.18 (0.586, 2.413)	0.647	0.65 (0.265, 1.586)	0.341	0.64 (0.287, 1.447)	0.283
Splice (*n* = 15)	12 (2.343, 219.578)	0.017	10.43 (1.876, 196.413)	0.029	8.76 (1.644, 162.49)	0.040
Source (Published (*n* = 62) as reference)						
Unpublished (*n* = 243)	0.27 (0.137, 0.505)	< 0.001	0.27 (0.127, 0.544)	< 0.001	0.26 (0.128, 0.516)	< 0.001
Sex (Females (*n* = 183) as reference)						
Males (*n* = 122)	0.58 (0.362, 0.913)	0.019	0.52 (0.279, 0.944)	0.033	0.52 (0.296, 0.907)	0.022
Presentation (Childhood (*n* = 46) as reference)						
Neonatal (*n* = 138)	6.34 (2.994, 14.491)	< 0.001	4.62 (1.911, 12.01)	0.001	5.03 (2.217, 12.216)	< 0.001
Infantile (*n* = 121)	4.47 (2.098, 10.257)	< 0.001	3.22 (1.386, 7.981)	0.008	3.32 (1.501, 7.876)	0.004

Analysis includes cases with available sex, age at presentation, seizures status (present or absent). Cases with variants in Exons 1 were excluded (does not fulfil ≥ 10 cases per comparison group criteria). Multivariable model with stepwise selection was acceptable (Hosmer–Lemeshow p = 0.807) and had explained approximately 23% of variance (Nagelkerke pR^2 = 0.233). NA – not applicable.

Supplementary Table 36. **Univariate and multivariable models of feeding difficulties (*n* = 287)**

**Parameter**	**Univariate model**		**Multivariable model** (unadjusted)		**Multivariable model** (stepwise selection)	
	**OR (95% CI)**	***P*-value**	**OR (95% CI)**	***P*-value**	**OR (95% CI)**	***P*-value**
Exon (Exon 11 (*n* = 57) as reference)						
Exon 3 (*n* = 24)	0.16 (0.041, 0.475)	0.002	0.45 (0.085, 2.037)	0.314	0.21 (0.046, 0.786)	0.029
Exon 4 (*n* = 19)	1.69 (0.581, 5.399)	0.348	4.84 (1.207, 21.342)	0.030	2.29 (0.723, 7.876)	0.169
Exon 5 (*n* = 42)	1.27 (0.565, 2.893)	0.565	3.86 (1.204, 13.091)	0.026	1.67 (0.689, 4.134)	0.258
Exon 6 (*n* = 14)	0.43 (0.12, 1.419)	0.177	0.77 (0.146, 3.729)	0.745	0.37 (0.082, 1.455)	0.169
Exon 7 (*n* = 32)	1 (0.42, 2.426)	0.992	1.94 (0.57, 6.867)	0.295	0.91 (0.337, 2.446)	0.849
Exon 8 (*n* = 22)	0.36 (0.123, 1.002)	0.057	1.53 (0.383, 6.091)	0.542	0.68 (0.212, 2.055)	0.501
Exon 9 (*n* = 19)	1.69 (0.581, 5.399)	0.348	4.31 (1.009, 19.963)	0.053	2.01 (0.605, 7.178)	0.265
Exon 10 (*n* = 58)	1.11 (0.528, 2.326)	0.788	1.99 (0.647, 6.469)	0.237	0.97 (0.418, 2.244)	0.943
Variant type (Missense (*n* = 185) as reference)						
Indels (*n* = 19)	0.75 (0.279, 1.94)	0.557	1.02 (0.287, 3.48)	0.975	Excluded	NA
Frameshift or Nonsense (NMD-escape) (*n* = 29)	1.96 (0.882, 4.608)	0.106	4.08 (1.185, 15.033)	0.029	Excluded	NA
Frameshift or Nonsense (NMD-predicted) (*n* = 38)	1.58 (0.784, 3.283)	0.205	0.97 (0.378, 2.498)	0.951	Excluded	NA
Splice (*n* = 16)	2.27 (0.793, 7.448)	0.142	0.83 (0.216, 3.371)	0.781	Excluded	NA
Source (Published (*n* = 64) as reference)						
Unpublished (*n* = 223)	0.13 (0.056, 0.256)	< 0.001	0.08 (0.031, 0.189)	< 0.001	0.1 (0.042, 0.225)	< 0.001
Sex (Females (*n* = 168) as reference)						
Males (*n* = 119)	0.67 (0.415, 1.068)	0.092	0.88 (0.439, 1.777)	0.725	Excluded	NA
Presentation (Childhood (*n* = 48) as reference)						
Neonatal (*n* = 135)	6.73 (3.23, 14.998)	< 0.001	4.5 (1.823, 11.866)	0.002	4.79 (2.039, 12.065)	0.001
Infantile (*n* = 104)	3.24 (1.53, 7.29)	0.003	2.7 (1.125, 6.914)	0.031	2.91 (1.23, 7.366)	0.018

Analysis includes cases with available sex, age at presentation, feeding difficulties status (present or absent). Cases with variants in Exons 1 were excluded (does not fulfil ≥ 10 cases per comparison group criteria). Multivariable model with stepwise selection was acceptable (Hosmer–Lemeshow p = 0.748) and had explained approximately 33% of variance (Nagelkerke $pR^2 = 0.328$). NA – not applicable.

Supplementary Table 37. **Univariate and multivariable models of dysmorphic features (*n* = 281)**

**Parameter**	**Univariate model**		**Multivariable model** (unadjusted)		**Multivariable model** (stepwise selection)	
	**OR (95% CI)**	***P*-value**	**OR (95% CI)**	***P*-value**	**OR (95% CI)**	***P*-value**
Exon (Exon 11 (*n* = 59) as reference)						
Exon 3 (*n* = 23)	0.18 (0.039, 0.589)	0.010	0.76 (0.126, 3.809)	0.741	Excluded	NA
Exon 4 (*n* = 20)	0.79 (0.273, 2.195)	0.654	1.18 (0.283, 4.897)	0.822	Excluded	NA
Exon 5 (*n* = 42)	0.28 (0.105, 0.68)	0.007	0.49 (0.136, 1.761)	0.278	Excluded	NA
Exon 6 (*n* = 12)	0.11 (0.006, 0.608)	0.038	0.14 (0.006, 1.134)	0.107	Excluded	NA
Exon 7 (*n* = 28)	0.66 (0.254, 1.644)	0.377	0.85 (0.233, 3.121)	0.809	Excluded	NA
Exon 8 (*n* = 23)	0.11 (0.017, 0.433)	0.005	0.29 (0.036, 1.626)	0.189	Excluded	NA
Exon 9 (*n* = 15)	0.59 (0.167, 1.885)	0.389	0.87 (0.167, 4.296)	0.860	Excluded	NA
Exon 10 (*n* = 59)	1.15 (0.556, 2.369)	0.712	0.96 (0.3, 3.053)	0.938	Excluded	NA
Variant type (Missense (*n* = 173) as reference)						
Indels (*n* = 23)	1.43 (0.545, 3.519)	0.447	1.23 (0.358, 3.97)	0.736	Excluded	NA
Frameshift or Nonsense (NMD-escape) (*n* = 32)	2.37 (1.086, 5.127)	0.029	1.28 (0.355, 4.635)	0.708	Excluded	NA
Frameshift or Nonsense (NMD-predicted) (*n* = 40)	2.68 (1.323, 5.453)	0.006	1.3 (0.506, 3.32)	0.586	Excluded	NA
Splice (*n* = 13)	0.8 (0.175, 2.763)	0.749	0.38 (0.063, 1.722)	0.238	Excluded	NA
Source (Published (*n* = 53) as reference)						
Unpublished (*n* = 228)	0.18 (0.091, 0.329)	< 0.001	0.12 (0.049, 0.266)	< 0.001	0.12 (0.053, 0.26)	< 0.001
Sex (Females (*n* = 168) as reference)						
Males (*n* = 113)	0.42 (0.24, 0.71)	0.002	0.54 (0.237, 1.186)	0.130	0.41 (0.198, 0.799)	0.011
Presentation (Childhood (*n* = 44) as reference)						
Neonatal (*n* = 99)	21 (6.131, 131.969)	< 0.001	16.77 (4.074, 119.253)	0.001	17.05 (4.547, 112.72)	< 0.001
Infantile (*n* = 138)	6 (1.656, 38.598)	0.019	6.51 (1.566, 46.016)	0.023	5.79 (1.485, 38.897)	0.027

Analysis includes cases with available sex, age at presentation, dysmorphic features status (present or absent). Cases with variants in Exons 1 were excluded (does not fulfil ≥ 10 cases per comparison group criteria). Multivariable model with stepwise selection was acceptable (Hosmer–Lemeshow p = 0.415) and had explained approximately 34% of variance (Nagelkerke $pR^2 = 0.339$). NA – not applicable.

Supplementary Table 38. **Univariate and multivariable models of abnormal movements (*n* = 323)**

**Parameter**	**Univariate model**		**Multivariable model** (unadjusted)		**Multivariable model** (stepwise selection)	
	**OR (95% CI)**	***P*-value**	**OR (95% CI)**	***P*-value**	**OR (95% CI)**	***P*-value**
Exon (Exon 11 (*n* = 64) as reference)						
Exon 3 (*n* = 34)	1.17 (0.491, 2.896)	0.723	0.49 (0.154, 1.519)	0.221	Excluded	NA
Exon 4 (*n* = 25)	0.84 (0.327, 2.217)	0.722	0.73 (0.221, 2.368)	0.098	Excluded	NA
Exon 5 (*n* = 49)	0.63 (0.295, 1.352)	0.239	0.42 (0.148, 1.149)	0.098	Excluded	NA
Exon 6 (*n* = 13)	0.9 (0.267, 3.267)	0.863	0.55 (0.13, 2.408)	0.418	Excluded	NA
Exon 7 (*n* = 33)	1.5 (0.608, 3.893)	0.391	1.07 (0.336, 3.39)	0.910	Excluded	NA
Exon 8 (*n* = 38)	1.81 (0.748, 4.644)	0.200	0.82 (0.252, 2.631)	0.735	Excluded	NA
Exon 9 (*n* = 18)	0.7 (0.242, 2.071)	0.512	0.71 (0.186, 2.712)	0.612	Excluded	NA
Exon 10 (*n* = 49)	0.81 (0.377, 1.753)	0.597	1.03 (0.364, 2.874)	0.952	Excluded	NA
Variant type (Missense (*n* = 215) as reference)						
Indels (*n* = 23)	0.64 (0.269, 1.569)	0.317	0.48 (0.172, 1.34)	0.159	Excluded	NA
Frameshift or Nonsense (NMD-escape) (*n* = 34)	0.7 (0.338, 1.501)	0.353	0.62 (0.205, 1.812)	0.384	Excluded	NA
Frameshift or Nonsense (NMD-predicted) (*n* = 31)	0.46 (0.214, 0.991)	0.046	0.65 (0.254, 1.648)	0.362	Excluded	NA
Splice (*n* = 20)	0.92 (0.359, 2.529)	0.858	1.31 (0.468, 3.903)	0.618	Excluded	NA
Sex (Females (*n* = 169) as reference)						
Males (*n* = 154)	2.18 (1.372, 3.494)	0.001	1.16 (0.619, 2.167)	0.644	1.12 (0.637, 1.954)	0.696
Source (Published (*n* = 99) as reference)						
Unpublished (*n* = 224)	0.03 (0.007, 0.082)	< 0.001	0.03 (0.008, 0.093)	< 0.001	0.03 (0.008, 0.094)	< 0.001
Presentation (Childhood (*n* = 75) as reference)						
Neonatal (*n* = 113)	0.32 (0.163, 0.607)	0.001	0.57 (0.249, 1.299)	0.187	0.55 (0.255, 1.17)	0.124
Infantile (*n* = 135)	0.55 (0.282, 1.033)	0.069	0.62 (0.283, 1.329)	0.224	0.62 (0.292, 1.283)	0.201

Analysis includes cases with available sex, age at presentation, abnormal movements status (present or absent). Cases with variants in Exons 1 were excluded (does not fulfil ≥ 10 cases per comparison group criteria). Source of the cases (published or unpublished) was not included as a variable, as only 3/99 (3%) had no hypotonia in published subgroup, compared to 85/224 (51%) in unpublished subgroup. Multivariable model with stepwise selection was acceptable (Hosmer–Lemeshow p = 0.182) and had explained approximately 33% of variance (Nagelkerke pR^2 = 0.333). NA – not applicable.

Supplementary Table 39. **Univariate and multivariable models of peripheral neuropathy (*n* = 281)**

**Parameter**	**Univariate model**		**Multivariable model** (unadjusted)		**Multivariable model** (stepwise selection)	
	**OR (95% CI)**	***P*-value**	**OR (95% CI)**	***P*-value**	**OR (95% CI)**	***P*-value**
Exon (Exon 11 (*n* = 32) as reference)						
Exon 3 (*n* = 30)	3.34 (1.188, 9.958)	0.025	Multicollinearity	NA	Excluded	NA
Exon 4 (*n* = 14)	1.42 (0.356, 5.382)	0.608	Multicollinearity	NA	Excluded	NA
Exon 5 (*n* = 28)	0.56 (0.151, 1.865)	0.352	Multicollinearity	NA	Excluded	NA
Exon 6 (*n* = 10)	1.7 (0.364, 7.501)	0.481	Multicollinearity	NA	Excluded	NA
Exon 7 (*n* = 26)	1.35 (0.44, 4.188)	0.596	Multicollinearity	NA	Excluded	NA
Exon 8 (*n* = 26)	6.94 (2.27, 23.513)	0.001	Multicollinearity	NA	Excluded	NA
Exon 9 (*n* = 11)	0.57 (0.077, 2.779)	0.518	Multicollinearity	NA	Excluded	NA
Exon 10 (*n* = 32)	0.59 (0.174, 1.887)	0.379	Multicollinearity	NA	Excluded	NA
Variant type (Missense (*n* = 156) as reference)						
Indels (*n* = 10)	0.6 (0.126, 2.246)	0.471	0.99 (0.188, 4.249)	0.994	Excluded	NA
Frameshift or Nonsense (NMD-escape) (*n* = 13)	0.88 (0.255, 2.744)	0.822	3.3 (0.809, 13.041)	0.087	Excluded	NA
Frameshift or Nonsense (NMD-predicted) (*n* = 19)	0.08 (0.004, 0.391)	0.014	0.51 (0.026, 3.137)	0.540	Excluded	NA
Splice (*n* = 11)	0.31 (0.046, 1.257)	0.144	0.2 (0.023, 1.166)	0.092	Excluded	NA
Source (Published (*n* = 52) as reference)						
Unpublished (*n* = 157)	0.09 (0.041, 0.183)	< 0.001	0.09 (0.035, 0.214)	< 0.001	0.12 (0.051, 0.26)	< 0.001
Sex (Females (*n* = 96) as reference)						
Males (*n* = 113)	5.66 (3.008, 11.139)	< 0.001	2.61 (1.198, 5.85)	0.017	2.67 (1.261, 5.777)	0.011
Presentation (Childhood (*n* = 54) as reference)						
Neonatal (*n* = 63)	0.1 (0.038, 0.241)	< 0.001	0.16 (0.053, 0.465)	0.001	0.15 (0.051, 0.416)	< 0.001
Infantile (*n* = 92)	0.44 (0.22, 0.872)	0.020	0.38 (0.162, 0.841)	0.019	0.39 (0.174, 0.854)	0.020

Analysis includes cases with available sex, age at presentation, peripheral neuropathy status (present or absent). Cases with variants in Exons 1 were excluded (does not fulfil ≥ 10 cases per comparison group criteria). Exon variable was excluded do to multicollinearity with variant variable (variant GVIF 6.567, exons GVIF 8.983). Multivariable model with stepwise selection was acceptable (Hosmer–Lemeshow p = 0.923) and had explained approximately 42% of variance (Nagelkerke $pR^2 = 0.423$). NA – not applicable.

Supplementary Table 40. **Univariate and multivariable models of visual impairment (*n* = 243)**

**Parameter**	**Univariate model**		**Multivariable model** (unadjusted)		**Multivariable model** (stepwise selection)	
	**OR (95% CI)**	***P*-value**	**OR (95% CI)**	***P*-value**	**OR (95% CI)**	***P*-value**
Exon (Exon 11 (*n* = 48) as reference)						
Exon 3 (*n* = 25)	0.21 (0.054, 0.642)	0.011	0.33 (0.073, 1.319)	0.131	Excluded	NA
Exon 4 (*n* = 14)	1.09 (0.325, 3.64)	0.891	1.04 (0.248, 4.374)	0.959	Excluded	NA
Exon 5 (*n* = 39)	0.48 (0.195, 1.156)	0.107	0.69 (0.215, 2.228)	0.537	Excluded	NA
Exon 6 (*n* = 11)	0.11 (0.006, 0.634)	0.041	0.11 (0.005, 0.836)	0.065	Excluded	NA
Exon 7 (*n* = 26)	1.27 (0.487, 3.34)	0.627	1.23 (0.366, 4.167)	0.741	Excluded	NA
Exon 8 (*n* = 19)	0.2 (0.043, 0.71)	0.022	0.32 (0.056, 1.465)	0.161	Excluded	NA
Exon 9 (*n* = 16)	1.09 (0.346, 3.419)	0.885	0.68 (0.159, 2.85)	0.594	Excluded	NA
Exon 10 (*n* = 45)	0.95 (0.42, 2.152)	0.904	0.54 (0.168, 1.683)	0.288	Excluded	NA
Variant type (Missense (*n* = 162) as reference)						
Indels (*n* = 16)	0.67 (0.18, 2.017)	0.500	0.74 (0.175, 2.601)	0.655	Excluded	NA
Frameshift or Nonsense (NMD-escape) (*n* = 25)	2.17 (0.923, 5.132)	0.075	1.21 (0.354, 4.15)	0.759	Excluded	NA
Frameshift or Nonsense (NMD-predicted) (*n* = 27)	2.91 (1.275, 6.864)	0.012	2.08 (0.758, 5.866)	0.159	Excluded	NA
Splice (*n* = 13)	1.71 (0.529, 5.406)	0.353	1.24 (0.326, 4.514)	0.747	Excluded	NA
Source (Published (*n* = 39) as reference)						
Unpublished (*n* = 204)	0.36 (0.178, 0.727)	0.005	0.27 (0.112, 0.615)	0.002	0.24 (0.108, 0.525)	< 0.001
Sex (Females (*n* = 143) as reference)						
Males (*n* = 100)	0.4 (0.226, 0.688)	0.001	0.52 (0.249, 1.074)	0.080	0.44 (0.226, 0.838)	0.014
Presentation (Childhood (*n* = 45) as reference)						
Neonatal (*n* = 102)	3.62 (1.694, 8.209)	0.001	1.9 (0.756, 4.951)	0.177	2.63 (1.131, 6.448)	0.028
Infantile (*n* = 96)	1.21 (0.546, 2.808)	0.646	0.79 (0.319, 2.018)	0.623	0.95 (0.408, 2.289)	0.903

Analysis includes cases with available sex, age at presentation, visual impairment status (present or absent). Cases with variants in Exons 1 were excluded (does not fulfil ≥ 10 cases per comparison group criteria). Multivariable model with stepwise selection was acceptable (Hosmer–Lemeshow p = 0.513) and had explained approximately 18% of variance (Nagelkerke $pR^2 = 0.183$). NA – not applicable.

Supplementary table 41. **Univariate and multivariable models of hearing impairment (*n* = 219)**

**Parameter**	**Univariate model**		**Multivariable model** (unadjusted)		**Multivariable model** (stepwise selection)	
	**OR (95% CI)**	***P*-value**	**OR (95% CI)**	***P*-value**	**OR (95% CI)**	***P*-value**
Exon (Exon 11 (*n* = 43) as reference)						
Exon 3 (*n* = 22)	0.12 (0.007, 0.697)	0.052	0.24 (0.011, 1.926)	0.232	Excluded	NA
Exon 4 (*n* = 14)	0.7 (0.141, 2.747)	0.634	0.64 (0.097, 3.796)	0.623	Excluded	NA
Exon 5 (*n* = 40)	0.86 (0.319, 2.291)	0.764	0.82 (0.208, 3.465)	0.777	Excluded	NA
Exon 6 (Excluded)						
Exon 7 (*n* = 24)	0.86 (0.262, 2.632)	0.797	0.81 (0.18, 3.776)	0.785	Excluded	NA
Exon 8 (*n* = 19)	0.14 (0.008, 0.822)	0.073	0.23 (0.011, 1.852)	0.218	Excluded	NA
Exon 9 (*n* = 15)	1.72 (0.487, 5.886)	0.386	1.35 (0.26, 7.253)	0.723	Excluded	NA
Exon 10 (*n* = 42)	1.94 (0.791, 4.877)	0.152	1.67 (0.44, 6.836)	0.458	Excluded	NA
Variant type (Missense (*n* = 139) as reference)						
Indels (*n* = 16)	0.52 (0.079, 1.996)	0.403	0.37 (0.051, 1.73)	0.255	Excluded	NA
Frameshift or Nonsense (NMD-escape) (*n* = 25)	1.71 (0.644, 4.251)	0.260	1.01 (0.239, 4.468)	0.990	Excluded	NA
Frameshift or Nonsense (NMD-predicted) (*n* = 28)	2.72 (1.148, 6.375)	0.021	0.85 (0.287, 2.446)	0.763	Excluded	NA
Splice (*n* = 11)	3.03 (0.823, 10.735)	0.083	1.65 (0.368, 7.136)	0.503	Excluded	NA
Source (Published (*n* = 23) as reference)						
Unpublished (*n* = 196)	1 (0.39, 2.886)	0.995	0.9 (0.28, 3.166)	0.857	Excluded	NA
Sex (Females (*n* = 132) as reference)						
Males (*n* = 87)	0.4 (0.196, 0.766)	0.008	0.85 (0.347, 2.052)	0.716	Excluded	NA
Presentation (Childhood (*n* = 43) as reference)						
Neonatal (*n* = 93)	10 (3.334, 43.291)	< 0.001	5.47 (1.633, 25.295)	0.012	10.06 (3.341, 43.668)	< 0.001
Infantile (*n* = 83)	2.65 (0.808, 11.964)	0.143	1.96 (0.553, 9.238)	0.335	2.71 (0.822, 12.248)	0.135

Analysis includes cases with available sex, age at presentation, hearing impairment status (present or absent). Cases with variants in exon 1 were excluded (does not fulfil ≥ 10 cases per comparison group criteria). Cases with variants in exon 6 had no hearing impairment and were excluded from subanlysis. Multivariable model with stepwise selection had poor fit (Hosmer–Lemeshow $p < 0.001$) and had explained approximately 17% of variance (Nagelkerke $pR^2 = 0.169$). NA – not applicable.

Supplementary Table 42. **Univariate and multivariable models of abnormal skeletal morphology (*n* = 258)**

**Parameter**	**Univariate model**		**Multivariable model** (unadjusted)		**Multivariable model** (stepwise selection)	
	**OR (95% CI)**	***P*-value**	**OR (95% CI)**	***P*-value**	**OR (95% CI)**	***P*-value**
Exon (Exon 11 (*n* = 50) as reference)						
Exon 3 (*n* = 25)	0.83 (0.275, 2.327)	0.723	2.29 (0.522, 10.475)	0.274	Excluded	NA
Exon 4 (*n* = 18)	1.06 (0.321, 3.278)	0.917	1.52 (0.322, 7.249)	0.592	Excluded	NA
Exon 5 (*n* = 39)	0.31 (0.094, 0.898)	0.040	0.79 (0.18, 3.394)	0.749	Excluded	NA
Exon 6 (*n* = 11)	0.47 (0.067, 2.105)	0.371	0.88 (0.092, 6.042)	0.903	Excluded	NA
Exon 7 (*n* = 30)	1.06 (0.397, 2.772)	0.902	1.33 (0.352, 5.274)	0.679	Excluded	NA
Exon 8 (*n* = 20)	0.71 (0.202, 2.198)	0.565	2.4 (0.489, 11.749)	0.274	Excluded	NA
Exon 9 (*n* = 16)	0.71 (0.176, 2.406)	0.597	0.86 (0.147, 4.633)	0.864	Excluded	NA
Exon 10 (*n* = 49)	1.59 (0.704, 3.661)	0.266	1.73 (0.525, 6.159)	0.379	Excluded	NA
Variant type (Missense (*n* = 165) as reference)						
Indels (*n* = 19)	1.82 (0.641, 4.856)	0.238	2.99 (0.909, 9.575)	0.065	2.98 (0.99, 8.59)	0.045
Frameshift or Nonsense (NMD-escape) (*n* = 28)	2.02 (0.855, 4.637)	0.100	2.01 (0.53, 8.074)	0.311	1.63 (0.625, 4.103)	0.302
Frameshift or Nonsense (NMD-predicted) (*n* = 34)	2.78 (1.289, 5.971)	0.009	1.83 (0.701, 4.844)	0.217	2.24 (0.965, 5.19)	0.059
Splice (*n* = 12)	0.62 (0.094, 2.498)	0.555	0.39 (0.047, 2.046)	0.310	0.41 (0.055, 1.876)	0.302
Source (Published (*n* = 34) as reference)						
Unpublished (*n* = 224)	0.2 (0.092, 0.424)	< 0.001	0.12 (0.044, 0.283)	< 0.001	0.11 (0.044, 0.261)	< 0.001
Sex (Females (*n* = 157) as reference)						
Males (*n* = 101)	0.37 (0.198, 0.666)	0.001	0.29 (0.124, 0.647)	0.003	0.3 (0.142, 0.613)	0.001
Presentation (Childhood (*n* = 44) as reference)						
Neonatal (*n* = 114)	2.02 (0.912, 4.861)	0.096	1.68 (0.589, 5.149)	0.346	Excluded	NA
Infantile (*n* = 100)	1.51 (0.663, 3.709)	0.342	1.65 (0.613, 4.778)	0.335	Excluded	NA

Analysis includes cases with available sex, age at presentation, abnormal skeletal morphology status (present or absent). Cases with variants in exon 1 were excluded (does not fulfil ≥ 10 cases per comparison group criteria). Multivariable model with stepwise selection was acceptable (Hosmer–Lemeshow p = 0.503) and had explained approximately 22% of variance (Nagelkerke $pR^2 = 0.220$). NA – not applicable.

Supplementary Table 43. **Univariate and multivariable models of strabismus (*n* = 245)**

**Parameter**	**Univariate model**		**Multivariable model** (unadjusted)		**Multivariable model** (stepwise selection)	
	**OR (95% CI)**	***P*-value**	**OR (95% CI)**	***P*-value**	**OR (95% CI)**	***P*-value**
Exon (Exon 11 (*n* = 48) as reference)						
Exon 3 (*n* = 21)	0.23 (0.05, 0.807)	0.035	0.45 (0.081, 2.098)	0.330	Excluded	NA
Exon 4 (*n* = 16)	0.47 (0.117, 1.564)	0.239	0.59 (0.124, 2.584)	0.494	Excluded	NA
Exon 5 (*n* = 41)	0.99 (0.423, 2.314)	0.985	1.22 (0.388, 4.015)	0.737	Excluded	NA
Exon 6 (*n* = 10)	0.35 (0.049, 1.581)	0.213	0.47 (0.059, 2.683)	0.425	Excluded	NA
Exon 7 (*n* = 28)	0.78 (0.29, 2.019)	0.609	1 (0.296, 3.455)	0.997	Excluded	NA
Exon 8 (*n* = 21)	0.56 (0.174, 1.641)	0.305	1.03 (0.254, 4.124)	0.962	Excluded	NA
Exon 9 (*n* = 16)	0.64 (0.177, 2.048)	0.461	0.86 (0.185, 3.821)	0.843	Excluded	NA
Exon 10 (*n* = 44)	0.8 (0.342, 1.852)	0.603	1.16 (0.359, 3.909)	0.809	Excluded	NA
Variant type (Missense (*n* = 158) as reference)						
Indels (*n* = 17)	0.29 (0.044, 1.073)	0.107	0.26 (0.038, 1.048)	0.092	0.29 (0.045, 1.114)	0.116
Frameshift or Nonsense (NMD-escape) (*n* = 26)	2.52 (1.087, 5.931)	0.031	1.95 (0.584, 6.85)	0.284	2.3 (0.978, 5.48)	0.056
Frameshift or Nonsense (NMD-predicted) (*n* = 31)	1.03 (0.435, 2.3)	0.947	0.67 (0.242, 1.764)	0.421	0.81 (0.329, 1.916)	0.644
Splice (*n* = 13)	2.52 (0.798, 8.2)	0.112	1.82 (0.521, 6.504)	0.347	2.18 (0.675, 7.205)	0.189
Source (Published (*n* = 26) as reference)						
Unpublished (*n* = 219)	0.96 (0.418, 2.36)	0.933	0.97 (0.366, 2.673)	0.946	Excluded	NA
Sex (Females (*n* = 143) as reference)						
Males (*n* = 102)	0.61 (0.346, 1.043)	0.074	0.77 (0.385, 1.521)	0.448	Excluded	NA
Presentation (Childhood (*n* = 45) as reference)						
Neonatal (*n* = 106)	3.04 (1.34, 7.593)	0.011	2.22 (0.868, 6.144)	0.107	2.84 (1.202, 7.348)	0.022
Infantile (*n* = 94)	2.5 (1.084, 6.343)	0.040	1.96 (0.801, 5.213)	0.153	2.34 (1, 6.005)	0.060

Analysis includes cases with available sex, age at presentation, strabismus status (present or absent). Cases with variants in exon 1 were excluded (does not fulfil ≥ 10 cases per comparison group criteria). Multivariable model with stepwise selection was acceptable (Hosmer–Lemeshow p = 0.948) and had explained approximately 9% of variance (Nagelkerke $pR^2 = 0.094$). NA – not applicable.

Supplementary Table 44. **Univariate and multivariable models of nystagmus (*n* = 250)**

**Parameter**	**Univariate model**		**Multivariable model** (unadjusted)		**Multivariable model** (stepwise selection)	
	**OR (95% CI)**	***P*-value**	**OR (95% CI)**	***P*-value**	**OR (95% CI)**	***P*-value**
Exon (Exon 11 (*n* = 49) as reference)						
Exon 3 (*n* = 22)	0.15 (0.008, 0.827)	0.075	0.13 (0.006, 0.889)	0.075	Excluded	NA
Exon 4 (*n* = 16)	0.44 (0.063, 1.892)	0.321	0.49 (0.063, 2.662)	0.441	Excluded	NA
Exon 5 (*n* = 41)	0.33 (0.087, 1.056)	0.078	0.34 (0.073, 1.401)	0.144	Excluded	NA
Exon 6 (*n* = 11)	0.31 (0.016, 1.872)	0.285	0.31 (0.015, 2.262)	0.314	Excluded	NA
Exon 7 (*n* = 27)	1.54 (0.54, 4.332)	0.411	1.39 (0.399, 4.984)	0.605	Excluded	NA
Exon 8 (*n* = 21)	1.23 (0.372, 3.822)	0.721	1.24 (0.299, 5.099)	0.765	Excluded	NA
Exon 9 (*n* = 15)	0.47 (0.068, 2.057)	0.368	0.76 (0.093, 4.418)	0.772	Excluded	NA
Exon 10 (*n* = 48)	0.71 (0.262, 1.876)	0.494	0.82 (0.236, 2.843)	0.751	Excluded	NA
Variant type (Missense (*n* = 163) as reference)						
Indels (*n* = 19)	1.46 (0.446, 4.14)	0.495	1.44 (0.373, 5.016)	0.579	Excluded	NA
Frameshift or Nonsense (NMD-escape) (*n* = 25)	0.78 (0.216, 2.225)	0.668	0.67 (0.144, 2.866)	0.599	Excluded	NA
Frameshift or Nonsense (NMD-predicted) (*n* = 30)	0.45 (0.104, 1.394)	0.218	0.46 (0.095, 1.718)	0.283	Excluded	NA
Splice (*n* = 13)	0.74 (0.112, 2.952)	0.710	0.81 (0.103, 4.182)	0.819	Excluded	NA
Source (Published (*n* = 31) as reference)						
Unpublished (*n* = 219)	0.29 (0.13, 0.667)	0.003	0.4 (0.158, 1.037)	0.056	0.29 (0.13, 0.667)	0.003
Sex (Females (*n* = 144) as reference)						
Males (*n* = 106)	1.62 (0.853, 3.103)	0.14	1.52 (0.666, 3.538)	0.322	Excluded	NA
Presentation (Childhood (*n* = 45) as reference)						
Neonatal (*n* = 106)	1.26 (0.511, 3.444)	0.628	1.62 (0.553, 5.236)	0.393	Excluded	NA
Infantile (*n* = 99)	1.29 (0.517, 3.538)	0.600	1.31 (0.476, 3.901)	0.612	Excluded	NA

Analysis includes cases with available sex, age at presentation, nystagmus status (present or absent). Cases with variants in exon 1 were excluded (does not fulfil ≥ 10 cases per comparison group criteria). Model with stepwise selection had explained approximately 5% of variance (Nagelkerke pR^2 = 0.053). NA – not applicable.

Supplementary Table 45. **Univariate and multivariable models of ophthalmoplegia (*n* = 207)**

**Parameter**	**Univariate model**		**Multivariable model** (unadjusted)		**Multivariable model** (stepwise selection)	
	**OR (95% CI)**	***P*-value**	**OR (95% CI)**	***P*-value**	**OR (95% CI)**	***P*-value**
Exon (Exon 11 (*n* = 33) as reference)						
Exon 3 (*n* = 23)	2.25 (0.387, 13.101)	0.345	3.04 (0.345, 29.903)	0.318	Excluded	NA
Exon 4 (excluded)						
Exon 5 (*n* = 41)	4.22 (1.156, 20.146)	0.041	13.37 (2.136, 116.347)	0.010	Excluded	NA
Exon 6 (*n* = 10)	1.67 (0.078, 14.82)	0.673	4.96 (0.184, 73.593)	0.258	Excluded	NA
Exon 7 (*n* = 27)	1.87 (0.325, 10.821)	0.462	1.02 (0.134, 7.775)	0.984	Excluded	NA
Exon 8 (*n* = 21)	6 (1.406, 31.322)	0.020	11.06 (1.551, 104.144)	0.023	Excluded	NA
Exon 9 (*n* = 16)	2.14 (0.263, 14.225)	0.429	5.25 (0.407, 68.637)	0.193	Excluded	NA
Exon 10 (*n* = 25)	1.87 (0.432, 9.609)	0.409	5.76 (0.762, 56.265)	0.106	Excluded	NA
Variant type (Missense (*n* = 148) as reference)						
Indels (*n* = 19)	0.55 (0.084, 2.089)	0.445	0.62 (0.068, 3.435)	0.614	Excluded	NA
Frameshift or Nonsense (NMD-escape) (excluded)						
Frameshift or Nonsense (NMD-predicted) (*n* = 28)	0.56 (0.127, 1.764)	0.375	0.98 (0.175, 4.689)	0.977	Excluded	NA
Splice (*n* = 12)	0.43 (0.023, 2.341)	0.424	0.1 (0.004, 0.831)	0.068	Excluded	NA
Source (Published (*n* = 30) as reference)						
Unpublished (*n* = 177)	0.16 (0.066, 0.375)	< 0.001	0.05 (0.01, 0.183)	< 0.001	0.17 (0.069, 0.42)	< 0.001
Sex (Females (*n* = 110) as reference)						
Males (*n* = 97)	1.82 (0.852, 3.987)	0.126	0.91 (0.315, 2.643)	0.868	Excluded	NA
Presentation (Childhood (*n* = 40) as reference)						
Neonatal (*n* = 82)	1.4 (0.395, 6.555)	0.625	3.49 (0.637, 27.121)	0.183	1.95 (0.517, 9.658)	0.356
Infantile (*n* = 85)	3.79 (1.199, 16.87)	0.041	5.79 (1.305, 38.121)	0.037	3.72 (1.112, 17.191)	0.053

Analysis includes cases with available sex, age at presentation, ophthalmoplegia status (present or absent). Cases with variants in exon 1 were excluded (does not fulfil ≥ 10 cases per comparison group criteria). Cases with variants in exon 4 or frameshift or nonsense in NMD-escape region had no ophthalmoplegia and were excluded from sub-analysis. Multivariable model with stepwise selection was acceptable (Hosmer–Lemeshow p = 0.781) and had explained approximately 17% of variance (Nagelkerke $pR^2 = 0.173$). NA – not applicable.

Supplementary Table 46. **Univariate and multivariable models of drooling (*n* = 207)**

**Parameter**	**Univariate model**		**Multivariable model** (unadjusted)		**Multivariable model** (stepwise selection)	
	**OR (95% CI)**	***P*-value**	**OR (95% CI)**	***P*-value**	**OR (95% CI)**	***P*-value**
Exon (Exon 11 (*n* = 43) as reference)						
Exon 3 (*n* = 22)	0.52 (0.107, 1.954)	0.364	1.26 (0.191, 8.443)	0.803	Excluded	NA
Exon 4 (*n* = 15)	1.65 (0.432, 5.895)	0.445	2.61 (0.487, 16.274)	0.272	Excluded	NA
Exon 5 (*n* = 37)	2.25 (0.867, 6.055)	0.100	4.3 (1.04, 23.038)	0.059	Excluded	NA
Exon 6 (excluded)						
Exon 7 (*n* = 25)	1.28 (0.405, 3.934)	0.663	1.96 (0.428, 10.894)	0.404	Excluded	NA
Exon 8 (*n* = 18)	0.94 (0.228, 3.368)	0.930	1.99 (0.343, 12.705)	0.444	Excluded	NA
Exon 9 (*n* = 15)	0.82 (0.164, 3.261)	0.795	1.04 (0.146, 7.404)	0.966	Excluded	NA
Exon 10 (*n* = 41)	1.37 (0.515, 3.688)	0.532	1.92 (0.44, 10.374)	0.405	Excluded	NA
Variant type (Missense (*n* = 135) as reference)						
Indels (*n* = 17)	0.19 (0.01, 0.963)	0.109	0.21 (0.011, 1.254)	0.158	0.18 (0.01, 0.966)	0.109
Frameshift or Nonsense (NMD-escape) (*n* = 24)	1.49 (0.56, 3.699)	0.406	2.66 (0.602, 14.424)	0.215	1.28 (0.47, 3.247)	0.618
Frameshift or Nonsense (NMD-predicted) (*n* = 28)	1.65 (0.675, 3.87)	0.256	1.59 (0.539, 4.704)	0.396	1.3 (0.509, 3.208)	0.572
Splice (*n* = 12)	2.97 (0.876, 10.1)	0.074	2.06 (0.536, 7.891)	0.286	2.87 (0.839, 9.864)	0.086
Source (Published (*n* = 21) as reference)						
Unpublished (*n* = 195)	0.73 (0.286, 2.01)	0.516	0.49 (0.159, 1.589)	0.223	Excluded	NA
Sex (Females (*n* = 127) as reference)						
Males (*n* = 89)	0.53 (0.276, 0.993)	0.052	0.54 (0.236, 1.185)	0.128	0.59 (0.29, 1.159)	0.129
Presentation (Childhood (*n* = 40) as reference)						
Neonatal (*n* = 90)	2.13 (0.876, 5.762)	0.111	1.1 (0.365, 3.559)	0.863	Excluded	NA
Infantile (*n* = 86)	1.82 (0.74, 4.987)	0.211	1.11 (0.399, 3.325)	0.839	Excluded	NA

Analysis includes cases with available sex, age at presentation, drooling status (present or absent). Cases with variants in exon 1 and 6 were excluded (does not fulfil ≥ 10 cases per comparison group criteria). Multivariable model with stepwise selection was acceptable (Hosmer–Lemeshow p = 0.449) and had explained approximately 8% of variance (Nagelkerke pR^2 = 0.078). NA – not applicable.

Supplementary Table 47. **Univariate and multivariable models of cerebral atrophy (*n* = 448)**

**Parameter**	**Univariate model**		**Multivariable model** (unadjusted)		**Multivariable model** (stepwise selection)	
	**OR (95% CI)**	***P*-value**	**OR (95% CI)**	***P*-value**	**OR (95% CI)**	***P*-value**
Exon (Exon 11 (*n* = 98) as reference)						
Exon 3 (*n* = 43)	0.74 (0.348, 1.554)	0.438	1.32 (0.495, 3.515)	0.578	Excluded	NA
Exon 4 (*n* = 30)	0.93 (0.395, 2.118)	0.858	0.4 (0.141, 1.118)	0.083	Excluded	NA
Exon 5 (*n* = 57)	1.44 (0.747, 2.787)	0.276	0.93 (0.385, 2.248)	0.869	Excluded	NA
Exon 6 (*n* = 19)	1.91 (0.712, 5.34)	0.202	2.05 (0.601, 7.24)	0.256	Excluded	NA
Exon 7 (*n* = 49)	0.88 (0.433, 1.767)	0.722	0.74 (0.295, 1.862)	0.527	Excluded	NA
Exon 8 (*n* = 43)	0.23 (0.08, 0.55)	0.002	0.31 (0.09, 0.948)	0.048	Excluded	NA
Exon 9 (*n* = 30)	2.09 (0.914, 4.9)	0.084	1.2 (0.409, 3.611)	0.737	Excluded	NA
Exon 10 (*n* = 79)	1.29 (0.71, 2.346)	0.405	0.51 (0.223, 1.139)	0.101	Excluded	NA
Variant type (Missense (*n* = 292) as reference)						
Indels (*n* = 35)	1.33 (0.652, 2.7)	0.423	1.05 (0.444, 2.485)	0.909	Excluded	NA
Frameshift or Nonsense (NMD-escape) (*n* = 44)	1.1 (0.568, 2.08)	0.779	0.47 (0.183, 1.203)	0.118	Excluded	NA
Frameshift or Nonsense (NMD-predicted) (*n* = 47)	2.55 (1.366, 4.884)	0.004	1.14 (0.526, 2.48)	0.747	Excluded	NA
Splice (*n* = 30)	1.21 (0.557, 2.58)	0.621	1.11 (0.451, 2.717)	0.812	Excluded	NA
Source (Published (*n* = 218) as reference)						
Unpublished (*n* = 230)	2.98 (2.022, 4.427)	< 0.001	2.73 (1.75, 4.302)	< 0.001	2.58 (1.696, 3.963)	< 0.001
Sex (Females (*n* = 232) as reference)						
Males (*n* = 216)	0.19 (0.127, 0.29)	< 0.001	0.21 (0.126, 0.348)	< 0.001	0.25 (0.159, 0.379)	< 0.001
Presentation (Childhood (*n* = 80) as reference)						
Neonatal (*n* = 200)	3.78 (2.108, 7.085)	< 0.001	3.06 (1.498, 6.456)	0.003	2.7 (1.418, 5.332)	0.003
Infantile (*n* = 169)	2.71 (1.489, 5.146)	0.002	2.59 (1.296, 5.353)	0.008	2.37 (1.237, 4.72)	0.011

Analysis includes cases with available sex, age at presentation, cerebral atrophy status (present or absent). Cases with variants in exon 1 were excluded (does not fulfil ≥ 10 cases per comparison group criteria). Multivariable model with stepwise selection was acceptable (Hosmer–Lemeshow p = 0.669) and had explained approximately 26% of variance (Nagelkerke $pR^2 = 0.257$). NA – not applicable.

Supplementary Table 48. **Univariate and multivariable models of basal ganglia findings (*n* = 448)**

**Parameter**	**Univariate model**		**Multivariable model** (unadjusted)		**Multivariable model** (stepwise selection)	
	**OR (95% CI)**	***P*-value**	**OR (95% CI)**	***P*-value**	**OR (95% CI)**	***P*-value**
Exon (Exon 11 (*n* = 98) as reference)						
Exon 3 (*n* = 43)	2.61 (1.262, 5.521)	0.010	0.59 (0.231, 1.492)	0.265	0.59 (0.231, 1.492)	0.265
Exon 4 (*n* = 30)	0.81 (0.32, 1.911)	0.634	0.59 (0.205, 1.639)	0.322	0.59 (0.205, 1.639)	0.322
Exon 5 (*n* = 57)	1.69 (0.871, 3.31)	0.121	0.75 (0.315, 1.779)	0.519	0.75 (0.315, 1.779)	0.519
Exon 6 (*n* = 19)	0.67 (0.203, 1.925)	0.480	0.21 (0.053, 0.74)	0.019	0.21 (0.053, 0.74)	0.019
Exon 7 (*n* = 49)	1.67 (0.827, 3.359)	0.152	0.8 (0.333, 1.904)	0.614	0.8 (0.333, 1.904)	0.614
Exon 8 (*n* = 43)	3.51 (1.678, 7.609)	0.001	0.86 (0.335, 2.242)	0.759	0.86 (0.335, 2.242)	0.759
Exon 9 (*n* = 30)	0.68 (0.262, 1.651)	0.414	0.48 (0.15, 1.453)	0.204	0.48 (0.15, 1.453)	0.204
Exon 10 (*n* = 79)	0.56 (0.28, 1.076)	0.086	0.65 (0.279, 1.51)	0.324	0.65 (0.279, 1.51)	0.324
Variant type (Missense (*n* = 292) as reference)						
Indels (*n* = 35)	0.51 (0.233, 1.059)	0.080	0.47 (0.182, 1.133)	0.100	0.47 (0.182, 1.133)	0.100
Frameshift or Nonsense (NMD-escape) (*n* = 44)	0.29 (0.126, 0.594)	0.001	0.23 (0.079, 0.612)	0.004	0.23 (0.079, 0.612)	0.004
Frameshift or Nonsense (NMD-predicted) (*n* = 47)	0.16 (0.061, 0.369)	< 0.001	0.45 (0.151, 1.177)	0.122	0.45 (0.151, 1.177)	0.122
Splice (*n* = 30)	0.85 (0.393, 1.814)	0.682	1.41 (0.585, 3.326)	0.440	1.41 (0.585, 3.326)	0.440
Source (Published (*n* = 218) as reference)						
Unpublished (*n* = 230)	1.21 (0.829, 1.773)	0.322	1.91 (1.206, 3.072)	0.006	1.91 (1.206, 3.072)	0.006
Sex (Females (*n* = 232) as reference)						
Males (*n* = 216)	4 (2.687, 6.024)	< 0.001	3.07 (1.877, 5.095)	< 0.001	3.07 (1.877, 5.095)	< 0.001
Presentation (Childhood (*n* = 80) as reference)						
Neonatal (*n* = 200)	0.14 (0.08, 0.252)	< 0.001	0.18 (0.094, 0.354)	< 0.001	0.18 (0.094, 0.354)	< 0.001
Infantile (*n* = 169)	0.41 (0.231, 0.706)	0.002	0.42 (0.227, 0.777)	0.006	0.42 (0.227, 0.777)	0.006

Analysis includes cases with available sex, age at presentation, basal ganglia findings status (present or absent). Cases with variants in exon 1 were excluded (does not fulfil ≥ 10 cases per comparison group criteria). Multivariable model with stepwise selection was acceptable (Hosmer–Lemeshow p = 0.165) and had explained approximately 29% of variance (Nagelkerke $pR^2 = 0.292$). NA – not applicable.

Supplementary Table 49. **Univariate and multivariable models of corpus callosum malformations (*n* = 448)**

Parameter	Univariate model		Multivariable model (unadjusted)		Multivariable model (stepwise selection)	
	**OR (95% CI)**	***P*-value**	**OR (95% CI)**	***P*-value**	**OR (95% CI)**	***P*-value**
Exon (Exon 11 (*n* = 98) as reference)						
Exon 3 (*n* = 43)	0.06 (0.003, 0.295)	0.006	0.2 (0.01, 1.222)	0.146	0.13 (0.007, 0.722)	0.059
Exon 4 (*n* = 30)	0.91 (0.345, 2.22)	0.839	1.07 (0.332, 3.358)	0.914	0.69 (0.247, 1.783)	0.451
Exon 5 (*n* = 57)	0.98 (0.466, 2)	0.947	1.92 (0.715, 5.343)	0.200	1.26 (0.568, 2.737)	0.569
Exon 6 (*n* = 19)	1.82 (0.643, 4.978)	0.246	4.3 (1.175, 16.067)	0.028	2.86 (0.884, 9.209)	0.076
Exon 7 (*n* = 49)	1.1 (0.514, 2.316)	0.798	2.18 (0.802, 6.118)	0.131	1.44 (0.625, 3.279)	0.387
Exon 8 (*n* = 43)	0.12 (0.019, 0.435)	0.005	0.45 (0.063, 2.08)	0.355	0.32 (0.047, 1.238)	0.146
Exon 9 (*n* = 30)	2.86 (1.236, 6.709)	0.014	3.57 (1.179, 11.245)	0.026	2.44 (0.971, 6.281)	0.059
Exon 10 (*n* = 79)	1.89 (1.014, 3.55)	0.046	1.59 (0.661, 3.989)	0.306	1.09 (0.549, 2.168)	0.804
Variant type (Missense (*n* = 292) as reference)						
Indels (*n* = 35)	1.19 (0.503, 2.57)	0.679	1.02 (0.378, 2.603)	0.973	Excluded	NA
Frameshift or Nonsense (NMD-escape) (*n* = 44)	2.37 (1.21, 4.569)	0.010	2.44 (0.911, 6.774)	0.080	Excluded	NA
Frameshift or Nonsense (NMD-predicted) (*n* = 47)	3.89 (2.064, 7.396)	< 0.001	1.23 (0.568, 2.702)	0.595	Excluded	NA
Splice (*n* = 30)	1.71 (0.737, 3.762)	0.192	1.04 (0.404, 2.598)	0.928	Excluded	NA
Source (Published (*n* = 218) as reference)						
Unpublished (*n* = 230)	0.97 (0.643, 1.462)	0.881	0.63 (0.379, 1.033)	0.069	0.66 (0.402, 1.069)	0.093
Sex (Females (*n* = 232) as reference)						
Males (*n* = 216)	0.19 (0.113, 0.296)	< 0.001	0.25 (0.136, 0.431)	< 0.001	0.23 (0.127, 0.39)	< 0.001
Presentation (Childhood (*n* = 80) as reference)						
Neonatal (*n* = 200)	8.36 (3.905, 20.78)	< 0.001	3.96 (1.688, 10.473)	0.003	4.07 (1.747, 10.726)	0.002
Infantile (*n* = 169)	2.45 (1.088, 6.3)	0.042	1.45 (0.593, 3.93)	0.437	1.45 (0.596, 3.941)	0.431

Analysis includes cases with available sex, age at presentation, corpus callosum malformations status (present or absent). Cases with variants in exon 1 were excluded (does not fulfil ≥ 10 cases per comparison group criteria). Multivariable model with stepwise selection was acceptable (Hosmer–Lemeshow p = 0.730) and had explained approximately 31% of variance (Nagelkerke $pR^2 = 0.314$). NA – not applicable.

Supplementary Table 50. **Univariate and multivariable models of ventriculomegaly or hydrocephalus (*n* = 448)**

**Parameter**	**Univariate model**		**Multivariable model** (unadjusted)		**Multivariable model** (stepwise selection)	
	**OR (95% CI)**	***P*-value**	**OR (95% CI)**	***P*-value**	**OR (95% CI)**	***P*-value**
Exon (Exon 11 (*n* = 98) as reference)						
Exon 3 (*n* = 43)	0.53 (0.183, 1.34)	0.203	0.92 (0.263, 3.031)	0.896	Excluded	NA
Exon 4 (*n* = 30)	0.65 (0.202, 1.783)	0.433	0.43 (0.112, 1.467)	0.190	Excluded	NA
Exon 5 (*n* = 57)	0.46 (0.17, 1.096)	0.094	0.37 (0.113, 1.133)	0.088	Excluded	NA
Exon 6 (*n* = 19)	1.51 (0.483, 4.284)	0.456	1.48 (0.384, 5.469)	0.559	Excluded	NA
Exon 7 (*n* = 49)	0.73 (0.297, 1.691)	0.481	0.78 (0.262, 2.256)	0.645	Excluded	NA
Exon 8 (*n* = 43)	0.24 (0.056, 0.758)	0.029	0.58 (0.115, 2.275)	0.464	Excluded	NA
Exon 9 (*n* = 30)	2.85 (1.208, 6.762)	0.016	1.9 (0.631, 5.833)	0.254	Excluded	NA
Exon 10 (*n* = 79)	1.69 (0.877, 3.294)	0.117	0.92 (0.374, 2.296)	0.857	Excluded	NA
Variant type (Missense (*n* = 292) as reference)						
Indels (*n* = 35)	1.53 (0.644, 3.336)	0.309	1.1 (0.409, 2.815)	0.839	Excluded	NA
Frameshift or Nonsense (NMD-escape) (*n* = 44)	1.3 (0.577, 2.701)	0.506	0.75 (0.255, 2.156)	0.594	Excluded	NA
Frameshift or Nonsense (NMD-predicted) (*n* = 47)	2.74 (1.399, 5.253)	0.003	0.86 (0.386, 1.867)	0.700	Excluded	NA
Splice (*n* = 30)	1.89 (0.784, 4.242)	0.135	1.92 (0.689, 5.203)	0.201	Excluded	NA
Source (Published (*n* = 218) as reference)						
Unpublished (*n* = 230)	0.8 (0.511, 1.248)	0.325	0.5 (0.288, 0.842)	0.010	0.45 (0.265, 0.75)	0.002
Sex (Females (*n* = 232) as reference)						
Males (*n* = 216)	0.19 (0.111, 0.325)	< 0.001	0.24 (0.121, 0.448)	< 0.001	0.24 (0.133, 0.432)	< 0.001
Presentation (Childhood (*n* = 80) as reference)						
Neonatal (*n* = 200)	3.8 (1.868, 8.579)	0.001	1.94 (0.831, 4.897)	0.140	1.99 (0.919, 4.707)	0.095
Infantile (*n* = 169)	1.44 (0.664, 3.412)	0.373	0.9 (0.372, 2.303)	0.818	0.86 (0.367, 2.121)	0.724
Cerebral atrophy (Absent (*n* = 259) as reference)						
Present (*n* = 189)	3.17 (2.007, 5.079)	< 0.001	2.24 (1.297, 3.908)	0.004	2.41 (1.42, 4.144)	0.001

Analysis includes cases with available sex, age at presentation, corpus callosum malformations status (present or absent). Cases with variants in exon 1 were excluded (does not fulfil ≥ 10 cases per comparison group criteria). Multivariable model with stepwise selection was acceptable (Hosmer–Lemeshow p = 0.288) and had explained approximately 23% of variance (Nagelkerke pR^2 = 0.226). NA – not applicable.

Supplementary Table 51. **Heterogeneity of enzyme activity values among cases with available calculation methods**

Tissue	Calculation method			Comparison between different methods
	Cutoff of normal (*n* = 67)	Lowest of normal (*n* = 31)	Mean of normal (*n* = 76)	
Fibroblasts	42.4% (IQR 24.6, range 10-88.1, *n* = 22)	59.3% (IQR 48.8, range 14.7-100, *n* = 16)	25.1% (IQR 24, range 3-100, *n* = 62)	Mean *vs*. Lowest, *p* = 0.001<br>Mean *vs*. Cutoff, *p* = 0.002<br>Lowest *vs*. Cutoff, *p* = 0.659
Lymphocytes	97% (IQR 66.3, range 0-100, *n* = 26)	0	19% (IQR 41.4, range 1-100, *n* = 12)	NS
Muscle	48.3% (IQR 75.8, range 2-100, *n* = 19)	37.7% (IQR 42.0, range 5.6-100, *n* = 15)	Range 11-33% (*n* = 2)	NS
Comparison between tissues	NS	NS	NS	–
Overall	48.3% (IQR 60.5)<br>(range 0-100)	47.1% (IQR 45.8)<br>(range 5.6-100)	24% (IQR 26.2)<br>(range 1-100)	Mean *vs*. Lowest, *p* = 0.001<br>Mean *vs*. Cutoff, *p* = 0.0002<br>Lowest *vs*. Cutoff, *p* = 1

Supplementary table 51 presents the differences in residual PDHc enzyme activity values in a subset of cases where the enzyme activity calculation methods were reported. Enzyme activity is expressed as the median (%) with the interquartile range (IQR) and minimum-maximum range. NS – Not significant. Cutoff: calculation method "Cutoff of normal," where enzyme activity values are expressed as percentages relative to a specific cutoff value. Mean: calculation method "Mean of normal," where enzyme activity values are expressed as percentages relative to the mean activity of the control group. Lowest: calculation method "Lowest of normal," where enzyme activity values are expressed as percentages relative to the lowest activity value within the control group, as defined by the normal range.

Supplementary references

1. Endo H, Hasegawa K, Narisawa K, Tada K, Kagawa Y, Ohta S. Defective gene in lactic acidosis: abnormal pyruvate dehydrogenase E1 alpha-subunit caused by a frame shift. *Am J Hum Genet*. 1989;44(3):358-364.

2. Dahl HH, Maragos C, Brown RM, Hansen LL, Brown GK. Pyruvate dehydrogenase deficiency caused by deletion of a 7-bp repeat sequence in the E1 alpha gene. *Am J Hum Genet*. 1990;47(2):286-293.

3. Chun K, MacKay N, Petrova-Benedict R, Robinson BH. Pyruvate dehydrogenase deficiency due to a 20-bp deletion in exon II of the pyruvate dehydrogenase (PDH) E1 alpha gene. *Am J Hum Genet*. 1991;49(2):414-420.

4. de Meirleir LJ, Lissens W, Vamos E, Liebaers I. Pyruvate dehydrogenase deficiency due to a mutation of the E1 alpha subunit. *J Inherit Metab Dis*. 1991;14(3):301-304. doi:10.1007/BF01811687

5. Endo H, Miyabayashi S, Tada K, Narisawa K. A four-nucleotide insertion at the E1 alpha gene in a patient with pyruvate dehydrogenase deficiency. *J Inherit Metab Dis*. 1991;14(5):793-799. doi:10.1007/BF01799952

6. Ito M, Huq AH, Naito E, Saijo T, Takeda E, Kuroda Y. Mutation of E1 alpha gene in a female patient with pyruvate dehydrogenase deficiency due to rapid degradation of E1 protein. *J Inherit Metab Dis*. 1992;15(6):848-856. doi:10.1007/BF01800220

7. De Meirleir L, Lissens W, Vamos E, Liebaers I. Pyruvate dehydrogenase (PDH) deficiency caused by a 21-base pair insertion mutation in the E1 alpha subunit. *Hum Genet*. 1992;88(6):649-652. doi:10.1007/BF02265291

8. Matthews PM, Brown RM, Otero L, Marchington D, Leonard JV, Brown GK. Neurodevelopmental abnormalities and lactic acidosis in a girl with a 20-bp deletion in the X-linked pyruvate dehydrogenase E1 alpha subunit gene. *Neurology*. 1993;43(10):2025-2030. doi:10.1212/wnl.43.10.2025

9. Hansen LL, Brown GK, Brown RM, Dahl HH. Pyruvate dehydrogenase deficiency caused by a 5 base pair duplication in the E1 alpha subunit. *Hum Mol Genet*. 1993;2(6):805-807. doi:10.1093/hmg/2.6.805

10. Takakubo F, Thorburn DR, Dahl HH. A four-nucleotide insertion hotspot in the X chromosome located pyruvate dehydrogenase E1 alpha gene (PDHA1). *Hum Mol Genet*. 1993;2(4):473-474. doi:10.1093/hmg/2.4.473

11. Takakubo F, Thorburn DR, Dahl HH. A novel mutation and a polymorphism in the X chromosome located pyruvate dehydrogenase E1 alpha gene (PDHA1). *Hum Mol Genet*. 1993;2(11):1961-1962. doi:10.1093/hmg/2.11.1961

12. De Meirleir L, Lissens W, Denis R, et al. Pyruvate dehydrogenase deficiency: clinical and biochemical diagnosis. *Pediatr Neurol*. 1993;9(3):216-220. doi:10.1016/0887-8994(93)90088-t

13. Naito E, Ito M, Takeda E, Yokota I, Yoshijima S, Kuroda Y. Molecular analysis of abnormal pyruvate dehydrogenase in a patient with thiamine-responsive congenital lactic acidemia. *Pediatr Res*. 1994;36(3):340-346. doi:10.1203/00006450-199409000-00013

14. Naito E, Ito M, Yokota I, Matsuda J, Yara A, Kuroda Y. Pyruvate dehydrogenase deficiency caused by a four-nucleotide insertion in the E1 alpha subunit gene. *Hum Mol Genet*. 1994;3(7):1193-1194. doi:10.1093/hmg/3.7.1193

15. Hansen LL, Horn N, Dahl HH, Kruse TA. Pyruvate dehydrogenase deficiency caused by a 33 base pair duplication in the PDH E1 alpha subunit. *Hum Mol Genet*. 1994;3(6):1021-1022. doi:10.1093/hmg/3.6.1021

16. Dahl HH, Brown GK. Pyruvate dehydrogenase deficiency in a male caused by a point mutation (F205L) in the E1 alpha subunit. *Hum Mutat*. 1994;3(2):152-155. doi:10.1002/humu.1380030210

17. Otero LJ, Brown GK, Silver K, Arnold DL, Matthews PM. Association of cerebral dysgenesis and lactic acidemia with X-linked PDH E1 alpha subunit mutations in females. *Pediatr Neurol*. 1995;13(4):327-332. doi:10.1016/0887-8994(95)00222-7

18. Hemalatha SG, Kerr DS, Wexler ID, et al. Pyruvate dehydrogenase complex deficiency due to a point mutation (P188L) within the thiamine pyrophosphate binding loop of the E1 alpha subunit. *Hum Mol Genet*. 1995;4(2):315-318. doi:10.1093/hmg/4.2.315

19. Takakubo F, Thorburn DR, Brown RM, Brown GK, Dahl HH. A novel mutation (P316L) in a female with pyruvate dehydrogenase E1 alpha deficiency. *Hum Mutat*. 1995;6(3):274-275. doi:10.1002/humu.1380060317

20. Lissens W, Desguerre I, Benelli C, et al. Pyruvate dehydrogenase deficiency in a female due to a 4 base pair deletion in exon 10 of the E1 alpha gene. *Hum Mol Genet*. 1995;4(2):307-308. doi:10.1093/hmg/4.2.307

21. Ito M, Naito E, Yokota I, et al. Molecular genetic analysis of a female patient with pyruvate dehydrogenase deficiency: detection of a new mutation and differential expression of mutant gene product in cultured cells. *J Inherit Metab Dis*. 1995;18(5):547-557. doi:10.1007/BF02435999

22. Naito E, Ito M, Yokota I, et al. Biochemical and molecular analysis of an X-linked case of Leigh syndrome associated with thiamin-responsive pyruvate dehydrogenase deficiency. *J Inherit Metab Dis*. 1997;20(4):539-548. doi:10.1023/a:1005305614374

23. Takahashi S, Oki J, Miyamoto A, et al. Autopsy findings in pyruvate dehydrogenase E1alpha deficiency: case report. *J Child Neurol*. 1997;12(8):519-524. doi:10.1177/088307389701200812

24. Rubio-Gozalbo ME, Heerschap A, Trijbels JM, De Meirleir L, Thijssen HO, Smeitink JA. Proton MR spectroscopy in a child with pyruvate dehydrogenase complex deficiency. *Magn Reson Imaging*. 1999;17(6):939-944. doi:10.1016/s0730-725x(99)00002-8

25. Naito E, Ito M, Yokota I, et al. Concomitant administration of sodium dichloroacetate and thiamine in west syndrome caused by thiamine-responsive pyruvate dehydrogenase complex deficiency. *J Neurol Sci*. 1999;171(1):56-59. doi:10.1016/s0022-510x(99)00250-6

26. Seyda A, Chun K, Packman S, Robinson BH. A case of PDH-E1 alpha mosaicism in a male patient with severe metabolic lactic acidosis. *J Inherit Metab Dis*. 2001;24(5):551-559. doi:10.1023/a:1012463726810

27. Benelli C, Fouque F, Redonnet-Vernhet I, et al. A novel Y243S mutation in the pyruvate dehydrogenase El alpha gene subunit: correlation with thiamine pyrophosphate interaction. *J Inherit Metab Dis*. 2002;25(4):325-327. doi:10.1023/a:1016570828778

28. Miné M, Brivet M, Touati G, Grabowski P, Abitbol M, Marsac C. Splicing error in E1alpha pyruvate dehydrogenase mRNA caused by novel intronic mutation responsible for lactic acidosis and mental retardation. *J Biol Chem*. 2003;278(14):11768-11772. doi:10.1074/jbc.M211106200

29. Brown RM, Head RA, Boubriak II, Leonard JV, Brown GK. A pathogenic glutamate-to-aspartate substitution (D296E) in the pyruvate dehydrogenase E1 subunit gene PDHA1. *Hum Mutat*. 2003;22(6):496-497. doi:10.1002/humu.9198

30. Wada N, Matsuishi T, Nonaka M, Naito E, Yoshino M. Pyruvate dehydrogenase E1alpha subunit deficiency in a female patient: evidence of antenatal origin of brain damage and possible etiology of infantile spasms. *Brain Dev*. 2004;26(1):57-60. doi:10.1016/s0387-7604(03)00072-x

31. Silva MJ, Cabral A, Eusebio F, Tasso T, Gaspar A, De Almeida TI, et al. The Pyruvate Dehydrogenase Complex (PDHc) First Case Identified in Portugal. *Acta Pediatr Port*. 2004;(35:525–31).

32. Brivet M, Moutard ML, Zater M, et al. First characterization of a large deletion of the PDHA 1 gene. *Mol Genet Metab*. 2005;86(4):456-461. doi:10.1016/j.ymgme.2005.08.009

33. Lee EH, Ahn MS, Hwang JS, Ryu KH, Kim SJ, Kim SH. A Korean female patient with thiamine-responsive pyruvate dehydrogenase complex deficiency due to a novel point mutation (Y161C)in the PDHA1 gene. *J Korean Med Sci*. 2006;21(5):800-804. doi:10.3346/jkms.2006.21.5.800

34. Okajima K, Warman ML, Byrne LC, Kerr DS. Somatic mosaicism in a male with an exon skipping mutation in PDHA1 of the pyruvate dehydrogenase complex results in a milder phenotype. *Mol Genet Metab*. 2006;87(2):162-168. doi:10.1016/j.ymgme.2005.09.023

35. Ridout CK, Brown RM, Walter JH, Brown GK. Somatic mosaicism for a PDHA1 mutation in a female with pyruvate dehydrogenase deficiency. *Hum Genet*. 2008;124(2):187-193. doi:10.1007/s00439-008-0538-0

36. Ridout CK, Keighley P, Krywawych S, Brown RM, Brown GK. A putative exonic splicing enhancer in exon 7 of the PDHA1 gene affects splicing of adjacent exons. *Hum Mutat*. 2008;29(3):451. doi:10.1002/humu.9525

37. Soares-Fernandes JP, Teixeira-Gomes R, Cruz R, et al. Neonatal pyruvate dehydrogenase deficiency due to a R302H mutation in the PDHA1 gene: MRI findings. *Pediatr Radiol*. 2008;38(5):559-562. doi:10.1007/s00247-007-0721-9

38. Sedel F, Challe G, Mayer JM, et al. Thiamine responsive pyruvate dehydrogenase deficiency in an adult with peripheral neuropathy and optic neuropathy. *J Neurol Neurosurg Psychiatry*. 2008;79(7):846-847. doi:10.1136/jnnp.2007.136630

39. Bachmann-Gagescu R, Merritt JL, Hahn SH. A cognitively normal PDH-deficient 18-year-old man carrying the R263G mutation in the PDHA1 gene. *J Inherit Metab Dis*. 2009;32 Suppl 1. doi:10.1007/s10545-009-1101-4

40. João Silva M, Pinheiro A, Eusébio F, Gaspar A, Tavares de Almeida I, Rivera I. Pyruvate dehydrogenase deficiency: identification of a novel mutation in the PDHA1 gene which responds to amino acid supplementation. *Eur J Pediatr*. 2009;168(1):17-22. doi:10.1007/s00431-008-0700-7

41. Coughlin CR, Krantz ID, Schmitt ES, et al. Somatic mosaicism for PDHA1 mutation in a male with pyruvate dehydrogenase complex deficiency. *Mol Genet Metab*. 2010;100(3):296-299. doi:10.1016/j.ymgme.2010.04.004

42. Tamaru S, Kikuchi A, Takagi K, et al. A case of pyruvate dehydrogenase E1α subunit deficiency with antenatal brain dysgenesis demonstrated by prenatal sonography and magnetic resonance imaging. *J Clin Ultrasound JCU*. 2012;40(4):234-238. doi:10.1002/jcu.20864

43. Koga Y, Povalko N, Katayama K, et al. Beneficial effect of pyruvate therapy on Leigh syndrome due to a novel mutation in PDH E1α gene. *Brain Dev*. 2012;34(2):87-91. doi:10.1016/j.braindev.2011.03.003

44. Giribaldi G, Doria-Lamba L, Biancheri R, et al. Intermittent-relapsing pyruvate dehydrogenase complex deficiency: a case with clinical, biochemical, and neuroradiological reversibility. *Dev Med Child Neurol*. 2012;54(5):472-476. doi:10.1111/j.1469-8749.2011.04151.x

45. Deeb KK, Bedoyan JK, Wang R, et al. Somatic mosaicism for a novel PDHA1 mutation in a male with severe pyruvate dehydrogenase complex deficiency. *Mol Genet Metab Rep*. 2014;1:362-367. doi:10.1016/j.ymgmr.2014.08.001

46. Steller J, Gargus JJ, Gibbs LH, Hasso AN, Kimonis VE. Mild phenotype in a male with pyruvate dehydrogenase complex deficiency associated with novel hemizygous in-frame duplication of the E1α subunit gene (PDHA1). *Neuropediatrics*. 2014;45(1):56-60. doi:10.1055/s-0033-1341601

47. Kim JA, Yu R, Jo W, et al. A modified Atkin's diet for an infant with pyruvate dehydrogenase complex deficiency confirmed by PDHA1 gene mutation.

48. Castiglioni C, Verrigni D, Okuma C, et al. Pyruvate dehydrogenase deficiency presenting as isolated paroxysmal exercise induced dystonia successfully reversed with thiamine supplementation. Case report and mini-review. *Eur J Paediatr Neurol EJPN Off J Eur Paediatr Neurol Soc*. 2015;19(5):497-503. doi:10.1016/j.ejpn.2015.04.008

49. Jauhari P, Sankhyan N, Vyas S, Singhi P. Thiamine Responsive Pyruvate Dehydrogenase Complex Deficiency: A Potentially Treatable Cause of Leigh's Disease. *J Pediatr Neurosci*. 2017;12(3):265-267. doi:10.4103/jpn.JPN_191_16

50. Kara B, Genç HM, Uyur-Yalçın E, et al. Pyruvate dehydrogenase-E1α deficiency presenting as recurrent acute proximal muscle weakness of upper and lower extremities in an 8-year-old boy. *Neuromuscul Disord NMD*. 2017;27(1):94-97. doi:10.1016/j.nmd.2016.11.001

51. Kim JH, Kim HR, Jang JH, Jo HS, Lee KH. *A Case of Early Diagnosis of Pyruvate Dehydrogenase Complex Deficiency: The Use of Next-Generation Sequencing*. Iranian Journal of Pediatrics; 2019. Accessed June 16, 2025. https://brieflands.com/articles/ijp-84965#abstract

52. Ma Y, Zhang Y, Zhang T, et al. Pyruvate dehydrogenase deficiency disease detected by the enzyme activity of peripheral leukocytes. *Mol Genet Genomic Med*. 2021;9(8):e1728. doi:10.1002/mgg3.1728

53. Gong K, Xie L, Wu ZS, Xie X, Zhang XX, Chen JL. Clinical exome sequencing reveals a mutation in PDHA1 in Leigh syndrome: A case of a Chinese boy with lethal neuropathy. *Mol Genet Genomic Med*. 2021;9(4):e1651. doi:10.1002/mgg3.1651

54. Pavuluri H, F A, Menon RN, Nair SS, Sundaram S. Pyruvate Dehydrogenase Complex Deficiency Due to PDHA1 Mutation-A Rare Treatable Cause for Episodic Ataxia in Children. *Indian J Pediatr*. 2022;89(5):519. doi:10.1007/s12098-021-04068-x

55. Hayano S, Amamoto M, Naito E. Thiamine-responsive pyruvate dehydrogenase complex deficiency presenting as recurrent muscle weakness: Identification of a novel mutation (p.T111I) in the PDHA1 gene. *Pediatr Int Off J Jpn Pediatr Soc*. 2023;65(1):e15385. doi:10.1111/ped.15385

56. Tanner LM, Tynninen O, Piippo K, Puhakka AM. X-linked pyruvate dehydrogenase complex deficiency due to a novel PDHA1 variant associated with structural brain abnormalities in a fetus. *Prenat Diagn*. 2023;43(6):730-733. doi:10.1002/pd.6349

57. de Gusmao CM, Peixoto de Barcelos I, Pinto ALR, Silveira-Moriyama L. Pearls & Oy-sters: Paroxysmal Exercise-Induced Dyskinesias Due to Pyruvate Dehydrogenase Deficiency. *Neurology*. 2023;101(1):46-49. doi:10.1212/WNL.0000000000207142

58. Croci C, Cataldi M, Baratto S, et al. Recurrent Sensory-Motor Neuropathy Mimicking CIDP as Predominant Presentation of PDH Deficiency. *Neuropediatrics*. 2023;54(3):211-216. doi:10.1055/a-2018-4845

59. Laxmi V, Gunasekaran PK, Kumar A, Manjunathan S, Tiwari S, Saini L. Acute Flaccid Paralysis due to Pyruvate Dehydrogenase E1-Alpha Deficiency. *Indian J Pediatr*. 2024;91(5):518. doi:10.1007/s12098-023-04853-w

60. Fecarotta S, D'Amico A, Di Gennaro S, et al. Metabolic stroke-like events in a girl with pyruvate dehydrogenase complex deficiency caused by a novel de novo mutation in PDHA1. *Neurol Sci Off J Ital Neurol Soc Ital Soc Clin Neurophysiol*. 2024;45(6):2913-2914. doi:10.1007/s10072-024-07355-5

61. Moola S, Munn Z, Sears K, et al. Conducting systematic reviews of association (etiology): The Joanna Briggs Institute's approach. *Int J Evid Based Healthc*. 2015;13(3):163-169. doi:10.1097/XEB.0000000000000064

62. Hansen LL, Brown GK, Kirby DM, Dahl HH. Characterization of the mutations in three patients with pyruvate dehydrogenase E1 alpha deficiency. *J Inherit Metab Dis*. 1991;14(2):140-151. doi:10.1007/BF01800586

63. Wexler ID, Hemalatha SG, Liu TC, Berry SA, Kerr DS, Patel MS. A mutation in the E1 alpha subunit of pyruvate dehydrogenase associated with variable expression of pyruvate dehydrogenase complex deficiency. *Pediatr Res*. 1992;32(2):169-174. doi:10.1203/00006450-199208000-00009

64. Dahl HH, Hansen LL, Brown RM, Danks DM, Rogers JG, Brown GK. X-linked pyruvate dehydrogenase E1 alpha subunit deficiency in heterozygous females: variable manifestation of the same mutation. *J Inherit Metab Dis*. 1992;15(6):835-847. doi:10.1007/BF01800219

65. Chun K, MacKay N, Petrova-Benedict R, Robinson BH. Mutations in the X-linked E1 alpha subunit of pyruvate dehydrogenase leading to deficiency of the pyruvate dehydrogenase complex. *Hum Mol Genet*. 1993;2(4):449-454. doi:10.1093/hmg/2.4.449

66. De Meirleir L, Lissens W, Benelli C, et al. Aberrant splicing of exon 6 in the pyruvate dehydrogenase-E1 alpha mRNA linked to a silent mutation in a large family with Leigh's encephalomyelopathy. *Pediatr Res*. 1994;36(6):707-712. doi:10.1203/00006450-199412000-00004

67. Awata H, Endo F, Tanoue A, Kitano A, Matsuda I. Characterization of a point mutation in the pyruvate dehydrogenase E1 alpha gene from two boys with primary lactic acidaemia. *J Inherit Metab Dis*. 1994;17(2):189-195. doi:10.1007/BF00711616

68. Brown RM, Brown GK. Prenatal diagnosis of pyruvate dehydrogenase E1 alpha subunit deficiency. *Prenat Diagn*. 1994;14(6):435-441. doi:10.1002/pd.1970140604

69. Matthews PM, Brown RM, Otero LJ, et al. Pyruvate dehydrogenase deficiency. Clinical presentation and molecular genetic characterization of five new patients. *Brain J Neurol*. 1994;117 (Pt 3):435-443. doi:10.1093/brain/117.3.435

70. Takakubo F, Cartwright P, Hoogenraad N, et al. An amino acid substitution in the pyruvate dehydrogenase E1 alpha gene, affecting mitochondrial import of the precursor protein. *Am J Hum Genet*. 1995;57(4):772-780.

71. Chun K, MacKay N, Petrova-Benedict R, et al. Mutations in the X-linked E1 alpha subunit of pyruvate dehydrogenase: exon skipping, insertion of duplicate sequence, and missense mutations leading to the deficiency of the pyruvate dehydrogenase complex. *Am J Hum Genet*. 1995;56(3):558-569.

72. Matsuda J, Ito M, Naito E, Yokota I, Kuroda Y. DNA diagnosis of pyruvate dehydrogenase deficiency in female patients with congenital lactic acidaemia. *J Inherit Metab Dis*. 1995;18(5):534-546. doi:10.1007/BF02435998

73. Fujii T, Garcia Alvarez MB, Sheu KF, Kranz-Eble PJ, De Vivo DC. Pyruvate dehydrogenase deficiency: the relation of the E1 alpha mutation to the E1 beta subunit deficiency. *Pediatr Neurol*. 1996;14(4):328-334. doi:10.1016/0887-8994(96)00058-6

74. Lissens W, De Meirleir L, Seneca S, et al. Mutation analysis of the pyruvate dehydrogenase E1 alpha gene in eight patients with a pyruvate dehydrogenase complex deficiency. *Hum Mutat*. 1996;7(1):46-51. doi:10.1002/(SICI)1098-1004(1996)7:1<46::AID-HUMU6>3.0.CO;2-N

75. Tripatara A, Kerr DS, Lusk MM, Kolli M, Tan J, Patel MS. Three new mutations of the pyruvate dehydrogenase alpha subunit: a point mutation (M181V), 3 bp deletion (-R282), and 16 bp insertion/frameshift (K358SVS-->TVDQS). *Hum Mutat*. 1996;8(2):180-182. doi:10.1002/(SICI)1098-1004(1996)8:2<180::AID-HUMU11>3.0.CO;2-Z

76. Marsac C, Benelli C, Desguerre I, et al. Biochemical and genetic studies of four patients with pyruvate dehydrogenase E1 alpha deficiency. *Hum Genet*. 1997;99(6):785-792. doi:10.1007/s004390050449

77. Wexler ID, Hemalatha SG, McConnell J, et al. Outcome of pyruvate dehydrogenase deficiency treated with ketogenic diets. Studies in patients with identical mutations. *Neurology*. 1997;49(6):1655-1661. doi:10.1212/wnl.49.6.1655

78. De Meirleir L, Specola N, Seneca S, Lissens W. Pyruvate dehydrogenase E1 alpha deficiency in a family: different clinical presentation in two siblings. *J Inherit Metab Dis*. 1998;21(3):224-226. doi:10.1023/a:1005347501111

79. Otero LJ, Brown RM, Brown GK. Arginine 302 mutations in the pyruvate dehydrogenase E1alpha subunit gene: identification of further patients and in vitro demonstration of pathogenicity. *Hum Mutat*. 1998;12(2):114-121. doi:10.1002/(SICI)1098-1004(1998)12:2<114::AID-HUMU6>3.0.CO;2-#

80. Lissens W, Vreken P, Barth PG, et al. Cerebral palsy and pyruvate dehydrogenase deficiency: identification of two new mutations in the E1alpha gene. *Eur J Pediatr*. 1999;158(10):853-857. doi:10.1007/s004310051222

81. Lissens W, De Meirleir L, Seneca S, et al. Mutations in the X-linked pyruvate dehydrogenase (E1) alpha subunit gene (PDHA1) in patients with a pyruvate dehydrogenase complex deficiency. *Hum Mutat*. 2000;15(3):209-219. doi:10.1002/(SICI)1098-1004(200003)15:3<209::AID-HUMU1>3.0.CO;2-K

82. Naito E, Ito M, Yokota I, et al. Gender-specific occurrence of West syndrome in patients with pyruvate dehydrogenase complex deficiency. *Neuropediatrics*. 2001;32(6):295-298. doi:10.1055/s-2001-20404

83. Naito E, Ito M, Yokota I, et al. Thiamine-responsive pyruvate dehydrogenase deficiency in two patients caused by a point mutation (F205L and L216F) within the thiamine pyrophosphate binding region. *Biochim Biophys Acta*. 2002;1588(1):79-84. doi:10.1016/s0925-4439(02)00142-4

84. Naito E, Ito M, Yokota I, Saijo T, Ogawa Y, Kuroda Y. Diagnosis and molecular analysis of three male patients with thiamine-responsive pyruvate dehydrogenase complex deficiency. *J Neurol Sci*. 2002;201(1-2):33-37. doi:10.1016/s0022-510x(02)00187-9

85. Head RA, de Goede CGEL, Newton RWN, et al. Pyruvate dehydrogenase deficiency presenting as dystonia in childhood. *Dev Med Child Neurol*. 2004;46(10):710-712. doi:10.1017/s0012162204001197

86. Cameron JM, Levandovskiy V, Mackay N, Tein I, Robinson BH. Deficiency of pyruvate dehydrogenase caused by novel and known mutations in the E1alpha subunit. *Am J Med Genet A*. 2004;131(1):59-66. doi:10.1002/ajmg.a.30287

87. Tulinius M, Darin N, Wiklund LM, et al. A family with pyruvate dehydrogenase complex deficiency due to a novel C>T substitution at nucleotide position 407 in exon 4 of the X-linked Epsilon1alpha gene. *Eur J Pediatr*. 2005;164(2):99-103. doi:10.1007/s00431-004-1570-2

88. Willemsen M, Rodenburg RJT, Teszas A, van den Heuvel L, Kosztolanyi G, Morava E. Females with PDHA1 gene mutations: a diagnostic challenge. *Mitochondrion*. 2006;6(3):155-159. doi:10.1016/j.mito.2006.03.001

89. Debray FG, Lambert M, Vanasse M, et al. Intermittent peripheral weakness as the presenting feature of pyruvate dehydrogenase deficiency. *Eur J Pediatr*. 2006;165(7):462-466. doi:10.1007/s00431-006-0104-5

90. Strassburg HM, Koch J, Mayr J, Sperl W, Boltshauser E. Acute flaccid paralysis as initial symptom in 4 patients with novel E1alpha mutations of the pyruvate dehydrogenase complex. *Neuropediatrics*. 2006;37(3):137-141. doi:10.1055/s-2006-924555

91. Debray FG, Lambert M, Gagne R, et al. Pyruvate dehydrogenase deficiency presenting as intermittent isolated acute ataxia. *Neuropediatrics*. 2008;39(1):20-23. doi:10.1055/s-2008-1077084

92. Boichard A, Venet L, Naas T, et al. Two silent substitutions in the PDHA1 gene cause exon 5 skipping by disruption of a putative exonic splicing enhancer. *Mol Genet Metab*. 2008;93(3):323-330. doi:10.1016/j.ymgme.2007.09.020

93. Ostergaard E, Moller LB, Kalkanoglu-Sivri HS, et al. Four novel PDHA1 mutations in pyruvate dehydrogenase deficiency. *J Inherit Metab Dis*. 2009;32 Suppl 1:S235-239. doi:10.1007/s10545-009-1179-8

94. Vasta V, Ng SB, Turner EH, Shendure J, Hahn SH. Next generation sequence analysis for mitochondrial disorders. *Genome Med*. 2009;1(10):100. doi:10.1186/gm100

95. Koene S, Kozicz TL, Rodenburg RJT, et al. Major depression in adolescent children consecutively diagnosed with mitochondrial disorder. *J Affect Disord*. 2009;114(1-3):327-332. doi:10.1016/j.jad.2008.06.023

96. Rizza T, Vazquez-Memije ME, Meschini MC, et al. Assaying ATP synthesis in cultured cells: a valuable tool for the diagnosis of patients with mitochondrial disorders. *Biochem Biophys Res Commun*. 2009;383(1):58-62. doi:10.1016/j.bbrc.2009.03.121

97. Quintana E, Gort L, Busquets C, et al. Mutational study in the PDHA1 gene of 40 patients suspected of pyruvate dehydrogenase complex deficiency. *Clin Genet*. 2010;77(5):474-482. doi:10.1111/j.1399-0004.2009.01313.x

98. Egel RT, Hoganson GE, Katerji MA, Borenstein MJ. Zonisamide ameliorates symptoms of secondary paroxysmal dystonia. *Pediatr Neurol*. 2010;43(3):205-208. doi:10.1016/j.pediatrneurol.2010.04.008

99. Imbard A, Boutron A, Vequaud C, et al. Molecular characterization of 82 patients with pyruvate dehydrogenase complex deficiency. Structural implications of novel amino acid substitutions in E1 protein. *Mol Genet Metab*. 2011;104(4):507-516. doi:10.1016/j.ymgme.2011.08.008

100. Ah Mew N, Loewenstein JB, Kadom N, et al. MRI features of 4 female patients with pyruvate dehydrogenase E1 alpha deficiency. *Pediatr Neurol*. 2011;45(1):57-59. doi:10.1016/j.pediatrneurol.2011.02.003

101. Glushakova LG, Judge S, Cruz A, Pourang D, Mathews CE, Stacpoole PW. Increased superoxide accumulation in pyruvate dehydrogenase complex deficient fibroblasts. *Mol Genet Metab*. 2011;104(3):255-260. doi:10.1016/j.ymgme.2011.07.023

102. Magner M, Vinšová K, Tesařová M, et al. Two patients with clinically distinct manifestation of pyruvate dehydrogenase deficiency due to mutations in PDHA1 gene. *Prague Med Rep*. 2011;112(1):18-28.

103. Prasad C, Rupar T, Prasad AN. Pyruvate dehydrogenase deficiency and epilepsy. *Brain Dev*. 2011;33(10):856-865. doi:10.1016/j.braindev.2011.08.003

104. de Ligt J, Willemsen MH, van Bon BWM, et al. Diagnostic exome sequencing in persons with severe intellectual disability. *N Engl J Med*. 2012;367(20):1921-1929. doi:10.1056/NEJMoa1206524

105. DeBrosse SD, Okajima K, Zhang S, et al. Spectrum of neurological and survival outcomes in pyruvate dehydrogenase complex (PDC) deficiency: lack of correlation with genotype. *Mol Genet Metab*. 2012;107(3):394-402. doi:10.1016/j.ymgme.2012.09.001

106. Patel KP, O'Brien TW, Subramony SH, Shuster J, Stacpoole PW. The spectrum of pyruvate dehydrogenase complex deficiency: clinical, biochemical and genetic features in 371 patients. *Mol Genet Metab*. 2012;105(1):34-43. doi:10.1016/j.ymgme.2011.09.032

107. Joost K, Rodenburg RJ, Piirsoo A, et al. A Diagnostic Algorithm for Mitochondrial Disorders in Estonian Children. *Mol Syndromol*. 2012;3(3):113-119. doi:10.1159/000341375

108. Ferriero R, Boutron A, Brivet M, et al. Phenylbutyrate increases pyruvate dehydrogenase complex activity in cells harboring a variety of defects. *Ann Clin Transl Neurol*. 2014;1(7):462-470. doi:10.1002/acn3.73

109. Zhu X, Petrovski S, Xie P, et al. Whole-exome sequencing in undiagnosed genetic diseases: interpreting 119 trios. *Genet Med Off J Am Coll Med Genet*. 2015;17(10):774-781. doi:10.1038/gim.2014.191

110. van Dongen S, Brown RM, Brown GK, Thorburn DR, Boneh A. Thiamine-Responsive and Non-responsive Patients with PDHC-E1 Deficiency: A Retrospective Assessment. *JIMD Rep*. 2015;15:13-27. doi:10.1007/8904_2014_293

111. Alfadhel M, Benmeakel M, Hossain MA, et al. Thirteen year retrospective review of the spectrum of inborn errors of metabolism presenting in a tertiary center in Saudi Arabia. *Orphanet J Rare Dis*. 2016;11(1):126. doi:10.1186/s13023-016-0510-3

112. Pronicka E, Piekutowska-Abramczuk D, Ciara E, et al. New perspective in diagnostics of mitochondrial disorders: two years' experience with whole-exome sequencing at a national paediatric centre. *J Transl Med*. 2016;14(1):174. doi:10.1186/s12967-016-0930-9

113. Ciara E, Rokicki D, Halat P, et al. Difficulties in recognition of pyruvate dehydrogenase complex deficiency on the basis of clinical and biochemical features. The role of next-generation sequencing. *Mol Genet Metab Rep*. 2016;7:70-76. doi:10.1016/j.ymgmr.2016.03.004

114. Qin L, Wang J, Tian X, et al. Detection and Quantification of Mosaic Mutations in Disease Genes by Next-Generation Sequencing. *J Mol Diagn JMD*. 2016;18(3):446-453. doi:10.1016/j.jmoldx.2016.01.002

115. Pirot N, Crahes M, Adle-Biassette H, et al. Phenotypic and Neuropathological Characterization of Fetal Pyruvate Dehydrogenase Deficiency. *J Neuropathol Exp Neurol*. 2016;75(3):227-238. doi:10.1093/jnen/nlv022

116. Wang J, Yu H, Zhang VW, et al. Capture-based high-coverage NGS: a powerful tool to uncover a wide spectrum of mutation types. *Genet Med Off J Am Coll Med Genet*. 2016;18(5):513-521. doi:10.1038/gim.2015.121

117. Asencio C, Rodríguez-Hernandez MA, Briones P, et al. Severe encephalopathy associated to pyruvate dehydrogenase mutations and unbalanced coenzyme Q10 content. *Eur J Hum Genet EJHG*. 2016;24(3):367-372. doi:10.1038/ejhg.2015.112

118. Yoshida T, Kido J, Mitsubuchi H, Matsumoto S, Endo F, Nakamura K. Clinical manifestations in two patients with pyruvate dehydrogenase deficiency and long-term survival. *Hum Genome Var*. 2017;4:17020. doi:10.1038/hgv.2017.20

119. Fang F, Liu Z, Fang H, et al. The clinical and genetic characteristics in children with mitochondrial disease in China. *Sci China Life Sci*. 2017;60(7):746-757. doi:10.1007/s11427-017-9080-y

120. Shin HK, Grahame G, McCandless SE, Kerr DS, Bedoyan JK. Enzymatic testing sensitivity, variability and practical diagnostic algorithm for pyruvate dehydrogenase complex (PDC) deficiency. *Mol Genet Metab*. 2017;122(3):61-66. doi:10.1016/j.ymgme.2017.09.001

121. Winters L, Van Hoof E, De Catte L, et al. Massive parallel sequencing identifies RAPSN and PDHA1 mutations causing fetal akinesia deformation sequence. *Eur J Paediatr Neurol EJPN Off J Eur Paediatr Neurol Soc*. 2017;21(5):745-753. doi:10.1016/j.ejpn.2017.04.641

122. Jou C, Ortigoza-Escobar JD, O'Callaghan MM, et al. Muscle Involvement in a Large Cohort of Pediatric Patients with Genetic Diagnosis of Mitochondrial Disease. *J Clin Med*. 2019;8(1):68. doi:10.3390/jcm8010068

123. Horga A, Woodward CE, Mills A, et al. Differential phenotypic expression of a novel PDHA1 mutation in a female monozygotic twin pair. *Hum Genet*. 2019;138(11-12):1313-1322. doi:10.1007/s00439-019-02075-9

124. Zouvelou V, Yubero D, Apostolakopoulou L, et al. The genetic etiology in cerebral palsy mimics: The results from a Greek tertiary care center. *Eur J Paediatr Neurol EJPN Off J Eur Paediatr Neurol Soc*. 2019;23(3):427-437. doi:10.1016/j.ejpn.2019.02.001

125. Dong HL, Ma Y, Li QF, et al. Genetic and clinical features of Chinese patients with mitochondrial ataxia identified by targeted next-generation sequencing. *CNS Neurosci Ther*. 2019;25(1):21-29. doi:10.1111/cns.12972

126. Hu C, Li X, Zhao L, et al. Clinical and molecular characterization of pediatric mitochondrial disorders in south of China. *Eur J Med Genet*. 2020;63(8):103898. doi:10.1016/j.ejmg.2020.103898

127. Pavlu-Pereira H, Silva MJ, Florindo C, et al. Pyruvate dehydrogenase complex deficiency: updating the clinical, metabolic and mutational landscapes in a cohort of Portuguese patients. *Orphanet J Rare Dis*. 2020;15(1):298. doi:10.1186/s13023-020-01586-3

128. Ziats MN, Ahmad A, Bernat JA, et al. Genotype-phenotype analysis of 523 patients by genetics evaluation and clinical exome sequencing. *Pediatr Res*. 2020;87(4):735-739. doi:10.1038/s41390-019-0611-5

129. Sen K, Grahame G, Bedoyan JK, Gropman AL. Novel presentations associated with a PDHA1 variant - Alternating hemiplegia in Hemizygote proband and Guillain Barre Syndrome in Heterozygote mother. *Eur J Paediatr Neurol EJPN Off J Eur Paediatr Neurol Soc*. 2021;31:27-30. doi:10.1016/j.ejpn.2021.01.006

130. Kose M, Isik E, Aykut A, et al. The utility of next-generation sequencing technologies in diagnosis of Mendelian mitochondrial diseases and reflections on clinical spectrum. *J Pediatr Endocrinol Metab JPEM*. 2021;34(4):417-430. doi:10.1515/jpem-2020-0410

131. Goergen SK, Alibrahim E, Christie J, et al. The Fetus with Ganglionic Eminence Abnormality: Head Size and Extracranial Sonographic Findings Predict Genetic Diagnoses and Postnatal Outcomes. *AJNR Am J Neuroradiol*. 2021;42(8):1528-1534. doi:10.3174/ajnr.A7131

132. Schon KR, Horvath R, Wei W, et al. Use of whole genome sequencing to determine genetic basis of suspected mitochondrial disorders: cohort study. *BMJ*. 2021;375:e066288. doi:10.1136/bmj-2021-066288

133. Stenton SL, Zou Y, Cheng H, et al. Leigh Syndrome: A Study of 209 Patients at the Beijing Children's Hospital. *Ann Neurol*. 2022;91(4):466-482. doi:10.1002/ana.26313

134. Inui T, Wada Y, Shibuya M, et al. Intravenous ketogenic diet therapy for neonatal-onset pyruvate dehydrogenase complex deficiency. *Brain Dev*. 2022;44(3):244-248. doi:10.1016/j.braindev.2021.11.005

135. Coste T, Aloui C, Petit F, et al. Rare metabolic disease mimicking COL4A1/COL4A2 fetal brain phenotype. *Ultrasound Obstet Gynecol Off J Int Soc Ultrasound Obstet Gynecol*. 2022;60(6):805-811. doi:10.1002/uog.26046

136. Koh HY, Haghighi A, Keywan C, et al. Genetic Determinants of Sudden Unexpected Death in Pediatrics. *Genet Med Off J Am Coll Med Genet*. 2022;24(4):839-850. doi:10.1016/j.gim.2021.12.004

137. Kistol D, Tsygankova P, Krylova T, et al. Leigh Syndrome: Spectrum of Molecular Defects and Clinical Features in Russia. *Int J Mol Sci*. 2023;24(2):1597. doi:10.3390/ijms24021597

138. Wang Y, Fu F, Lei T, et al. Genetic diagnosis of fetal microcephaly at a single tertiary center in China. *Front Genet*. 2023;14:1112153. doi:10.3389/fgene.2023.1112153

139. Zhou H, Fu F, Wang Y, et al. Genetic causes of isolated and severe fetal growth restriction in normal chromosomal microarray analysis. *Int J Gynaecol Obstet Off Organ Int Fed Gynaecol Obstet*. 2023;161(3):1004-1011. doi:10.1002/ijgo.14620

140. Savvidou A, Sofou K, Eklund EA, Aronsson J, Darin N. Manifestations of X-linked pyruvate dehydrogenase complex deficiency in female PDHA1 carriers. *Eur J Neurol*. 2024;31(7):e16283. doi:10.1111/ene.16283

141. Alsehli H, Alshahrani SM, Alzahrani S, et al. Fetal and neonatal outcomes of posterior fossa anomalies: a retrospective cohort study. *Sci Rep*. 2024;14(1):8411. doi:10.1038/s41598-024-59163-8

142. Ferreira T, Polavarapu K, Olimpio C, Paramonov I, Lochmüller H, Horvath R. Variants in mitochondrial disease genes are common causes of inherited peripheral neuropathies. *J Neurol*. 2024;271(6):3546-3553. doi:10.1007/s00415-024-12319-y

143. Westenius E, Conner P, Pettersson M, et al. Whole-genome sequencing in prenatally detected congenital malformations: prospective cohort study in clinical setting. *Ultrasound Obstet Gynecol Off J Int Soc Ultrasound Obstet Gynecol*. 2024;63(5):658-663. doi:10.1002/uog.27592

144. Olimpio C, Paramonov I, Matalonga L, et al. Increased Diagnostic Yield by Reanalysis of Whole Exome Sequencing Data in Mitochondrial Disease. *J Neuromuscul Dis*. 2024;11(4):767-775. doi:10.3233/JND-240020

145. Munn Z, Barker TH, Moola S, et al. Methodological quality of case series studies: an introduction to the JBI critical appraisal tool. *JBI Evid Synth*. 2020;18(10):2127-2133. doi:10.11124/JBISRIR-D-19-00099

146. Richards S, Aziz N, Bale S, et al. Standards and guidelines for the interpretation of sequence variants: a joint consensus recommendation of the American College of Medical Genetics and Genomics and the Association for Molecular Pathology. *Genet Med Off J Am Coll Med Genet*. 2015;17(5):405-424. doi:10.1038/gim.2015.30

147. Tavtigian SV, Harrison SM, Boucher KM, Biesecker LG. Fitting a naturally scaled point system to the ACMG/AMP variant classification guidelines. *Hum Mutat*. 2020;41(10):1734-1737. doi:10.1002/humu.24088

148. Caldovic L, Abdikarim I, Narain S, Tuchman M, Morizono H. Genotype-Phenotype Correlations in Ornithine Transcarbamylase Deficiency: A Mutation Update. *J Genet Genomics Yi Chuan Xue Bao*. 2015;42(5):181-194. doi:10.1016/j.jgg.2015.04.003

149. Germain DP, Oliveira JP, Bichet DG, et al. Use of a rare disease registry for establishing phenotypic classification of previously unassigned GLA variants: a consensus classification system by a multispecialty Fabry disease genotype-phenotype workgroup. *J Med Genet*. 2020;57(8):542-551. doi:10.1136/jmedgenet-2019-106467

150. Peruzzo P, Pavan E, Dardis A. Molecular genetics of Pompe disease: a comprehensive overview. *Ann Transl Med*. 2019;7(13):278. doi:10.21037/atm.2019.04.13

151. Bareil C, Bergougnoux A. CFTR gene variants, epidemiology and molecular pathology. *Arch Pediatr Organe Off Soc Francaise Pediatr*. 2020;27 Suppl 1:eS8-eS12. doi:10.1016/S0929-693X(20)30044-0

152. Hillert A, Anikster Y, Belanger-Quintana A, et al. The Genetic Landscape and Epidemiology of Phenylketonuria. *Am J Hum Genet*. 2020;107(2):234-250. doi:10.1016/j.ajhg.2020.06.006

153. Cheng J, Novati G, Pan J, et al. Accurate proteome-wide missense variant effect prediction with AlphaMissense. *Science*. 2023;381(6664):eadg7492. doi:10.1126/science.adg7492

154. Vervoort R, Lennon A, Bird AC, et al. Mutational hot spot within a new RPGR exon in X-linked retinitis pigmentosa. *Nat Genet*. 2000;25(4):462-466. doi:10.1038/78182

155. Chen S, Francioli LC, Goodrich JK, et al. A genomic mutational constraint map using variation in 76,156 human genomes. *Nature*. 2024;625(7993):92-100. doi:10.1038/s41586-023-06045-0

156. Cioni G, Sgandurra G. Normal psychomotor development. *Handb Clin Neurol*. 2013;111:3-15. doi:10.1016/B978-0-444-52891-9.00001-4

157. Pérez Pico AM, Mingorance Álvarez E, Villar Rodríguez J, Mayordomo Acevedo R. Differences in Hygiene Habits among Children Aged 8 to 11 Years by Type of Schooling. *Child Basel Switz*. 2022;9(2):129. doi:10.3390/children9020129

158. Arachchige CNPG, Prendergast LA, Staudte RG. Robust analogs to the coefficient of variation. *J Appl Stat*. 2022;49(2):268-290. doi:10.1080/02664763.2020.1808599

PRISMA 2020 Checklist

Section and Topic	Item #	Checklist item	Location where item is reported
**TITLE**			
Title	1	Identify the report as a systematic review.	NR
**ABSTRACT**			
Abstract	2	See the PRISMA 2020 for Abstracts checklist.	NR
**INTRODUCTION**			
Rationale	3	Describe the rationale for the review in the context of existing knowledge.	Page 7
Objectives	4	Provide an explicit statement of the objective(s) or question(s) the review addresses.	Page 7
**METHODS**			
Eligibility criteria	5	Specify the inclusion and exclusion criteria for the review and how studies were grouped for the syntheses.	Page 8
Information sources	6	Specify all databases, registers, websites, organisations, reference lists and other sources searched or consulted to identify studies. Specify the date when each source was last searched or consulted.	Page 8
Search strategy	7	Present the full search strategies for all databases, registers and websites, including any filters and limits used.	Page 8
Selection process	8	Specify the methods used to decide whether a study met the inclusion criteria of the review, including how many reviewers screened each record and each report retrieved, whether they worked independently, and if applicable, details of automation tools used in the process.	Page 8
Data collection process	9	Specify the methods used to collect data from reports, including how many reviewers collected data from each report, whether they worked independently, any processes for obtaining or confirming data from study investigators, and if applicable, details of automation tools used in the process.	Page 8
Data items	10a	List and define all outcomes for which data were sought. Specify whether all results that were compatible with each outcome domain in each study were sought (e.g. for all measures, time points, analyses), and if not, the methods used to decide which results to collect.	Page 8
	10b	List and define all other variables for which data were sought (e.g. participant and intervention characteristics, funding sources). Describe any assumptions made about any missing or unclear information.	NR
Study risk of bias assessment	11	Specify the methods used to assess risk of bias in the included studies, including details of the tool(s) used, how many reviewers assessed each study and whether they worked independently, and if applicable, details of automation tools used in the process.	Page 8
Effect measures	12	Specify for each outcome the effect measure(s) (e.g. risk ratio, mean difference) used in the synthesis or presentation of results.	Not applied
Synthesis methods	13a	Describe the processes used to decide which studies were eligible for each synthesis (e.g. tabulating the study intervention characteristics and comparing against the planned groups for each synthesis (item #5)).	Not applied
	13b	Describe any methods required to prepare the data for presentation or synthesis, such as handling of missing summary statistics, or data conversions.	Page 9, 10
	13c	Describe any methods used to tabulate or visually display results of individual studies and syntheses.	Not applied
	13d	Describe any methods used to synthesize results and provide a rationale for the choice(s). If meta-analysis was performed, describe the model(s), method(s) to identify the presence and extent of statistical heterogeneity, and software package(s) used.	Page 9, 10
	13e	Describe any methods used to explore possible causes of heterogeneity among study results (e.g. subgroup analysis, meta-regression).	Page 10-17
	13f	Describe any sensitivity analyses conducted to assess robustness of the synthesized results.	Not applied
Reporting bias assessment	14	Describe any methods used to assess risk of bias due to missing results in a synthesis (arising from reporting biases).	Not applied
Certainty assessment	15	Describe any methods used to assess certainty (or confidence) in the body of evidence for an outcome.	Not applied

PRISMA 2020 Checklist *(continued)*

Section and Topic	Item #	Checklist item	Location where item is reported
**RESULTS**			
Study selection	16a	Describe the results of the search and selection process, from the number of records identified in the search to the number of studies included in the review, ideally using a flow diagram.	Figure 1
	16b	Cite studies that might appear to meet the inclusion criteria, but which were excluded, and explain why they were excluded.	NR
Study characteristics	17	Cite each included study and present its characteristics.	Suppl. Table 1-2
Risk of bias in studies	18	Present assessments of risk of bias for each included study.	Not applied
Results of individual studies	19	For all outcomes, present, for each study: (a) summary statistics for each group (where appropriate) and (b) an effect estimate and its precision (e.g. confidence/credible interval), ideally using structured tables or plots.	Not applied
Results of syntheses	20a	For each synthesis, briefly summarise the characteristics and risk of bias among contributing studies.	Not applied
	20b	Present results of all statistical syntheses conducted. If meta-analysis was done, present for each the summary estimate and its precision (e.g. confidence/credible interval) and measures of statistical heterogeneity. If comparing groups, describe the direction of the effect.	Page 10-17
	20c	Present results of all investigations of possible causes of heterogeneity among study results.	Not applied
	20d	Present results of all sensitivity analyses conducted to assess the robustness of the synthesized results.	Not applied
Reporting biases	21	Present assessments of risk of bias due to missing results (arising from reporting biases) for each synthesis assessed.	Not applied
Certainty of evidence	22	Present assessments of certainty (or confidence) in the body of evidence for each outcome assessed.	Not applied
**DISCUSSION**			
Discussion	23a	Provide a general interpretation of the results in the context of other evidence.	Page 18-24
	23b	Discuss any limitations of the evidence included in the review.	Page 24
	23c	Discuss any limitations of the review processes used.	NR
	23d	Discuss implications of the results for practice, policy, and future research.	Page 24
**OTHER INFORMATION**			
Registration and protocol	24a	Provide registration information for the review, including register name and registration number, or state that the review was not registered.	Page 8
	24b	Indicate where the review protocol can be accessed, or state that a protocol was not prepared.	Page 8
	24c	Describe and explain any amendments to information provided at registration or in the protocol.	Not applied
Support	25	Describe sources of financial or non-financial support for the review, and the role of the funders or sponsors in the review.	Page 25-26
Competing interests	26	Declare any competing interests of review authors.	Page 26
Availability of data, code and other materials	27	Report which of the following are publicly available and where they can be found: template data collection forms; data extracted from included studies; data used for all analyses; analytic code; any other materials used in the review.	Page 8, 10

From: Page MJ, McKenzie JE, Bossuyt PM, Boutron I, Hoffmann TC, Mulrow CD, et al. The PRISMA 2020 statement: an updated guideline for reporting systematic reviews. BMJ 2021;372:n71. doi: 10.1136/bmj.n71. This work is licensed under CC BY 4.0. NR – not reported.
